# Plasma proteomics for risk prediction of Alzheimer's disease in the general population

**DOI:** 10.1111/acel.14330

**Published:** 2024-09-09

**Authors:** Sisi Yang, Ziliang Ye, Panpan He, Yuanyuan Zhang, Mengyi Liu, Chun Zhou, Yanjun Zhang, Xiaoqin Gan, Yu Huang, Hao Xiang, Xianhui Qin

**Affiliations:** ^1^ Division of Nephrology, Nanfang Hospital Southern Medical University Guangzhou China; ^2^ National Clinical Research Center for Kidney Disease Guangzhou China; ^3^ State Key Laboratory of Organ Failure Research Guangzhou China; ^4^ Guangdong Provincial Institute of Nephrology Guangzhou China; ^5^ Guangdong Provincial Key Laboratory of Renal Failure Research Guangzhou China

**Keywords:** Alzheimer's disease, dementia, enrichment analyses, protein–protein interaction network, proteomics

## Abstract

We aimed to develop and validate a protein risk score for predicting Alzheimer's disease (AD) and compare its performance with a validated clinical risk model (Cognitive Health and Dementia Risk Index for AD [CogDrisk‐AD]) and apolipoprotein E (APOE) genotypes. The development cohort, consisting of 35,547 participants from England in the UK Biobank, was randomly divided into a 7:3 training–testing ratio. The validation cohort included 4667 participants from Scotland and Wales in the UK Biobank. In the training set, an AD protein risk score was constructed using 31 proteins out of 2911 proteins. In the testing set, the AD protein risk score had a C‐index of 0.867 (95% CI, 0.828, 0.906) for AD prediction, followed by CogDrisk‐AD risk factors (C‐index, 0.856; 95% CI, 0.823, 0.889), and APOE genotypes (C‐index, 0.705; 95% CI, 0.660, 0.750). Adding the AD protein risk score to CogDrisk‐AD risk factors (C‐index increase, 0.050; 95% CI, 0.008, 0.093) significantly improved the predictive performance for AD. However, adding CogDrisk‐AD risk factors (C‐index increase, 0.040; 95% CI, −0.007, 0.086) or APOE genotypes (C‐index increase, 0.000; 95% CI, −0.054, 0.055) to the AD protein risk score did not significantly improve the predictive performance for AD. The top 10 proteins with the highest coefficients in the AD protein risk score contributed most of the predictive power for AD risk. These results were verified in the external validation cohort. EGFR, GFAP, and CHGA were identified as key proteins within the protein network. Our result suggests that the AD protein risk score demonstrated a good predictive performance for AD risk.

## Graphical abstract

An Alzheimer's disease (AD) protein risk score was constructed using 31 proteins out of 2911 proteins. The AD protein risk score had a C‐index of 0.867 (95% CI, 0.828, 0.906) for AD prediction, followed by CogDrisk‐AD risk factors, and APOE genotypes. Our result suggests that the AD protein risk score demonstrated a good predictive performance for AD risk.

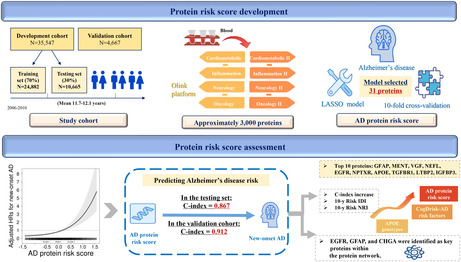

**Abbreviations**
AD: Alzheimer's disease
ANU‐ADRI: Australian National University‐Alzheimer Disease Risk Index
APOE: apolipoprotein E
AUC: area under curve
BMI: body mass index
CAIDE: Cardiovascular Risk Factors Aging and Dementia
CHGA: Chromogranin‐A
CIs: confidence intervals
CogDrisk‐AD: Cognitive Health and Dementia Risk Index for Alzheimer's disease
EGFR: epidermal growth factor receptor
FDR: false discovery rate
GFAP: glial fibrillary acidic protein
GO: Gene ontology
HRs: hazard ratios
ICD: International Classification of Disease
IDI: integrated discrimination improvement
IGFBP3: insulin‐like growth factor‐binding protein 3
KEGG: Kyoto Encyclopedia of Genes and Genomes
LASSO: least absolute shrinkage and selection operator
LTBP2: latent‐transforming growth factor beta‐binding protein 2
MENT: methylated in normal thymocytes protein
NEFL: neurofilament light polypeptide
NPTXR: neuronal pentraxin receptor
NRI: net reclassification improvement
PPI: protein–protein interaction
TBI: traumatic brain injury
TGFBR1: TGF‐beta receptor type‐1
UKB‐PPP: UK Biobank Pharma Proteomics Project
VGF: neurosecretory protein VGF

## 1 INTRODUCTION

Alzheimer's disease (AD) is a kind of neurodegenerative disease involving multiple biological processes, which is characterized by high incidence, protracted course, high disability rate, and high mortality rate (Prince et al., 2016). Despite extensive research underway, there is currently a lack of effective drug treatments that can cure or slow the progression of AD (Geng et al., 2023). Therefore, accurate identification of people at high risk of AD and early prevention would help alleviate the burden of AD on both individuals and society.

Proteins regulate biological processes and can integrate the influence of genes with the environment, age, behaviors, comorbidities, and treatments (Williams et al., 2019). Large‐scale plasma proteomic analysis can provide valuable insights into both specific biomarkers and the intricate molecular mechanisms underlying diseases (Emilsson et al., 2018). However, although a previous study has examined the performance of proteomics in predicting future dementia including AD (Guo et al., 2024), this study (Guo et al., 2024) established a proteomic risk score based on only 1463 detected proteins (the initial round of protein measurements in the UK Biobank) and did not compare its prediction performance with validated clinical risk model for AD risk, nor did they establish a testing set and a validation cohort. Therefore, the association between proteomics and AD risk requires more studies covering a larger number of proteins.

Over the past few years, although a biomarker‐based amyloid/tau/ neurodegeneration (A/T/N) framework has been established in AD, it has been limited by its invasiveness, high cost, and failure to comprehensively encompass the entire spectrum of AD pathology (Huang et al., 2022). In addition, many existing prediction models for AD often contain information that is not generally available in routine clinical practice (such as advanced imaging (Barnes et al., 2009; Stephan et al., 2015), cognitive testing (Barnes et al., 2009; Chary et al., 2013), or cerebrospinal fluid biomarkers [Zhan et al., 2015]), which limits their clinical application. The Cognitive Health and Dementia Risk Index for AD (CogDrisk‐AD) is a recently updated and validated predictive model for AD (Anstey et al., 2022; Huque et al., 2023). It was developed using data from up‐to‐date systematic evidence and covered the widest range of established clinical risk factors of AD than other AD risk predictive scores, such as the Australian National University‐Alzheimer Disease Risk Index (ANU‐ADRI) and the Cardiovascular Risk Factors Aging and Dementia (CAIDE) (Huque et al., 2023). Additionally, the apolipoprotein E (APOE) genotype has been identified as being significantly associated with the dementia risk (Xu et al., 2023). We hypothesize that a multiple protein risk score derived from routine blood‐based testing may improve the accuracy of AD risk prediction based on traditional clinical risk factors and genetic risk factors for AD. To date, this hypothesis has not been investigated.

To address the above important knowledge gaps, using data from the UK Biobank Pharma Proteomics Project (UKB‐PPP), a study that involved large‐scale measurements of approximately 3000 proteins in 50,000 participants, we aimed to develop and validate a multi‐protein risk score for assessing the risk of AD, and compare its predictive performance with the latest validated clinical risk model (CogDrisk‐AD) and APOE genotypes.

## 2 METHODS

### 2.1 Study populations

The UKB is a large prospective cohort study that recruited over half a million participants aged 40–69 years from 2006 to 2010 in 22 recruitment centers across the United Kingdom (Sudlow et al., 2015; Yang et al., 2023; Zhou et al., 2022). Participants completed a series of computer‐based questionnaires on lifestyles and medical history, underwent a standardized portfolio of clinical measurements, and provided biological samples. The UKB was approved by the North West Center for Research Ethics Committee (11/NW/0382) and all participants gave written informed consent before recruitment.

UKB‐PPP is a subset study in which plasma proteomic measurements were performed in a sample size of more than 50,000 UKB participants. The included sample was mainly a randomly selected population from the UKB (Sun et al., 2023) and is therefore highly representative of the overall UKB participants. In the current study, participants had any form of dementia at baseline (defined by a well‐validated algorithm in the UKB: self‐reported dementia/AD/cognitive impairment, or diagnosed using International Classification of Disease, 9th Revision [ICD‐9] codes 290/291.2/294.1/331, or ICD‐10 codes A81.0/F00/F01/F02/F03/F05.1/F10.6/G30/G31/I67.3; UK Biobank, n.d.) (*n* = 51), or had missing APOE gene data or sex discrepancies between the self‐reported and X‐chromosome heterozygosity (*n* = 1871), or had missing data of established risk factors for AD (including all the clinical risk factors in the CogDrisk‐AD) (Huque et al., 2023) at baseline (*n* = 10,893) were excluded. Finally, a total of 40,214 individuals were included in the analyses, of which, 35,547 participants from the England area were used to develop a multi‐protein risk score for new‐onset AD (development cohort), and 4667 participants from the Scotland and Wales area were used to externally validate the model (validation cohort). In the development cohort, participants were randomly divided into a training set (70% of the participants, *n* = 24,882) and a testing set (30% of the participants, *n* = 10,665) (Figure S1).

### 2.2 Blood proteomics measurements

Proteomic profiling of blood plasma samples was performed by the Olink platform, using the antibody‐based Olink Explore 3072 proximity extension assay (PEA). The UKB laboratory team executed the randomization and plating of all samples before delivery. The processing of these samples occurred across three NovaSeq 6000 Sequencing Systems. Olink's facilities implemented rigorous quality control measures. Olink offers a range of assay panels, with each Olink's panel comprising a set of predefined protein biomarkers validated through a comprehensive literature review and expert evaluation (Olink website, n.d.). In the initial round of measurements, 1463 unique proteins across Olink's panels (cardiometabolic, inflammation, neurology, and oncology) in the UKB‐PPP plasma sample collected at baseline were assessed. In the latest round of measurements, 1460 unique proteins across Olink's panels (cardiometabolic II, inflammation II, neurology II, and oncology II) were evaluated. Thus, a total of 2923 unique proteins were measured for each plasma sample. Protein concentrations were normalized, resulting in the generation of inverse‐rank normalized protein expression (NPX) values for each protein, measured on a log2 scale using Olink's relative protein quantification unit. Details of the proteomics assays, data processing, and quality control are provided in previous publications (Sun et al., 2023).

### 2.3 Clinical risk factors for AD

Clinical risk factors were selected based on the CogDrisk‐AD risk model (Huque et al., 2023). The CogDrisk‐AD model (Huque et al., 2023) included 16 established clinical risk factors for increased or decreased risk of AD: age, sex, education, obesity, diabetes, depression, high cholesterol, traumatic brain injury (TBI), smoking, loneliness, physical activity, cognitive activity, fish intake, hypertension, stroke, and occupational pesticides exposure.

Information on clinical risk factors was collected using standardized questionnaires at baseline, including age, sex, education, smoking status, physical activity, fish intake (oily fish and non‐oily fish), cognitive activity (adult education class and attending a religious), loneliness, depression, and occupational pesticides exposure. Anthropometric measurements including height, weight, and blood pressure were assessed by trained nurses. Body mass index (BMI) was calculated as weight (kg)/height (m)^2^. Obesity was defined as BMI ≥30 kg/m^2^. Prevalent hypertension, diabetes, high cholesterol, stroke, and TBI were diagnosed by self‐reported medical history or health records at baseline.

### 2.4 APOE genotype ascertainment

Participants were genotyped using two highly similar genotyping arrays: UK BiLEVE or UKB Axiom arrays. Details about genotyping, imputation, and quality control procedures in the UKB study have been previously documented (Bycroft et al., 2018). The APOE ε4‐carrying status was determined based on the genetic variants of rs429358 and rs7412. Participants who possessed 1 or 2 ε4 alleles were defined as APOE ε4 carriers, while those without any ε4 alleles were defined as APOE ε4 noncarriers (Zhang et al., 2021).

### 2.5 Assessment of study outcome

The study outcome was new‐onset AD, ascertained from algorithmically‐defined outcomes in the UKB (Field ID = 42,020), which incorporated information from UKB's baseline assessment data, along with linked data from hospital admissions (diagnoses and procedures) and death registries (UK Biobank, n.d.; Wang et al., 2024).

The follow‐up time was calculated from the date of first assessment until the first date of new‐onset AD, date of death, date of loss to follow‐up, or end of follow‐up (England: September 30, 2021; Scotland: July 31, 2021; Wales: February 28, 2018), whichever came first.

### 2.6 Statistical analysis

#### 2.6.1 Protein risk score development

The AD protein risk score was constructed in the training set (including 24,882 participants; 249 events) and tested in the testing set (including 10,665 participants; 107 events) and validation cohort (including 4667 participants; 36 events). Differences in population characteristics between the validation cohort and development cohort and between the training set and testing set in the development cohort were compared using analysis of variance (ANOVA) for continuous variables and chi‐squared tests for categorical variables.

Proteins (*n* = 12) with a missing rate of more than 20% were excluded, while the remaining 2911 proteins with missing measurements were imputed using mean values (Sun et al., 2023). Given the high dimensionality of proteomic data, least absolute shrinkage and selection operator (LASSO) Cox regression model was used for feature selection to alleviate overfitting and improve the interpretability of the model. The protein risk score, serving as an integrated framework that captures the influence of genetic and environmental factors on AD risk, was constructed using the 2911 protein levels, age, and sex in the LASSO Cox regression model (Helgason et al., 2023). During the training procedures, age and sex were included as covariates in the model, while the Lasso penalty was employed to effectively shrink less significant coefficients of proteins to zero thus facilitating the selection of the most relevant protein features. The whole training set was utilized along with 10‐fold cross‐validation and the lowest error plus one standard deviation (to minimize overfitting) in the lasso objective function to control model complexity (number of included proteins) by shrinkage parameter λ (Figure S2). Subsequently, coefficients for the proteins in the protein risk score were derived (Table S1). We used the R package *glmnet* to train the model. For all individuals with available protein measurements in the testing set and validation cohort, the protein risk score was calculated as the linear part of the Cox model, excluding age and sex, using the protein score coefficients obtained from the training set. In addition, to compare candidate proteins between dementia and AD, a LASSO Cox regression model was also employed with dementia as the outcome variable.

#### 2.6.2 Protein risk score assessment

In the testing set and validation cohort, the hazard ratios (HRs) and 95% confidence intervals (CIs) for the risk of new‐onset AD associated with AD protein risk score were estimated by Cox proportional hazard models and visualized using curve fitting. The discriminant ability of the model was evaluated by the Harrell C index, and CIs were estimated with the bootstrap method. The C‐index value ranges from 0 to 1, with a higher value indicating a better ability to accurately distinguish individuals who will or will not develop AD. The continuous 10‐year net reclassification improvement (NRI) and the 10‐year integrated discrimination improvement (IDI) were used to assess reclassification performance and improvement in discrimination using the R package *nricens* and *survIDINRI*. Using half of the AD incident rate observed in the current study as a threshold (Cook, 2018), we also calculated the 10‐year categorical NRI. A positive NRI and IDI value indicated improved reclassification and predictive accuracy compared to the reference model, while a negative value indicated worse performance. Bootstrapping was used to estimate CIs.

#### 2.6.3 Protein enrichment and network analyses

To interpret the underlying biological pathway associated with incident AD, the proteins derived from the AD protein risk score developed by the LASSO model were fed into the Database for Annotation, Visualization and Integrated Discovery (DAVID) website (https://david.ncifcrf.gov/) for enrichment analyses. Gene Ontology (GO) function enrichment analyses and Kyoto Encyclopedia of Genes and Genomes (KEGG) pathway enrichment analyses were employed. The protein–protein interaction (PPI) network was analyzed using the Search Tool for the Retrieval of Interacting Genes/Proteins (STRING) website (https://cn.string‐db.org/) to comprehend the complexity of protein–protein interaction networks and identify key proteins. In addition, Cox survival models adjusted for age and sex were used to assess the association between proteins from different protein panels (731 proteins from the cardiometabolic/cardiometabolic II panel, 724 from inflammation/inflammation II, 726 from neurology/neurology II, and 730 from oncology/oncology II) with the risk of incident AD. False discovery rate (FDR) correction was applied to adjust for multiple tests of proteins within each protein panel.

A two‐tailed *p*‐value <0.05 was considered statistically significant in all analyses. Analyses were performed using software R (Version 4.3.0).

## 3 RESULTS

### 3.1 Population characteristics

In the development cohort, a total of 35,547 eligible participants were enrolled, with a mean age of 56.8 (SD, 8.2) years, and 47.1% were male. During a mean follow‐up of 12.1 years, there were 356 (1.0%) cases of new‐onset AD, with 249 (1.0%) cases in the training set and 107 (1.0%) cases in the testing set. Participants in the training and testing sets have similar characteristics (Table 1).

**TABLE 1:** General characteristics of study participants.^a^

Characteristics	All participants		Development cohort	
	Development cohort	Validation cohort	Training set	Testing set
N	35,547	4667	24,882	10,665
Age, years	56.8 (8.2)	56.4 (8.1)	56.7 (8.2)	56.9 (8.1)
Male, *n* (%)	16,726 (47.1)	2156 (46.2)	11,759 (47.3)	4967 (46.6)
Education ≥10 years, *n* (%)	20,434 (57.5)	2863 (61.3)	14,401 (57.9)	6033 (56.6)
Obesity, *n* (%)	8402 (23.6)	1193 (25.6)	5875 (23.6)	2527 (23.7)
Diabetes, *n* (%)	2185 (6.1)	273 (5.8)	1544 (6.2)	641 (6.0)
Depression, *n* (%)	10,541 (29.7)	1358 (29.1)	7392 (29.7)	3149 (29.5)
High cholesterol, *n* (%)	17,491 (49.2)	2253 (48.3)	12,197 (49.0)	5294 (49.6)
Traumatic brain injury, *n* (%)	53 (0.1)	5 (0.1)	39 (0.2)	14 (0.1)
Non‐smoker, *n* (%)	19,201 (54.0)	2616 (56.1)	13,499 (54.3)	5702 (53.5)
Optimal physical activity, *n* (%)	20,443 (57.5)	2574 (55.2)	14,350 (57.7)	6093 (57.1)
Loneliness, *n* (%)	6376 (17.9)	807 (17.3)	4470 (18.0)	1906 (17.9)
Cognitive activity, *n* (%)				
Adult education class	942 (2.7)	132 (2.8)	656 (2.6)	286 (2.7)
Attending a religious group	3422 (9.6)	564 (12.1)	2397 (9.6)	1025 (9.6)
Fish serves per week, *n* (%)				
Oily fish	20,164 (56.7)	2500 (53.6)	14,189 (57.0)	5975 (56.0)
Non‐oily fish	23,602 (66.4)	3152 (67.5)	16,473 (66.2)	7129 (66.8)
Hypertension, *n* (%)	19,575 (55.1)	2707 (58.0)	13,672 (54.9)	5903 (55.3)
Stroke, *n* (%)	638 (1.8)	87 (1.9)	449 (1.8)	189 (1.8)
Occupational pesticide exposure, *n* (%)				
Sometimes/often	161 (0.5)	27 (0.6)	103 (0.4)	58 (0.5)
Rarely/never	8912 (25.1)	1037 (22.2)	6227 (25.0)	2685 (25.2)
Uncertain	26,280 (73.9)	3577 (76.6)	18,408 (74.0)	7872 (73.8)
APOE ε4 carrier	9582 (27.0)	1247 (26.7)	6685 (26.9)	2897 (27.2)
Incident Alzheimer's disease, *n* (%)	356 (1.0)	36 (0.8)	249 (1.0)	107 (1.0)
Follow‐up time, years	12.1 (1.9)	11.7 (2.4)	12.1 (1.9)	12.1 (1.9)

Abbreviations: APOE, apolipoprotein E; AD, Alzheimer's disease.

^a^ Continuous and categorical variables are expressed as mean (SD) or *n* (%), accordingly.

The validation cohort comprised 4667 participants, with a mean follow‐up of 11.7 years, during which 36 (0.8%) new‐onset cases of AD were recorded. Compared to those in the development cohort, participants in the validation cohort were younger, had higher levels of education and cognitive activity, lower levels of physical activity and oily fish intake, and a higher prevalence of obesity and non‐smoking status (Table 1).

### 3.2 Association between AD protein risk score and risk of new‐onset AD

In the training set, an AD protein risk score containing 31 proteins was constructed out of 2911 measured plasma proteins using the LASSO Cox regression model (Figure S2, Table S1). In the Cox regression analysis of the identified 31 proteins, all 31 proteins were significantly associated with the risk of new‐onset AD, with 14 proteins positively associated with AD risk and 17 proteins negatively associated with AD risk (Table S2). Additionally, in the LASSO model using dementia as the outcome, 34 proteins were identified, 20 of which overlapped with the 31 identified AD‐related proteins (Figure S3).

**FIGURE 1:**
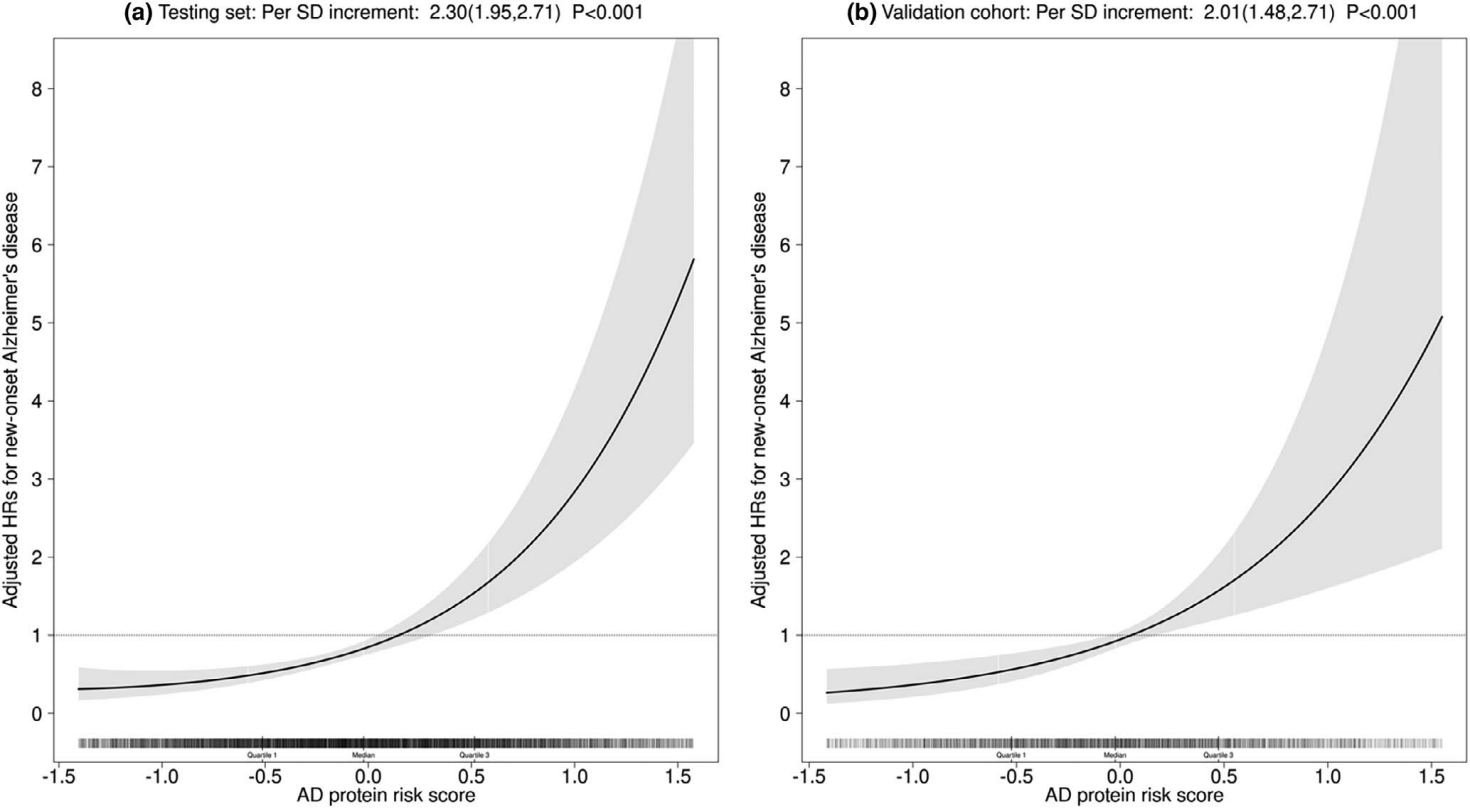
The dose–response association between the Alzheimer's disease (AD) protein risk score and new‐onset AD in (a) the testing set of the development cohort and (b) the validation cohort. Adjusted for APOE genotypes and clinical risk factors in the CogDrisk‐AD including age, sex, education, obesity, diabetes, depression, high cholesterol, traumatic brain injury, smoking, loneliness, physical activity, cognitive activity, fish intake, hypertension, stroke, and occupational pesticides exposure.

In the testing set, after adjusting for APOE genotypes and the clinical factors in the CogDrisk‐AD risk model, the AD protein risk score was significantly positively associated with the risk of new‐onset AD (per SD increment: HR, 2.30; 95% CI, 1.95, 2.71) (Figure 1a). Similar results were found in the validation cohort (per SD increment: HR, 2.01; 95% CI, 1.48, 2.71) (Figure 1b).

### 3.3 Discrimination ability assessment

In the testing set, the C‐index of AD protein risk score predicting AD risk was 0.867 (95% CI, 0.828, 0.906), which was comparable to that of CogDrisk‐AD risk factors (C‐index, 0.856; 95% CI, 0.823, 0.889), but significantly superior to that of APOE genotypes (C‐index, 0.705; 95% CI, 0.660, 0.750). Adding the AD protein risk score to the model including CogDrisk‐AD risk factors (C‐index from 0.856 to 0.907; C‐index increase, 0.050; 95% CI, 0.008, 0.093) significantly improved the predictive performance for new‐onset AD. However, adding CogDrisk‐AD risk factors (C‐index from 0.867 to 0.907; C‐index increase, 0.040; 95% CI, −0.007, 0.086) or APOE genotypes (C‐index from 0.867 to 0.867; C‐index increase, 0; 95% CI, −0.054, 0.055) to the model including the AD protein risk score did not significantly improve the predictive performance for new‐onset AD (Table 2). That is, the AD protein risk score alone is already an AD risk prediction model with good predictive performance (Table 2). Similar results were found in the validation cohort (Table 2).

**TABLE 2:** The Alzheimer's disease (AD) risk prediction performance of AD protein risk score in the testing set of the development cohort and in the validation cohort.^a^

Model	C‐index	Comparison to reference models		
		C‐index increase (95% CI)	10‐year risk continuous NRI (95% CI)	10‐year risk IDI (95% CI)
Testing set in the development cohort				
Reference model: AD protein risk score	0.867 (0.828,0.906)			
CogDrisk‐AD risk factors	0.856 (0.823,0.889)	−0.011 (−0.062, 0.040)	−0.060 (−0.285, 0.139)	−0.007 (−0.021, 0.014)
APOE genotypes	0.705 (0.660,0.750)	−0.162 (−0.221, −0.102)	−0.219 (−0.409, −0.110)	−0.019 (−0.035, −0.004)
CogDrisk‐AD risk factors + AD protein risk score	0.907 (0.881,0.933)	0.040 (−0.007, 0.086)	0.272 (0.037, 0.442)	0.021 (0.005, 0.044)
APOE genotype + AD protein risk score	0.867 (0.829,0.905)	0 (−0.054, 0.055)	0.212 (−0.040, 0.429)	0.017 (0.003, 0.026)
Reference model: CogDrisk‐AD risk factors	0.856 (0.823,0.889)			
CogDrisk‐AD risk factors + AD protein risk score	0.907 (0.881,0.933)	0.050 (0.008, 0.093)	0.351 (0.176, 0.424)	0.028 (0.007, 0.059)
CogDrisk‐AD risk factors + APOE genotype	0.885 (0.855,0.914)	0.029 (−0.016, 0.073)	0.357 (0.103, 0.443)	0.018 (0.003, 0.038)
Validation cohort				
Reference model: AD protein risk score	0.912 (0.870,0.953)			
CogDrisk‐AD risk factors	0.922 (0.888,0.955)	0.010 (−0.069, 0.039)	−0.019 (−0.258, 0.384)	0.001 (−0.007, 0.087)
APOE genotypes	0.746 (0.672,0.820)	−0.166 (−0.208, −0.098)	−0.134 (−0.461, 0.003)	−0.011 (−0.018, 0)
CogDrisk‐AD risk factors + AD protein risk score	0.950 (0.929,0.970)	0.038 (−0.021, 0.057)	0.112 (−0.255, 0.770)	0.025 (0.007, 0.204)
APOE genotype + AD protein risk score	0.899 (0.846,0.953)	−0.012 (−0.041, 0.008)	0.112 (−0.254, 0.770)	0.023 (0.004, 0.032)
Reference model: CogDrisk‐AD risk factors	0.922 (0.888,0.955)			
CogDrisk‐AD risk factors + AD protein risk score	0.950 (0.929,0.970)	0.028 (0.011, 0.056)	0.156 (0.070, 0.726)	0.024 (0.003, 0.130)
CogDrisk‐AD risk factors + APOE genotype	0.951 (0.932,0.970)	0.030 (0.022, 0.056)	0.157 (−0.087, 0.720)	0.016 (0.002, 0.156)

Abbreviations: APOE, apolipoprotein E; CogDrisk‐AD, Cognitive Health and Dementia Risk Index for Alzheimer's disease; INI, integrated discrimination improvement; NRI, net reclassification improvement.

^a^ CogDrisk‐AD risk factors represent the risk factors included in the CogDrisk‐AD, including age, sex, education, obesity, diabetes, depression, high cholesterol, traumatic brain injury, smoking, loneliness, physical activity, cognitive activity, fish intake, hypertension, stroke, and occupational pesticides exposure.

**FIGURE 2:**
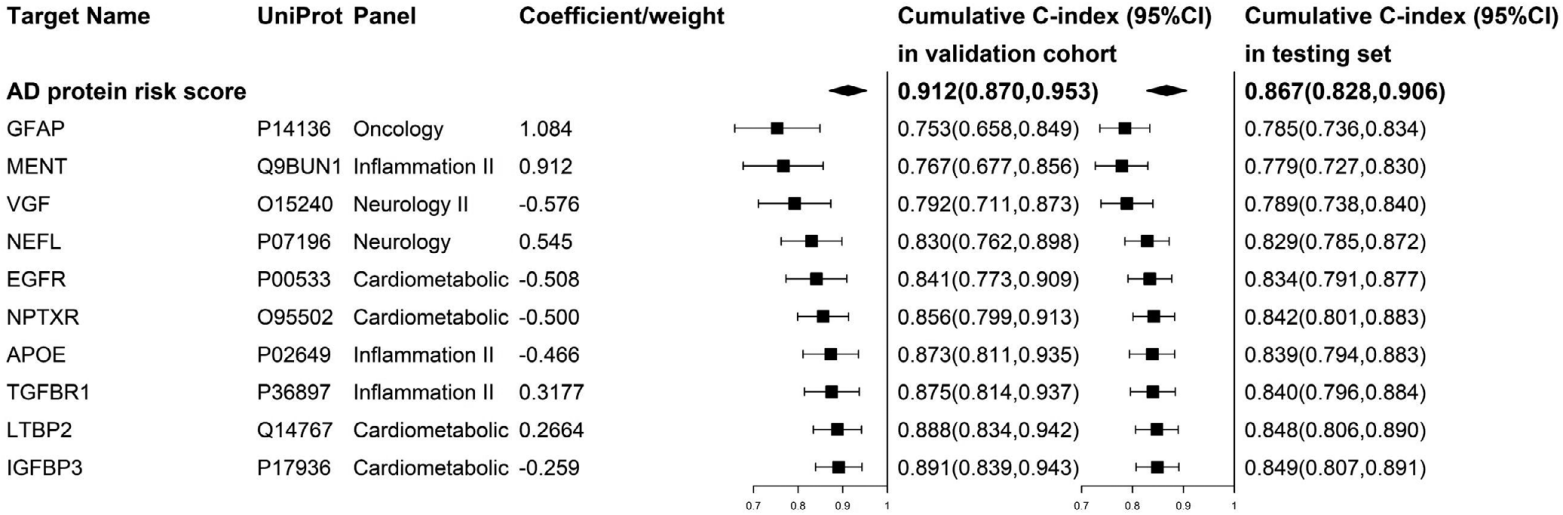
The cumulative C‐index of the top 10 proteins with the largest absolute coefficients in the Alzheimer's disease (AD) protein risk score for new‐onset AD in the testing set of the development cohort and in the validation cohort. Based on the 31 candidate proteins, the top 10 proteins with the largest absolute coefficients were included in the AD prediction model sequentially (in order of coefficient from the largest to smallest).

Of the top 10 proteins with the largest absolute coefficients in the AD protein risk score, five proteins (glial fibrillary acidic protein [GFAP], methylated in normal thymocytes protein [MENT], neurofilament light polypeptide [NEFL], TGF‐beta receptor type‐1 [TGFBR1] and latent‐transforming growth factor beta‐binding protein 2 [LTBP2]) were positively associated with the risk of new‐onset AD, while the other five proteins (neurosecretory protein VGF [VGF], epidermal growth factor receptor [EGFR], neuronal pentraxin receptor [NPTXR], APOE and insulin‐like growth factor‐binding protein 3 [IGFBP3]) were negatively associated with the risk of new‐onset AD (Table S1). When the top 10 proteins with the largest absolute coefficients in the AD protein risk score were sequentially included (ordered from the largest to the smallest coefficient), the cumulative C‐index for predicting AD events gradually increased. In the validation cohort, the cumulative C‐index ranged from 0.753 to 0.891, while in the testing set, it ranged from 0.785 to 0.849 (Figure 2, Table S3). That is, the top 10 proteins in the AD protein risk score contributed the most to the ability of the AD protein risk score to predict AD risk.

### 3.4 Reclassification ability assessment

In the testing set, the reclassification ability of AD protein risk score for AD risk was comparable to that of cogrisk‐AD risk factors, and significantly better than that of APOE genotype (Table 2). Adding the AD protein risk score to the model of CogDrisk‐AD risk factors significantly improved the reclassification ability for predicting new‐onset AD (10‐year continuous NRI, 0.351; 95% CI, 0.176, 0.424; 10‐year IDI, 0.028; 95% CI, 0.007, 0.059) (Table 2). Similar results were found in the validation cohort (Table 2).

Of the 107 cases in the testing set, 68 developed incident AD within 10 years. When the AD risk threshold was set at 0.5% (half of AD incident rate in the study), the AD protein risk score correctly identified 86.8% of AD cases, which was comparable to the CogDrisk‐AD risk factors (86.8%; 10‐year categorical NRI, −0.003; 95% CI, −0.150, 0.080), and higher than APOE genotypes (66.17%; 10‐year categorical NRI, 0.216; 95% CI, 0.073, 0.330). In the validation cohort (19 cases within 10 years), the AD protein risk score correctly diagnosed 89.5% of AD cases, followed by CogDrisk‐AD risk factors (84.2%; 10‐year categorical NRI, 0.049; 95% CI, −0.347, 0.235), and APOE genotypes (73.7%; 10‐year categorical NRI, 0.164; 95% CI, 0.117, 0.376) (Table S4).

### 3.5 Enrichment and network analyses of candidate proteins

**FIGURE 3:**
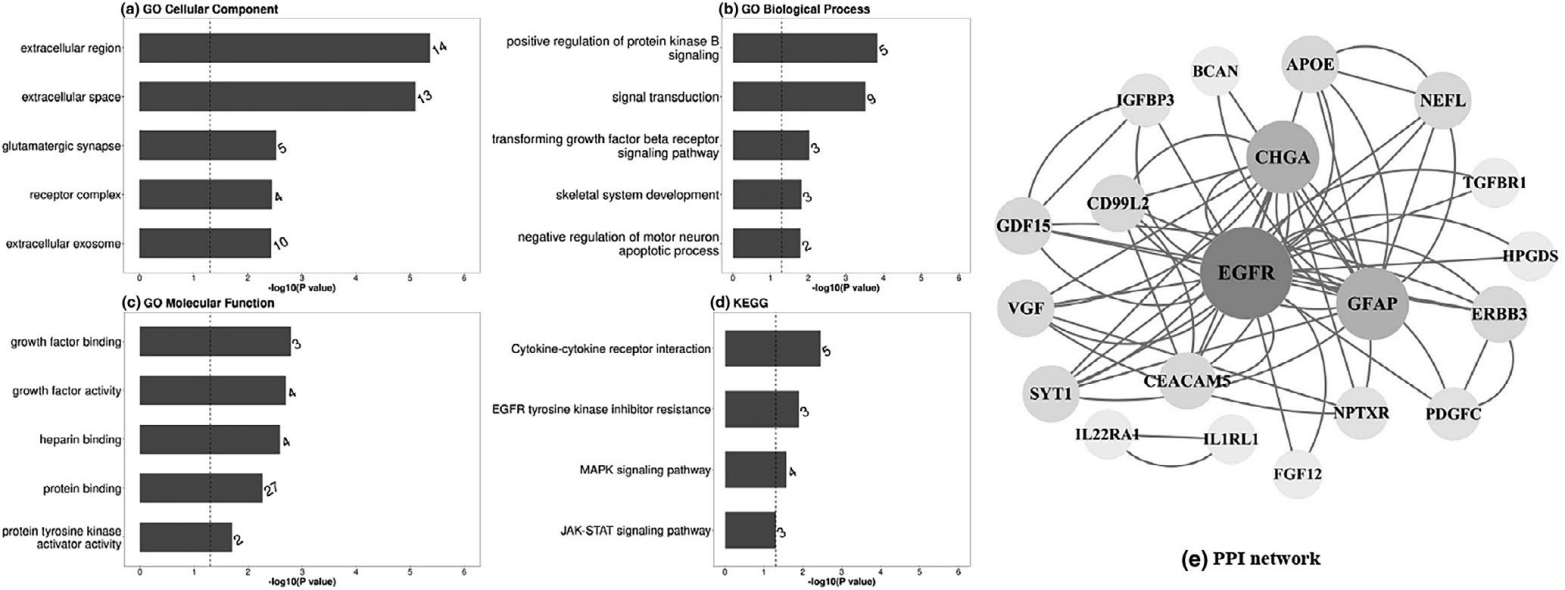
The Gene Ontology (GO) function enrichment analyses, including (a) cellular component, (b) biological process and (c) molecular function, (d) Kyoto Encyclopedia of Genes and Genomes (KEGG) pathway enrichment analyses, and (e) protein–protein interaction (PPI) network of proteins derived from the AD protein risk score. The number beside each bar represents the number of observed proteins in each pathway. Statistical significance was defined as a two‐tailed *p* value<0.05 (dotted vertical line). Network nodes represent proteins and edges represent protein–protein associations. The larger‐sized node represents core proteins.

The enrichment analyses demonstrated that 31 plasma proteins derived from the AD protein risk score were significantly enriched in GO cellular components (number of involved proteins) such as extracellular region (*n* = 14) and extracellular space (*n* = 13) (Figure 3a, Table S5). Proteins were also enriched in GO biological processes related to positive regulation of protein kinase B signaling (*n* = 5), GO molecular function related to growth factor binding (*n* = 3), and KEGG related to cytokine‐cytokine receptor interaction (*n* = 5) (Figure 3b–d, Table S5). In addition, after FDR correction within each protein panel, 10 proteins from the cardiometabolic panel, 8 from the inflammation panel, 5 from the neurology panel, and 5 from the oncology panel showed a significant association with the risk of AD (Table S6–S9).

Moreover, we further investigated the complex interactions between proteins through PPI analysis. As shown in Figure 3e, the identified proteins coalesced into distinct, interconnected protein clusters. Within the PPI network, EGFR, GFAP, and Chromogranin‐A (CHGA) emerged as the top three proteins exhibiting elevated values of betweenness centrality, closeness centrality, and degree centrality (Figure 3e, Table S10).

## 4 DISCUSSION

Using large‐scale protein profiling data from a population‐based cohort study, we developed and validated a protein risk score of 31 proteins for predicting AD risk. The AD protein risk score alone constitutes a straightforward yet effective model for predicting AD risk with a C‐index of 0.867 in the testing set and 0.912 in the validation cohort. Moreover, our results suggest that EGFR, GFAP, and CHGA may be key proteins in the mechanistic pathways associated with new‐onset AD.

To date, few studies have investigated whether a protein score developed based on major proteins associated with AD risk may contribute to the risk stratification of AD. Previous studies have shown that candidate protein combinations have a certain discriminative ability for risk stratification of all‐cause dementia risk (C‐index = 0.616–0.830). (Walker et al., 2021, 2023; You et al., 2023) Given that all‐cause dementia is a composite outcome, different subtypes of dementia, including AD, vascular dementia, frontotemporal dementia, etc., may have different characteristic proteins. Therefore, an AD‐specific protein score is needed to improve the risk stratification for future AD. A previous study, using protein data from the initial round of protein measurements in the UKB‐PPP, reported that a 7‐protein panel out of 1463 plasma proteins achieved an unvalidated area under curve (AUC) of 0.854 for predicting AD risk (Guo et al., 2024). However, after the latest round of protein measurements in the UKB‐PPP, a total of 2923 unique proteins were quantified. Therefore, it is necessary to use these broader plasma protein data to more fully explore the candidate proteins involved in predicting incident AD events. In addition, the prediction performance of the AD protein risk score compared to validated clinical prediction models (e.g., CogDrisk‐AD risk factors) and genetic factors (e.g., APOE genotype) remains unclear. Our study utilized 2923 proteins and identified 31 proteins that were associated with the risk of AD. Of the 31 identified proteins, 12 were from the latest protein measurements in the UKB‐PPP, such as MENT, VGF, APOE, and TGFBR1, etc., and 6 proteins (including GFAP, NEFL, LTBP2, NPTXR, growth/differentiation factor 15, and brevican core protein) overlapped with the 7 proteins identified in the previous study (Guo et al., 2024). Moreover, the AD protein risk score alone had a good discriminative performance in predicting AD (a C‐index of 0.867 in the testing set and 0.912 in the external validation cohort), followed by CogDrisk‐AD risk factors and APOE genotypes. These results were consistent across the training set, the testing set and the external validation cohort.

The current study provides some novel insights. First, the AD protein risk score showed good predictive accuracy for AD risk, and adding the AD protein risk score to the model including CogDrisk‐AD risk factors or APOE genotypes can significantly improve the discrimination and reclassification of AD risk. In terms of coverage, the AD protein risk score can capture a wider range of pathophysiological processes compared to the APOE genotypes. From the central dogma of molecular biology, proteins are downstream products of gene expression, located closer to the disease phenotype (Babu & Snyder, 2023). Therefore, the AD protein risk score can complement traditional risk factors for AD and APOE genotypes to help clinicians better identify people at high risk for AD and enhance their early surveillance and prevention.

Second, the top 10 proteins with the highest coefficients in the AD protein risk score contributed the most to the ability of the AD protein risk score to predict AD risk, with a C‐index of 0.849 in the testing set and 0.891 in the external validation cohort. First, the current results reinforce previous findings that GFAP and NEFL may have the potential not only to predict the overall risk of developing dementia (Walker et al., 2021, 2023; You et al., 2023) but also to serve as specific biomarkers to assess AD risk. Moreover, consistent with prior studies in humans or animals, LTBP2 (Guo et al., 2024), EGFR (Jayaswamy et al., 2023), APOE (Aslam et al., 2023), TGFBR1 (Kuroda et al., 2020), VGF (Beckmann et al., 2020), NPTXR (Wildsmith et al., 2014), and IGFBP3 (Duron et al., 2012) were associated with the risk of AD. Second, our study expands existing knowledge by identifying elevated plasma MENT levels as a potential novel biomarker for AD risk. Previous studies have shown that MENT has a potential role in cell differentiation and maturation (Alkebsi et al., 2013; Hlady et al., 2012), while its mechanisms in the development of AD remain unclear. Furthermore, PPI analysis indicates that EGFR, GFAP, and CHGA may have a central role in maintaining connectivity and facilitating communication within the network of AD pathogenesis. Enrichment analysis suggests that candidate proteins were enriched in GO biological processes related to positive regulation of protein kinase B signaling, GO molecular function related to growth factor binding, and KEGG related to cytokine‐cytokine receptor interaction, which was partially consistent with previous findings (Beydoun et al., 2024). However, more future studies are needed to further confirm their potential pivotal role in the mechanism of new‐onset AD.

Our study has some limitations. First, the characteristics of the UKB population limit the generalizability of the findings to other populations. Second, although the diagnosis of AD in the current study was based on multiple medical sources and validated in previous studies (Wilkinson et al., 2019), the use of diagnostic and procedural codes to define AD may have underestimated the true number of AD cases. Given the potentially longer latency period of AD and its complexity across different stages, future studies incorporating positron emission tomography imaging and cerebral spinal fluid analysis will be critical to accurately define AD cases. Third, although the Olink platform offers a comprehensive assessment of circulating proteins, it does not cover the full human proteome, with no detection of proteins such as amyloid beta‐40, amyloid beta‐42, phosphorylated tau, and neurofilament light.

## 5 CONCLUSION

In summary, we developed and validated an AD protein risk score that had a better predictive performance for AD risk. Adding the AD protein risk score to the model including CogDrisk‐AD risk factors significantly improved determination and reclassification of new‐onset AD risk. Blood is easily collected in a clinical setting. Blood biomarker detection is objective, quantifiable and convenient, while the collection of clinical risk factors is relatively cumbersome, requiring reports or medical records from participants and multiple tests. If further confirmed, our findings provide a simple method for predicting AD, especially in large populations, that is, by simply obtaining blood samples for protein detection without the need for face‐to‐face clinical assessment to identify people at high risk for AD for intensive management and prevention.

## AUTHOR CONTRIBUTIONS

Sisi Yang, Panpan He, Ziliang Ye, and Xianhui Qin conceived and designed the study. Sisi Yang, Panpan He, Ziliang Ye, and Xianhui Qin conducted the study. Sisi Yang, Panpan He, and Ziliang Ye contributed to statistical analysis. Sisi Yang, Ziliang Ye, and Xianhui Qin drafted the manuscript. All authors reviewed/edited the manuscript important intellectual content. All authors read and approver the final manuscript.

## FUNDING INFORMATION

This study was supported by the National Key Research and Development Program of China (2021YFC2500200, 2022YFC2009600 and 2022YFC2009605), National Natural Science Foundation of China (81973133, 82030022, 82330020), Key Technologies R&D Program of Guangdong Province (2023B1111030004), Guangdong Provincial Clinical Research Center for Kidney Disease (2020B1111170013) and the Program of Introducing Talents of Discipline to Universities, 111 Plan (D18005).

## CONFLICT OF INTEREST STATEMENT

The authors declare no conflicts of interest.

## Supporting information


Data S1.

## Data Availability

The data underlying this article are available in UK Biobank at https://www.ukbiobank.ac.uk/, and can be accessed with reasonable request.
