# Supplementary material for: Plasma proteomics for risk prediction of Alzheimer's disease in the general population
Source: Aging Cell. 2024 Sep 9;23(12):e14330. doi: 10.1111/acel.14330 (PMC11634738; doi:10.1111/acel.14330)
Supplement: Supplementary file 1 — Data S1. [file ACEL-23-e14330-s001.docx]

53,029 participants with available proteomics data at baseline based on the October 2023 release of the UK Biobank

Excluded n=12,815

Prevalent dementia at baseline: n=51

Missing APOE gene data or sex discrepancies between the self-reported and X-chromosome heterozygosity: n=1,871

Missing baseline covariate (age, sex, education, obesity, diabetes, depression, high cholesterol, traumatic brain injury, smoking, loneliness, physical activity, cognitive activity, fish intake, hypertension, stroke and occupational pesticides exposure) data: n= 10,893

40,214 participants were finally included in this study

**Development cohort** from UKB England area, N=35,547

**Validation cohort** from UKB Scotland and Wales area, N=4,667

Training set (70%)

N=24,882

Testing set (30%)

N=10,665

**Figure S1. Flow chart of the participants in the current analysis.**


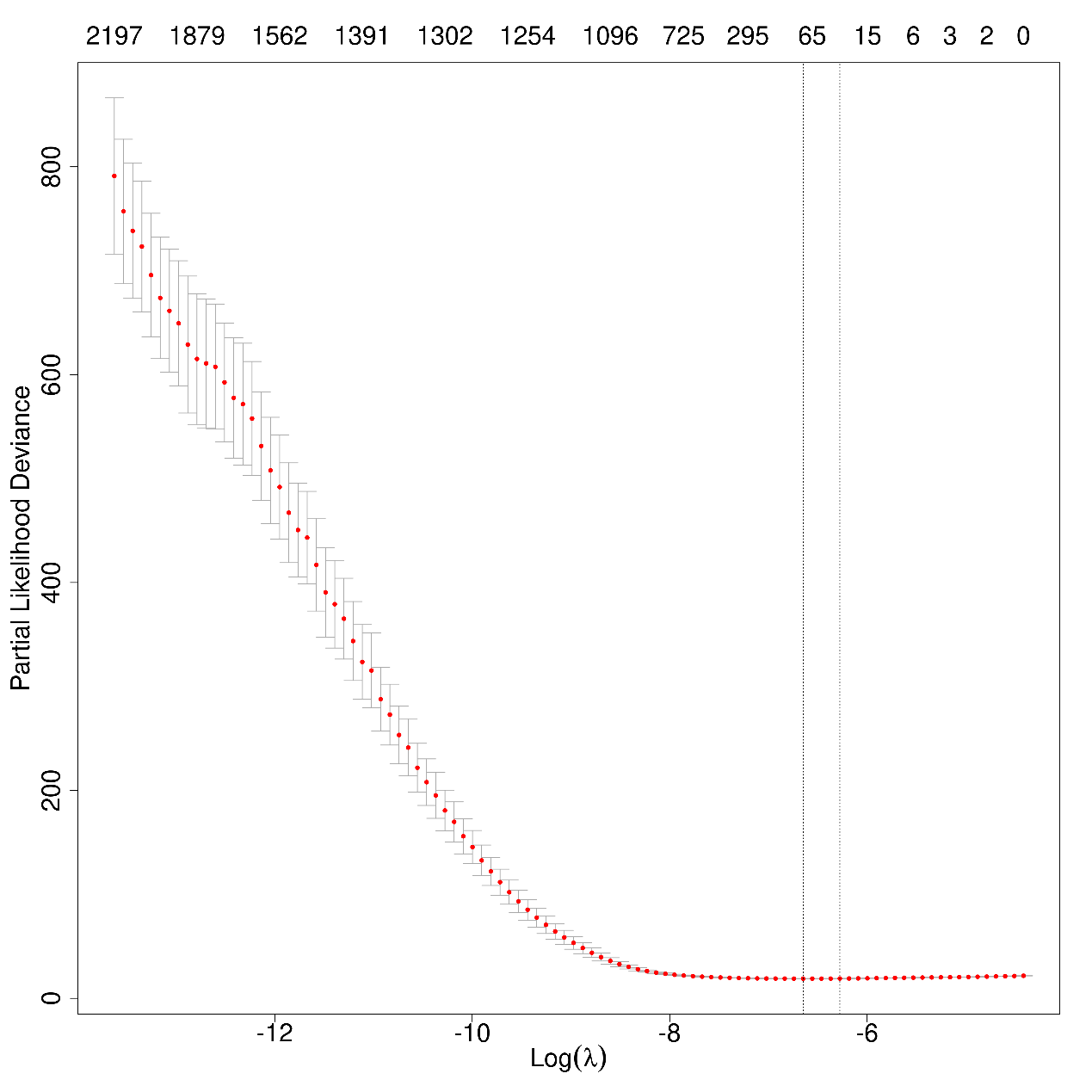


**Figure S2. Cross-Validation for Selecting Penalization Strength for Fitting Protein Alzheimer’s disease Risk Prediction Models.**

The figure demonstrates how the penalization strength λ for fitting the protein risk score using all 2911 proteins, age and sex is selected based on the partial likelihood of deviance (PLD) in ten-fold cross-validation. The red dots represent the mean PLD for each λ that was used and the bars represent one standard deviation in each direction for the cross-validations. The top x-axis shows how the number of nonzero coefficients changes with the penalization strength.


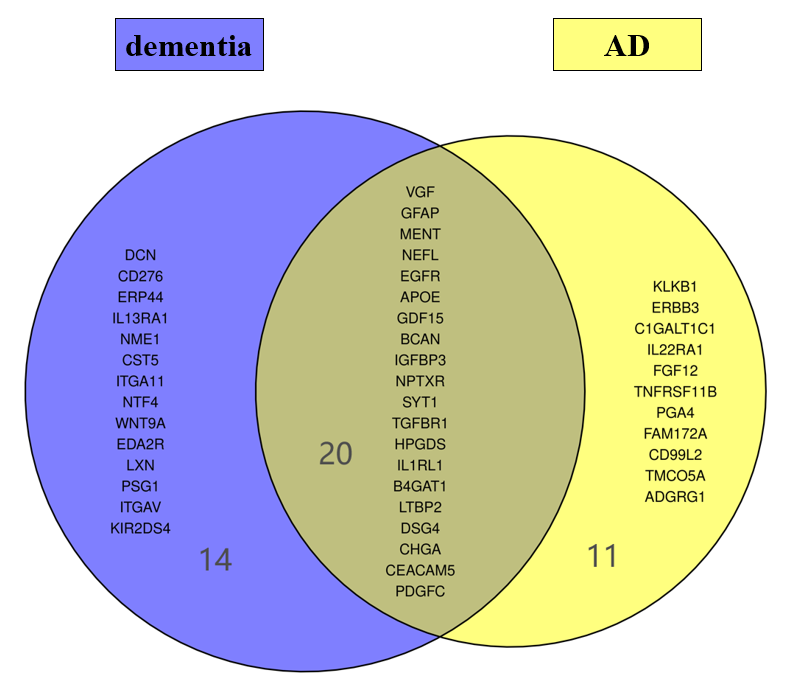


**Figure S3. Venn diagram showing the number of proteins selected by the LASSO model for dementia and Alzheimer’s disease (AD).**

**Table S1. 31 proteins selected by the LASSO model to create an Alzheimer’s disease protein risk score.**

| **Target Name** | **Target Full Name** | **UniProt** | **Panel^*^** | **Coefficient/**  **weight** |
| --- | --- | --- | --- | --- |
| GFAP | Glial fibrillary acidic protein | P14136 | Oncology | 1.084 |
| MENT | Protein methylated in normal thymocytes protein | Q9BUN1 | Inflammation II | 0.912 |
| VGF | Neurosecretory protein VGF | O15240 | Neurology II | -0.576 |
| NEFL | Neurofilament light polypeptide | P07196 | Neurology | 0.545 |
| EGFR | Epidermal growth factor receptor | P00533 | Cardiometabolic | -0.508 |
| NPTXR | Neuronal pentraxin receptor | O95502 | Cardiometabolic | -0.500 |
| APOE | Apolipoprotein E | P02649 | Inflammation II | -0.466 |
| TGFBR1 | TGF-beta receptor type-1 | P36897 | Inflammation II | 0.318 |
| LTBP2 | Latent-transforming growth factor beta-binding protein 2 | Q14767 | Cardiometabolic | 0.266 |
| IGFBP3 | Insulin-like growth factor-binding protein 3 | P17936 | Cardiometabolic | -0.259 |
| SYT1 | Synaptotagmin-1 | P21579 | Neurology II | 0.215 |
| BCAN | Brevican core protein | Q96GW7 | Neurology | -0.212 |
| PDGFC | Platelet-derived growth factor C | Q9NRA1 | Oncology | -0.151 |
| GDF15 | Growth/differentiation factor 15 | Q99988 | Cardiometabolic | 0.146 |
| KLKB1 | Plasma kallikrein | P03952 | Inflammation II | -0.092 |
| ERBB3 | Receptor tyrosine-protein kinase erbB-3 | P21860 | Inflammation | -0.085 |
| IL1RL1 | Interleukin-1 receptor-like 1 | Q01638 | Cardiometabolic | 0.080 |
| DSG4 | Desmoglein-4 | Q86SJ6 | Oncology | -0.064 |
| C1GALT1C1 | C1GALT1-specific chaperone 1 | Q96EU7 | Cardiometabolic II | -0.062 |
| CEACAM5 | Carcinoembryonic antigen-related cell adhesion molecule 5 | P06731 | Oncology | 0.047 |
| HPGDS | Hematopoietic prostaglandin D synthase | O60760 | Oncology | -0.046 |
| IL22RA1 | Interleukin-22 receptor subunit alpha-1 | Q8N6P7 | Inflammation | -0.042 |
| FGF12 | Fibroblast growth factor 12 | P61328 | Inflammation II | 0.028 |
| TNFRSF11B | Tumor necrosis factor receptor superfamily member 11B | O00300 | Inflammation | 0.026 |
| PGA4 | Pepsin A-4 | P0DJD7 | Inflammation II | 0.025 |
| CHGA | Chromogranin-A | P10645 | Cardiometabolic II | 0.023 |
| FAM172A | Cotranscriptional regulator FAM172A | Q8WUF8 | Cardiometabolic II | -0.020 |
| CD99L2 | CD99 antigen-like protein 2 | Q8TCZ2 | Neurology | -0.009 |
| TMCO5A | Transmembrane and coiled-coil domain-containing protein 5A | Q8N6Q1 | Neurology II | -0.006 |
| ADGRG1 | Adhesion G-protein coupled receptor G1 | Q9Y653 | Oncology | 0.001 |
| B4GAT1 | Beta-1,4-glucuronyltransferase 1 | O43505 | Neurology | -0.001 |

**^*^** In the UK Biobank Pharma Proteomics Project (UKB-PPP), in the initial round of measurements, 1463 unique proteins across Olink’s panels (cardiometabolic, inflammation, neurology, and oncology) in UKB-PPP plasma sample collected at baseline was assessed. In the latest round of measurements, 1460 unique proteins across Olink’s panels (cardiometabolic II, inflammation II, neurology II, and oncology II) were evaluated.

**Table S2. Cox regression analysis for the associations between 31 proteins and the risk of new-onset Alzheimer’s disease in the training set. ^*^**

| **Target Name** | **UniProt** | **Panel** | **HR (95%CI)** | ***P* value** | **FDR-corrected *P* value** |
| --- | --- | --- | --- | --- | --- |
| GFAP | P14136 | Oncology | 3.42(2.90,4.04) | 2.92E-47 | <0.001 |
| MENT | Q9BUN1 | Inflammation II | 8.04(4.76,13.60) | 7.37E-15 | <0.001 |
| VGF | O15240 | Neurology II | 0.45(0.29,0.69) | 2.45E-04 | <0.001 |
| NEFL | P07196 | Neurology | 2.35(1.96,2.82) | 4.27E-20 | <0.001 |
| EGFR | P00533 | Cardiometabolic | 0.29(0.15,0.56) | 2.21E-04 | <0.001 |
| NPTXR | O95502 | Cardiometabolic | 0.48(0.33,0.68) | 5.18E-05 | <0.001 |
| APOE | P02649 | Inflammation II | 0.29(0.23,0.37) | 1.02E-22 | <0.001 |
| TGFBR1 | P36897 | Inflammation II | 2.00(1.45,2.76) | 2.74E-05 | <0.001 |
| LTBP2 | Q14767 | Cardiometabolic | 2.20(1.59,3.05) | 2.11E-06 | <0.001 |
| IGFBP3 | P17936 | Cardiometabolic | 0.51(0.37,0.71) | 7.12E-05 | <0.001 |
| SYT1 | P21579 | Neurology II | 1.53(1.28,1.82) | 2.04E-06 | <0.001 |
| BCAN | Q96GW7 | Neurology | 0.62(0.46,0.86) | 3.50E-03 | 0.004 |
| PDGFC | Q9NRA1 | Oncology | 0.45(0.30,0.68) | 1.30E-04 | <0.001 |
| GDF15 | Q99988 | Cardiometabolic | 1.65(1.36,2.01) | 6.07E-07 | <0.001 |
| KLKB1 | P03952 | Inflammation II | 0.34(0.20,0.56) | 3.01E-05 | <0.001 |
| ERBB3 | P21860 | Inflammation | 0.32(0.18,0.59) | 2.10E-04 | <0.001 |
| IL1RL1 | Q01638 | Cardiometabolic | 1.77(1.37,2.28) | 9.80E-06 | <0.001 |
| DSG4 | Q86SJ6 | Oncology | 0.72(0.58,0.91) | 4.55E-03 | 0.005 |
| C1GALT1C1 | Q96EU7 | Cardiometabolic II | 0.49(0.31,0.78) | 2.58E-03 | 0.003 |
| CEACAM5 | P06731 | Oncology | 1.37(1.16,1.61) | 1.54E-04 | <0.001 |
| HPGDS | O60760 | Oncology | 0.58(0.42,0.79) | 5.83E-04 | 0.001 |
| IL22RA1 | Q8N6P7 | Inflammation | 0.62(0.45,0.85) | 3.08E-03 | 0.004 |
| FGF12 | P61328 | Inflammation II | 1.30(1.10,1.53) | 1.86E-03 | 0.003 |
| TNFRSF11B | O00300 | Inflammation | 1.96(1.35,2.85) | 3.98E-04 | 0.001 |
| PGA4 | P0DJD7 | Inflammation II | 1.34(1.13,1.58) | 7.74E-04 | 0.001 |
| CHGA | P10645 | Cardiometabolic II | 1.25(1.11,1.40) | 1.83E-04 | <0.001 |
| FAM172A | Q8WUF8 | Cardiometabolic II | 0.73(0.58,0.92) | 6.56E-03 | 0.007 |
| CD99L2 | Q8TCZ2 | Neurology | 0.61(0.39,0.97) | 3.87E-02 | 0.039 |
| TMCO5A | Q8N6Q1 | Neurology II | 0.66(0.50,0.88) | 4.30E-03 | 0.005 |
| ADGRG1 | Q9Y653 | Oncology | 1.14(1.02,1.29) | 2.72E-02 | 0.028 |
| B4GAT1 | O43505 | Neurology | 0.53(0.33,0.84) | 6.90E-03 | 0.007 |

**^*^**Cox regression model adjusted for age and sex. *P* values were calculated under two-sided tests, and statistical significance was defined as a false discovery rate (FDR)-corrected *P* value <0.05 with adjustments for the number of proteins tested (n=31).

**Table S3. The cumulative C-index of 31 proteins for predicting Alzheimer’s disease (AD) risk.**

| **Target Name** | **UniProt** | **Coefficient /weight** | **^*^Cumulative C-index (95%CI) in validation cohort** | **^*^Cumulative C-index (95%CI) in testing set** |
| --- | --- | --- | --- | --- |
| GFAP | P14136 | 1.084 | 0.753(0.658,0.849) | 0.785(0.736,0.834) |
| MENT | Q9BUN1 | 0.912 | 0.767(0.677,0.856) | 0.779(0.727,0.830) |
| VGF | O15240 | -0.576 | 0.792(0.711,0.873) | 0.789(0.738,0.840) |
| NEFL | P07196 | 0.545 | 0.830(0.762,0.898) | 0.829(0.785,0.872) |
| EGFR | P00533 | -0.508 | 0.841(0.773,0.909) | 0.834(0.791,0.877) |
| NPTXR | O95502 | -0.500 | 0.856(0.799,0.913) | 0.842(0.801,0.883) |
| APOE | P02649 | -0.466 | 0.873(0.811,0.935) | 0.839(0.794,0.883) |
| TGFBR1 | P36897 | 0.318 | 0.875(0.814,0.937) | 0.840(0.796,0.884) |
| LTBP2 | Q14767 | 0.266 | 0.888(0.834,0.942) | 0.848(0.806,0.890) |
| IGFBP3 | P17936 | -0.259 | 0.891(0.839,0.943) | 0.849(0.807,0.891) |
| SYT1 | P21579 | 0.215 | 0.892(0.840,0.944) | 0.850(0.808,0.892) |
| BCAN | Q96GW7 | -0.212 | 0.900(0.853,0.947) | 0.855(0.814,0.896) |
| PDGFC | Q9NRA1 | -0.151 | 0.904(0.859,0.949) | 0.855(0.814,0.897) |
| GDF15 | Q99988 | 0.146 | 0.909(0.866,0.951) | 0.863(0.824,0.903) |
| KLKB1 | P03952 | -0.092 | 0.908(0.865,0.952) | 0.863(0.823,0.902) |
| ERBB3 | P21860 | -0.085 | 0.909(0.865,0.952) | 0.862(0.823,0.902) |
| IL1RL1 | Q01638 | 0.080 | 0.907(0.864,0.951) | 0.864(0.825,0.904) |
| DSG4 | Q86SJ6 | -0.064 | 0.909(0.866,0.952) | 0.864(0.824,0.903) |
| C1GALT1C1 | Q96EU7 | -0.062 | 0.909(0.867,0.952) | 0.864(0.825,0.904) |
| CEACAM5 | P06731 | 0.047 | 0.910(0.867,0.953) | 0.865(0.826,0.904) |
| HPGDS | O60760 | -0.046 | 0.911(0.868,0.953) | 0.866(0.827,0.905) |
| IL22RA1 | Q8N6P7 | -0.042 | 0.910(0.867,0.953) | 0.866(0.827,0.905) |
| FGF12 | P61328 | 0.028 | 0.910(0.867,0.953) | 0.865(0.826,0.904) |
| TNFRSF11B | O00300 | 0.026 | 0.910(0.867,0.953) | 0.865(0.826,0.904) |
| PGA4 | P0DJD7 | 0.025 | 0.909(0.867,0.952) | 0.866(0.827,0.905) |
| CHGA | P10645 | 0.023 | 0.910(0.868,0.953) | 0.867(0.828,0.906) |
| FAM172A | Q8WUF8 | -0.020 | 0.911(0.869,0.953) | 0.867(0.828,0.906) |
| CD99L2 | Q8TCZ2 | -0.009 | 0.911(0.869,0.953) | 0.867(0.828,0.906) |
| TMCO5A | Q8N6Q1 | -0.006 | 0.912(0.870,0.953) | 0.867(0.828,0.906) |
| ADGRG1 | Q9Y653 | 0.001 | 0.912(0.870,0.953) | 0.867(0.828,0.906) |
| B4GAT1 | O43505 | -0.001 | 0.912(0.870,0.953) | 0.867(0.828,0.906) |

* The cumulative C-index for predicting new-onset AD risk was calculated when proteins in the AD protein risk score were included sequentially (in order of coefficient from the largest to smallest).

**Table S4. Classification table of 10-year incident Alzheimer’s disease (AD) risk and 10-year risk categorical net reclassification improvement (NRI) in the testing set (10,665 participants; 68 events within 10 years) and in the validation cohort (4,667 participants; 19 events within 10 years).^*^**

^*^ p = 1.0% (392/40214), is the incident rate of AD in the whole cohort.

**Table S5. Detailed information on the Gene ontology (GO) function enrichment analyses and the Kyoto Encyclopedia of Genes and Genomes (KEGG) pathway enrichment analyses.**

| **Enrichment** | ***P* value** | **^*^Count** | **Proteins** |
| --- | --- | --- | --- |
| **GO cellular component** |  |  |  |
| extracellular region | <0.001 | 14 | KLKB1, CHGA, MENT, ADGRG1, LTBP2, BCAN, TNFRSF11B, APOE, IGFBP3, CD99L2, PDGFC, IL1RL1, GDF15, CEACAM5 |
| extracellular space | <0.001 | 13 | VGF, KLKB1, CHGA, LTBP2, BCAN, TNFRSF11B, APOE, IGFBP3, ERBB3, EGFR, PDGFC, FGF12, GDF15 |
| glutamatergic synapse | 0.003 | 5 | VGF, SYT1, NPTXR, APOE, BCAN |
| receptor complex | 0.004 | 4 | ERBB3, TGFBR1, EGFR, TNFRSF11B |
| extracellular exosome | 0.004 | 10 | PGA4, KLKB1, ADGRG1, APOE, LTBP2, PDGFC, C1GALT1C1, CEACAM5, GDF15, B4GAT1 |
| clathrin-coated endocytic vesicle membrane | 0.005 | 3 | SYT1, APOE, EGFR |
| endoplasmic reticulum lumen | 0.010 | 4 | VGF, APOE, IGFBP3, PDGFC |
| plasma membrane region | 0.013 | 2 | ERBB3, EGFR |
| cell surface | 0.014 | 5 | TGFBR1, CD99L2, EGFR, PDGFC, CEACAM5 |
| plasma membrane | 0.032 | 14 | KLKB1, ADGRG1, IL22RA1, TGFBR1, TNFRSF11B, SYT1, APOE, DSG4, ERBB3, CD99L2, EGFR, PDGFC, IL1RL1, CEACAM5 |
| membrane raft | 0.037 | 3 | ADGRG1, TGFBR1, EGFR |
| extracellular matrix | 0.040 | 3 | APOE, LTBP2, TNFRSF11B |
| basolateral plasma membrane | 0.056 | 3 | ERBB3, EGFR, CEACAM5 |
| Golgi apparatus | 0.073 | 5 | VGF, SYT1, APOE, GDF15, B4GAT1 |
| Golgi membrane | 0.078 | 4 | EGFR, PDGFC, C1GALT1C1, B4GAT1 |
| membrane | 0.090 | 13 | ADGRG1, NPTXR, IL22RA1, TGFBR1, C1GALT1C1, SYT1, APOE, ERBB3, CD99L2, EGFR, PDGFC, TMCO5A, CEACAM5 |
| basal plasma membrane | 0.097 | 2 | ERBB3, EGFR |
| **GO biological process** |  |  |  |
| positive regulation of protein kinase B signaling | <0.001 | 5 | ERBB3, TGFBR1, EGFR, PDGFC, GDF15 |
| signal transduction | <0.001 | 9 | VGF, HPGDS, ERBB3, TGFBR1, EGFR, FGF12, IL1RL1, TNFRSF11B, GDF15 |
| transforming growth factor beta receptor signaling pathway | 0.009 | 3 | LTBP2, TGFBR1, GDF15 |
| skeletal system development | 0.015 | 3 | TGFBR1, BCAN, TNFRSF11B |
| negative regulation of motor neuron apoptotic process | 0.016 | 2 | NEFL, ERBB3 |
| regulation of cell proliferation | 0.019 | 3 | MENT, ERBB3, EGFR |
| motor neuron apoptotic process | 0.022 | 2 | NEFL, ERBB3 |
| response to dietary excess | 0.024 | 2 | VGF, APOE |
| regulation of protein complex assembly | 0.032 | 2 | APOE, GFAP |
| regulation of neuronal synaptic plasticity | 0.033 | 2 | VGF, APOE |
| regulation of peptidyl-tyrosine phosphorylation | 0.033 | 2 | EGFR, PDGFC |
| artery morphogenesis | 0.036 | 2 | APOE, TGFBR1 |
| positive regulation of cell proliferation | 0.036 | 4 | ERBB3, TGFBR1, EGFR, PDGFC |
| negative regulation of apoptotic process | 0.036 | 4 | ERBB3, TGFBR1, EGFR, CEACAM5 |
| heart development | 0.037 | 3 | ERBB3, TGFBR1, FGF12 |
| positive regulation of ERK1 and ERK2 cascade | 0.042 | 3 | APOE, EGFR, PDGFC |
| negative regulation of smooth muscle cell proliferation | 0.043 | 2 | APOE, IGFBP3 |
| cell-cell signaling | 0.044 | 3 | ADGRG1, FGF12, GDF15 |
| positive regulation of cell migration | 0.053 | 3 | TGFBR1, EGFR, PDGFC |
| apoptotic process | 0.054 | 4 | IGFBP3, TGFBR1, TNFRSF11B, CEACAM5 |
| negative regulation of endothelial cell proliferation | 0.055 | 2 | APOE, TGFBR1 |
| hair follicle development | 0.061 | 2 | DSG4, EGFR |
| vasodilation | 0.062 | 2 | APOE, EGFR |
| negative regulation of signal transduction | 0.070 | 2 | IGFBP3, ERBB3 |
| positive regulation of fibroblast proliferation | 0.076 | 2 | EGFR, PDGFC |
| positive regulation of kinase activity | 0.077 | 2 | ERBB3, EGFR |
| positive regulation of MAP kinase activity | 0.078 | 2 | EGFR, PDGFC |
| cell surface receptor signaling pathway | 0.080 | 3 | ADGRG1, ERBB3, EGFR |
| neurogenesis | 0.084 | 2 | ERBB3, EGFR |
| cellular response to amino acid stimulus | 0.088 | 2 | EGFR, PDGFC |
| receptor-mediated endocytosis | 0.096 | 2 | APOE, EGFR |
| intermediate filament organization | 0.096 | 2 | NEFL, GFAP |
| negative regulation of neuron projection development | 0.098 | 2 | APOE, GFAP |
| **GO molecular function** |  |  |  |
| growth factor binding | 0.002 | 3 | LTBP2, ERBB3, TGFBR1 |
| growth factor activity | 0.002 | 4 | VGF, PDGFC, FGF12, GDF15 |
| heparin binding | 0.003 | 4 | ADGRG1, APOE, LTBP2, FGF12 |
| protein binding | 0.005 | 27 | MENT, LTBP2, TNFRSF11B, GFAP, PGA4, NEFL, APOE, HPGDS, ERBB3, PDGFC, FGF12, TMCO5A, GDF15, FAM172A, KLKB1, ADGRG1, IL22RA1, TGFBR1, C1GALT1C1, BCAN, B4GAT1, SYT1, IGFBP3, CD99L2, EGFR, IL1RL1, CEACAM5 |
| protein tyrosine kinase activator activity | 0.020 | 2 | ERBB3, EGFR |
| protein homodimerization activity | 0.026 | 5 | HPGDS, APOE, PDGFC, CEACAM5, GDF15 |
| low-density lipoprotein particle receptor binding | 0.036 | 2 | SYT1, APOE |
| identical protein binding | 0.045 | 7 | SYT1, NEFL, APOE, ERBB3, EGFR, CEACAM5, GFAP |
| transmembrane receptor protein tyrosine kinase activity | 0.063 | 2 | ERBB3, EGFR |
| cytokine receptor activity | 0.071 | 2 | IL22RA1, IL1RL1 |
| ubiquitin protein ligase binding | 0.083 | 3 | ERBB3, TGFBR1, EGFR |
| **KEGG** |  |  |  |
| Cytokine-cytokine receptor interaction | 0.003 | 5 | IL22RA1, TGFBR1, IL1RL1, TNFRSF11B, GDF15 |
| EGFR tyrosine kinase inhibitor resistance | 0.013 | 3 | ERBB3, EGFR, PDGFC |
| MAPK signaling pathway | 0.027 | 4 | ERBB3, TGFBR1, EGFR, PDGFC |
| JAK-STAT signaling pathway | 0.050 | 3 | IL22RA1, EGFR, GFAP |

^*^Count: the number of observed proteins in each pathway.

**Table S6. Cox regression analysis for the associations between 731 proteins from the cardiometabolic panel and the risk of new-onset Alzheimer’s disease in the training set. ^*^**

| **Target Name** | **UniProt** | **Panel** | **HR (95%CI)** | ***P* value** | **FDR-corrected *P* value** |
| --- | --- | --- | --- | --- | --- |
| GDF15 | Q99988 | Cardiometabolic | 1.65(1.36,2.01) | 6.07E-07 | <0.001 |
| LTBP2 | Q14767 | Cardiometabolic | 2.20(1.59,3.05) | 2.11E-06 | 0.001 |
| LDLR | P01130 | Cardiometabolic | 0.60(0.48,0.75) | 4.36E-06 | 0.001 |
| IL1RL1 | Q01638 | Cardiometabolic | 1.77(1.37,2.28) | 9.80E-06 | 0.002 |
| NPTXR | O95502 | Cardiometabolic | 0.48(0.33,0.68) | 5.18E-05 | 0.008 |
| IGFBP3 | P17936 | Cardiometabolic | 0.51(0.37,0.71) | 7.12E-05 | 0.009 |
| CHGA | P10645 | Cardiometabolic II | 1.25(1.11,1.40) | 1.83E-04 | 0.019 |
| EGFR | P00533 | Cardiometabolic | 0.29(0.15,0.56) | 2.21E-04 | 0.020 |
| SPP1 | P10451 | Cardiometabolic | 1.57(1.23,2.00) | 3.11E-04 | 0.025 |
| DCN | P07585 | Cardiometabolic | 2.19(1.41,3.41) | 4.96E-04 | 0.036 |
| MAN2B2 | Q9Y2E5 | Cardiometabolic II | 0.71(0.58,0.88) | 1.97E-03 | 0.124 |
| CES1 | P23141 | Cardiometabolic | 0.80(0.69,0.92) | 2.04E-03 | 0.124 |
| DPP4 | P27487 | Cardiometabolic | 0.54(0.36,0.80) | 2.25E-03 | 0.125 |
| REN | P00797 | Cardiometabolic | 1.21(1.07,1.37) | 2.39E-03 | 0.125 |
| C1GALT1C1 | Q96EU7 | Cardiometabolic II | 0.49(0.31,0.78) | 2.58E-03 | 0.126 |
| TFF3 | Q07654 | Cardiometabolic | 1.40(1.12,1.76) | 2.88E-03 | 0.128 |
| ACTA2 | P62736 | Cardiometabolic | 1.39(1.12,1.72) | 2.98E-03 | 0.128 |
| TINAGL1 | Q9GZM7 | Cardiometabolic | 2.13(1.28,3.55) | 3.80E-03 | 0.135 |
| REG3A | Q06141 | Cardiometabolic | 1.27(1.08,1.49) | 3.83E-03 | 0.135 |
| CLEC5A | Q9NY25 | Cardiometabolic | 1.71(1.19,2.46) | 3.90E-03 | 0.135 |
| CD59 | P13987 | Cardiometabolic | 1.92(1.23,2.99) | 3.97E-03 | 0.135 |
| NOTCH3 | Q9UM47 | Cardiometabolic | 1.61(1.16,2.24) | 4.06E-03 | 0.135 |
| ERVV-1 | B6SEH8 | Cardiometabolic II | 0.60(0.42,0.86) | 5.45E-03 | 0.173 |
| ACY1 | Q03154 | Cardiometabolic | 0.76(0.63,0.92) | 5.92E-03 | 0.180 |
| FAM172A | Q8WUF8 | Cardiometabolic II | 0.73(0.58,0.92) | 6.56E-03 | 0.192 |
| PDGFRA | P16234 | Cardiometabolic | 1.68(1.15,2.46) | 6.92E-03 | 0.195 |
| CA3 | P07451 | Cardiometabolic | 1.27(1.06,1.51) | 7.61E-03 | 0.197 |
| NAA10 | P41227 | Cardiometabolic II | 0.70(0.54,0.91) | 7.62E-03 | 0.197 |
| MYL3 | P08590 | Cardiometabolic II | 1.27(1.06,1.51) | 8.06E-03 | 0.197 |
| SRPX | P78539 | Cardiometabolic II | 1.63(1.13,2.34) | 8.29E-03 | 0.197 |
| KHK | P50053 | Cardiometabolic II | 0.75(0.61,0.93) | 8.86E-03 | 0.197 |
| PI3 | P19957 | Cardiometabolic | 1.29(1.06,1.56) | 9.05E-03 | 0.197 |
| MYBPC2 | Q14324 | Cardiometabolic II | 1.19(1.04,1.35) | 9.63E-03 | 0.197 |
| CNDP1 | Q96KN2 | Cardiometabolic | 0.74(0.59,0.93) | 9.93E-03 | 0.197 |
| PON2 | Q15165 | Cardiometabolic | 0.65(0.47,0.90) | 9.98E-03 | 0.197 |
| ITGBL1 | O95965 | Cardiometabolic II | 1.52(1.11,2.10) | 1.01E-02 | 0.197 |
| MYBPC1 | Q00872 | Cardiometabolic II | 1.24(1.05,1.47) | 1.02E-02 | 0.197 |
| HMGCL | P35914 | Cardiometabolic II | 0.68(0.50,0.91) | 1.03E-02 | 0.197 |
| TSNAX | Q99598 | Cardiometabolic II | 0.63(0.44,0.90) | 1.05E-02 | 0.197 |
| PROC | P04070 | Cardiometabolic | 0.57(0.36,0.89) | 1.27E-02 | 0.230 |
| ACADSB | P45954 | Cardiometabolic II | 0.82(0.71,0.96) | 1.35E-02 | 0.230 |
| ASS1 | P00966 | Cardiometabolic II | 0.80(0.66,0.95) | 1.35E-02 | 0.230 |
| ECHS1 | P30084 | Cardiometabolic II | 0.84(0.72,0.96) | 1.35E-02 | 0.230 |
| IGFBP2 | P18065 | Cardiometabolic | 1.24(1.05,1.48) | 1.39E-02 | 0.231 |
| CHMP6 | Q96FZ7 | Cardiometabolic II | 0.75(0.59,0.94) | 1.47E-02 | 0.239 |
| ROR1 | Q01973 | Cardiometabolic | 1.53(1.08,2.17) | 1.60E-02 | 0.252 |
| EDN1 | P05305 | Cardiometabolic II | 1.70(1.10,2.61) | 1.62E-02 | 0.252 |
| COQ7 | Q99807 | Cardiometabolic II | 0.65(0.45,0.93) | 1.83E-02 | 0.276 |
| CHI3L1 | P36222 | Cardiometabolic | 1.17(1.03,1.34) | 1.89E-02 | 0.276 |
| COL6A3 | P12111 | Cardiometabolic | 1.41(1.06,1.87) | 1.89E-02 | 0.276 |
| COCH | O43405 | Cardiometabolic II | 0.71(0.53,0.95) | 1.98E-02 | 0.284 |
| NXPE4 | Q6UWF7 | Cardiometabolic II | 0.56(0.34,0.91) | 2.03E-02 | 0.285 |
| APOM | O95445 | Cardiometabolic | 0.64(0.43,0.94) | 2.19E-02 | 0.302 |
| TWF2 | Q6IBS0 | Cardiometabolic II | 0.83(0.71,0.97) | 2.26E-02 | 0.306 |
| F7 | P08709 | Cardiometabolic | 0.67(0.47,0.95) | 2.30E-02 | 0.306 |
| AHNAK | Q09666 | Cardiometabolic II | 0.61(0.39,0.94) | 2.36E-02 | 0.308 |
| ENPEP | Q07075 | Cardiometabolic II | 1.31(1.04,1.65) | 2.42E-02 | 0.310 |
| ATP2B4 | P23634 | Cardiometabolic II | 0.72(0.55,0.96) | 2.49E-02 | 0.310 |
| SPINK8 | P0C7L1 | Cardiometabolic II | 0.60(0.39,0.94) | 2.59E-02 | 0.310 |
| CDHR5 | Q9HBB8 | Cardiometabolic | 0.73(0.56,0.96) | 2.61E-02 | 0.310 |
| GRHPR | Q9UBQ7 | Cardiometabolic II | 0.79(0.64,0.97) | 2.64E-02 | 0.310 |
| MB | P02144 | Cardiometabolic | 1.25(1.03,1.52) | 2.65E-02 | 0.310 |
| DPP7 | Q9UHL4 | Cardiometabolic | 0.81(0.67,0.98) | 2.68E-02 | 0.310 |
| ADH4 | P08319 | Cardiometabolic | 0.86(0.75,0.98) | 2.72E-02 | 0.310 |
| ATP6V1G1 | O75348 | Cardiometabolic II | 0.81(0.67,0.98) | 2.77E-02 | 0.310 |
| ANGPTL3 | Q9Y5C1 | Cardiometabolic | 0.70(0.51,0.96) | 2.80E-02 | 0.310 |
| KYAT1 | Q16773 | Cardiometabolic | 0.80(0.65,0.98) | 2.84E-02 | 0.310 |
| SNED1 | Q8TER0 | Cardiometabolic II | 1.50(1.04,2.17) | 2.92E-02 | 0.314 |
| FAP | Q12884 | Cardiometabolic | 0.64(0.42,0.96) | 3.14E-02 | 0.314 |
| HRC | P23327 | Cardiometabolic II | 1.42(1.03,1.95) | 3.21E-02 | 0.314 |
| CTHRC1 | Q96CG8 | Cardiometabolic II | 1.56(1.04,2.35) | 3.26E-02 | 0.314 |
| TIA1 | P31483 | Cardiometabolic | 0.90(0.81,0.99) | 3.27E-02 | 0.314 |
| DTYMK | P23919 | Cardiometabolic II | 0.81(0.67,0.98) | 3.28E-02 | 0.314 |
| LONP1 | P36776 | Cardiometabolic II | 0.84(0.72,0.99) | 3.29E-02 | 0.314 |
| ENTPD5 | O75356 | Cardiometabolic | 0.57(0.34,0.96) | 3.30E-02 | 0.314 |
| FHIP2A | Q5W0V3 | Cardiometabolic II | 1.29(1.02,1.62) | 3.30E-02 | 0.314 |
| PDAP1 | Q13442 | Cardiometabolic II | 0.86(0.75,0.99) | 3.37E-02 | 0.314 |
| RETN | Q9HD89 | Cardiometabolic | 1.32(1.02,1.70) | 3.38E-02 | 0.314 |
| CMC1 | Q7Z7K0 | Cardiometabolic II | 0.85(0.74,0.99) | 3.40E-02 | 0.314 |
| STAB2 | Q8WWQ8 | Cardiometabolic II | 0.62(0.40,0.97) | 3.44E-02 | 0.314 |
| PPIF | P30405 | Cardiometabolic II | 0.78(0.62,0.98) | 3.61E-02 | 0.326 |
| IL6R | P08887 | Cardiometabolic | 0.70(0.50,0.98) | 3.74E-02 | 0.328 |
| DNAJC6 | O75061 | Cardiometabolic II | 0.84(0.72,0.99) | 3.77E-02 | 0.328 |
| PTGR1 | Q14914 | Cardiometabolic II | 0.84(0.72,0.99) | 3.77E-02 | 0.328 |
| RAB10 | P61026 | Cardiometabolic II | 0.77(0.60,0.99) | 4.02E-02 | 0.337 |
| SDC4 | P31431 | Cardiometabolic | 0.78(0.62,0.99) | 4.07E-02 | 0.337 |
| FABP3 | P05413 | Cardiometabolic II | 1.27(1.01,1.59) | 4.08E-02 | 0.337 |
| SERPINE2 | P07093 | Cardiometabolic II | 0.80(0.65,0.99) | 4.18E-02 | 0.337 |
| LRRC59 | Q96AG4 | Cardiometabolic II | 0.74(0.55,0.99) | 4.23E-02 | 0.337 |
| CCER2 | I3L3R5 | Cardiometabolic II | 1.23(1.01,1.51) | 4.28E-02 | 0.337 |
| SORT1 | Q99523 | Cardiometabolic | 0.72(0.53,0.99) | 4.31E-02 | 0.337 |
| RILPL2 | Q969X0 | Cardiometabolic II | 0.87(0.76,1.00) | 4.32E-02 | 0.337 |
| VCAM1 | P19320 | Cardiometabolic | 1.52(1.01,2.28) | 4.40E-02 | 0.337 |
| ACTN2 | P35609 | Cardiometabolic II | 1.24(1.01,1.53) | 4.43E-02 | 0.337 |
| PRG2 | P13727 | Cardiometabolic II | 1.28(1.01,1.63) | 4.53E-02 | 0.337 |
| CEP43 | O95684 | Cardiometabolic | 0.84(0.71,1.00) | 4.71E-02 | 0.337 |
| PNMA1 | Q8ND90 | Cardiometabolic II | 0.77(0.60,1.00) | 4.80E-02 | 0.337 |
| MYDGF | Q969H8 | Cardiometabolic II | 0.87(0.76,1.00) | 4.84E-02 | 0.337 |
| FGFBP2 | Q9BYJ0 | Cardiometabolic II | 0.76(0.59,1.00) | 4.85E-02 | 0.337 |
| LAMP1 | P11279 | Cardiometabolic II | 0.53(0.29,1.00) | 4.85E-02 | 0.337 |
| EFEMP1 | Q12805 | Cardiometabolic | 1.42(1.00,2.01) | 4.86E-02 | 0.337 |
| RAB27B | O00194 | Cardiometabolic II | 0.86(0.74,1.00) | 4.93E-02 | 0.337 |
| L3HYPDH | Q96EM0 | Cardiometabolic II | 0.86(0.74,1.00) | 4.98E-02 | 0.337 |
| FSTL1 | Q12841 | Cardiometabolic II | 1.73(1.00,3.00) | 4.99E-02 | 0.337 |
| TNNI3 | P19429 | Cardiometabolic | 1.15(1.00,1.33) | 5.10E-02 | 0.337 |
| IGSF9 | Q9P2J2 | Cardiometabolic II | 0.86(0.74,1.00) | 5.10E-02 | 0.337 |
| PTGDS | P41222 | Cardiometabolic | 1.42(1.00,2.01) | 5.11E-02 | 0.337 |
| PLAT | P00750 | Cardiometabolic | 0.81(0.65,1.00) | 5.15E-02 | 0.337 |
| DIABLO | Q9NR28 | Cardiometabolic | 0.90(0.81,1.00) | 5.19E-02 | 0.337 |
| S100P | P25815 | Cardiometabolic | 1.19(1.00,1.43) | 5.26E-02 | 0.337 |
| AGXT | P21549 | Cardiometabolic | 0.85(0.73,1.00) | 5.29E-02 | 0.337 |
| EHBP1 | Q8NDI1 | Cardiometabolic II | 0.84(0.70,1.00) | 5.30E-02 | 0.337 |
| RNF149 | Q8NC42 | Cardiometabolic II | 1.49(0.99,2.24) | 5.30E-02 | 0.337 |
| SPON2 | Q9BUD6 | Cardiometabolic | 1.36(1.00,1.87) | 5.33E-02 | 0.337 |
| MEGF9 | Q9H1U4 | Cardiometabolic | 0.56(0.31,1.01) | 5.35E-02 | 0.337 |
| TOMM20 | Q15388 | Cardiometabolic II | 0.84(0.70,1.00) | 5.43E-02 | 0.337 |
| NPTX2 | P47972 | Cardiometabolic II | 1.47(0.99,2.17) | 5.45E-02 | 0.337 |
| CEBPB | P17676 | Cardiometabolic | 1.32(0.99,1.76) | 5.49E-02 | 0.337 |
| CKB | P12277 | Cardiometabolic II | 1.22(1.00,1.49) | 5.52E-02 | 0.337 |
| CSPG4 | Q6UVK1 | Cardiometabolic II | 1.46(0.99,2.14) | 5.64E-02 | 0.337 |
| CHRDL2 | Q6WN34 | Cardiometabolic | 1.25(0.99,1.58) | 5.68E-02 | 0.337 |
| ATP1B3 | P54709 | Cardiometabolic II | 0.67(0.44,1.01) | 5.68E-02 | 0.337 |
| CTLA4 | P16410 | Cardiometabolic II | 0.65(0.42,1.01) | 5.70E-02 | 0.337 |
| BRD3 | Q15059 | Cardiometabolic II | 0.72(0.52,1.01) | 5.71E-02 | 0.337 |
| RYR1 | P21817 | Cardiometabolic II | 0.63(0.40,1.02) | 5.78E-02 | 0.338 |
| CALY | Q9NYX4 | Cardiometabolic II | 0.67(0.44,1.02) | 5.97E-02 | 0.341 |
| PTPRF | P10586 | Cardiometabolic | 0.69(0.47,1.02) | 5.99E-02 | 0.341 |
| ALCAM | Q13740 | Cardiometabolic | 1.74(0.98,3.10) | 6.07E-02 | 0.341 |
| KITLG | P21583 | Cardiometabolic | 1.35(0.99,1.86) | 6.12E-02 | 0.341 |
| LRP11 | Q86VZ4 | Cardiometabolic | 1.33(0.99,1.80) | 6.16E-02 | 0.341 |
| THOP1 | P52888 | Cardiometabolic | 0.70(0.48,1.02) | 6.16E-02 | 0.341 |
| EPHB4 | P54760 | Cardiometabolic | 1.52(0.98,2.35) | 6.20E-02 | 0.341 |
| BRDT | Q58F21 | Cardiometabolic II | 0.70(0.48,1.02) | 6.21E-02 | 0.341 |
| DKK3 | Q9UBP4 | Cardiometabolic | 1.42(0.98,2.06) | 6.38E-02 | 0.348 |
| GMFG | O60234 | Cardiometabolic II | 0.84(0.70,1.01) | 6.45E-02 | 0.349 |
| THPO | P40225 | Cardiometabolic | 0.75(0.56,1.02) | 6.57E-02 | 0.353 |
| MYH9 | P35579 | Cardiometabolic II | 0.90(0.80,1.01) | 6.80E-02 | 0.354 |
| CCL27 | Q9Y4X3 | Cardiometabolic | 0.84(0.69,1.01) | 6.82E-02 | 0.354 |
| EIF2S2 | P20042 | Cardiometabolic II | 0.72(0.51,1.03) | 6.87E-02 | 0.354 |
| NTproBNP | NTproBNP | Cardiometabolic | 1.09(0.99,1.21) | 7.00E-02 | 0.354 |
| BDNF | P23560 | Cardiometabolic II | 0.87(0.74,1.01) | 7.06E-02 | 0.354 |
| ADRA2A | P08913 | Cardiometabolic II | 0.81(0.64,1.02) | 7.07E-02 | 0.354 |
| ADGRG2 | Q8IZP9 | Cardiometabolic | 0.69(0.46,1.03) | 7.09E-02 | 0.354 |
| RAB11FIP3 | O75154 | Cardiometabolic II | 0.90(0.79,1.01) | 7.12E-02 | 0.354 |
| GUSB | P08236 | Cardiometabolic | 0.85(0.71,1.01) | 7.13E-02 | 0.354 |
| MAN1A2 | O60476 | Cardiometabolic II | 0.61(0.36,1.04) | 7.14E-02 | 0.354 |
| PRSS27 | Q9BQR3 | Cardiometabolic | 1.29(0.98,1.71) | 7.15E-02 | 0.354 |
| SARG | Q9BW04 | Cardiometabolic II | 0.90(0.80,1.01) | 7.23E-02 | 0.354 |
| PDIA4 | P13667 | Cardiometabolic II | 0.82(0.65,1.02) | 7.26E-02 | 0.354 |
| TSHB | P01222 | Cardiometabolic | 0.88(0.76,1.01) | 7.28E-02 | 0.354 |
| HS1BP3 | Q53T59 | Cardiometabolic II | 0.87(0.74,1.01) | 7.33E-02 | 0.354 |
| ANPEP | P15144 | Cardiometabolic | 1.43(0.97,2.13) | 7.36E-02 | 0.354 |
| TIMM10 | P62072 | Cardiometabolic II | 0.73(0.52,1.03) | 7.47E-02 | 0.357 |
| GSTA1 | P08263 | Cardiometabolic | 0.89(0.77,1.01) | 7.61E-02 | 0.360 |
| RPS10 | P46783 | Cardiometabolic II | 0.71(0.48,1.04) | 7.64E-02 | 0.360 |
| NAGK | Q9UJ70 | Cardiometabolic II | 0.80(0.63,1.03) | 7.95E-02 | 0.370 |
| PTPRB | P23467 | Cardiometabolic II | 0.67(0.42,1.05) | 7.95E-02 | 0.370 |
| SNAP23 | O00161 | Cardiometabolic | 0.89(0.79,1.01) | 8.03E-02 | 0.370 |
| SERPINA12 | Q8IW75 | Cardiometabolic | 1.09(0.99,1.21) | 8.12E-02 | 0.370 |
| SDC1 | P18827 | Cardiometabolic | 1.28(0.97,1.68) | 8.20E-02 | 0.370 |
| VIT | Q6UXI7 | Cardiometabolic II | 1.35(0.96,1.89) | 8.22E-02 | 0.370 |
| EIF5 | P55010 | Cardiometabolic II | 0.74(0.52,1.04) | 8.26E-02 | 0.370 |
| GP1BB | P13224 | Cardiometabolic II | 0.84(0.69,1.02) | 8.26E-02 | 0.370 |
| ADAM15 | Q13444 | Cardiometabolic | 1.39(0.96,2.02) | 8.35E-02 | 0.372 |
| ITIH3 | Q06033 | Cardiometabolic | 1.31(0.96,1.78) | 8.62E-02 | 0.382 |
| CRTAC1 | Q9NQ79 | Cardiometabolic | 1.40(0.95,2.06) | 8.91E-02 | 0.392 |
| GRAP2 | O75791 | Cardiometabolic | 0.93(0.85,1.01) | 9.17E-02 | 0.393 |
| DOK2 | O60496 | Cardiometabolic | 0.92(0.84,1.01) | 9.19E-02 | 0.393 |
| C2 | P06681 | Cardiometabolic | 0.71(0.48,1.06) | 9.30E-02 | 0.393 |
| ASAH1 | Q13510 | Cardiometabolic II | 0.77(0.56,1.05) | 9.30E-02 | 0.393 |
| CLEC1A | Q8NC01 | Cardiometabolic | 1.34(0.95,1.88) | 9.31E-02 | 0.393 |
| NPL | Q9BXD5 | Cardiometabolic II | 0.78(0.59,1.04) | 9.33E-02 | 0.393 |
| SHISA5 | Q8N114 | Cardiometabolic II | 1.45(0.94,2.23) | 9.34E-02 | 0.393 |
| RAB33A | Q14088 | Cardiometabolic II | 0.83(0.68,1.03) | 9.39E-02 | 0.393 |
| PRCP | P42785 | Cardiometabolic | 0.74(0.52,1.05) | 9.44E-02 | 0.393 |
| AMDHD2 | Q9Y303 | Cardiometabolic II | 0.66(0.41,1.07) | 9.50E-02 | 0.393 |
| SERPINE1 | P05121 | Cardiometabolic | 0.87(0.74,1.02) | 9.59E-02 | 0.393 |
| NPDC1 | Q9NQX5 | Cardiometabolic | 1.33(0.95,1.86) | 9.62E-02 | 0.393 |
| PLXNB3 | Q9ULL4 | Cardiometabolic | 0.81(0.63,1.04) | 9.66E-02 | 0.393 |
| TCTN3 | Q6NUS6 | Cardiometabolic II | 0.69(0.44,1.07) | 9.68E-02 | 0.393 |
| RNF5 | Q99942 | Cardiometabolic II | 0.86(0.72,1.03) | 9.93E-02 | 0.401 |
| GAMT | Q14353 | Cardiometabolic II | 0.68(0.43,1.08) | 1.01E-01 | 0.406 |
| THBD | P07204 | Cardiometabolic | 1.36(0.94,1.98) | 1.03E-01 | 0.406 |
| VSIR | Q9H7M9 | Cardiometabolic | 0.85(0.70,1.03) | 1.03E-01 | 0.406 |
| SCRIB | Q14160 | Cardiometabolic II | 0.87(0.74,1.03) | 1.03E-01 | 0.406 |
| COX6B1 | P14854 | Cardiometabolic II | 0.71(0.46,1.08) | 1.04E-01 | 0.406 |
| SUSD1 | Q6UWL2 | Cardiometabolic | 0.83(0.66,1.04) | 1.05E-01 | 0.406 |
| MECR | Q9BV79 | Cardiometabolic II | 0.88(0.76,1.03) | 1.05E-01 | 0.406 |
| NFKB1 | P19838 | Cardiometabolic II | 0.86(0.71,1.03) | 1.05E-01 | 0.406 |
| RIPK4 | P57078 | Cardiometabolic II | 0.82(0.65,1.04) | 1.06E-01 | 0.408 |
| MCAM | P43121 | Cardiometabolic | 1.31(0.94,1.82) | 1.08E-01 | 0.411 |
| NOP56 | O00567 | Cardiometabolic II | 0.89(0.77,1.03) | 1.08E-01 | 0.411 |
| SYTL4 | Q96C24 | Cardiometabolic II | 0.86(0.71,1.04) | 1.11E-01 | 0.420 |
| NIT1 | Q86X76 | Cardiometabolic II | 0.80(0.61,1.05) | 1.12E-01 | 0.420 |
| OPLAH | O14841 | Cardiometabolic II | 0.85(0.70,1.04) | 1.12E-01 | 0.420 |
| CA4 | P22748 | Cardiometabolic | 1.45(0.91,2.30) | 1.16E-01 | 0.430 |
| LGALS3 | P17931 | Cardiometabolic | 1.35(0.93,1.97) | 1.16E-01 | 0.430 |
| CBX2 | Q14781 | Cardiometabolic II | 0.73(0.49,1.08) | 1.18E-01 | 0.431 |
| NT5C | Q8TCD5 | Cardiometabolic II | 0.87(0.73,1.04) | 1.18E-01 | 0.431 |
| PYY | P10082 | Cardiometabolic II | 0.90(0.79,1.03) | 1.18E-01 | 0.431 |
| PDZD2 | O15018 | Cardiometabolic II | 0.82(0.64,1.05) | 1.20E-01 | 0.433 |
| IL19 | Q9UHD0 | Cardiometabolic | 1.13(0.97,1.32) | 1.21E-01 | 0.433 |
| POSTN | Q15063 | Cardiometabolic II | 1.31(0.93,1.84) | 1.21E-01 | 0.433 |
| PDGFA | P04085 | Cardiometabolic | 0.88(0.76,1.03) | 1.22E-01 | 0.433 |
| TNC | P24821 | Cardiometabolic | 1.20(0.95,1.51) | 1.22E-01 | 0.433 |
| HPSE | Q9Y251 | Cardiometabolic II | 0.89(0.78,1.03) | 1.22E-01 | 0.433 |
| CDH2 | P19022 | Cardiometabolic | 0.78(0.57,1.07) | 1.24E-01 | 0.436 |
| HIP1 | O00291 | Cardiometabolic II | 0.69(0.43,1.11) | 1.24E-01 | 0.436 |
| COL18A1 | P39060 | Cardiometabolic | 1.45(0.90,2.33) | 1.26E-01 | 0.437 |
| SFTPD | P35247 | Cardiometabolic | 0.88(0.74,1.04) | 1.26E-01 | 0.437 |
| SGSH | P51688 | Cardiometabolic II | 0.82(0.64,1.06) | 1.26E-01 | 0.437 |
| HSDL2 | Q6YN16 | Cardiometabolic II | 0.80(0.60,1.07) | 1.29E-01 | 0.445 |
| GYS1 | P13807 | Cardiometabolic | 0.91(0.80,1.03) | 1.31E-01 | 0.450 |
| LEP | P41159 | Cardiometabolic | 0.92(0.82,1.03) | 1.32E-01 | 0.451 |
| STK4 | Q13043 | Cardiometabolic | 0.90(0.79,1.03) | 1.33E-01 | 0.452 |
| IL18BP | O95998 | Cardiometabolic | 1.31(0.92,1.87) | 1.34E-01 | 0.453 |
| TPK1 | Q9H3S4 | Cardiometabolic II | 0.69(0.42,1.12) | 1.35E-01 | 0.454 |
| FAM3C | Q92520 | Cardiometabolic | 1.32(0.92,1.92) | 1.36E-01 | 0.454 |
| GZMH | P20718 | Cardiometabolic | 0.89(0.76,1.04) | 1.36E-01 | 0.454 |
| EEF1D | P29692 | Cardiometabolic II | 0.90(0.78,1.04) | 1.37E-01 | 0.455 |
| CTSD | P07339 | Cardiometabolic | 0.79(0.58,1.08) | 1.38E-01 | 0.456 |
| SSC4D | Q8WTU2 | Cardiometabolic | 0.93(0.85,1.02) | 1.39E-01 | 0.458 |
| CD2AP | Q9Y5K6 | Cardiometabolic | 0.88(0.74,1.04) | 1.41E-01 | 0.462 |
| SLC12A2 | P55011 | Cardiometabolic II | 0.67(0.39,1.14) | 1.42E-01 | 0.463 |
| INPP5D | Q92835 | Cardiometabolic II | 0.84(0.66,1.06) | 1.44E-01 | 0.466 |
| SEC31A | O94979 | Cardiometabolic II | 0.88(0.74,1.04) | 1.44E-01 | 0.466 |
| RBPMS2 | Q6ZRY4 | Cardiometabolic II | 0.91(0.80,1.03) | 1.45E-01 | 0.466 |
| BECN1 | Q14457 | Cardiometabolic II | 0.84(0.67,1.06) | 1.46E-01 | 0.466 |
| CTF1 | Q16619 | Cardiometabolic | 0.90(0.78,1.04) | 1.47E-01 | 0.466 |
| SLC51B | Q86UW2 | Cardiometabolic II | 0.87(0.71,1.05) | 1.47E-01 | 0.466 |
| CANT1 | Q8WVQ1 | Cardiometabolic | 0.66(0.38,1.16) | 1.48E-01 | 0.466 |
| CST3 | P01034 | Cardiometabolic | 1.31(0.91,1.90) | 1.48E-01 | 0.466 |
| F9 | P00740 | Cardiometabolic | 0.65(0.36,1.17) | 1.50E-01 | 0.469 |
| MMP7 | P09237 | Cardiometabolic | 1.19(0.94,1.52) | 1.50E-01 | 0.469 |
| PMM2 | O15305 | Cardiometabolic II | 0.85(0.67,1.06) | 1.51E-01 | 0.470 |
| COPB2 | P35606 | Cardiometabolic II | 0.79(0.57,1.09) | 1.52E-01 | 0.471 |
| CHEK2 | O96017 | Cardiometabolic | 1.29(0.91,1.82) | 1.54E-01 | 0.473 |
| ECHDC3 | Q96DC8 | Cardiometabolic II | 0.87(0.72,1.05) | 1.54E-01 | 0.473 |
| CDH17 | Q12864 | Cardiometabolic | 0.88(0.75,1.05) | 1.56E-01 | 0.473 |
| IL2RA | P01589 | Cardiometabolic | 1.21(0.93,1.57) | 1.57E-01 | 0.473 |
| TFPI | P10646 | Cardiometabolic | 0.76(0.51,1.11) | 1.57E-01 | 0.473 |
| VSIG10L | Q86VR7 | Cardiometabolic II | 1.32(0.90,1.93) | 1.57E-01 | 0.473 |
| CRHR1 | P34998 | Cardiometabolic | 1.13(0.95,1.34) | 1.58E-01 | 0.473 |
| NIT2 | Q9NQR4 | Cardiometabolic II | 0.88(0.73,1.05) | 1.58E-01 | 0.473 |
| SERPINA11 | Q86U17 | Cardiometabolic | 1.22(0.92,1.60) | 1.60E-01 | 0.477 |
| BHMT2 | Q9H2M3 | Cardiometabolic II | 0.69(0.41,1.16) | 1.63E-01 | 0.483 |
| TIMD4 | Q96H15 | Cardiometabolic | 1.20(0.93,1.55) | 1.64E-01 | 0.483 |
| CPXM2 | Q8N436 | Cardiometabolic II | 1.42(0.87,2.33) | 1.64E-01 | 0.483 |
| TP53INP1 | Q96A56 | Cardiometabolic | 0.73(0.47,1.14) | 1.65E-01 | 0.484 |
| CNST | Q6PJW8 | Cardiometabolic | 0.93(0.84,1.03) | 1.67E-01 | 0.485 |
| EHD3 | Q9NZN3 | Cardiometabolic II | 0.92(0.82,1.03) | 1.67E-01 | 0.485 |
| FUCA1 | P04066 | Cardiometabolic | 0.91(0.81,1.04) | 1.68E-01 | 0.485 |
| PPP1R2 | P41236 | Cardiometabolic | 0.90(0.78,1.04) | 1.68E-01 | 0.485 |
| NRP1 | O14786 | Cardiometabolic | 1.33(0.88,2.01) | 1.69E-01 | 0.486 |
| OSMR | Q99650 | Cardiometabolic | 1.49(0.84,2.61) | 1.71E-01 | 0.486 |
| TNFRSF10C | O14798 | Cardiometabolic | 1.18(0.93,1.49) | 1.71E-01 | 0.486 |
| ADAMTSL4 | Q6UY14 | Cardiometabolic II | 1.48(0.84,2.60) | 1.71E-01 | 0.486 |
| TPR | P12270 | Cardiometabolic II | 0.80(0.58,1.10) | 1.72E-01 | 0.487 |
| QDPR | P09417 | Cardiometabolic | 0.83(0.64,1.09) | 1.74E-01 | 0.490 |
| ANXA4 | P09525 | Cardiometabolic | 0.89(0.76,1.05) | 1.75E-01 | 0.490 |
| TSPAN15 | O95858 | Cardiometabolic II | 0.82(0.61,1.09) | 1.75E-01 | 0.490 |
| CLEC4M | Q9H2X3 | Cardiometabolic II | 0.83(0.63,1.09) | 1.77E-01 | 0.494 |
| TMED10 | P49755 | Cardiometabolic II | 0.77(0.53,1.13) | 1.79E-01 | 0.497 |
| REG1B | P48304 | Cardiometabolic | 1.14(0.94,1.37) | 1.80E-01 | 0.497 |
| CCDC80 | Q76M96 | Cardiometabolic | 1.22(0.91,1.64) | 1.81E-01 | 0.497 |
| TFRC | P02786 | Cardiometabolic | 1.22(0.91,1.62) | 1.82E-01 | 0.497 |
| GADD45GIP1 | Q8TAE8 | Cardiometabolic II | 0.83(0.64,1.09) | 1.82E-01 | 0.497 |
| CA5A | P35218 | Cardiometabolic | 0.92(0.80,1.04) | 1.83E-01 | 0.497 |
| TCOF1 | Q13428 | Cardiometabolic II | 0.74(0.47,1.15) | 1.83E-01 | 0.497 |
| IL6ST | P40189 | Cardiometabolic | 1.55(0.81,2.98) | 1.86E-01 | 0.503 |
| ADA2 | Q9NZK5 | Cardiometabolic | 1.19(0.92,1.55) | 1.87E-01 | 0.503 |
| CA13 | Q8N1Q1 | Cardiometabolic | 0.93(0.84,1.04) | 1.87E-01 | 0.503 |
| SLITRK6 | Q9H5Y7 | Cardiometabolic | 1.25(0.90,1.74) | 1.89E-01 | 0.504 |
| CRYBB1 | P53674 | Cardiometabolic II | 0.87(0.70,1.07) | 1.89E-01 | 0.504 |
| MYOM2 | P54296 | Cardiometabolic II | 1.19(0.92,1.53) | 1.90E-01 | 0.505 |
| SNX5 | Q9Y5X3 | Cardiometabolic II | 0.72(0.45,1.17) | 1.91E-01 | 0.506 |
| VSTM2L | Q96N03 | Cardiometabolic | 1.12(0.94,1.34) | 1.94E-01 | 0.511 |
| B3GAT3 | O94766 | Cardiometabolic II | 0.63(0.31,1.27) | 1.95E-01 | 0.511 |
| CRYZL1 | O95825 | Cardiometabolic II | 0.91(0.78,1.05) | 1.95E-01 | 0.511 |
| NADK | O95544 | Cardiometabolic | 1.14(0.94,1.38) | 1.96E-01 | 0.511 |
| ITPA | Q9BY32 | Cardiometabolic II | 0.87(0.71,1.07) | 1.97E-01 | 0.511 |
| ITPR1 | Q14643 | Cardiometabolic II | 0.87(0.70,1.08) | 1.97E-01 | 0.511 |
| NECTIN2 | Q92692 | Cardiometabolic | 1.27(0.88,1.84) | 2.00E-01 | 0.515 |
| PRRT3 | Q5FWE3 | Cardiometabolic II | 1.25(0.89,1.76) | 2.00E-01 | 0.515 |
| GATD3 | P0DPI2 | Cardiometabolic II | 0.89(0.74,1.06) | 2.02E-01 | 0.517 |
| ADAMTSL5 | Q6ZMM2 | Cardiometabolic II | 1.25(0.89,1.75) | 2.03E-01 | 0.517 |
| MYL1 | P05976 | Cardiometabolic II | 1.21(0.90,1.61) | 2.03E-01 | 0.517 |
| CCL15 | Q16663 | Cardiometabolic | 1.17(0.91,1.51) | 2.08E-01 | 0.524 |
| FABP2 | P12104 | Cardiometabolic | 1.09(0.95,1.26) | 2.08E-01 | 0.524 |
| USP8 | P40818 | Cardiometabolic | 0.92(0.82,1.05) | 2.08E-01 | 0.524 |
| GP2 | P55259 | Cardiometabolic | 1.09(0.95,1.24) | 2.10E-01 | 0.528 |
| PACS2 | Q86VP3 | Cardiometabolic II | 0.87(0.70,1.08) | 2.11E-01 | 0.528 |
| COMMD1 | Q8N668 | Cardiometabolic II | 0.87(0.69,1.09) | 2.13E-01 | 0.531 |
| CLTA | P09496 | Cardiometabolic | 0.85(0.66,1.10) | 2.14E-01 | 0.532 |
| TSLP | Q969D9 | Cardiometabolic | 0.87(0.71,1.08) | 2.15E-01 | 0.533 |
| MSMB | P08118 | Cardiometabolic | 1.10(0.94,1.29) | 2.17E-01 | 0.534 |
| NFE2 | Q16621 | Cardiometabolic II | 0.89(0.74,1.07) | 2.17E-01 | 0.534 |
| BPIFB2 | Q8N4F0 | Cardiometabolic II | 0.89(0.73,1.07) | 2.20E-01 | 0.535 |
| CSDE1 | O75534 | Cardiometabolic II | 0.93(0.82,1.05) | 2.20E-01 | 0.535 |
| CRX | O43186 | Cardiometabolic | 1.06(0.96,1.17) | 2.21E-01 | 0.535 |
| GPNMB | Q14956 | Cardiometabolic | 1.29(0.86,1.92) | 2.21E-01 | 0.535 |
| GBP6 | Q6ZN66 | Cardiometabolic II | 0.80(0.56,1.14) | 2.21E-01 | 0.535 |
| PPM1F | P49593 | Cardiometabolic II | 0.85(0.66,1.10) | 2.22E-01 | 0.536 |
| FADD | Q13158 | Cardiometabolic | 0.91(0.78,1.06) | 2.23E-01 | 0.536 |
| CNP | P09543 | Cardiometabolic II | 0.90(0.76,1.07) | 2.28E-01 | 0.546 |
| FRMD4B | Q9Y2L6 | Cardiometabolic II | 0.89(0.73,1.08) | 2.29E-01 | 0.547 |
| NPPB | P16860 | Cardiometabolic | 1.05(0.97,1.14) | 2.31E-01 | 0.548 |
| PDGFRB | P09619 | Cardiometabolic | 1.22(0.88,1.70) | 2.31E-01 | 0.548 |
| RPL14 | P50914 | Cardiometabolic II | 0.80(0.55,1.16) | 2.32E-01 | 0.549 |
| QPCT | Q16769 | Cardiometabolic | 1.29(0.85,1.96) | 2.33E-01 | 0.549 |
| EXOSC10 | Q01780 | Cardiometabolic II | 0.85(0.65,1.11) | 2.36E-01 | 0.555 |
| DENND2B | P78524 | Cardiometabolic II | 1.14(0.92,1.41) | 2.38E-01 | 0.558 |
| MMUT | P22033 | Cardiometabolic II | 0.85(0.64,1.12) | 2.40E-01 | 0.561 |
| IGFBP6 | P24592 | Cardiometabolic | 1.23(0.87,1.75) | 2.42E-01 | 0.561 |
| CD69 | Q07108 | Cardiometabolic | 0.93(0.81,1.05) | 2.43E-01 | 0.561 |
| ITGB1 | P05556 | Cardiometabolic | 0.72(0.42,1.25) | 2.44E-01 | 0.561 |
| PECR | Q9BY49 | Cardiometabolic II | 0.74(0.44,1.23) | 2.44E-01 | 0.561 |
| PGLYRP4 | Q96LB8 | Cardiometabolic II | 0.86(0.67,1.11) | 2.44E-01 | 0.561 |
| LPP | Q93052 | Cardiometabolic II | 0.88(0.72,1.09) | 2.45E-01 | 0.561 |
| SELP | P16109 | Cardiometabolic | 0.89(0.72,1.09) | 2.49E-01 | 0.569 |
| HEBP1 | Q9NRV9 | Cardiometabolic | 0.87(0.69,1.10) | 2.52E-01 | 0.574 |
| LACTB2 | Q53H82 | Cardiometabolic | 0.90(0.76,1.08) | 2.53E-01 | 0.574 |
| TCN2 | P20062 | Cardiometabolic | 1.22(0.87,1.72) | 2.57E-01 | 0.582 |
| FBLN2 | P98095 | Cardiometabolic II | 1.26(0.85,1.87) | 2.58E-01 | 0.582 |
| GPR37 | O15354 | Cardiometabolic | 1.10(0.93,1.31) | 2.59E-01 | 0.583 |
| CNTN1 | Q12860 | Cardiometabolic | 1.26(0.84,1.90) | 2.60E-01 | 0.583 |
| MAP1LC3B2 | A6NCE7 | Cardiometabolic II | 0.87(0.68,1.11) | 2.63E-01 | 0.587 |
| AHCY | P23526 | Cardiometabolic | 0.91(0.77,1.07) | 2.64E-01 | 0.587 |
| ACSL1 | P33121 | Cardiometabolic II | 0.79(0.53,1.19) | 2.65E-01 | 0.587 |
| KLK3 | P07288 | Cardiometabolic II | 0.94(0.83,1.05) | 2.65E-01 | 0.587 |
| TNFSF8 | P32971 | Cardiometabolic II | 1.23(0.86,1.76) | 2.66E-01 | 0.587 |
| FCGR2A | P12318 | Cardiometabolic | 0.91(0.76,1.08) | 2.68E-01 | 0.589 |
| GAS6 | Q14393 | Cardiometabolic | 1.29(0.82,2.03) | 2.69E-01 | 0.589 |
| BAG6 | P46379 | Cardiometabolic | 0.83(0.60,1.15) | 2.70E-01 | 0.589 |
| PTN | P21246 | Cardiometabolic | 1.14(0.90,1.43) | 2.72E-01 | 0.589 |
| ZP3 | P21754 | Cardiometabolic II | 0.97(0.93,1.02) | 2.73E-01 | 0.589 |
| ACP5 | P13686 | Cardiometabolic | 1.22(0.86,1.73) | 2.74E-01 | 0.589 |
| FBP1 | P09467 | Cardiometabolic | 0.92(0.79,1.07) | 2.74E-01 | 0.589 |
| IGFBP7 | Q16270 | Cardiometabolic | 1.22(0.85,1.75) | 2.74E-01 | 0.589 |
| MET | P08581 | Cardiometabolic | 0.73(0.42,1.28) | 2.74E-01 | 0.589 |
| PLPBP | O94903 | Cardiometabolic | 0.91(0.77,1.08) | 2.75E-01 | 0.589 |
| PCDH17 | O14917 | Cardiometabolic | 0.84(0.61,1.15) | 2.76E-01 | 0.589 |
| GALNT5 | Q7Z7M9 | Cardiometabolic II | 0.77(0.48,1.24) | 2.77E-01 | 0.589 |
| TIMP2 | P16035 | Cardiometabolic II | 1.26(0.83,1.93) | 2.77E-01 | 0.589 |
| CASP3 | P42574 | Cardiometabolic | 0.93(0.82,1.06) | 2.79E-01 | 0.589 |
| RARRES2 | Q99969 | Cardiometabolic | 0.88(0.70,1.11) | 2.79E-01 | 0.589 |
| IGHMBP2 | P38935 | Cardiometabolic II | 0.87(0.67,1.12) | 2.80E-01 | 0.590 |
| CTSZ | Q9UBR2 | Cardiometabolic | 0.83(0.59,1.17) | 2.82E-01 | 0.591 |
| COL4A1 | P02462 | Cardiometabolic | 1.17(0.88,1.55) | 2.84E-01 | 0.591 |
| CR2 | P20023 | Cardiometabolic | 1.14(0.90,1.44) | 2.84E-01 | 0.591 |
| MCFD2 | Q8NI22 | Cardiometabolic | 0.87(0.66,1.13) | 2.84E-01 | 0.591 |
| UPB1 | Q9UBR1 | Cardiometabolic II | 0.92(0.80,1.07) | 2.86E-01 | 0.594 |
| FCGR3B | O75015 | Cardiometabolic | 1.13(0.90,1.43) | 2.90E-01 | 0.598 |
| NCAM1 | P13591 | Cardiometabolic | 1.22(0.85,1.76) | 2.90E-01 | 0.598 |
| YOD1 | Q5VVQ6 | Cardiometabolic II | 0.89(0.72,1.10) | 2.92E-01 | 0.598 |
| ACOX1 | Q15067 | Cardiometabolic | 0.88(0.70,1.12) | 2.93E-01 | 0.598 |
| GGACT | Q9BVM4 | Cardiometabolic II | 0.90(0.75,1.09) | 2.93E-01 | 0.598 |
| IGSF21 | Q96ID5 | Cardiometabolic II | 0.82(0.56,1.19) | 2.93E-01 | 0.598 |
| REG1A | P05451 | Cardiometabolic | 1.12(0.90,1.39) | 2.98E-01 | 0.604 |
| IDO1 | P14902 | Cardiometabolic II | 1.12(0.91,1.38) | 2.98E-01 | 0.604 |
| MPHOSPH8 | Q99549 | Cardiometabolic | 0.92(0.79,1.08) | 2.99E-01 | 0.604 |
| PLA2G1B | P04054 | Cardiometabolic | 1.14(0.89,1.47) | 2.99E-01 | 0.604 |
| OLR1 | P78380 | Cardiometabolic | 1.10(0.91,1.34) | 3.02E-01 | 0.607 |
| FAS | P25445 | Cardiometabolic | 1.16(0.87,1.55) | 3.04E-01 | 0.607 |
| PEAR1 | Q5VY43 | Cardiometabolic | 1.30(0.79,2.14) | 3.04E-01 | 0.607 |
| ACY3 | Q96HD9 | Cardiometabolic II | 0.88(0.68,1.13) | 3.04E-01 | 0.607 |
| COMT | P21964 | Cardiometabolic | 0.93(0.81,1.07) | 3.09E-01 | 0.615 |
| STK11 | Q15831 | Cardiometabolic | 0.92(0.78,1.08) | 3.11E-01 | 0.616 |
| GIP | P09681 | Cardiometabolic II | 0.89(0.72,1.11) | 3.11E-01 | 0.616 |
| FCN2 | Q15485 | Cardiometabolic | 0.87(0.67,1.14) | 3.12E-01 | 0.616 |
| ABRAXAS2 | Q15018 | Cardiometabolic II | 0.90(0.73,1.11) | 3.16E-01 | 0.623 |
| DLK1 | P80370 | Cardiometabolic | 0.90(0.74,1.11) | 3.21E-01 | 0.625 |
| DPT | Q07507 | Cardiometabolic | 1.24(0.81,1.89) | 3.22E-01 | 0.625 |
| ASRGL1 | Q7L266 | Cardiometabolic II | 0.94(0.82,1.07) | 3.22E-01 | 0.625 |
| SCPEP1 | Q9HB40 | Cardiometabolic II | 0.87(0.66,1.15) | 3.22E-01 | 0.625 |
| IGFBPL1 | Q8WX77 | Cardiometabolic | 1.18(0.85,1.63) | 3.23E-01 | 0.625 |
| CSRP3 | P50461 | Cardiometabolic II | 1.07(0.94,1.22) | 3.23E-01 | 0.625 |
| PRKD2 | Q9BZL6 | Cardiometabolic II | 0.84(0.60,1.18) | 3.25E-01 | 0.625 |
| CD2 | P06729 | Cardiometabolic II | 1.17(0.86,1.58) | 3.26E-01 | 0.625 |
| GET3 | O43681 | Cardiometabolic II | 0.88(0.67,1.14) | 3.27E-01 | 0.625 |
| MAMDC2 | Q7Z304 | Cardiometabolic II | 1.20(0.84,1.71) | 3.27E-01 | 0.625 |
| SBSN | Q6UWP8 | Cardiometabolic II | 1.20(0.83,1.74) | 3.28E-01 | 0.625 |
| PILRB | Q9UKJ0 | Cardiometabolic | 1.10(0.91,1.35) | 3.29E-01 | 0.625 |
| ADAMTSL2 | Q86TH1 | Cardiometabolic II | 1.20(0.83,1.74) | 3.29E-01 | 0.625 |
| PKD2 | Q13563 | Cardiometabolic II | 0.89(0.70,1.13) | 3.30E-01 | 0.625 |
| HNRNPK | P61978 | Cardiometabolic | 0.93(0.80,1.08) | 3.31E-01 | 0.625 |
| TOR1AIP1 | Q5JTV8 | Cardiometabolic II | 0.87(0.66,1.15) | 3.31E-01 | 0.625 |
| IFNW1 | P05000 | Cardiometabolic II | 0.81(0.53,1.24) | 3.32E-01 | 0.625 |
| SNX9 | Q9Y5X1 | Cardiometabolic | 0.92(0.78,1.09) | 3.33E-01 | 0.625 |
| FETUB | Q9UGM5 | Cardiometabolic | 0.85(0.60,1.19) | 3.34E-01 | 0.625 |
| ADGRE5 | P48960 | Cardiometabolic | 1.19(0.83,1.71) | 3.35E-01 | 0.625 |
| CEACAM8 | P31997 | Cardiometabolic | 1.12(0.89,1.40) | 3.35E-01 | 0.625 |
| ARL2BP | Q9Y2Y0 | Cardiometabolic II | 0.85(0.60,1.19) | 3.38E-01 | 0.629 |
| SNU13 | P55769 | Cardiometabolic II | 0.89(0.70,1.13) | 3.40E-01 | 0.631 |
| ACAN | P16112 | Cardiometabolic | 1.22(0.81,1.82) | 3.44E-01 | 0.637 |
| ICAM2 | P13598 | Cardiometabolic | 1.18(0.83,1.69) | 3.46E-01 | 0.637 |
| GGCT | O75223 | Cardiometabolic II | 0.87(0.66,1.16) | 3.46E-01 | 0.637 |
| GCLM | P48507 | Cardiometabolic II | 0.86(0.63,1.18) | 3.50E-01 | 0.641 |
| KRT17 | Q04695 | Cardiometabolic II | 0.84(0.58,1.21) | 3.50E-01 | 0.641 |
| NUDT15 | Q9NV35 | Cardiometabolic II | 0.87(0.65,1.17) | 3.51E-01 | 0.641 |
| TSPAN1 | O60635 | Cardiometabolic | 0.92(0.78,1.09) | 3.56E-01 | 0.649 |
| LPL | P06858 | Cardiometabolic | 1.12(0.88,1.43) | 3.57E-01 | 0.649 |
| MRPL24 | Q96A35 | Cardiometabolic II | 0.83(0.55,1.24) | 3.58E-01 | 0.649 |
| IGFBP1 | P08833 | Cardiometabolic | 1.04(0.95,1.14) | 3.59E-01 | 0.650 |
| TMED4 | Q7Z7H5 | Cardiometabolic II | 0.88(0.68,1.15) | 3.62E-01 | 0.653 |
| PDE4D | Q08499 | Cardiometabolic II | 0.89(0.69,1.15) | 3.65E-01 | 0.655 |
| ZBTB17 | Q13105 | Cardiometabolic | 1.13(0.87,1.46) | 3.67E-01 | 0.655 |
| NECAP2 | Q9NVZ3 | Cardiometabolic II | 0.91(0.74,1.12) | 3.67E-01 | 0.655 |
| NRCAM | Q92823 | Cardiometabolic | 1.20(0.81,1.77) | 3.68E-01 | 0.655 |
| PPIB | P23284 | Cardiometabolic | 0.95(0.84,1.07) | 3.72E-01 | 0.655 |
| VAMP5 | O95183 | Cardiometabolic | 0.90(0.71,1.14) | 3.72E-01 | 0.655 |
| PALM3 | A6NDB9 | Cardiometabolic II | 0.91(0.74,1.12) | 3.72E-01 | 0.655 |
| CHL1 | O00533 | Cardiometabolic | 1.18(0.82,1.72) | 3.73E-01 | 0.655 |
| TGFBR3 | Q03167 | Cardiometabolic | 1.18(0.82,1.71) | 3.73E-01 | 0.655 |
| ROBO4 | Q8WZ75 | Cardiometabolic II | 1.41(0.66,2.98) | 3.73E-01 | 0.655 |
| SLC4A1 | P02730 | Cardiometabolic II | 0.88(0.66,1.17) | 3.73E-01 | 0.655 |
| CCN3 | P48745 | Cardiometabolic | 1.16(0.84,1.59) | 3.76E-01 | 0.658 |
| FABP6 | P51161 | Cardiometabolic | 1.09(0.90,1.33) | 3.76E-01 | 0.658 |
| ADAMTS16 | Q8TE57 | Cardiometabolic | 1.16(0.83,1.62) | 3.78E-01 | 0.659 |
| HMGCS1 | Q01581 | Cardiometabolic II | 0.87(0.63,1.19) | 3.79E-01 | 0.660 |
| LILRA5 | A6NI73 | Cardiometabolic | 1.16(0.83,1.63) | 3.80E-01 | 0.660 |
| TREH | O43280 | Cardiometabolic II | 0.93(0.78,1.10) | 3.82E-01 | 0.660 |
| MEGF11 | A6BM72 | Cardiometabolic II | 0.82(0.52,1.28) | 3.83E-01 | 0.660 |
| GDF2 | Q9UK05 | Cardiometabolic | 1.15(0.84,1.58) | 3.85E-01 | 0.660 |
| HK2 | P52789 | Cardiometabolic | 1.20(0.79,1.81) | 3.86E-01 | 0.660 |
| NID1 | P14543 | Cardiometabolic | 0.88(0.65,1.18) | 3.86E-01 | 0.660 |
| SPARCL1 | Q14515 | Cardiometabolic | 1.19(0.80,1.77) | 3.87E-01 | 0.660 |
| ADGRF5 | Q8IZF2 | Cardiometabolic II | 0.81(0.51,1.30) | 3.88E-01 | 0.660 |
| HSPB1 | P04792 | Cardiometabolic | 0.93(0.79,1.10) | 3.89E-01 | 0.660 |
| PRTN3 | P24158 | Cardiometabolic | 1.11(0.88,1.39) | 3.89E-01 | 0.660 |
| FAM20A | Q96MK3 | Cardiometabolic II | 0.82(0.52,1.29) | 3.89E-01 | 0.660 |
| LBP | P18428 | Cardiometabolic | 1.09(0.90,1.32) | 3.90E-01 | 0.660 |
| PROCR | Q9UNN8 | Cardiometabolic II | 0.83(0.53,1.28) | 3.91E-01 | 0.660 |
| ARL13B | Q3SXY8 | Cardiometabolic II | 0.92(0.75,1.12) | 3.92E-01 | 0.660 |
| CCL5 | P13501 | Cardiometabolic | 0.95(0.85,1.07) | 3.93E-01 | 0.660 |
| ATP6V1G2 | O95670 | Cardiometabolic II | 0.94(0.81,1.09) | 3.97E-01 | 0.663 |
| CSF2 | P04141 | Cardiometabolic II | 0.85(0.57,1.25) | 3.98E-01 | 0.663 |
| MED21 | Q13503 | Cardiometabolic II | 0.88(0.65,1.19) | 3.98E-01 | 0.663 |
| FCRL1 | Q96LA6 | Cardiometabolic | 1.11(0.87,1.41) | 3.99E-01 | 0.663 |
| BGLAP | P02818 | Cardiometabolic II | 1.07(0.92,1.24) | 4.00E-01 | 0.663 |
| RNASET2 | O00584 | Cardiometabolic | 1.19(0.79,1.78) | 4.01E-01 | 0.663 |
| ICAM1 | P05362 | Cardiometabolic | 1.18(0.80,1.75) | 4.03E-01 | 0.663 |
| CEP170 | Q5SW79 | Cardiometabolic II | 0.93(0.78,1.11) | 4.03E-01 | 0.663 |
| FCAMR | Q8WWV6 | Cardiometabolic II | 0.93(0.78,1.10) | 4.03E-01 | 0.663 |
| PAM | P19021 | Cardiometabolic | 1.19(0.79,1.79) | 4.07E-01 | 0.667 |
| GASK1A | Q9UFP1 | Cardiometabolic II | 0.87(0.64,1.20) | 4.07E-01 | 0.667 |
| XG | P55808 | Cardiometabolic | 1.15(0.82,1.62) | 4.09E-01 | 0.667 |
| UBE2L6 | O14933 | Cardiometabolic II | 0.91(0.73,1.13) | 4.09E-01 | 0.667 |
| CBS | P35520 | Cardiometabolic II | 0.89(0.68,1.17) | 4.15E-01 | 0.673 |
| NOS2 | P35228 | Cardiometabolic II | 1.14(0.83,1.57) | 4.15E-01 | 0.673 |
| SART1 | O43290 | Cardiometabolic II | 1.14(0.83,1.57) | 4.15E-01 | 0.673 |
| TYMP | P19971 | Cardiometabolic | 0.93(0.78,1.11) | 4.18E-01 | 0.676 |
| MSTN | O14793 | Cardiometabolic | 1.09(0.88,1.36) | 4.20E-01 | 0.676 |
| CLC | Q05315 | Cardiometabolic | 0.93(0.78,1.11) | 4.21E-01 | 0.676 |
| TALDO1 | P37837 | Cardiometabolic II | 0.88(0.65,1.20) | 4.22E-01 | 0.676 |
| TNN | Q9UQP3 | Cardiometabolic II | 0.88(0.65,1.20) | 4.22E-01 | 0.676 |
| HCG22 | E2RYF7 | Cardiometabolic II | 0.89(0.67,1.18) | 4.23E-01 | 0.677 |
| DDC | P20711 | Cardiometabolic | 0.91(0.71,1.15) | 4.28E-01 | 0.683 |
| DUOX2 | Q9NRD8 | Cardiometabolic | 0.93(0.79,1.11) | 4.32E-01 | 0.687 |
| UHRF2 | Q96PU4 | Cardiometabolic II | 0.85(0.56,1.28) | 4.32E-01 | 0.687 |
| ATP5F1D | P30049 | Cardiometabolic II | 0.89(0.67,1.19) | 4.33E-01 | 0.687 |
| ICAM5 | Q9UMF0 | Cardiometabolic | 1.10(0.86,1.41) | 4.35E-01 | 0.688 |
| LILRA3 | Q8N6C8 | Cardiometabolic II | 1.04(0.94,1.16) | 4.36E-01 | 0.688 |
| ZCCHC8 | Q6NZY4 | Cardiometabolic II | 0.91(0.72,1.16) | 4.38E-01 | 0.690 |
| LILRB1 | Q8NHL6 | Cardiometabolic | 1.17(0.78,1.75) | 4.40E-01 | 0.690 |
| SCRG1 | O75711 | Cardiometabolic II | 1.20(0.76,1.91) | 4.40E-01 | 0.690 |
| EIF4EBP1 | Q13541 | Cardiometabolic | 0.93(0.78,1.11) | 4.43E-01 | 0.693 |
| CD209 | Q9NNX6 | Cardiometabolic | 0.89(0.67,1.19) | 4.45E-01 | 0.694 |
| TCL1B | O95988 | Cardiometabolic | 0.94(0.80,1.10) | 4.46E-01 | 0.694 |
| CEP112 | Q8N8E3 | Cardiometabolic II | 0.88(0.62,1.23) | 4.46E-01 | 0.694 |
| CTSH | P09668 | Cardiometabolic | 1.06(0.91,1.24) | 4.51E-01 | 0.700 |
| EXTL1 | Q92935 | Cardiometabolic II | 0.89(0.66,1.21) | 4.53E-01 | 0.702 |
| UMOD | P07911 | Cardiometabolic | 0.93(0.76,1.13) | 4.56E-01 | 0.705 |
| CILP | O75339 | Cardiometabolic II | 0.91(0.72,1.16) | 4.57E-01 | 0.705 |
| VIM | P08670 | Cardiometabolic | 0.96(0.87,1.06) | 4.65E-01 | 0.714 |
| UBQLN3 | Q9H347 | Cardiometabolic II | 1.09(0.87,1.36) | 4.65E-01 | 0.714 |
| HSPG2 | P98160 | Cardiometabolic | 1.16(0.77,1.75) | 4.66E-01 | 0.714 |
| AIF1L | Q9BQI0 | Cardiometabolic II | 0.90(0.69,1.19) | 4.68E-01 | 0.716 |
| BPIFB1 | Q8TDL5 | Cardiometabolic | 1.07(0.89,1.27) | 4.73E-01 | 0.720 |
| ENG | P17813 | Cardiometabolic | 1.27(0.66,2.44) | 4.73E-01 | 0.720 |
| FABP4 | P15090 | Cardiometabolic | 0.93(0.78,1.13) | 4.77E-01 | 0.723 |
| FDX1 | P10109 | Cardiometabolic II | 1.08(0.88,1.32) | 4.77E-01 | 0.723 |
| GP1BA | P07359 | Cardiometabolic | 0.88(0.62,1.25) | 4.84E-01 | 0.730 |
| ITGB1BP2 | Q9UKP3 | Cardiometabolic | 0.96(0.87,1.07) | 4.84E-01 | 0.730 |
| CD80 | P33681 | Cardiometabolic II | 1.14(0.78,1.67) | 4.85E-01 | 0.730 |
| ECI2 | O75521 | Cardiometabolic II | 1.08(0.87,1.33) | 4.87E-01 | 0.730 |
| OXCT1 | P55809 | Cardiometabolic II | 0.93(0.76,1.14) | 4.87E-01 | 0.730 |
| SKIV2L | Q15477 | Cardiometabolic II | 1.12(0.81,1.54) | 4.87E-01 | 0.730 |
| REXO2 | Q9Y3B8 | Cardiometabolic II | 0.90(0.67,1.21) | 4.88E-01 | 0.730 |
| TRAF3IP2 | O43734 | Cardiometabolic II | 1.10(0.84,1.43) | 4.89E-01 | 0.730 |
| DNAJB8 | Q8NHS0 | Cardiometabolic | 0.93(0.74,1.15) | 4.92E-01 | 0.731 |
| HSBP1 | O75506 | Cardiometabolic II | 1.05(0.91,1.21) | 4.92E-01 | 0.731 |
| LEPR | P48357 | Cardiometabolic | 0.88(0.62,1.26) | 4.94E-01 | 0.732 |
| SHD | Q96IW2 | Cardiometabolic II | 0.94(0.79,1.12) | 4.98E-01 | 0.737 |
| PLA2G2A | P14555 | Cardiometabolic | 1.07(0.88,1.30) | 4.99E-01 | 0.737 |
| LILRB5 | O75023 | Cardiometabolic | 0.95(0.81,1.11) | 5.01E-01 | 0.738 |
| ASPN | Q9BXN1 | Cardiometabolic II | 0.84(0.51,1.38) | 5.02E-01 | 0.738 |
| TGM2 | P21980 | Cardiometabolic | 0.95(0.82,1.10) | 5.10E-01 | 0.747 |
| CD93 | Q9NPY3 | Cardiometabolic | 1.13(0.78,1.64) | 5.12E-01 | 0.747 |
| SIRPA | P78324 | Cardiometabolic | 1.07(0.88,1.31) | 5.12E-01 | 0.747 |
| PSAP | P07602 | Cardiometabolic II | 0.85(0.53,1.38) | 5.12E-01 | 0.747 |
| CNPY2 | Q9Y2B0 | Cardiometabolic | 0.94(0.77,1.14) | 5.16E-01 | 0.751 |
| HSD17B3 | P37058 | Cardiometabolic II | 0.91(0.68,1.21) | 5.18E-01 | 0.751 |
| BNIP2 | Q12982 | Cardiometabolic II | 0.88(0.59,1.30) | 5.19E-01 | 0.751 |
| ENPP6 | Q6UWR7 | Cardiometabolic II | 0.89(0.63,1.26) | 5.19E-01 | 0.751 |
| ATP1B2 | P14415 | Cardiometabolic II | 0.87(0.56,1.34) | 5.22E-01 | 0.754 |
| GUK1 | Q16774 | Cardiometabolic II | 0.88(0.60,1.30) | 5.23E-01 | 0.754 |
| HYOU1 | Q9Y4L1 | Cardiometabolic | 1.18(0.71,1.94) | 5.25E-01 | 0.755 |
| MTPN | P58546 | Cardiometabolic | 0.89(0.61,1.29) | 5.28E-01 | 0.755 |
| BOLA2_BOLA2B | Q9H3K6 | Cardiometabolic II | 0.92(0.72,1.18) | 5.28E-01 | 0.755 |
| COL1A1 | P02452 | Cardiometabolic | 1.16(0.74,1.81) | 5.29E-01 | 0.755 |
| CST6 | Q15828 | Cardiometabolic | 1.08(0.85,1.37) | 5.30E-01 | 0.755 |
| VWF | P04275 | Cardiometabolic | 1.05(0.90,1.23) | 5.31E-01 | 0.755 |
| ENTR1 | Q96C92 | Cardiometabolic II | 1.08(0.86,1.35) | 5.31E-01 | 0.755 |
| HEPH | Q9BQS7 | Cardiometabolic II | 0.85(0.52,1.41) | 5.33E-01 | 0.755 |
| SCGB2A2 | Q13296 | Cardiometabolic II | 0.86(0.54,1.38) | 5.33E-01 | 0.755 |
| RNASE10 | Q5GAN6 | Cardiometabolic II | 1.09(0.83,1.44) | 5.37E-01 | 0.759 |
| SERPINB5 | P36952 | Cardiometabolic | 0.93(0.74,1.17) | 5.38E-01 | 0.759 |
| ENOX2 | Q16206 | Cardiometabolic II | 1.16(0.73,1.83) | 5.40E-01 | 0.760 |
| NPR1 | P16066 | Cardiometabolic II | 0.90(0.63,1.28) | 5.42E-01 | 0.760 |
| CA1 | P00915 | Cardiometabolic | 1.06(0.88,1.28) | 5.44E-01 | 0.760 |
| SEMA3F | Q13275 | Cardiometabolic | 1.13(0.76,1.70) | 5.44E-01 | 0.760 |
| MTR | Q99707 | Cardiometabolic II | 0.93(0.75,1.16) | 5.44E-01 | 0.760 |
| ENPP2 | Q13822 | Cardiometabolic | 0.88(0.58,1.33) | 5.45E-01 | 0.760 |
| MFAP3 | P55082 | Cardiometabolic | 0.93(0.72,1.19) | 5.47E-01 | 0.760 |
| PGD | P52209 | Cardiometabolic II | 0.93(0.72,1.19) | 5.47E-01 | 0.760 |
| LRCH4 | O75427 | Cardiometabolic II | 0.94(0.77,1.15) | 5.48E-01 | 0.760 |
| DEFA1_DEFA1B | P59665 | Cardiometabolic | 1.06(0.86,1.31) | 5.58E-01 | 0.772 |
| NFYA | P23511 | Cardiometabolic II | 0.89(0.61,1.30) | 5.59E-01 | 0.772 |
| IRAG2 | Q12912 | Cardiometabolic | 1.04(0.92,1.18) | 5.60E-01 | 0.772 |
| CEACAM6 | P40199 | Cardiometabolic II | 1.10(0.79,1.53) | 5.61E-01 | 0.772 |
| MARCO | Q9UEW3 | Cardiometabolic | 0.88(0.57,1.35) | 5.63E-01 | 0.772 |
| CALCOCO2 | Q13137 | Cardiometabolic II | 0.95(0.80,1.13) | 5.63E-01 | 0.772 |
| AKR1C4 | P17516 | Cardiometabolic | 0.94(0.75,1.17) | 5.65E-01 | 0.772 |
| SOD1 | P00441 | Cardiometabolic | 0.93(0.74,1.18) | 5.65E-01 | 0.772 |
| HYAL1 | Q12794 | Cardiometabolic | 0.86(0.52,1.43) | 5.69E-01 | 0.776 |
| NUP50 | Q9UKX7 | Cardiometabolic II | 0.88(0.56,1.38) | 5.71E-01 | 0.777 |
| MELTF | P08582 | Cardiometabolic II | 0.88(0.57,1.37) | 5.73E-01 | 0.779 |
| IGF2BP3 | O00425 | Cardiometabolic II | 0.91(0.67,1.25) | 5.77E-01 | 0.783 |
| CPQ | Q9Y646 | Cardiometabolic II | 0.90(0.62,1.31) | 5.79E-01 | 0.783 |
| VASN | Q6EMK4 | Cardiometabolic | 0.89(0.58,1.36) | 5.81E-01 | 0.783 |
| PRKAR1A | P10644 | Cardiometabolic | 0.96(0.84,1.10) | 5.82E-01 | 0.783 |
| CELA3A | P09093 | Cardiometabolic | 1.05(0.87,1.28) | 5.84E-01 | 0.783 |
| CYP24A1 | Q07973 | Cardiometabolic II | 0.95(0.81,1.13) | 5.84E-01 | 0.783 |
| PGLYRP1 | O75594 | Cardiometabolic | 1.08(0.83,1.40) | 5.86E-01 | 0.783 |
| COL2A1 | P02458 | Cardiometabolic II | 1.05(0.89,1.24) | 5.86E-01 | 0.783 |
| GSTM4 | Q03013 | Cardiometabolic II | 1.05(0.88,1.25) | 5.87E-01 | 0.783 |
| LILRA4 | P59901 | Cardiometabolic II | 0.92(0.70,1.23) | 5.87E-01 | 0.783 |
| KIT | P10721 | Cardiometabolic | 0.90(0.62,1.31) | 5.89E-01 | 0.784 |
| SIL1 | Q9H173 | Cardiometabolic II | 0.88(0.55,1.40) | 5.93E-01 | 0.788 |
| AXL | P30530 | Cardiometabolic | 1.12(0.74,1.69) | 5.95E-01 | 0.788 |
| GGH | Q92820 | Cardiometabolic | 0.92(0.67,1.26) | 5.96E-01 | 0.788 |
| GRK5 | P34947 | Cardiometabolic | 0.94(0.74,1.19) | 5.96E-01 | 0.788 |
| PAGR1 | Q9BTK6 | Cardiometabolic II | 0.92(0.67,1.26) | 5.97E-01 | 0.788 |
| AAMDC | Q9H7C9 | Cardiometabolic II | 0.93(0.72,1.21) | 6.04E-01 | 0.796 |
| PYDC1 | Q8WXC3 | Cardiometabolic II | 1.09(0.79,1.48) | 6.07E-01 | 0.798 |
| ART3 | Q13508 | Cardiometabolic | 1.09(0.79,1.50) | 6.08E-01 | 0.798 |
| SOST | Q9BQB4 | Cardiometabolic | 0.93(0.69,1.24) | 6.09E-01 | 0.798 |
| MYH4 | Q9Y623 | Cardiometabolic II | 0.92(0.67,1.27) | 6.10E-01 | 0.798 |
| RCOR1 | Q9UKL0 | Cardiometabolic | 0.93(0.69,1.24) | 6.12E-01 | 0.799 |
| LIPF | P07098 | Cardiometabolic II | 0.95(0.76,1.18) | 6.14E-01 | 0.800 |
| KIF22 | Q14807 | Cardiometabolic II | 1.03(0.92,1.15) | 6.15E-01 | 0.800 |
| ELN | P15502 | Cardiometabolic II | 1.09(0.77,1.55) | 6.20E-01 | 0.805 |
| CDA | P32320 | Cardiometabolic II | 0.93(0.71,1.23) | 6.24E-01 | 0.809 |
| VIPR1 | P32241 | Cardiometabolic II | 1.14(0.67,1.95) | 6.31E-01 | 0.816 |
| DHODH | Q02127 | Cardiometabolic II | 0.93(0.70,1.24) | 6.34E-01 | 0.819 |
| WASF1 | Q92558 | Cardiometabolic | 0.97(0.86,1.10) | 6.37E-01 | 0.821 |
| CLUL1 | Q15846 | Cardiometabolic | 0.94(0.73,1.21) | 6.44E-01 | 0.829 |
| CXCL5 | P42830 | Cardiometabolic | 0.98(0.89,1.08) | 6.45E-01 | 0.829 |
| CSTB | P04080 | Cardiometabolic | 1.06(0.82,1.39) | 6.50E-01 | 0.834 |
| DMD | P11532 | Cardiometabolic II | 1.06(0.82,1.37) | 6.55E-01 | 0.838 |
| ATP1B1 | P05026 | Cardiometabolic II | 0.93(0.69,1.26) | 6.56E-01 | 0.838 |
| RAB39B | Q96DA2 | Cardiometabolic II | 0.96(0.82,1.13) | 6.57E-01 | 0.838 |
| TRPV3 | Q8NET8 | Cardiometabolic II | 0.94(0.71,1.24) | 6.70E-01 | 0.853 |
| HMOX1 | P09601 | Cardiometabolic | 1.05(0.83,1.34) | 6.76E-01 | 0.859 |
| EPHX2 | P34913 | Cardiometabolic | 1.04(0.87,1.24) | 6.81E-01 | 0.864 |
| GHRL | Q9UBU3 | Cardiometabolic | 0.97(0.85,1.11) | 6.82E-01 | 0.864 |
| GNPDA1 | P46926 | Cardiometabolic II | 1.07(0.76,1.52) | 6.84E-01 | 0.865 |
| DDA1 | Q9BW61 | Cardiometabolic II | 0.93(0.66,1.32) | 6.86E-01 | 0.865 |
| LILRA6 | Q6PI73 | Cardiometabolic II | 0.96(0.81,1.15) | 6.86E-01 | 0.865 |
| HBZ | P02008 | Cardiometabolic II | 1.03(0.91,1.16) | 6.88E-01 | 0.866 |
| PKD1 | P98161 | Cardiometabolic II | 0.90(0.55,1.49) | 6.91E-01 | 0.868 |
| CELA2A | P08217 | Cardiometabolic II | 1.04(0.84,1.29) | 6.93E-01 | 0.868 |
| AMY2B | P19961 | Cardiometabolic | 0.95(0.75,1.21) | 6.94E-01 | 0.868 |
| LGALS1 | P09382 | Cardiometabolic | 0.94(0.68,1.29) | 6.95E-01 | 0.868 |
| UGDH | O60701 | Cardiometabolic II | 0.97(0.85,1.12) | 7.00E-01 | 0.873 |
| ST6GAL1 | P15907 | Cardiometabolic | 0.93(0.63,1.36) | 7.06E-01 | 0.878 |
| CASQ2 | O14958 | Cardiometabolic II | 0.95(0.71,1.27) | 7.08E-01 | 0.878 |
| SLURP1 | P55000 | Cardiometabolic II | 0.93(0.65,1.34) | 7.08E-01 | 0.878 |
| TMEM132A | Q24JP5 | Cardiometabolic II | 1.04(0.84,1.29) | 7.09E-01 | 0.878 |
| PTPRS | Q13332 | Cardiometabolic | 1.09(0.69,1.71) | 7.14E-01 | 0.883 |
| NFX1 | Q12986 | Cardiometabolic II | 0.97(0.79,1.17) | 7.21E-01 | 0.890 |
| CDH6 | P55285 | Cardiometabolic | 1.05(0.78,1.43) | 7.28E-01 | 0.897 |
| ICAM3 | P32942 | Cardiometabolic | 1.08(0.71,1.64) | 7.32E-01 | 0.900 |
| CD163 | Q86VB7 | Cardiometabolic | 1.05(0.81,1.35) | 7.33E-01 | 0.900 |
| LCN2 | P80188 | Cardiometabolic | 1.05(0.78,1.42) | 7.34E-01 | 0.900 |
| CPA1 | P15085 | Cardiometabolic | 0.97(0.81,1.16) | 7.38E-01 | 0.903 |
| ESAM | Q96AP7 | Cardiometabolic | 0.94(0.66,1.35) | 7.40E-01 | 0.903 |
| PLIN3 | O60664 | Cardiometabolic | 0.96(0.73,1.25) | 7.40E-01 | 0.903 |
| PAG1 | Q9NWQ8 | Cardiometabolic | 0.97(0.78,1.19) | 7.43E-01 | 0.903 |
| ANK2 | Q01484 | Cardiometabolic II | 0.97(0.80,1.17) | 7.43E-01 | 0.903 |
| EDEM2 | Q9BV94 | Cardiometabolic II | 1.06(0.73,1.54) | 7.44E-01 | 0.903 |
| KRT6C | P48668 | Cardiometabolic II | 1.06(0.74,1.52) | 7.46E-01 | 0.904 |
| PM20D1 | Q6GTS8 | Cardiometabolic | 1.01(0.95,1.08) | 7.49E-01 | 0.906 |
| TNF | P01375 | Cardiometabolic | 0.95(0.70,1.30) | 7.57E-01 | 0.914 |
| SEMA7A | O75326 | Cardiometabolic | 1.06(0.72,1.57) | 7.58E-01 | 0.914 |
| COMP | P49747 | Cardiometabolic | 0.95(0.67,1.33) | 7.59E-01 | 0.914 |
| SAT1 | P21673 | Cardiometabolic II | 1.04(0.80,1.35) | 7.60E-01 | 0.914 |
| TIMP1 | P01033 | Cardiometabolic | 1.06(0.71,1.59) | 7.63E-01 | 0.916 |
| DDT | P30046 | Cardiometabolic II | 1.05(0.77,1.42) | 7.69E-01 | 0.921 |
| SPESP1 | Q6UW49 | Cardiometabolic II | 0.94(0.62,1.42) | 7.70E-01 | 0.921 |
| SIGLEC8 | Q9NYZ4 | Cardiometabolic II | 0.96(0.71,1.28) | 7.73E-01 | 0.921 |
| CGREF1 | Q99674 | Cardiometabolic | 1.04(0.80,1.35) | 7.74E-01 | 0.921 |
| CCL14 | Q16627 | Cardiometabolic | 0.96(0.72,1.28) | 7.75E-01 | 0.921 |
| ELOB | Q15370 | Cardiometabolic II | 1.05(0.75,1.48) | 7.75E-01 | 0.921 |
| TGFBI | Q15582 | Cardiometabolic | 0.94(0.61,1.44) | 7.77E-01 | 0.922 |
| CHIT1 | Q13231 | Cardiometabolic | 0.99(0.92,1.06) | 7.80E-01 | 0.922 |
| GH1 | P01241 | Cardiometabolic | 1.01(0.95,1.08) | 7.80E-01 | 0.922 |
| CCL18 | P55774 | Cardiometabolic | 1.02(0.87,1.21) | 7.81E-01 | 0.922 |
| PRAP1 | Q96NZ9 | Cardiometabolic II | 0.96(0.72,1.29) | 7.84E-01 | 0.924 |
| PMCH | P20382 | Cardiometabolic II | 0.98(0.83,1.15) | 7.88E-01 | 0.927 |
| NOTCH2 | Q04721 | Cardiometabolic II | 1.09(0.58,2.04) | 7.89E-01 | 0.927 |
| CACNA1H | O95180 | Cardiometabolic II | 0.97(0.75,1.24) | 7.91E-01 | 0.928 |
| NPC2 | P61916 | Cardiometabolic II | 1.05(0.72,1.55) | 7.92E-01 | 0.928 |
| FDX2 | Q6P4F2 | Cardiometabolic II | 1.03(0.81,1.32) | 7.94E-01 | 0.929 |
| CD55 | P08174 | Cardiometabolic | 1.06(0.68,1.64) | 7.99E-01 | 0.930 |
| MEP1B | Q16820 | Cardiometabolic | 0.99(0.90,1.09) | 8.00E-01 | 0.930 |
| MANSC4 | A6NHS7 | Cardiometabolic II | 0.98(0.80,1.18) | 8.00E-01 | 0.930 |
| APLP1 | P51693 | Cardiometabolic | 1.03(0.83,1.28) | 8.01E-01 | 0.930 |
| TTN | Q8WZ42 | Cardiometabolic II | 0.97(0.79,1.20) | 8.02E-01 | 0.930 |
| SELE | P16581 | Cardiometabolic | 1.03(0.83,1.28) | 8.03E-01 | 0.930 |
| EIF2AK3 | Q9NZJ5 | Cardiometabolic II | 0.97(0.80,1.19) | 8.05E-01 | 0.931 |
| CXCL16 | Q9H2A7 | Cardiometabolic | 1.06(0.66,1.70) | 8.08E-01 | 0.932 |
| RBM19 | Q9Y4C8 | Cardiometabolic II | 1.04(0.78,1.37) | 8.08E-01 | 0.932 |
| USP47 | Q96K76 | Cardiometabolic II | 0.98(0.83,1.16) | 8.13E-01 | 0.934 |
| PCSK9 | Q8NBP7 | Cardiometabolic | 1.03(0.78,1.37) | 8.14E-01 | 0.934 |
| COL3A1 | P02461 | Cardiometabolic II | 1.07(0.61,1.89) | 8.14E-01 | 0.934 |
| GRP | P07492 | Cardiometabolic II | 0.97(0.76,1.24) | 8.15E-01 | 0.934 |
| LAMB1 | P07942 | Cardiometabolic II | 1.05(0.67,1.66) | 8.21E-01 | 0.937 |
| MFAP5 | Q13361 | Cardiometabolic | 1.03(0.79,1.34) | 8.22E-01 | 0.937 |
| LAMA1 | P25391 | Cardiometabolic II | 0.97(0.75,1.25) | 8.22E-01 | 0.937 |
| BOC | Q9BWV1 | Cardiometabolic | 1.06(0.64,1.74) | 8.25E-01 | 0.939 |
| CD248 | Q9HCU0 | Cardiometabolic II | 1.04(0.75,1.43) | 8.27E-01 | 0.939 |
| ANG | P03950 | Cardiometabolic | 1.04(0.74,1.46) | 8.28E-01 | 0.939 |
| BLMH | Q13867 | Cardiometabolic | 0.96(0.67,1.37) | 8.31E-01 | 0.939 |
| BCAT1 | P54687 | Cardiometabolic II | 0.95(0.62,1.47) | 8.31E-01 | 0.939 |
| ENO3 | P13929 | Cardiometabolic II | 1.02(0.82,1.28) | 8.34E-01 | 0.939 |
| AZU1 | P20160 | Cardiometabolic | 1.02(0.87,1.18) | 8.35E-01 | 0.939 |
| TNFSF13B | Q9Y275 | Cardiometabolic | 1.04(0.70,1.56) | 8.35E-01 | 0.939 |
| SCN4B | Q8IWT1 | Cardiometabolic II | 1.03(0.76,1.41) | 8.35E-01 | 0.939 |
| IGDCC4 | Q8TDY8 | Cardiometabolic II | 1.05(0.64,1.73) | 8.38E-01 | 0.941 |
| SIGLEC7 | Q9Y286 | Cardiometabolic | 0.96(0.64,1.43) | 8.43E-01 | 0.945 |
| THRAP3 | Q9Y2W1 | Cardiometabolic II | 1.01(0.90,1.14) | 8.54E-01 | 0.956 |
| S100A11 | P31949 | Cardiometabolic | 1.02(0.80,1.30) | 8.55E-01 | 0.956 |
| ENTPD6 | O75354 | Cardiometabolic | 1.04(0.68,1.59) | 8.57E-01 | 0.956 |
| CD46 | P15529 | Cardiometabolic | 0.97(0.64,1.45) | 8.64E-01 | 0.960 |
| RTKN2 | Q8IZC4 | Cardiometabolic II | 1.02(0.79,1.32) | 8.64E-01 | 0.960 |
| ISM2 | Q6H9L7 | Cardiometabolic II | 1.03(0.76,1.39) | 8.65E-01 | 0.960 |
| EDIL3 | O43854 | Cardiometabolic | 1.03(0.74,1.43) | 8.67E-01 | 0.960 |
| GM2A | P17900 | Cardiometabolic II | 1.03(0.75,1.42) | 8.68E-01 | 0.960 |
| CD14 | P08571 | Cardiometabolic | 1.02(0.77,1.36) | 8.71E-01 | 0.960 |
| HADH | Q16836 | Cardiometabolic II | 0.98(0.81,1.20) | 8.71E-01 | 0.960 |
| CYTL1 | Q9NRR1 | Cardiometabolic II | 1.04(0.62,1.77) | 8.73E-01 | 0.960 |
| PLXNB2 | O15031 | Cardiometabolic | 0.96(0.60,1.55) | 8.74E-01 | 0.960 |
| MYL4 | P12829 | Cardiometabolic II | 1.02(0.79,1.31) | 8.77E-01 | 0.960 |
| NEB | P20929 | Cardiometabolic II | 1.03(0.73,1.45) | 8.77E-01 | 0.960 |
| DCTPP1 | Q9H773 | Cardiometabolic | 1.03(0.71,1.48) | 8.78E-01 | 0.960 |
| EPPK1 | P58107 | Cardiometabolic II | 0.99(0.81,1.19) | 8.78E-01 | 0.960 |
| THBS4 | P35443 | Cardiometabolic | 0.98(0.77,1.25) | 8.79E-01 | 0.960 |
| MUC2 | Q02817 | Cardiometabolic II | 0.99(0.86,1.14) | 8.84E-01 | 0.963 |
| POMC | P01189 | Cardiometabolic II | 1.01(0.84,1.23) | 8.84E-01 | 0.963 |
| TYRO3 | Q06418 | Cardiometabolic | 0.97(0.65,1.45) | 8.88E-01 | 0.963 |
| SEL1L | Q9UBV2 | Cardiometabolic II | 1.03(0.68,1.57) | 8.89E-01 | 0.963 |
| BMP6 | P22004 | Cardiometabolic | 1.02(0.76,1.37) | 8.91E-01 | 0.963 |
| SCARF1 | Q14162 | Cardiometabolic | 0.98(0.75,1.28) | 8.92E-01 | 0.963 |
| ABCA2 | Q9BZC7 | Cardiometabolic II | 1.03(0.70,1.50) | 8.92E-01 | 0.963 |
| RECK | O95980 | Cardiometabolic II | 1.05(0.55,2.01) | 8.92E-01 | 0.963 |
| NUDT10 | Q8NFP7 | Cardiometabolic II | 1.03(0.63,1.70) | 8.96E-01 | 0.966 |
| APOBR | Q0VD83 | Cardiometabolic II | 1.02(0.74,1.41) | 8.99E-01 | 0.966 |
| CORO1A | P31146 | Cardiometabolic | 1.01(0.89,1.14) | 9.00E-01 | 0.966 |
| COL15A1 | P39059 | Cardiometabolic II | 1.03(0.65,1.63) | 9.00E-01 | 0.966 |
| CCL16 | O15467 | Cardiometabolic | 0.99(0.80,1.22) | 9.01E-01 | 0.966 |
| DLL4 | Q9NR61 | Cardiometabolic II | 0.99(0.85,1.16) | 9.06E-01 | 0.969 |
| CDH5 | P33151 | Cardiometabolic | 0.98(0.67,1.43) | 9.08E-01 | 0.969 |
| RANBP1 | P43487 | Cardiometabolic II | 0.99(0.76,1.27) | 9.08E-01 | 0.969 |
| LECT2 | O14960 | Cardiometabolic II | 1.01(0.84,1.22) | 9.09E-01 | 0.969 |
| GLO1 | Q04760 | Cardiometabolic | 0.99(0.81,1.20) | 9.12E-01 | 0.970 |
| NOTCH1 | P46531 | Cardiometabolic | 1.04(0.51,2.12) | 9.13E-01 | 0.970 |
| SSC5D | A1L4H1 | Cardiometabolic | 1.01(0.78,1.32) | 9.17E-01 | 0.973 |
| ARNTL | O00327 | Cardiometabolic II | 0.99(0.76,1.28) | 9.19E-01 | 0.974 |
| AK1 | P00568 | Cardiometabolic | 1.01(0.85,1.20) | 9.32E-01 | 0.981 |
| CTSB | P07858 | Cardiometabolic | 0.99(0.81,1.22) | 9.32E-01 | 0.981 |
| PRSS2 | P07478 | Cardiometabolic | 1.01(0.82,1.25) | 9.33E-01 | 0.981 |
| LCP1 | P13796 | Cardiometabolic II | 1.01(0.77,1.33) | 9.34E-01 | 0.981 |
| TEF | Q10587 | Cardiometabolic II | 0.99(0.72,1.36) | 9.34E-01 | 0.981 |
| PDCD6 | O75340 | Cardiometabolic | 1.01(0.86,1.19) | 9.36E-01 | 0.981 |
| AHNAK2 | Q8IVF2 | Cardiometabolic II | 0.98(0.63,1.54) | 9.36E-01 | 0.981 |
| TPM3 | P06753 | Cardiometabolic II | 1.01(0.75,1.37) | 9.39E-01 | 0.981 |
| IGSF8 | Q969P0 | Cardiometabolic | 0.99(0.67,1.44) | 9.40E-01 | 0.981 |
| LILRB2 | Q8N423 | Cardiometabolic | 1.01(0.73,1.40) | 9.40E-01 | 0.981 |
| ANGPTL1 | O95841 | Cardiometabolic | 0.99(0.66,1.47) | 9.42E-01 | 0.981 |
| M6PR | P20645 | Cardiometabolic II | 0.99(0.70,1.40) | 9.42E-01 | 0.981 |
| CBLIF | P27352 | Cardiometabolic | 1.01(0.87,1.16) | 9.43E-01 | 0.981 |
| KIF1C | O43896 | Cardiometabolic II | 0.99(0.66,1.48) | 9.46E-01 | 0.982 |
| C1QTNF1 | Q9BXJ1 | Cardiometabolic | 0.99(0.76,1.30) | 9.47E-01 | 0.982 |
| GOT1 | P17174 | Cardiometabolic II | 0.99(0.72,1.37) | 9.52E-01 | 0.986 |
| PLTP | P55058 | Cardiometabolic | 1.01(0.74,1.38) | 9.55E-01 | 0.987 |
| AOC3 | Q16853 | Cardiometabolic | 0.99(0.67,1.45) | 9.56E-01 | 0.987 |
| PRG3 | Q9Y2Y8 | Cardiometabolic II | 0.99(0.77,1.29) | 9.57E-01 | 0.987 |
| CTSL | P07711 | Cardiometabolic | 1.01(0.63,1.61) | 9.59E-01 | 0.987 |
| PTPRC | P08575 | Cardiometabolic II | 0.99(0.56,1.74) | 9.62E-01 | 0.989 |
| FSHB | P01225 | Cardiometabolic II | 1.00(0.82,1.21) | 9.67E-01 | 0.993 |
| ACE2 | Q9BYF1 | Cardiometabolic | 1.00(0.81,1.25) | 9.70E-01 | 0.994 |
| ADAMTS13 | Q76LX8 | Cardiometabolic | 0.99(0.62,1.59) | 9.71E-01 | 0.994 |
| CDH1 | P12830 | Cardiometabolic | 0.99(0.73,1.36) | 9.73E-01 | 0.995 |
| ITGB2 | P05107 | Cardiometabolic | 0.99(0.66,1.49) | 9.76E-01 | 0.996 |
| PTPRZ1 | P23471 | Cardiometabolic II | 1.01(0.67,1.51) | 9.78E-01 | 0.997 |
| MNDA | P41218 | Cardiometabolic | 1.00(0.89,1.12) | 9.82E-01 | 0.998 |
| ANP32C | O43423 | Cardiometabolic II | 1.00(0.71,1.40) | 9.82E-01 | 0.998 |
| GLRX | P35754 | Cardiometabolic | 1.00(0.82,1.22) | 9.84E-01 | 0.999 |
| TIE1 | P35590 | Cardiometabolic | 1.00(0.76,1.32) | 9.85E-01 | 0.999 |
| AMY2A | P04746 | Cardiometabolic | 1.00(0.79,1.27) | 9.87E-01 | 0.999 |
| NGFR | P08138 | Cardiometabolic II | 1.00(0.63,1.58) | 9.88E-01 | 0.999 |
| DMP1 | Q13316 | Cardiometabolic II | 1.00(0.80,1.24) | 9.89E-01 | 0.999 |
| ANXA2 | P07355 | Cardiometabolic II | 1.00(0.83,1.21) | 9.92E-01 | 0.999 |
| MMP15 | P51511 | Cardiometabolic II | 1.00(0.76,1.32) | 9.94E-01 | 0.999 |
| NTRK2 | Q16620 | Cardiometabolic | 1.00(0.64,1.55) | 9.95E-01 | 0.999 |
| CPB1 | P15086 | Cardiometabolic | 1.00(0.83,1.21) | 9.96E-01 | 0.999 |
| RNASE3 | P12724 | Cardiometabolic | 1.00(0.90,1.11) | 9.96E-01 | 0.999 |
| CPTP | Q5TA50 | Cardiometabolic II | 1.00(0.70,1.42) | 9.98E-01 | 0.999 |
| B3GNT7 | Q8NFL0 | Cardiometabolic II | 1.00(0.69,1.44) | 9.99E-01 | 0.999 |

**^*^**Cox regression model adjusted for age and sex. *P* value was calculated under two-sided tests, and statistical significance was defined as a false discovery rate (FDR)-corrected *P* value <0.05, adjusted for the number of proteins tested (n=731).

**Table S7. Cox regression analysis for the associations between 724 proteins from the inflammation panel and the risk of new-onset Alzheimer’s disease in the training set. ^*^**

| **Target Name** | **UniProt** | **Panel** | **HR (95%CI)** | ***P* value** | **FDR-corrected *P* value** |
| --- | --- | --- | --- | --- | --- |
| APOE | P02649 | Inflammation II | 0.29(0.23,0.37) | 1.02E-22 | <0.001 |
| MENT | Q9BUN1 | Inflammation II | 8.04(4.76,13.60) | 7.37E-15 | <0.001 |
| TGFBR1 | P36897 | Inflammation II | 2.00(1.45,2.76) | 2.74E-05 | 0.005 |
| KLKB1 | P03952 | Inflammation II | 0.34(0.20,0.56) | 3.01E-05 | 0.005 |
| LCAT | P04180 | Inflammation II | 0.51(0.36,0.71) | 7.57E-05 | 0.011 |
| ERBB3 | P21860 | Inflammation | 0.32(0.18,0.59) | 2.10E-04 | 0.025 |
| TNFRSF11B | O00300 | Inflammation | 1.96(1.35,2.85) | 3.98E-04 | 0.041 |
| TTR | P02766 | Inflammation II | 0.40(0.24,0.67) | 5.07E-04 | 0.046 |
| WNT9A | O14904 | Inflammation | 1.87(1.30,2.70) | 7.47E-04 | 0.051 |
| SERPINA5 | P05154 | Inflammation II | 0.45(0.28,0.71) | 7.63E-04 | 0.051 |
| PGA4 | P0DJD7 | Inflammation II | 1.34(1.13,1.58) | 7.74E-04 | 0.051 |
| CD276 | Q5ZPR3 | Inflammation | 1.73(1.25,2.38) | 8.58E-04 | 0.052 |
| LRRN1 | Q6UXK5 | Inflammation | 0.63(0.47,0.84) | 1.56E-03 | 0.082 |
| APOA2 | P02652 | Inflammation II | 1.51(1.17,1.95) | 1.66E-03 | 0.082 |
| ESM1 | Q9NQ30 | Inflammation | 1.67(1.21,2.30) | 1.69E-03 | 0.082 |
| FGF12 | P61328 | Inflammation II | 1.30(1.10,1.53) | 1.86E-03 | 0.084 |
| PRELP | P51888 | Inflammation | 2.14(1.30,3.51) | 2.76E-03 | 0.111 |
| C1QA | P02745 | Inflammation | 2.08(1.29,3.37) | 2.77E-03 | 0.111 |
| IL22RA1 | Q8N6P7 | Inflammation | 0.62(0.45,0.85) | 3.08E-03 | 0.117 |
| PEPD | P12955 | Inflammation II | 0.48(0.30,0.79) | 3.25E-03 | 0.118 |
| LGALS4 | P56470 | Inflammation | 1.32(1.10,1.60) | 3.66E-03 | 0.126 |
| TCN1 | P20061 | Inflammation II | 1.49(1.13,1.96) | 4.62E-03 | 0.145 |
| NPHS2 | Q9NP85 | Inflammation II | 1.17(1.05,1.30) | 4.89E-03 | 0.145 |
| CSF1 | P09603 | Inflammation | 1.76(1.18,2.61) | 5.04E-03 | 0.145 |
| GHR | P10912 | Inflammation II | 0.54(0.35,0.83) | 5.17E-03 | 0.145 |
| AHSG | P02765 | Inflammation II | 0.60(0.41,0.86) | 5.23E-03 | 0.145 |
| TNFSF10 | P50591 | Inflammation | 0.53(0.34,0.83) | 5.41E-03 | 0.145 |
| TNFSF11 | O14788 | Inflammation | 0.77(0.64,0.93) | 5.63E-03 | 0.146 |
| TGFA | P01135 | Inflammation | 1.36(1.09,1.71) | 6.75E-03 | 0.166 |
| GLA | P06280 | Inflammation II | 0.63(0.45,0.88) | 6.89E-03 | 0.166 |
| AMOT | Q4VCS5 | Inflammation II | 0.62(0.44,0.88) | 7.68E-03 | 0.179 |
| PTX3 | P26022 | Inflammation | 1.42(1.09,1.84) | 8.50E-03 | 0.192 |
| FN1 | P02751 | Inflammation II | 0.52(0.32,0.85) | 9.45E-03 | 0.207 |
| IL31RA | Q8NI17 | Inflammation II | 0.63(0.44,0.89) | 1.01E-02 | 0.211 |
| CLEC3B | P05452 | Inflammation II | 0.43(0.23,0.82) | 1.02E-02 | 0.211 |
| SPON1 | Q9HCB6 | Inflammation | 1.48(1.09,2.01) | 1.11E-02 | 0.213 |
| GP5 | P40197 | Inflammation II | 0.64(0.45,0.90) | 1.13E-02 | 0.213 |
| APCS | P02743 | Inflammation II | 0.63(0.44,0.90) | 1.14E-02 | 0.213 |
| SERPINF2 | P08697 | Inflammation II | 0.32(0.13,0.77) | 1.15E-02 | 0.213 |
| IL17C | Q9P0M4 | Inflammation | 1.20(1.04,1.38) | 1.19E-02 | 0.215 |
| GZMB | P10144 | Inflammation | 0.78(0.65,0.95) | 1.23E-02 | 0.217 |
| KYNU | Q16719 | Inflammation | 0.72(0.56,0.93) | 1.26E-02 | 0.217 |
| YTHDF3 | Q7Z739 | Inflammation | 0.84(0.73,0.96) | 1.34E-02 | 0.217 |
| SNCA | P37840 | Inflammation II | 0.86(0.77,0.97) | 1.34E-02 | 0.217 |
| LATS1 | O95835 | Inflammation II | 0.82(0.69,0.96) | 1.35E-02 | 0.217 |
| IL32 | P24001 | Inflammation | 1.37(1.06,1.76) | 1.53E-02 | 0.241 |
| CKMT1A_CKMT1B | P12532 | Inflammation | 1.23(1.03,1.47) | 2.00E-02 | 0.308 |
| NUMB | P49757 | Inflammation II | 0.83(0.71,0.97) | 2.07E-02 | 0.312 |
| SMPDL3A | Q92484 | Inflammation | 0.82(0.70,0.97) | 2.12E-02 | 0.313 |
| F12 | P00748 | Inflammation II | 0.71(0.53,0.95) | 2.24E-02 | 0.324 |
| PRR4 | Q16378 | Inflammation II | 1.24(1.03,1.49) | 2.38E-02 | 0.328 |
| HS6ST2 | Q96MM7 | Inflammation II | 1.52(1.06,2.19) | 2.46E-02 | 0.328 |
| MOCS2 | O96007 | Inflammation II | 0.79(0.64,0.97) | 2.49E-02 | 0.328 |
| LY6D | Q14210 | Inflammation | 1.37(1.04,1.81) | 2.60E-02 | 0.328 |
| SCG3 | Q8WXD2 | Inflammation | 1.45(1.04,2.02) | 2.74E-02 | 0.328 |
| SCGB3A1 | Q96QR1 | Inflammation II | 1.74(1.06,2.85) | 2.76E-02 | 0.328 |
| IL10 | P22301 | Inflammation | 1.14(1.01,1.29) | 2.79E-02 | 0.328 |
| BTD | P43251 | Inflammation II | 0.51(0.28,0.93) | 2.79E-02 | 0.328 |
| ANGPTL4 | Q9BY76 | Inflammation | 1.31(1.03,1.68) | 2.85E-02 | 0.328 |
| ADA | P00813 | Inflammation | 0.68(0.49,0.96) | 2.86E-02 | 0.328 |
| SERPINA4 | P29622 | Inflammation II | 0.55(0.32,0.94) | 2.86E-02 | 0.328 |
| FIS1 | Q9Y3D6 | Inflammation | 0.84(0.72,0.98) | 2.94E-02 | 0.328 |
| SUSD5 | O60279 | Inflammation II | 1.52(1.04,2.21) | 2.97E-02 | 0.328 |
| CD79B | P40259 | Inflammation | 1.28(1.02,1.61) | 3.06E-02 | 0.328 |
| TPD52L2 | O43399 | Inflammation II | 0.88(0.78,0.99) | 3.11E-02 | 0.328 |
| SPINK4 | O60575 | Inflammation | 1.20(1.02,1.41) | 3.16E-02 | 0.328 |
| CCL13 | Q99616 | Inflammation | 0.83(0.71,0.98) | 3.20E-02 | 0.328 |
| FSTL3 | O95633 | Inflammation | 1.41(1.03,1.94) | 3.23E-02 | 0.328 |
| LGMN | Q99538 | Inflammation | 0.70(0.51,0.97) | 3.23E-02 | 0.328 |
| GMPR2 | Q9P2T1 | Inflammation II | 0.80(0.66,0.98) | 3.31E-02 | 0.328 |
| ERMAP | Q96PL5 | Inflammation II | 0.72(0.53,0.97) | 3.35E-02 | 0.328 |
| REG4 | Q9BYZ8 | Inflammation | 1.26(1.02,1.56) | 3.36E-02 | 0.328 |
| CFB | P00751 | Inflammation II | 0.66(0.45,0.97) | 3.39E-02 | 0.328 |
| PLG | P00747 | Inflammation II | 0.53(0.29,0.95) | 3.39E-02 | 0.328 |
| C9 | P02748 | Inflammation II | 1.31(1.02,1.67) | 3.42E-02 | 0.328 |
| ADH1B | P00325 | Inflammation II | 0.82(0.68,0.99) | 3.46E-02 | 0.328 |
| VASP | P50552 | Inflammation II | 0.82(0.68,0.99) | 3.49E-02 | 0.328 |
| OSCAR | Q8IYS5 | Inflammation | 1.43(1.02,2.00) | 3.59E-02 | 0.329 |
| PRDX3 | P30048 | Inflammation | 0.84(0.71,0.99) | 3.64E-02 | 0.329 |
| FABP1 | P07148 | Inflammation | 1.13(1.01,1.26) | 3.68E-02 | 0.329 |
| CTBS | Q01459 | Inflammation II | 0.63(0.41,0.97) | 3.68E-02 | 0.329 |
| CDON | Q4KMG0 | Inflammation | 0.64(0.42,0.97) | 3.76E-02 | 0.332 |
| SYAP1 | Q96A49 | Inflammation II | 0.77(0.60,0.99) | 3.98E-02 | 0.340 |
| IL15 | P40933 | Inflammation | 1.41(1.02,1.95) | 4.01E-02 | 0.340 |
| MMP10 | P09238 | Inflammation | 1.21(1.01,1.46) | 4.01E-02 | 0.340 |
| NAGPA | Q9UK23 | Inflammation II | 0.54(0.30,0.98) | 4.11E-02 | 0.340 |
| FXYD5 | Q96DB9 | Inflammation | 0.82(0.68,0.99) | 4.12E-02 | 0.340 |
| CFP | P27918 | Inflammation II | 0.52(0.28,0.98) | 4.22E-02 | 0.340 |
| CTSC | P53634 | Inflammation | 0.76(0.58,0.99) | 4.24E-02 | 0.340 |
| EDAR | Q9UNE0 | Inflammation | 0.88(0.77,1.00) | 4.29E-02 | 0.340 |
| TIMP3 | P35625 | Inflammation | 0.91(0.83,1.00) | 4.31E-02 | 0.340 |
| APOL1 | O14791 | Inflammation II | 0.78(0.61,0.99) | 4.34E-02 | 0.340 |
| EVI5 | O60447 | Inflammation II | 0.86(0.74,1.00) | 4.38E-02 | 0.340 |
| APOC1 | P02654 | Inflammation II | 0.69(0.48,0.99) | 4.47E-02 | 0.340 |
| PLAUR | Q03405 | Inflammation | 1.50(1.01,2.23) | 4.48E-02 | 0.340 |
| FGF19 | O95750 | Inflammation | 0.86(0.74,1.00) | 4.52E-02 | 0.340 |
| CFI | P05156 | Inflammation II | 0.53(0.29,0.99) | 4.56E-02 | 0.340 |
| MYOM3 | Q5VTT5 | Inflammation II | 1.16(1.00,1.33) | 4.62E-02 | 0.341 |
| LRIG3 | Q6UXM1 | Inflammation II | 1.51(1.01,2.26) | 4.66E-02 | 0.341 |
| NRGN | Q92686 | Inflammation II | 0.86(0.75,1.00) | 4.79E-02 | 0.344 |
| SLC9A3R1 | O14745 | Inflammation II | 0.85(0.72,1.00) | 4.80E-02 | 0.344 |
| DECR1 | Q16698 | Inflammation | 0.89(0.79,1.00) | 4.89E-02 | 0.344 |
| CLEC12A | Q5QGZ9 | Inflammation II | 1.82(1.00,3.29) | 4.90E-02 | 0.344 |
| VEGFB | P49765 | Inflammation II | 1.55(1.00,2.41) | 4.95E-02 | 0.345 |
| F10 | P00742 | Inflammation II | 0.64(0.41,1.00) | 5.05E-02 | 0.345 |
| TPP1 | O14773 | Inflammation | 0.76(0.58,1.00) | 5.08E-02 | 0.345 |
| EPCAM | P16422 | Inflammation | 0.89(0.79,1.00) | 5.10E-02 | 0.345 |
| PDIA3 | P30101 | Inflammation II | 1.50(1.00,2.27) | 5.20E-02 | 0.349 |
| ITGA2 | P17301 | Inflammation II | 0.69(0.47,1.01) | 5.34E-02 | 0.355 |
| CD48 | P09326 | Inflammation | 1.43(0.99,2.06) | 5.46E-02 | 0.359 |
| ADIPOQ | Q15848 | Inflammation II | 1.19(1.00,1.43) | 5.65E-02 | 0.369 |
| ITGA6 | P23229 | Inflammation | 0.79(0.63,1.01) | 5.73E-02 | 0.370 |
| CXCL9 | Q07325 | Inflammation | 1.17(0.99,1.37) | 5.85E-02 | 0.375 |
| CD7 | P09564 | Inflammation II | 1.31(0.99,1.73) | 5.95E-02 | 0.378 |
| CCL24 | O00175 | Inflammation | 0.88(0.76,1.01) | 6.01E-02 | 0.378 |
| IL33 | O95760 | Inflammation | 0.76(0.56,1.01) | 6.09E-02 | 0.380 |
| METAP1D | Q6UB28 | Inflammation | 0.87(0.76,1.01) | 6.24E-02 | 0.386 |
| AMBN | Q9NP70 | Inflammation | 1.40(0.98,2.01) | 6.36E-02 | 0.390 |
| DAG1 | Q14118 | Inflammation | 0.79(0.62,1.01) | 6.44E-02 | 0.392 |
| NCK2 | O43639 | Inflammation | 0.90(0.81,1.01) | 6.53E-02 | 0.394 |
| SERPINA6 | P08185 | Inflammation II | 0.47(0.21,1.05) | 6.74E-02 | 0.402 |
| CKAP4 | Q07065 | Inflammation | 1.43(0.97,2.08) | 6.78E-02 | 0.402 |
| ATP5IF1 | Q9UII2 | Inflammation | 0.91(0.82,1.01) | 6.94E-02 | 0.405 |
| ATRN | O75882-2 | Inflammation II | 0.49(0.23,1.06) | 6.94E-02 | 0.405 |
| ADGRD1 | Q6QNK2 | Inflammation II | 0.72(0.51,1.03) | 7.07E-02 | 0.409 |
| ICA1 | Q05084 | Inflammation | 0.87(0.75,1.01) | 7.20E-02 | 0.414 |
| TLR4 | O00206 | Inflammation II | 1.47(0.96,2.23) | 7.33E-02 | 0.415 |
| IL17A | Q16552 | Inflammation | 0.76(0.56,1.03) | 7.40E-02 | 0.415 |
| F2 | P00734 | Inflammation II | 0.46(0.20,1.08) | 7.41E-02 | 0.415 |
| DTD1 | Q8TEA8 | Inflammation II | 0.87(0.74,1.01) | 7.49E-02 | 0.415 |
| AFM | P43652 | Inflammation II | 0.57(0.31,1.06) | 7.51E-02 | 0.415 |
| LZTFL1 | Q9NQ48 | Inflammation II | 0.84(0.69,1.02) | 7.84E-02 | 0.428 |
| TGOLN2 | O43493 | Inflammation II | 1.54(0.95,2.50) | 7.87E-02 | 0.428 |
| SERPIND1 | P05546 | Inflammation II | 0.67(0.42,1.05) | 7.96E-02 | 0.430 |
| SELPLG | Q14242 | Inflammation | 0.72(0.50,1.04) | 8.05E-02 | 0.430 |
| KLRF1 | Q9NZS2 | Inflammation II | 1.23(0.98,1.55) | 8.08E-02 | 0.430 |
| JCHAIN | P01591 | Inflammation | 1.17(0.98,1.41) | 8.29E-02 | 0.432 |
| F13B | P05160 | Inflammation II | 0.63(0.38,1.06) | 8.39E-02 | 0.432 |
| NELL2 | Q99435 | Inflammation | 0.70(0.47,1.05) | 8.41E-02 | 0.432 |
| GNPDA2 | Q8TDQ7 | Inflammation II | 0.72(0.50,1.05) | 8.41E-02 | 0.432 |
| ASGR2 | P07307 | Inflammation II | 0.65(0.39,1.06) | 8.42E-02 | 0.432 |
| CLSTN2 | Q9H4D0 | Inflammation | 1.26(0.97,1.65) | 8.71E-02 | 0.439 |
| SIGLEC1 | Q9BZZ2 | Inflammation | 1.25(0.97,1.62) | 8.78E-02 | 0.439 |
| LYVE1 | Q9Y5Y7 | Inflammation II | 1.78(0.92,3.43) | 8.78E-02 | 0.439 |
| CD83 | Q01151 | Inflammation | 1.32(0.96,1.80) | 8.82E-02 | 0.439 |
| APOF | Q13790 | Inflammation II | 0.72(0.50,1.05) | 8.85E-02 | 0.439 |
| SMOC2 | Q9H3U7 | Inflammation | 1.28(0.96,1.70) | 9.11E-02 | 0.444 |
| CPB2 | Q96IY4 | Inflammation II | 0.61(0.34,1.08) | 9.13E-02 | 0.444 |
| MGLL | Q99685 | Inflammation | 0.90(0.79,1.02) | 9.32E-02 | 0.444 |
| CD84 | Q9UIB8 | Inflammation | 0.73(0.51,1.05) | 9.38E-02 | 0.444 |
| CSF2RB | P32927 | Inflammation II | 1.32(0.95,1.81) | 9.46E-02 | 0.444 |
| ERP29 | P30040 | Inflammation II | 0.91(0.81,1.02) | 9.46E-02 | 0.444 |
| APOD | P05090 | Inflammation II | 1.24(0.96,1.60) | 9.54E-02 | 0.444 |
| EGF | P01133 | Inflammation | 0.91(0.82,1.02) | 9.55E-02 | 0.444 |
| SIRPB1 | O00241 | Inflammation | 1.23(0.96,1.56) | 9.56E-02 | 0.444 |
| PDE5A | O76074 | Inflammation II | 0.90(0.79,1.02) | 9.56E-02 | 0.444 |
| TNFSF12 | O43508 | Inflammation | 0.71(0.47,1.06) | 9.66E-02 | 0.445 |
| CEP164 | Q9UPV0 | Inflammation | 0.83(0.67,1.03) | 9.78E-02 | 0.445 |
| HLA-E | P13747 | Inflammation | 1.37(0.94,1.98) | 9.79E-02 | 0.445 |
| SLC39A5 | Q6ZMH5 | Inflammation | 0.83(0.67,1.03) | 9.84E-02 | 0.445 |
| CXCL17 | Q6UXB2 | Inflammation | 0.80(0.62,1.04) | 1.01E-01 | 0.448 |
| PROS1 | P07225 | Inflammation II | 0.65(0.39,1.09) | 1.02E-01 | 0.448 |
| NME1 | P15531 | Inflammation II | 1.63(0.91,2.94) | 1.03E-01 | 0.448 |
| SKAP2 | O75563 | Inflammation | 0.92(0.82,1.02) | 1.04E-01 | 0.448 |
| ST13 | P50502 | Inflammation II | 0.82(0.65,1.04) | 1.04E-01 | 0.448 |
| FLT3LG | P49771 | Inflammation | 0.77(0.56,1.06) | 1.05E-01 | 0.448 |
| CD40LG | P29965 | Inflammation | 0.89(0.77,1.03) | 1.06E-01 | 0.448 |
| GOPC | Q9HD26 | Inflammation | 0.91(0.82,1.02) | 1.07E-01 | 0.448 |
| NME3 | Q13232 | Inflammation | 0.66(0.39,1.09) | 1.07E-01 | 0.448 |
| PLA2G4A | P47712 | Inflammation | 0.90(0.79,1.02) | 1.07E-01 | 0.448 |
| TPSAB1 | Q15661 | Inflammation | 0.84(0.68,1.04) | 1.07E-01 | 0.448 |
| DNAJB6 | O75190 | Inflammation II | 0.88(0.74,1.03) | 1.07E-01 | 0.448 |
| IL12B | P29460 | Inflammation | 1.17(0.96,1.43) | 1.09E-01 | 0.448 |
| BCHE | P06276 | Inflammation II | 0.72(0.48,1.08) | 1.09E-01 | 0.448 |
| TFF2 | Q03403 | Inflammation | 1.17(0.97,1.41) | 1.10E-01 | 0.448 |
| VTI1A | Q96AJ9 | Inflammation II | 0.82(0.65,1.05) | 1.12E-01 | 0.448 |
| CD40 | P25942 | Inflammation | 0.82(0.65,1.05) | 1.13E-01 | 0.448 |
| PCDH1 | Q08174 | Inflammation | 0.63(0.36,1.11) | 1.13E-01 | 0.448 |
| DCTD | P32321 | Inflammation II | 0.89(0.78,1.03) | 1.13E-01 | 0.448 |
| SHMT1 | P34896 | Inflammation | 0.90(0.79,1.03) | 1.14E-01 | 0.448 |
| CRKL | P46109 | Inflammation | 0.92(0.83,1.02) | 1.15E-01 | 0.448 |
| FST | P19883 | Inflammation | 0.83(0.66,1.05) | 1.16E-01 | 0.448 |
| NT5C3A | Q9H0P0 | Inflammation | 0.90(0.79,1.03) | 1.16E-01 | 0.448 |
| CRIM1 | Q9NZV1 | Inflammation | 1.56(0.90,2.72) | 1.17E-01 | 0.448 |
| DEFB103A_DEFB103B | P81534 | Inflammation II | 1.22(0.95,1.56) | 1.17E-01 | 0.448 |
| LTBR | P36941 | Inflammation | 1.35(0.93,1.98) | 1.19E-01 | 0.448 |
| VASH1 | Q7L8A9 | Inflammation | 0.89(0.78,1.03) | 1.19E-01 | 0.448 |
| DAAM1 | Q9Y4D1 | Inflammation II | 0.88(0.75,1.03) | 1.19E-01 | 0.448 |
| PPL | O60437 | Inflammation II | 1.31(0.93,1.83) | 1.19E-01 | 0.448 |
| VNN1 | O95497 | Inflammation II | 0.87(0.74,1.04) | 1.20E-01 | 0.448 |
| OMD | Q99983 | Inflammation | 1.24(0.94,1.63) | 1.21E-01 | 0.448 |
| ADD1 | P35611 | Inflammation II | 0.86(0.72,1.04) | 1.21E-01 | 0.448 |
| BID | P55957 | Inflammation | 0.87(0.73,1.04) | 1.22E-01 | 0.448 |
| IFNGR1 | P15260 | Inflammation | 1.52(0.89,2.57) | 1.22E-01 | 0.448 |
| NCR1 | O76036 | Inflammation | 1.26(0.94,1.70) | 1.22E-01 | 0.448 |
| APOB | P04114 | Inflammation II | 0.64(0.37,1.13) | 1.22E-01 | 0.448 |
| IGLC2 | P0DOY2 | Inflammation II | 1.34(0.92,1.95) | 1.22E-01 | 0.448 |
| CD300A | Q9UGN4 | Inflammation II | 1.40(0.91,2.15) | 1.23E-01 | 0.450 |
| CACYBP | Q9HB71 | Inflammation II | 0.90(0.79,1.03) | 1.24E-01 | 0.451 |
| CHRDL1 | Q9BU40 | Inflammation | 1.32(0.93,1.89) | 1.25E-01 | 0.453 |
| GAL | P22466 | Inflammation | 1.14(0.96,1.36) | 1.27E-01 | 0.457 |
| FGF16 | O43320 | Inflammation II | 1.21(0.95,1.55) | 1.28E-01 | 0.459 |
| IL1RL2 | Q9HB29 | Inflammation | 0.79(0.59,1.07) | 1.29E-01 | 0.460 |
| RAB6A | P20340 | Inflammation | 0.82(0.64,1.06) | 1.30E-01 | 0.460 |
| RABEP1 | Q15276 | Inflammation II | 0.88(0.74,1.04) | 1.31E-01 | 0.460 |
| UBXN1 | Q04323 | Inflammation II | 0.86(0.70,1.05) | 1.31E-01 | 0.460 |
| RGS8 | P57771 | Inflammation | 0.86(0.70,1.05) | 1.33E-01 | 0.462 |
| C1S | P09871 | Inflammation II | 0.63(0.34,1.15) | 1.33E-01 | 0.462 |
| IFNLR1 | Q8IU57 | Inflammation | 0.77(0.56,1.08) | 1.34E-01 | 0.462 |
| IL7 | P13232 | Inflammation | 0.87(0.73,1.04) | 1.34E-01 | 0.462 |
| SERPINC1 | P01008 | Inflammation II | 0.52(0.22,1.23) | 1.35E-01 | 0.463 |
| IL21R | Q9HBE5 | Inflammation II | 1.17(0.95,1.43) | 1.36E-01 | 0.464 |
| FGF5 | P12034 | Inflammation | 1.28(0.92,1.79) | 1.38E-01 | 0.465 |
| GPI | P06744 | Inflammation II | 0.80(0.60,1.07) | 1.38E-01 | 0.465 |
| TNFAIP8L2 | Q6P589 | Inflammation II | 0.85(0.68,1.05) | 1.38E-01 | 0.465 |
| RBPMS | Q93062 | Inflammation II | 0.83(0.65,1.06) | 1.39E-01 | 0.466 |
| APPL2 | Q8NEU8 | Inflammation II | 0.92(0.82,1.03) | 1.41E-01 | 0.470 |
| FCRL6 | Q6DN72 | Inflammation | 1.13(0.96,1.33) | 1.43E-01 | 0.475 |
| DBN1 | Q16643 | Inflammation II | 0.82(0.62,1.07) | 1.45E-01 | 0.475 |
| ECM1 | Q16610 | Inflammation II | 0.77(0.55,1.09) | 1.45E-01 | 0.475 |
| STX5 | Q13190 | Inflammation II | 0.75(0.51,1.10) | 1.45E-01 | 0.475 |
| IL4R | P24394 | Inflammation | 1.25(0.93,1.68) | 1.47E-01 | 0.477 |
| SERPINI1 | Q99574 | Inflammation II | 0.71(0.45,1.13) | 1.47E-01 | 0.477 |
| SELENOP | P49908 | Inflammation II | 0.65(0.36,1.17) | 1.49E-01 | 0.479 |
| CLEC7A | Q9BXN2 | Inflammation | 1.17(0.95,1.45) | 1.50E-01 | 0.479 |
| PSPN | O60542 | Inflammation | 0.92(0.82,1.03) | 1.50E-01 | 0.479 |
| DFFA | O00273 | Inflammation | 0.88(0.74,1.05) | 1.51E-01 | 0.479 |
| PTRHD1 | Q6GMV3 | Inflammation II | 0.85(0.68,1.06) | 1.51E-01 | 0.479 |
| DENR | O43583 | Inflammation II | 0.83(0.65,1.07) | 1.53E-01 | 0.482 |
| SERPINA7 | P05543 | Inflammation II | 1.64(0.83,3.25) | 1.53E-01 | 0.482 |
| PTPRM | P28827 | Inflammation | 0.71(0.45,1.14) | 1.54E-01 | 0.483 |
| PIKFYVE | Q9Y2I7 | Inflammation II | 0.82(0.63,1.08) | 1.55E-01 | 0.484 |
| SH2D1A | O60880 | Inflammation | 0.86(0.69,1.06) | 1.57E-01 | 0.488 |
| LIFR | P42702 | Inflammation | 1.40(0.88,2.25) | 1.58E-01 | 0.489 |
| HGFAC | Q04756 | Inflammation II | 0.74(0.49,1.12) | 1.61E-01 | 0.494 |
| VAMP8 | Q9BV40 | Inflammation II | 0.90(0.77,1.04) | 1.61E-01 | 0.494 |
| NEXN | Q0ZGT2 | Inflammation II | 0.87(0.72,1.06) | 1.64E-01 | 0.501 |
| B2M | P61769 | Inflammation II | 1.26(0.91,1.75) | 1.65E-01 | 0.502 |
| CTSO | P43234 | Inflammation | 0.77(0.53,1.11) | 1.66E-01 | 0.503 |
| PLXNA4 | Q9HCM2 | Inflammation | 0.91(0.79,1.04) | 1.69E-01 | 0.510 |
| SELL | P14151 | Inflammation II | 0.68(0.39,1.18) | 1.71E-01 | 0.510 |
| SEMA3G | Q9NS98 | Inflammation II | 1.23(0.92,1.64) | 1.71E-01 | 0.510 |
| BACH1 | O14867 | Inflammation | 0.90(0.77,1.05) | 1.73E-01 | 0.510 |
| FGL1 | Q08830 | Inflammation II | 1.15(0.94,1.40) | 1.74E-01 | 0.510 |
| LAT | O43561 | Inflammation | 0.90(0.78,1.05) | 1.75E-01 | 0.510 |
| MANF | P55145 | Inflammation | 0.94(0.86,1.03) | 1.75E-01 | 0.510 |
| BMPER | Q8N8U9 | Inflammation II | 1.47(0.84,2.57) | 1.75E-01 | 0.510 |
| GIT1 | Q9Y2X7 | Inflammation II | 0.91(0.79,1.04) | 1.75E-01 | 0.510 |
| ITGA11 | Q9UKX5 | Inflammation | 0.80(0.57,1.11) | 1.76E-01 | 0.510 |
| PRKG1 | Q13976 | Inflammation II | 0.93(0.84,1.03) | 1.76E-01 | 0.510 |
| FOXO1 | Q12778 | Inflammation | 0.91(0.80,1.04) | 1.77E-01 | 0.511 |
| GIPR | P48546 | Inflammation II | 1.20(0.92,1.55) | 1.78E-01 | 0.511 |
| F11 | P03951 | Inflammation II | 0.73(0.46,1.16) | 1.81E-01 | 0.518 |
| MGMT | P16455 | Inflammation | 0.94(0.86,1.03) | 1.82E-01 | 0.519 |
| RABGAP1L | Q5R372 | Inflammation | 0.88(0.72,1.06) | 1.83E-01 | 0.519 |
| CDSN | Q15517 | Inflammation | 1.19(0.92,1.54) | 1.85E-01 | 0.519 |
| PROK1 | P58294 | Inflammation | 1.14(0.94,1.39) | 1.85E-01 | 0.519 |
| CR1 | P17927 | Inflammation II | 1.28(0.89,1.84) | 1.86E-01 | 0.519 |
| DBH | P09172 | Inflammation II | 0.93(0.83,1.04) | 1.86E-01 | 0.519 |
| FKBP1B | P68106 | Inflammation | 0.93(0.84,1.03) | 1.87E-01 | 0.519 |
| PCBD1 | P61457 | Inflammation II | 0.82(0.61,1.10) | 1.87E-01 | 0.519 |
| MPIG6B | O95866 | Inflammation | 0.91(0.79,1.05) | 1.89E-01 | 0.519 |
| FGF6 | P10767 | Inflammation II | 1.22(0.91,1.63) | 1.89E-01 | 0.519 |
| TLR3 | O15455 | Inflammation | 0.89(0.74,1.06) | 1.90E-01 | 0.519 |
| C7 | P10643 | Inflammation II | 1.33(0.87,2.05) | 1.90E-01 | 0.519 |
| MARS1 | P56192 | Inflammation II | 0.90(0.77,1.05) | 1.91E-01 | 0.520 |
| ANGPT1 | Q15389 | Inflammation | 0.92(0.80,1.05) | 1.96E-01 | 0.527 |
| TREML1 | Q86YW5 | Inflammation II | 0.84(0.65,1.09) | 1.96E-01 | 0.527 |
| RNASE1 | P07998 | Inflammation II | 1.36(0.85,2.18) | 1.98E-01 | 0.527 |
| MAP2K6 | P52564 | Inflammation | 0.93(0.83,1.04) | 1.99E-01 | 0.527 |
| TNFRSF4 | P43489 | Inflammation | 1.20(0.91,1.59) | 1.99E-01 | 0.527 |
| EIF4E | P06730 | Inflammation II | 0.91(0.78,1.05) | 2.00E-01 | 0.527 |
| FGFR4 | P22455 | Inflammation II | 0.85(0.66,1.09) | 2.00E-01 | 0.527 |
| POLR2A | P24928 | Inflammation II | 1.30(0.87,1.94) | 2.00E-01 | 0.527 |
| CD200 | P41217 | Inflammation | 1.29(0.87,1.91) | 2.01E-01 | 0.527 |
| IL12RB1 | P42701 | Inflammation | 1.21(0.90,1.63) | 2.01E-01 | 0.527 |
| MEGF10 | Q96KG7 | Inflammation | 1.21(0.90,1.63) | 2.02E-01 | 0.528 |
| UROD | P06132 | Inflammation II | 0.88(0.73,1.07) | 2.03E-01 | 0.529 |
| NCLN | Q969V3 | Inflammation | 0.84(0.65,1.10) | 2.04E-01 | 0.529 |
| DNAJA2 | O60884 | Inflammation | 0.92(0.80,1.05) | 2.08E-01 | 0.529 |
| TREM2 | Q9NZC2 | Inflammation | 0.87(0.70,1.08) | 2.08E-01 | 0.529 |
| GCHFR | P30047 | Inflammation II | 0.82(0.61,1.11) | 2.08E-01 | 0.529 |
| CXCL6 | P80162 | Inflammation | 0.91(0.78,1.05) | 2.09E-01 | 0.529 |
| FGF2 | P09038 | Inflammation | 0.90(0.76,1.06) | 2.09E-01 | 0.529 |
| AKR7L | Q8NHP1 | Inflammation II | 1.13(0.94,1.36) | 2.09E-01 | 0.529 |
| RNASE6 | Q93091 | Inflammation II | 1.27(0.87,1.84) | 2.11E-01 | 0.529 |
| CSF3R | Q99062 | Inflammation II | 1.32(0.85,2.04) | 2.13E-01 | 0.529 |
| GLRX5 | Q86SX6 | Inflammation II | 0.91(0.78,1.06) | 2.13E-01 | 0.529 |
| NDUFA5 | Q16718 | Inflammation II | 0.78(0.53,1.15) | 2.13E-01 | 0.529 |
| UBE2Z | Q9H832 | Inflammation II | 0.85(0.66,1.10) | 2.14E-01 | 0.529 |
| IL4 | P05112 | Inflammation | 0.93(0.84,1.04) | 2.15E-01 | 0.529 |
| STAT2 | P52630 | Inflammation II | 0.89(0.75,1.07) | 2.15E-01 | 0.529 |
| YWHAQ | P27348 | Inflammation II | 0.89(0.74,1.07) | 2.15E-01 | 0.529 |
| ZNF174 | Q15697 | Inflammation II | 1.23(0.89,1.71) | 2.15E-01 | 0.529 |
| ACYP1 | P07311 | Inflammation II | 0.88(0.72,1.08) | 2.16E-01 | 0.530 |
| GAPDH | P04406 | Inflammation II | 0.79(0.54,1.15) | 2.17E-01 | 0.531 |
| PPBP | P02775 | Inflammation II | 0.91(0.78,1.06) | 2.18E-01 | 0.531 |
| BABAM1 | Q9NWV8 | Inflammation II | 1.29(0.86,1.93) | 2.23E-01 | 0.542 |
| BCR | P11274 | Inflammation | 0.93(0.82,1.05) | 2.25E-01 | 0.542 |
| CCL25 | O15444 | Inflammation | 1.15(0.92,1.46) | 2.26E-01 | 0.542 |
| CST7 | O76096 | Inflammation | 0.92(0.81,1.05) | 2.26E-01 | 0.542 |
| HCLS1 | P14317 | Inflammation | 0.88(0.72,1.08) | 2.26E-01 | 0.542 |
| PGF | P49763 | Inflammation | 1.31(0.85,2.02) | 2.28E-01 | 0.545 |
| AMN | Q9BXJ7 | Inflammation | 0.86(0.67,1.10) | 2.29E-01 | 0.545 |
| TNFAIP8 | O95379 | Inflammation | 0.82(0.59,1.13) | 2.30E-01 | 0.546 |
| MVK | Q03426 | Inflammation | 0.91(0.79,1.06) | 2.32E-01 | 0.547 |
| LRG1 | P02750 | Inflammation II | 1.34(0.83,2.16) | 2.32E-01 | 0.547 |
| KLRD1 | Q13241 | Inflammation | 0.87(0.68,1.10) | 2.35E-01 | 0.552 |
| IRAK1 | P51617 | Inflammation | 0.90(0.76,1.07) | 2.39E-01 | 0.556 |
| PON3 | Q15166 | Inflammation | 0.81(0.57,1.15) | 2.39E-01 | 0.556 |
| PHYKPL | Q8IUZ5 | Inflammation II | 0.83(0.60,1.13) | 2.39E-01 | 0.556 |
| SHH | Q15465 | Inflammation II | 1.19(0.89,1.61) | 2.40E-01 | 0.557 |
| SPART | Q8N0X7 | Inflammation II | 0.90(0.76,1.07) | 2.41E-01 | 0.557 |
| TSPYL1 | Q9H0U9 | Inflammation II | 0.81(0.57,1.15) | 2.43E-01 | 0.560 |
| SHBG | P04278 | Inflammation II | 1.16(0.90,1.50) | 2.44E-01 | 0.560 |
| PARP1 | P09874 | Inflammation | 0.88(0.70,1.09) | 2.45E-01 | 0.560 |
| HMCN2 | Q8NDA2 | Inflammation II | 1.15(0.91,1.46) | 2.45E-01 | 0.560 |
| FGA | P02671 | Inflammation II | 0.77(0.50,1.19) | 2.46E-01 | 0.560 |
| CEACAM21 | Q3KPI0 | Inflammation | 1.08(0.95,1.23) | 2.48E-01 | 0.562 |
| CXADR | P78310 | Inflammation | 0.87(0.69,1.10) | 2.49E-01 | 0.562 |
| F2R | P25116 | Inflammation | 0.89(0.72,1.09) | 2.49E-01 | 0.562 |
| CD58 | P19256 | Inflammation | 1.46(0.76,2.79) | 2.52E-01 | 0.565 |
| LY9 | Q9HBG7 | Inflammation | 1.25(0.86,1.81) | 2.52E-01 | 0.565 |
| GZMA | P12544 | Inflammation | 0.83(0.60,1.15) | 2.58E-01 | 0.575 |
| RLN2 | P04090 | Inflammation II | 1.13(0.91,1.40) | 2.58E-01 | 0.575 |
| PTPN6 | P29350 | Inflammation | 0.93(0.82,1.05) | 2.61E-01 | 0.576 |
| PINLYP | A6NC86 | Inflammation II | 0.90(0.75,1.08) | 2.61E-01 | 0.576 |
| CD5L | O43866 | Inflammation II | 0.85(0.64,1.13) | 2.62E-01 | 0.576 |
| TBCA | O75347 | Inflammation II | 0.91(0.78,1.07) | 2.63E-01 | 0.576 |
| IL10RB | Q08334 | Inflammation | 1.24(0.85,1.81) | 2.64E-01 | 0.576 |
| DAPP1 | Q9UN19 | Inflammation | 0.95(0.87,1.04) | 2.65E-01 | 0.576 |
| DBNL | Q9UJU6 | Inflammation | 0.93(0.83,1.05) | 2.65E-01 | 0.576 |
| NFATC3 | Q12968 | Inflammation | 0.89(0.73,1.09) | 2.65E-01 | 0.576 |
| SRPK2 | P78362 | Inflammation | 0.92(0.81,1.06) | 2.68E-01 | 0.579 |
| NAGA | P17050 | Inflammation II | 0.86(0.66,1.12) | 2.68E-01 | 0.579 |
| IRAK4 | Q9NWZ3 | Inflammation | 0.94(0.85,1.05) | 2.69E-01 | 0.580 |
| BANK1 | Q8NDB2 | Inflammation | 0.94(0.85,1.05) | 2.70E-01 | 0.580 |
| MILR1 | Q7Z6M3 | Inflammation | 1.13(0.91,1.42) | 2.71E-01 | 0.580 |
| ACP1 | P24666 | Inflammation II | 0.84(0.61,1.15) | 2.72E-01 | 0.581 |
| ARHGEF12 | Q9NZN5 | Inflammation | 0.94(0.85,1.05) | 2.73E-01 | 0.581 |
| CASP9 | P55211 | Inflammation II | 0.88(0.70,1.10) | 2.75E-01 | 0.584 |
| IDS | P22304 | Inflammation | 2.09(0.55,7.92) | 2.76E-01 | 0.584 |
| MCEMP1 | Q8IX19 | Inflammation II | 0.92(0.79,1.07) | 2.78E-01 | 0.587 |
| PDGFB | P01127 | Inflammation | 0.93(0.82,1.06) | 2.79E-01 | 0.587 |
| BTN2A1 | Q7KYR7 | Inflammation | 1.25(0.83,1.89) | 2.81E-01 | 0.590 |
| CD36 | P16671 | Inflammation II | 0.85(0.62,1.15) | 2.84E-01 | 0.592 |
| PTGES2 | Q9H7Z7 | Inflammation II | 0.87(0.67,1.12) | 2.84E-01 | 0.592 |
| NHLRC3 | Q5JS37 | Inflammation II | 0.80(0.53,1.21) | 2.85E-01 | 0.592 |
| CSNK1D | P48730 | Inflammation II | 0.89(0.71,1.11) | 2.86E-01 | 0.592 |
| MTDH | Q86UE4 | Inflammation II | 0.92(0.80,1.07) | 2.86E-01 | 0.592 |
| PF4 | P02776 | Inflammation II | 0.93(0.81,1.06) | 2.87E-01 | 0.592 |
| AXIN1 | O15169 | Inflammation | 0.94(0.84,1.06) | 2.91E-01 | 0.599 |
| LGALS9 | O00182 | Inflammation | 1.20(0.85,1.70) | 2.92E-01 | 0.599 |
| LGALS3BP | Q08380 | Inflammation II | 1.24(0.83,1.84) | 2.95E-01 | 0.602 |
| TANK | Q92844 | Inflammation | 0.91(0.77,1.08) | 2.96E-01 | 0.602 |
| PTPN9 | P43378 | Inflammation II | 0.82(0.56,1.20) | 2.96E-01 | 0.602 |
| NUDC | Q9Y266 | Inflammation | 0.91(0.76,1.09) | 2.98E-01 | 0.604 |
| AGER | Q15109 | Inflammation | 0.86(0.66,1.14) | 3.01E-01 | 0.606 |
| TGFB1 | P01137 | Inflammation | 0.85(0.63,1.15) | 3.01E-01 | 0.606 |
| DGKA | P23743 | Inflammation II | 0.88(0.68,1.13) | 3.02E-01 | 0.606 |
| BCL2L11 | O43521-2 | Inflammation | 1.16(0.88,1.53) | 3.03E-01 | 0.606 |
| PAXX | Q9BUH6 | Inflammation II | 0.85(0.63,1.15) | 3.03E-01 | 0.606 |
| IL1R2 | P27930 | Inflammation | 0.75(0.42,1.31) | 3.07E-01 | 0.612 |
| FASLG | P48023 | Inflammation | 0.86(0.63,1.16) | 3.08E-01 | 0.613 |
| ITIH1 | P19827 | Inflammation II | 0.70(0.35,1.40) | 3.10E-01 | 0.613 |
| ITIH4 | Q14624 | Inflammation II | 0.77(0.46,1.28) | 3.10E-01 | 0.613 |
| CFHR5 | Q9BXR6 | Inflammation II | 0.86(0.63,1.16) | 3.12E-01 | 0.615 |
| TNFRSF11A | Q9Y6Q6 | Inflammation | 1.15(0.88,1.51) | 3.13E-01 | 0.615 |
| SPRY2 | O43597 | Inflammation | 0.94(0.83,1.06) | 3.15E-01 | 0.615 |
| XIAP | P98170 | Inflammation II | 0.89(0.70,1.12) | 3.16E-01 | 0.615 |
| SNX15 | Q9NRS6 | Inflammation II | 0.88(0.69,1.13) | 3.17E-01 | 0.615 |
| IL20RA | Q9UHF4 | Inflammation | 1.13(0.89,1.43) | 3.18E-01 | 0.615 |
| INSR | P06213 | Inflammation II | 1.48(0.69,3.20) | 3.18E-01 | 0.615 |
| KLK7 | P49862 | Inflammation II | 0.83(0.58,1.19) | 3.18E-01 | 0.615 |
| ACHE | P22303 | Inflammation II | 0.81(0.53,1.23) | 3.20E-01 | 0.615 |
| PADI2 | Q9Y2J8 | Inflammation | 1.11(0.91,1.35) | 3.21E-01 | 0.615 |
| MFAP4 | P55083 | Inflammation II | 1.23(0.81,1.87) | 3.21E-01 | 0.615 |
| TP53I3 | Q53FA7 | Inflammation II | 0.88(0.68,1.14) | 3.21E-01 | 0.615 |
| IL36G | Q9NZH8 | Inflammation II | 1.10(0.91,1.31) | 3.22E-01 | 0.615 |
| WASL | O00401 | Inflammation II | 0.88(0.69,1.13) | 3.23E-01 | 0.615 |
| IL2 | P60568 | Inflammation | 1.20(0.83,1.73) | 3.28E-01 | 0.623 |
| CD226 | Q15762 | Inflammation II | 0.85(0.62,1.18) | 3.29E-01 | 0.624 |
| ARHGAP45 | Q92619 | Inflammation II | 0.92(0.79,1.08) | 3.32E-01 | 0.628 |
| CFHR2 | P36980 | Inflammation II | 1.13(0.89,1.43) | 3.33E-01 | 0.628 |
| OSM | P13725 | Inflammation | 1.08(0.92,1.27) | 3.37E-01 | 0.634 |
| C3 | P01024 | Inflammation II | 0.86(0.63,1.17) | 3.38E-01 | 0.634 |
| CASP2 | P42575 | Inflammation | 0.92(0.78,1.09) | 3.45E-01 | 0.645 |
| NFATC1 | O95644 | Inflammation | 0.95(0.84,1.06) | 3.47E-01 | 0.647 |
| SFRP4 | Q6FHJ7 | Inflammation II | 0.86(0.62,1.19) | 3.49E-01 | 0.650 |
| HGF | P14210 | Inflammation | 1.16(0.85,1.57) | 3.52E-01 | 0.653 |
| HSPA1A | P0DMV8 | Inflammation | 0.93(0.79,1.09) | 3.53E-01 | 0.654 |
| HRG | P04196 | Inflammation II | 0.82(0.54,1.25) | 3.54E-01 | 0.654 |
| CCL3 | P10147 | Inflammation | 0.91(0.74,1.12) | 3.61E-01 | 0.665 |
| NPHS1 | O60500 | Inflammation II | 0.83(0.55,1.25) | 3.63E-01 | 0.666 |
| AGRP | O00253 | Inflammation | 0.90(0.73,1.12) | 3.64E-01 | 0.666 |
| TPT1 | P13693 | Inflammation | 0.91(0.75,1.11) | 3.65E-01 | 0.666 |
| SEMA6C | Q9H3T2 | Inflammation II | 1.24(0.78,1.96) | 3.65E-01 | 0.666 |
| PER3 | P56645 | Inflammation II | 0.95(0.85,1.06) | 3.67E-01 | 0.668 |
| MDH1 | P40925 | Inflammation II | 0.86(0.62,1.20) | 3.69E-01 | 0.670 |
| CCL4 | P13236 | Inflammation | 0.92(0.78,1.10) | 3.70E-01 | 0.670 |
| AGT | P01019 | Inflammation II | 1.30(0.73,2.31) | 3.72E-01 | 0.672 |
| ADAMTS1 | Q9UHI8 | Inflammation II | 1.22(0.79,1.87) | 3.73E-01 | 0.672 |
| HIF1A | Q16665 | Inflammation II | 0.90(0.71,1.13) | 3.74E-01 | 0.672 |
| SULT2A1 | Q06520 | Inflammation | 0.91(0.73,1.13) | 3.79E-01 | 0.676 |
| RICTOR | Q6R327 | Inflammation II | 1.11(0.88,1.42) | 3.79E-01 | 0.676 |
| SSBP1 | Q04837 | Inflammation II | 1.18(0.82,1.71) | 3.79E-01 | 0.676 |
| CD160 | O95971 | Inflammation | 1.11(0.88,1.40) | 3.81E-01 | 0.678 |
| CXCL1 | P09341 | Inflammation | 0.95(0.84,1.07) | 3.84E-01 | 0.680 |
| IL18 | Q14116 | Inflammation | 0.90(0.70,1.15) | 3.84E-01 | 0.680 |
| CCN2 | P29279 | Inflammation | 0.91(0.75,1.12) | 3.87E-01 | 0.683 |
| TRAF3 | Q13114-2 | Inflammation II | 0.84(0.57,1.24) | 3.89E-01 | 0.685 |
| HLA-DRA | P01903 | Inflammation | 0.87(0.63,1.20) | 3.92E-01 | 0.689 |
| FGF20 | Q9NP95 | Inflammation II | 0.86(0.61,1.21) | 3.94E-01 | 0.689 |
| TSC1 | Q92574 | Inflammation II | 0.92(0.75,1.12) | 3.94E-01 | 0.689 |
| GBP2 | P32456 | Inflammation | 0.92(0.75,1.12) | 3.98E-01 | 0.691 |
| ADAM12 | O43184 | Inflammation II | 1.18(0.80,1.74) | 3.98E-01 | 0.691 |
| SERPINA1 | P01009 | Inflammation II | 1.89(0.43,8.31) | 3.98E-01 | 0.691 |
| AFAP1 | Q8N556 | Inflammation II | 0.93(0.79,1.10) | 4.00E-01 | 0.692 |
| TNFRSF14 | Q92956 | Inflammation | 1.17(0.81,1.69) | 4.02E-01 | 0.692 |
| IL5RA | Q01344 | Inflammation | 1.09(0.89,1.33) | 4.03E-01 | 0.692 |
| TERF1 | P54274 | Inflammation II | 1.20(0.78,1.86) | 4.04E-01 | 0.692 |
| IL1A | P01583 | Inflammation | 1.08(0.90,1.29) | 4.05E-01 | 0.692 |
| C8B | P07358 | Inflammation II | 0.83(0.54,1.28) | 4.05E-01 | 0.692 |
| GLOD4 | Q9HC38 | Inflammation | 0.87(0.64,1.20) | 4.06E-01 | 0.692 |
| LAMA4 | Q16363 | Inflammation | 0.87(0.61,1.22) | 4.07E-01 | 0.692 |
| SPRED2 | Q7Z698 | Inflammation II | 0.87(0.63,1.21) | 4.07E-01 | 0.692 |
| LAMP3 | Q9UQV4 | Inflammation | 0.92(0.74,1.13) | 4.08E-01 | 0.692 |
| MRPS16 | Q9Y3D3 | Inflammation II | 0.80(0.47,1.37) | 4.12E-01 | 0.697 |
| DAND5 | Q8N907 | Inflammation II | 1.10(0.88,1.38) | 4.15E-01 | 0.699 |
| IL13 | P35225 | Inflammation | 0.89(0.68,1.18) | 4.17E-01 | 0.699 |
| C1QTNF9 | P0C862 | Inflammation II | 0.90(0.68,1.17) | 4.17E-01 | 0.699 |
| EPHA4 | P54764 | Inflammation II | 1.21(0.76,1.93) | 4.19E-01 | 0.699 |
| MMP1 | P03956 | Inflammation | 0.95(0.84,1.08) | 4.20E-01 | 0.699 |
| CELSR2 | Q9HCU4 | Inflammation II | 1.21(0.76,1.90) | 4.21E-01 | 0.699 |
| GSN | P06396 | Inflammation II | 1.34(0.66,2.75) | 4.22E-01 | 0.699 |
| CXCL3 | P19876 | Inflammation | 0.95(0.85,1.07) | 4.23E-01 | 0.699 |
| CSF3 | P09919 | Inflammation | 0.91(0.71,1.15) | 4.24E-01 | 0.699 |
| SAMD9L | Q8IVG5 | Inflammation | 0.94(0.80,1.10) | 4.24E-01 | 0.699 |
| VEGFA | P15692 | Inflammation | 0.92(0.76,1.12) | 4.24E-01 | 0.699 |
| SCGN | O76038 | Inflammation | 1.11(0.86,1.42) | 4.25E-01 | 0.699 |
| IL17RB | Q9NRM6 | Inflammation | 1.09(0.88,1.35) | 4.28E-01 | 0.701 |
| CPA4 | Q9UI42 | Inflammation II | 0.88(0.65,1.20) | 4.28E-01 | 0.701 |
| PPP1R9B | Q96SB3 | Inflammation | 0.95(0.85,1.07) | 4.31E-01 | 0.704 |
| PSMG4 | Q5JS54 | Inflammation II | 1.09(0.87,1.37) | 4.35E-01 | 0.705 |
| SERPING1 | P05155 | Inflammation II | 0.75(0.37,1.54) | 4.35E-01 | 0.705 |
| CCL21 | O00585 | Inflammation | 1.11(0.86,1.44) | 4.36E-01 | 0.705 |
| S100A13 | Q99584 | Inflammation II | 0.86(0.58,1.26) | 4.36E-01 | 0.705 |
| GAD2 | Q05329 | Inflammation II | 1.11(0.85,1.45) | 4.37E-01 | 0.705 |
| SPINK2 | P20155 | Inflammation II | 1.15(0.81,1.64) | 4.38E-01 | 0.705 |
| BSG | P35613 | Inflammation | 1.24(0.72,2.13) | 4.39E-01 | 0.705 |
| PRSS22 | Q9GZN4 | Inflammation II | 1.15(0.81,1.63) | 4.39E-01 | 0.705 |
| COLEC12 | Q5KU26 | Inflammation | 1.17(0.78,1.74) | 4.42E-01 | 0.706 |
| JUN | P05412 | Inflammation | 1.09(0.87,1.36) | 4.42E-01 | 0.706 |
| CXCL10 | P02778 | Inflammation | 0.93(0.79,1.11) | 4.44E-01 | 0.707 |
| JAM3 | Q9BX67 | Inflammation II | 0.90(0.68,1.19) | 4.45E-01 | 0.707 |
| SDK2 | Q58EX2 | Inflammation II | 0.87(0.61,1.24) | 4.45E-01 | 0.707 |
| ACRV1 | P26436 | Inflammation II | 1.06(0.92,1.22) | 4.47E-01 | 0.708 |
| HPCAL1 | P37235 | Inflammation | 0.94(0.81,1.10) | 4.51E-01 | 0.710 |
| DDX4 | Q9NQI0 | Inflammation II | 0.90(0.68,1.18) | 4.51E-01 | 0.710 |
| DNAJB2 | P25686 | Inflammation II | 0.91(0.71,1.16) | 4.52E-01 | 0.710 |
| CCL23 | P55773 | Inflammation | 0.90(0.69,1.18) | 4.53E-01 | 0.710 |
| SCGB3A2 | Q96PL1 | Inflammation | 1.05(0.93,1.19) | 4.53E-01 | 0.710 |
| CCL26 | Q9Y258 | Inflammation | 0.95(0.84,1.08) | 4.55E-01 | 0.711 |
| IL17D | Q8TAD2 | Inflammation | 1.12(0.82,1.54) | 4.59E-01 | 0.713 |
| ALPI | P09923 | Inflammation II | 0.96(0.86,1.07) | 4.59E-01 | 0.713 |
| KDM3A | Q9Y4C1 | Inflammation II | 0.94(0.79,1.11) | 4.59E-01 | 0.713 |
| SPINT2 | O43291 | Inflammation | 0.90(0.68,1.19) | 4.60E-01 | 0.713 |
| SCGB1A1 | P11684 | Inflammation | 0.93(0.76,1.13) | 4.61E-01 | 0.713 |
| NUB1 | Q9Y5A7 | Inflammation | 0.95(0.83,1.09) | 4.64E-01 | 0.715 |
| APOA1 | P02647 | Inflammation II | 0.83(0.51,1.36) | 4.64E-01 | 0.715 |
| DDX39A | O00148 | Inflammation II | 1.19(0.75,1.90) | 4.66E-01 | 0.716 |
| ALDH3A1 | P30838 | Inflammation | 0.94(0.78,1.12) | 4.71E-01 | 0.721 |
| WFIKKN2 | Q8TEU8 | Inflammation | 1.12(0.82,1.54) | 4.73E-01 | 0.721 |
| ADAMTS4 | O75173 | Inflammation II | 1.15(0.79,1.67) | 4.73E-01 | 0.721 |
| INHBB | P09529 | Inflammation II | 1.09(0.86,1.40) | 4.73E-01 | 0.721 |
| IFNG | P01579 | Inflammation | 1.04(0.94,1.15) | 4.76E-01 | 0.724 |
| MICB_MICA | Q29980_Q29983 | Inflammation | 1.03(0.95,1.11) | 4.79E-01 | 0.727 |
| IL10RA | Q13651 | Inflammation | 1.07(0.89,1.28) | 4.81E-01 | 0.729 |
| WAS | P42768 | Inflammation | 1.06(0.90,1.25) | 4.87E-01 | 0.736 |
| ANKMY2 | Q8IV38 | Inflammation II | 0.94(0.80,1.11) | 4.89E-01 | 0.736 |
| SUMF1 | Q8NBK3 | Inflammation II | 1.07(0.89,1.29) | 4.89E-01 | 0.736 |
| MATN2 | O00339 | Inflammation | 1.15(0.78,1.69) | 4.90E-01 | 0.736 |
| PGR | P06401 | Inflammation II | 0.95(0.82,1.10) | 4.92E-01 | 0.737 |
| ARTN | Q5T4W7 | Inflammation | 1.09(0.85,1.39) | 4.96E-01 | 0.742 |
| PDLIM7 | Q9NR12 | Inflammation | 0.97(0.89,1.06) | 4.97E-01 | 0.742 |
| SERPINA3 | P01011 | Inflammation II | 1.33(0.58,3.05) | 5.01E-01 | 0.746 |
| PRSS8 | Q16651 | Inflammation | 1.09(0.84,1.42) | 5.05E-01 | 0.747 |
| IKBKG | Q9Y6K9 | Inflammation | 0.96(0.85,1.08) | 5.06E-01 | 0.747 |
| MAPK9 | P45984 | Inflammation | 1.10(0.84,1.44) | 5.07E-01 | 0.747 |
| CLEC4A | Q9UMR7 | Inflammation | 1.12(0.81,1.54) | 5.08E-01 | 0.747 |
| BCL2L15 | Q5TBC7 | Inflammation II | 1.08(0.86,1.35) | 5.08E-01 | 0.747 |
| RALB | P11234 | Inflammation II | 0.86(0.55,1.35) | 5.09E-01 | 0.747 |
| TF | P02787 | Inflammation II | 0.81(0.44,1.50) | 5.09E-01 | 0.747 |
| AKAP12 | Q02952 | Inflammation II | 1.23(0.66,2.27) | 5.11E-01 | 0.749 |
| PTP4A3 | O75365 | Inflammation II | 1.08(0.86,1.37) | 5.12E-01 | 0.749 |
| EIF5A | P63241 | Inflammation | 0.87(0.58,1.31) | 5.15E-01 | 0.752 |
| TRIM5 | Q9C035 | Inflammation | 0.96(0.84,1.09) | 5.20E-01 | 0.756 |
| MBL2 | P11226 | Inflammation II | 1.04(0.92,1.18) | 5.20E-01 | 0.756 |
| MEPE | Q9NQ76 | Inflammation | 0.86(0.55,1.35) | 5.22E-01 | 0.757 |
| TNFRSF13C | Q96RJ3 | Inflammation | 1.09(0.84,1.42) | 5.23E-01 | 0.757 |
| QSOX1 | O00391 | Inflammation II | 0.89(0.62,1.28) | 5.27E-01 | 0.762 |
| RIDA | P52758 | Inflammation II | 0.92(0.70,1.20) | 5.30E-01 | 0.763 |
| TXN | P10599 | Inflammation II | 0.91(0.67,1.23) | 5.30E-01 | 0.763 |
| B4GALT1 | P15291 | Inflammation | 1.14(0.76,1.71) | 5.32E-01 | 0.763 |
| ISM1 | B1AKI9 | Inflammation | 0.91(0.68,1.22) | 5.32E-01 | 0.763 |
| TP53BP1 | Q12888 | Inflammation II | 0.95(0.81,1.11) | 5.33E-01 | 0.763 |
| C1QL2 | Q7Z5L3 | Inflammation II | 0.91(0.66,1.24) | 5.36E-01 | 0.765 |
| PRDX5 | P30044 | Inflammation | 0.95(0.80,1.12) | 5.39E-01 | 0.767 |
| GC | P02774 | Inflammation II | 0.85(0.51,1.42) | 5.39E-01 | 0.767 |
| CD22 | P20273 | Inflammation | 1.08(0.84,1.40) | 5.40E-01 | 0.767 |
| RAP1A | P62834 | Inflammation II | 0.94(0.76,1.16) | 5.43E-01 | 0.769 |
| LTA | P01374 | Inflammation | 1.09(0.83,1.44) | 5.44E-01 | 0.769 |
| PI16 | Q6UXB8 | Inflammation II | 1.18(0.69,2.01) | 5.50E-01 | 0.776 |
| DPP10 | Q8N608 | Inflammation | 1.10(0.80,1.51) | 5.53E-01 | 0.779 |
| CXCL12 | P48061 | Inflammation | 1.09(0.82,1.46) | 5.55E-01 | 0.780 |
| FABP9 | Q0Z7S8 | Inflammation | 0.94(0.77,1.15) | 5.57E-01 | 0.782 |
| FCAR | P24071 | Inflammation | 1.07(0.85,1.36) | 5.62E-01 | 0.787 |
| ANXA11 | P50995 | Inflammation | 0.95(0.81,1.12) | 5.64E-01 | 0.787 |
| EIF4G1 | Q04637 | Inflammation | 0.97(0.87,1.08) | 5.65E-01 | 0.787 |
| RNF31 | Q96EP0 | Inflammation II | 1.11(0.77,1.59) | 5.66E-01 | 0.787 |
| IL11 | P20809 | Inflammation | 0.94(0.77,1.15) | 5.67E-01 | 0.787 |
| IL20 | Q9NYY1 | Inflammation | 0.93(0.74,1.18) | 5.71E-01 | 0.787 |
| NXPH3 | O95157 | Inflammation II | 1.14(0.73,1.77) | 5.71E-01 | 0.787 |
| BMP10 | O95393 | Inflammation II | 1.14(0.72,1.83) | 5.73E-01 | 0.787 |
| CGB3_CGB5_CGB8 | P0DN86 | Inflammation II | 1.10(0.79,1.54) | 5.73E-01 | 0.787 |
| FOXJ3 | Q9UPW0 | Inflammation II | 0.91(0.67,1.25) | 5.73E-01 | 0.787 |
| STX7 | O15400 | Inflammation II | 0.93(0.71,1.21) | 5.73E-01 | 0.787 |
| PREB | Q9HCU5 | Inflammation | 0.89(0.59,1.34) | 5.82E-01 | 0.798 |
| HEG1 | Q9ULI3 | Inflammation II | 0.87(0.53,1.43) | 5.86E-01 | 0.802 |
| IL3RA | P26951 | Inflammation | 1.10(0.77,1.58) | 5.89E-01 | 0.805 |
| AOC1 | P19801 | Inflammation | 0.94(0.76,1.17) | 5.92E-01 | 0.806 |
| ACE | P12821 | Inflammation II | 1.14(0.70,1.85) | 5.93E-01 | 0.806 |
| TRIM21 | P19474 | Inflammation | 0.96(0.83,1.11) | 5.94E-01 | 0.806 |
| CLIP2 | Q9UDT6 | Inflammation | 0.97(0.88,1.07) | 5.96E-01 | 0.806 |
| ITGAL | P20701 | Inflammation II | 0.92(0.68,1.24) | 5.96E-01 | 0.806 |
| NPPC | P23582 | Inflammation | 1.06(0.85,1.34) | 5.97E-01 | 0.806 |
| IL1RN | P18510 | Inflammation | 0.95(0.78,1.16) | 6.10E-01 | 0.821 |
| RPA2 | P15927 | Inflammation II | 1.11(0.74,1.69) | 6.10E-01 | 0.821 |
| TLR1 | Q15399 | Inflammation II | 1.14(0.69,1.87) | 6.11E-01 | 0.821 |
| PGLYRP2 | Q96PD5 | Inflammation II | 0.89(0.58,1.38) | 6.12E-01 | 0.821 |
| CCNE1 | P24864 | Inflammation II | 0.96(0.82,1.13) | 6.14E-01 | 0.822 |
| PAPPA | Q13219 | Inflammation | 1.07(0.83,1.37) | 6.16E-01 | 0.822 |
| CD200R1 | Q8TD46 | Inflammation | 0.92(0.67,1.27) | 6.18E-01 | 0.822 |
| SIT1 | Q9Y3P8 | Inflammation | 1.05(0.86,1.29) | 6.18E-01 | 0.822 |
| PZP | P20742 | Inflammation II | 0.94(0.73,1.21) | 6.22E-01 | 0.824 |
| MERTK | Q12866 | Inflammation | 1.12(0.72,1.74) | 6.23E-01 | 0.824 |
| LHPP | Q9H008 | Inflammation | 1.05(0.87,1.26) | 6.25E-01 | 0.824 |
| GLI2 | P10070 | Inflammation II | 0.90(0.58,1.39) | 6.25E-01 | 0.824 |
| PKLR | P30613 | Inflammation | 1.04(0.88,1.23) | 6.27E-01 | 0.824 |
| BAG4 | O95429 | Inflammation II | 0.93(0.70,1.24) | 6.27E-01 | 0.824 |
| LPA | P08519 | Inflammation II | 1.02(0.95,1.10) | 6.27E-01 | 0.824 |
| MRC1 | P22897 | Inflammation II | 1.11(0.72,1.73) | 6.30E-01 | 0.826 |
| MKI67 | P46013 | Inflammation II | 1.08(0.78,1.49) | 6.38E-01 | 0.835 |
| TNFRSF17 | Q02223 | Inflammation II | 1.08(0.79,1.48) | 6.40E-01 | 0.836 |
| LTO1 | Q8WV07 | Inflammation | 0.93(0.70,1.24) | 6.42E-01 | 0.837 |
| TNFRSF13B | O14836 | Inflammation | 1.07(0.79,1.46) | 6.44E-01 | 0.839 |
| GMPR | P36959 | Inflammation | 0.95(0.76,1.19) | 6.51E-01 | 0.845 |
| PTH1R | Q03431 | Inflammation | 0.93(0.69,1.27) | 6.51E-01 | 0.845 |
| CEMIP2 | Q9UHN6 | Inflammation II | 0.91(0.59,1.39) | 6.53E-01 | 0.846 |
| C5 | P01031 | Inflammation II | 1.21(0.52,2.85) | 6.56E-01 | 0.847 |
| SAA4 | P35542 | Inflammation II | 1.09(0.75,1.59) | 6.56E-01 | 0.847 |
| CD6 | P30203 | Inflammation | 0.95(0.77,1.18) | 6.58E-01 | 0.847 |
| HEXIM1 | O94992 | Inflammation | 0.97(0.84,1.11) | 6.60E-01 | 0.847 |
| SIRT1 | Q96EB6 | Inflammation II | 0.96(0.81,1.14) | 6.60E-01 | 0.847 |
| IGFL4 | Q6B9Z1 | Inflammation II | 0.96(0.79,1.16) | 6.61E-01 | 0.847 |
| CRLF1 | O75462 | Inflammation | 1.10(0.71,1.71) | 6.67E-01 | 0.849 |
| CSF1R | P07333 | Inflammation II | 0.93(0.67,1.29) | 6.68E-01 | 0.849 |
| AGRN | O00468 | Inflammation | 0.93(0.67,1.29) | 6.69E-01 | 0.849 |
| PNPT1 | Q8TCS8 | Inflammation | 0.98(0.88,1.09) | 6.69E-01 | 0.849 |
| A1BG | P04217 | Inflammation II | 1.18(0.55,2.53) | 6.70E-01 | 0.849 |
| IL20RB | Q6UXL0 | Inflammation II | 0.93(0.67,1.29) | 6.70E-01 | 0.849 |
| CD4 | P01730 | Inflammation | 0.92(0.64,1.34) | 6.71E-01 | 0.849 |
| POF1B | Q8WVV4 | Inflammation II | 0.94(0.69,1.27) | 6.73E-01 | 0.850 |
| SOD3 | P08294 | Inflammation II | 1.04(0.85,1.28) | 6.76E-01 | 0.853 |
| ESR1 | P03372 | Inflammation II | 0.95(0.74,1.22) | 6.80E-01 | 0.854 |
| CNTNAP2 | Q9UHC6 | Inflammation | 0.94(0.72,1.25) | 6.82E-01 | 0.854 |
| VEGFD | O43915 | Inflammation | 1.07(0.78,1.45) | 6.82E-01 | 0.854 |
| IL12RB2 | Q99665 | Inflammation II | 0.93(0.67,1.30) | 6.82E-01 | 0.854 |
| NFAT5 | O94916 | Inflammation II | 0.97(0.83,1.13) | 6.87E-01 | 0.859 |
| IL1B | P01584 | Inflammation | 0.96(0.78,1.18) | 6.92E-01 | 0.862 |
| CD3G | P09693 | Inflammation II | 1.03(0.89,1.19) | 6.92E-01 | 0.862 |
| CD244 | Q9BZW8 | Inflammation | 1.08(0.72,1.63) | 6.97E-01 | 0.862 |
| IL18R1 | Q13478 | Inflammation | 0.94(0.67,1.31) | 6.97E-01 | 0.862 |
| TYRP1 | P17643 | Inflammation II | 1.04(0.84,1.29) | 6.97E-01 | 0.862 |
| FCRL3 | Q96P31 | Inflammation | 1.04(0.85,1.28) | 6.98E-01 | 0.862 |
| PSMG3 | Q9BT73 | Inflammation | 1.04(0.84,1.29) | 6.98E-01 | 0.862 |
| CD72 | P21854 | Inflammation II | 1.06(0.78,1.44) | 7.00E-01 | 0.863 |
| CTRC | Q99895 | Inflammation | 1.03(0.87,1.23) | 7.02E-01 | 0.864 |
| C1QTNF5 | Q9BXJ0 | Inflammation II | 0.93(0.65,1.33) | 7.06E-01 | 0.868 |
| PON1 | P27169 | Inflammation II | 0.91(0.56,1.48) | 7.13E-01 | 0.875 |
| PRKAB1 | Q9Y478 | Inflammation | 0.96(0.77,1.19) | 7.16E-01 | 0.877 |
| MLN | P12872 | Inflammation | 0.98(0.86,1.11) | 7.17E-01 | 0.877 |
| CHAD | O15335 | Inflammation II | 0.95(0.73,1.24) | 7.19E-01 | 0.878 |
| STX8 | Q9UNK0 | Inflammation | 0.96(0.76,1.21) | 7.26E-01 | 0.883 |
| COL5A1 | P20908 | Inflammation II | 1.06(0.76,1.48) | 7.26E-01 | 0.883 |
| DNER | Q8NFT8 | Inflammation | 1.08(0.70,1.67) | 7.30E-01 | 0.887 |
| NRTN | Q99748 | Inflammation | 1.04(0.83,1.30) | 7.32E-01 | 0.888 |
| EPHA1 | P21709 | Inflammation | 0.94(0.67,1.32) | 7.34E-01 | 0.888 |
| CA8 | P35219 | Inflammation II | 0.91(0.53,1.56) | 7.35E-01 | 0.888 |
| ADGRE2 | Q9UHX3 | Inflammation | 1.07(0.72,1.60) | 7.38E-01 | 0.891 |
| SLAMF1 | Q13291 | Inflammation | 1.03(0.85,1.25) | 7.44E-01 | 0.896 |
| ABO | P16442 | Inflammation II | 1.01(0.93,1.11) | 7.48E-01 | 0.899 |
| SLAMF7 | Q9NQ25 | Inflammation | 0.97(0.80,1.18) | 7.49E-01 | 0.899 |
| CEBPA | P49715 | Inflammation II | 0.94(0.64,1.38) | 7.51E-01 | 0.900 |
| DAPK2 | Q9UIK4 | Inflammation II | 1.04(0.82,1.33) | 7.53E-01 | 0.901 |
| FGF3 | P11487 | Inflammation II | 0.93(0.60,1.45) | 7.56E-01 | 0.903 |
| BLNK | Q8WV28 | Inflammation II | 0.95(0.66,1.35) | 7.57E-01 | 0.903 |
| PNLIPRP2 | P54317 | Inflammation | 0.99(0.96,1.03) | 7.60E-01 | 0.904 |
| SCRN1 | Q12765 | Inflammation | 1.03(0.84,1.27) | 7.60E-01 | 0.904 |
| ADAM23 | O75077 | Inflammation | 1.05(0.76,1.46) | 7.62E-01 | 0.904 |
| TNFSF13 | O75888 | Inflammation | 1.07(0.70,1.62) | 7.66E-01 | 0.906 |
| TOP2B | Q02880 | Inflammation II | 0.97(0.82,1.15) | 7.66E-01 | 0.906 |
| TBC1D5 | Q92609 | Inflammation | 1.02(0.89,1.17) | 7.67E-01 | 0.906 |
| CCL28 | Q9NRJ3 | Inflammation | 1.03(0.85,1.24) | 7.73E-01 | 0.911 |
| RAB37 | Q96AX2 | Inflammation | 0.98(0.85,1.13) | 7.76E-01 | 0.914 |
| GALNT3 | Q14435 | Inflammation | 1.04(0.79,1.36) | 7.83E-01 | 0.920 |
| THSD1 | Q9NS62 | Inflammation II | 1.06(0.71,1.58) | 7.88E-01 | 0.925 |
| KRT19 | P08727 | Inflammation | 1.02(0.86,1.21) | 7.90E-01 | 0.925 |
| CCL20 | P78556 | Inflammation | 1.02(0.90,1.15) | 7.92E-01 | 0.925 |
| CRELD1 | Q96HD1 | Inflammation II | 0.95(0.67,1.35) | 7.92E-01 | 0.925 |
| CLEC4C | Q8WTT0 | Inflammation | 0.97(0.77,1.22) | 7.95E-01 | 0.927 |
| FOLH1 | Q04609 | Inflammation II | 0.98(0.86,1.12) | 7.96E-01 | 0.927 |
| C1RL | Q9NZP8 | Inflammation II | 1.09(0.57,2.10) | 7.99E-01 | 0.928 |
| KLRB1 | Q12918 | Inflammation | 1.04(0.78,1.38) | 8.02E-01 | 0.928 |
| DIPK2B | Q9H7Y0 | Inflammation II | 0.94(0.58,1.53) | 8.02E-01 | 0.928 |
| ST8SIA1 | Q92185 | Inflammation II | 1.03(0.79,1.35) | 8.03E-01 | 0.928 |
| ANXA1 | P04083 | Inflammation II | 1.04(0.76,1.43) | 8.05E-01 | 0.928 |
| CLU | P10909 | Inflammation II | 0.94(0.60,1.48) | 8.05E-01 | 0.928 |
| RELB | Q01201 | Inflammation II | 0.97(0.73,1.28) | 8.06E-01 | 0.928 |
| BNIP3L | O60238 | Inflammation II | 1.04(0.77,1.40) | 8.10E-01 | 0.931 |
| PLCB1 | Q9NQ66 | Inflammation II | 1.04(0.76,1.41) | 8.14E-01 | 0.934 |
| IL36A | Q9UHA7 | Inflammation II | 0.97(0.76,1.25) | 8.19E-01 | 0.938 |
| HDAC8 | Q9BY41 | Inflammation II | 0.97(0.77,1.23) | 8.22E-01 | 0.940 |
| ARNT | P27540 | Inflammation | 0.97(0.70,1.34) | 8.30E-01 | 0.946 |
| NECTIN1 | Q15223 | Inflammation II | 1.05(0.70,1.57) | 8.30E-01 | 0.946 |
| CRELD2 | Q6UXH1 | Inflammation | 1.02(0.82,1.27) | 8.34E-01 | 0.947 |
| EDNRB | P24530 | Inflammation II | 1.03(0.76,1.40) | 8.36E-01 | 0.947 |
| FCN1 | O00602 | Inflammation II | 1.02(0.81,1.30) | 8.37E-01 | 0.947 |
| EGLN1 | Q9GZT9 | Inflammation | 1.02(0.84,1.25) | 8.38E-01 | 0.947 |
| IL24 | Q13007 | Inflammation | 1.02(0.82,1.29) | 8.38E-01 | 0.947 |
| CAT | P04040 | Inflammation II | 1.03(0.81,1.30) | 8.39E-01 | 0.947 |
| SERPINF1 | P36955 | Inflammation II | 0.95(0.60,1.52) | 8.40E-01 | 0.947 |
| EPO | P01588 | Inflammation | 1.02(0.87,1.18) | 8.42E-01 | 0.947 |
| IL17F | Q96PD4 | Inflammation | 0.98(0.84,1.15) | 8.42E-01 | 0.947 |
| ITGB6 | P18564 | Inflammation | 1.04(0.71,1.50) | 8.54E-01 | 0.959 |
| DNPH1 | O43598 | Inflammation | 1.02(0.82,1.26) | 8.57E-01 | 0.960 |
| PSIP1 | O75475 | Inflammation | 0.98(0.83,1.17) | 8.59E-01 | 0.961 |
| CLEC4G | Q6UXB4 | Inflammation | 1.03(0.74,1.44) | 8.60E-01 | 0.961 |
| CCL11 | P51671 | Inflammation | 1.03(0.76,1.40) | 8.63E-01 | 0.961 |
| CCL7 | P80098 | Inflammation | 1.02(0.84,1.22) | 8.64E-01 | 0.961 |
| CD70 | P32970 | Inflammation | 0.98(0.75,1.27) | 8.66E-01 | 0.961 |
| GSR | P00390 | Inflammation II | 0.95(0.52,1.72) | 8.66E-01 | 0.961 |
| PALLD | Q8WX93 | Inflammation II | 0.97(0.69,1.36) | 8.68E-01 | 0.961 |
| NTF3 | P20783 | Inflammation | 0.97(0.71,1.34) | 8.69E-01 | 0.961 |
| PIK3AP1 | Q6ZUJ8 | Inflammation | 1.02(0.85,1.21) | 8.70E-01 | 0.961 |
| ROBO1 | Q9Y6N7 | Inflammation | 0.96(0.60,1.54) | 8.71E-01 | 0.961 |
| NBN | O60934 | Inflammation | 0.98(0.79,1.22) | 8.73E-01 | 0.962 |
| C1R | P00736 | Inflammation II | 1.05(0.54,2.06) | 8.77E-01 | 0.965 |
| FUOM | A2VDF0 | Inflammation II | 0.98(0.80,1.21) | 8.80E-01 | 0.965 |
| CPOX | P36551 | Inflammation II | 0.96(0.58,1.60) | 8.81E-01 | 0.965 |
| REPS1 | Q96D71 | Inflammation II | 0.97(0.68,1.39) | 8.81E-01 | 0.965 |
| CRISP3 | P54108 | Inflammation II | 0.96(0.59,1.56) | 8.82E-01 | 0.965 |
| MXRA8 | Q9BRK3 | Inflammation II | 1.03(0.68,1.56) | 8.86E-01 | 0.967 |
| CCL22 | O00626 | Inflammation | 1.02(0.82,1.26) | 8.87E-01 | 0.967 |
| PRR5 | P85299 | Inflammation II | 1.02(0.76,1.37) | 8.89E-01 | 0.967 |
| YY1 | P25490 | Inflammation II | 1.03(0.64,1.66) | 8.90E-01 | 0.967 |
| BTN3A2 | P78410 | Inflammation | 0.98(0.73,1.32) | 8.91E-01 | 0.967 |
| NCF2 | P19878 | Inflammation | 1.01(0.88,1.15) | 8.92E-01 | 0.967 |
| PNLIP | P16233 | Inflammation II | 0.99(0.80,1.22) | 8.95E-01 | 0.969 |
| IL31 | Q6EBC2 | Inflammation II | 1.02(0.80,1.29) | 8.97E-01 | 0.969 |
| APOA4 | P06727 | Inflammation II | 1.02(0.71,1.47) | 9.03E-01 | 0.972 |
| CTSE | P14091 | Inflammation II | 0.98(0.76,1.27) | 9.04E-01 | 0.972 |
| IL16 | Q14005 | Inflammation | 0.98(0.76,1.28) | 9.05E-01 | 0.972 |
| SIGLEC10 | Q96LC7 | Inflammation | 1.02(0.74,1.41) | 9.06E-01 | 0.972 |
| PRDX2 | P32119 | Inflammation II | 1.01(0.82,1.25) | 9.07E-01 | 0.972 |
| CLEC4D | Q8WXI8 | Inflammation | 1.01(0.85,1.21) | 9.08E-01 | 0.972 |
| NEDD4L | Q96PU5 | Inflammation II | 1.02(0.77,1.33) | 9.10E-01 | 0.973 |
| DGKZ | Q13574 | Inflammation | 0.98(0.71,1.37) | 9.15E-01 | 0.973 |
| IL5 | P05113 | Inflammation | 1.00(0.92,1.08) | 9.15E-01 | 0.973 |
| PRKCQ | Q04759 | Inflammation | 1.01(0.78,1.32) | 9.15E-01 | 0.973 |
| LILRB4 | Q8NHJ6 | Inflammation | 1.01(0.79,1.31) | 9.18E-01 | 0.973 |
| ENPP7 | Q6UWV6 | Inflammation | 1.01(0.88,1.16) | 9.20E-01 | 0.973 |
| CFD | P00746 | Inflammation II | 1.03(0.56,1.89) | 9.20E-01 | 0.973 |
| CRHBP | P24387 | Inflammation | 0.98(0.66,1.46) | 9.21E-01 | 0.973 |
| GIMAP7 | Q8NHV1 | Inflammation II | 1.02(0.67,1.55) | 9.21E-01 | 0.973 |
| UNC5D | Q6UXZ4 | Inflammation II | 1.02(0.62,1.69) | 9.25E-01 | 0.976 |
| PENK | P01210 | Inflammation II | 1.02(0.67,1.55) | 9.27E-01 | 0.976 |
| ACTN4 | O43707 | Inflammation | 1.02(0.72,1.44) | 9.28E-01 | 0.976 |
| ITM2A | O43736 | Inflammation | 0.99(0.82,1.19) | 9.29E-01 | 0.976 |
| LY75 | O60449 | Inflammation | 1.01(0.76,1.36) | 9.31E-01 | 0.977 |
| CXCL14 | O95715 | Inflammation | 0.99(0.81,1.21) | 9.33E-01 | 0.978 |
| ANGPTL2 | Q9UKU9 | Inflammation | 1.01(0.77,1.33) | 9.36E-01 | 0.979 |
| LAIR1 | Q6GTX8 | Inflammation | 0.99(0.74,1.32) | 9.40E-01 | 0.980 |
| MST1 | P26927 | Inflammation II | 0.99(0.81,1.21) | 9.40E-01 | 0.980 |
| IL2RB | P14784 | Inflammation | 1.01(0.75,1.37) | 9.41E-01 | 0.980 |
| ENPP5 | Q9UJA9 | Inflammation | 0.99(0.76,1.29) | 9.44E-01 | 0.981 |
| TRAF2 | Q12933 | Inflammation | 1.01(0.86,1.18) | 9.44E-01 | 0.981 |
| SERPINB8 | P50452 | Inflammation | 0.99(0.82,1.20) | 9.50E-01 | 0.984 |
| PPM1B | O75688 | Inflammation II | 1.01(0.81,1.25) | 9.50E-01 | 0.984 |
| SLITRK1 | Q96PX8 | Inflammation II | 1.01(0.68,1.51) | 9.53E-01 | 0.986 |
| LSP1 | P33241 | Inflammation | 1.01(0.76,1.33) | 9.56E-01 | 0.986 |
| MZB1 | Q8WU39 | Inflammation | 0.99(0.80,1.23) | 9.57E-01 | 0.986 |
| MYO9B | Q13459 | Inflammation | 1.00(0.84,1.18) | 9.58E-01 | 0.986 |
| ZBP1 | Q9H171 | Inflammation II | 0.99(0.70,1.40) | 9.59E-01 | 0.986 |
| CFHR4 | Q92496 | Inflammation II | 1.00(0.84,1.20) | 9.60E-01 | 0.986 |
| COL9A1 | P20849 | Inflammation | 1.00(0.79,1.25) | 9.67E-01 | 0.988 |
| EP300 | Q09472 | Inflammation II | 0.99(0.65,1.52) | 9.67E-01 | 0.988 |
| ICAM4 | Q14773 | Inflammation | 1.00(0.81,1.24) | 9.69E-01 | 0.988 |
| NFASC | O94856 | Inflammation | 0.99(0.67,1.47) | 9.69E-01 | 0.988 |
| CSH1 | P0DML2 | Inflammation II | 1.01(0.64,1.60) | 9.69E-01 | 0.988 |
| NEDD9 | Q14511 | Inflammation II | 0.99(0.66,1.48) | 9.72E-01 | 0.990 |
| TPSG1 | Q9NRR2 | Inflammation II | 0.99(0.59,1.66) | 9.76E-01 | 0.991 |
| IL15RA | Q13261 | Inflammation | 1.00(0.72,1.39) | 9.79E-01 | 0.991 |
| SMPD3 | Q9NY59 | Inflammation II | 1.00(0.78,1.27) | 9.80E-01 | 0.991 |
| ENAH | Q8N8S7 | Inflammation | 1.00(0.78,1.27) | 9.81E-01 | 0.991 |
| DDI2 | Q5TDH0 | Inflammation II | 1.00(0.84,1.18) | 9.81E-01 | 0.991 |
| RNASE4 | P34096 | Inflammation II | 0.99(0.62,1.59) | 9.81E-01 | 0.991 |
| FCRL2 | Q96LA5 | Inflammation | 1.00(0.81,1.24) | 9.85E-01 | 0.993 |
| CCL17 | Q92583 | Inflammation | 1.00(0.88,1.13) | 9.88E-01 | 0.995 |
| PDZK1 | Q5T2W1 | Inflammation II | 1.00(0.74,1.36) | 9.90E-01 | 0.995 |
| LAP3 | P28838 | Inflammation | 1.00(0.79,1.27) | 9.91E-01 | 0.995 |
| RNF168 | Q8IYW5 | Inflammation II | 1.00(0.71,1.41) | 9.93E-01 | 0.996 |
| ORM1 | P02763 | Inflammation II | 1.00(0.52,1.91) | 9.95E-01 | 0.996 |
| HSD11B1 | P28845 | Inflammation | 1.00(0.75,1.32) | 9.97E-01 | 0.997 |

**^*^**Cox regression model adjusted for age and sex. *P* value was calculated under two-sided tests, and statistical significance was defined as a false discovery rate (FDR)-corrected *P* value <0.05, adjusted for the number of proteins tested (n=724).

**Table S8. Cox regression analysis for the associations between 726 proteins from the neurology panel and the risk of new-onset Alzheimer’s disease in the training set. ^*^**

| **Target Name** | **UniProt** | **Panel** | **HR (95%CI)** | ***P* value** | **FDR-corrected *P* value** |
| --- | --- | --- | --- | --- | --- |
| NEFL | P07196 | Neurology | 2.35(1.96,2.82) | 4.27E-20 | <0.001 |
| SNAP25 | P60880 | Neurology II | 1.92(1.48,2.50) | 9.63E-07 | <0.001 |
| SYT1 | P21579 | Neurology II | 1.53(1.28,1.82) | 2.04E-06 | <0.001 |
| BRK1 | Q8WUW1 | Neurology | 1.71(1.35,2.17) | 9.59E-06 | 0.002 |
| VGF | O15240 | Neurology II | 0.45(0.29,0.69) | 2.45E-04 | 0.036 |
| IL1R1 | P14778 | Neurology | 2.19(1.41,3.40) | 4.98E-04 | 0.056 |
| CEND1 | Q8N111 | Neurology II | 1.31(1.12,1.52) | 5.37E-04 | 0.056 |
| OGN | P20774 | Neurology | 1.51(1.18,1.94) | 1.06E-03 | 0.096 |
| PSG1 | P11464 | Neurology | 1.17(1.05,1.31) | 3.42E-03 | 0.219 |
| BCAN | Q96GW7 | Neurology | 0.62(0.46,0.86) | 3.50E-03 | 0.219 |
| PFDN2 | Q9UHV9 | Neurology | 0.73(0.59,0.90) | 3.60E-03 | 0.219 |
| CASC3 | O15234 | Neurology II | 0.59(0.42,0.84) | 3.62E-03 | 0.219 |
| DSC2 | Q02487 | Neurology | 1.57(1.16,2.14) | 3.92E-03 | 0.219 |
| TMCO5A | Q8N6Q1 | Neurology II | 0.66(0.50,0.88) | 4.30E-03 | 0.223 |
| MYLPF | Q96A32 | Neurology II | 1.36(1.09,1.69) | 5.78E-03 | 0.265 |
| SPINK5 | Q9NQ38 | Neurology | 1.57(1.14,2.17) | 5.85E-03 | 0.265 |
| CHCHD10 | Q8WYQ3 | Neurology II | 1.50(1.12,2.02) | 6.56E-03 | 0.278 |
| B4GAT1 | O43505 | Neurology | 0.53(0.33,0.84) | 6.90E-03 | 0.278 |
| TNFRSF8 | P28908 | Neurology | 1.36(1.08,1.72) | 8.85E-03 | 0.325 |
| TNFRSF1B | P20333 | Neurology | 1.37(1.08,1.75) | 9.27E-03 | 0.325 |
| TDO2 | P48775 | Neurology II | 0.58(0.38,0.88) | 1.13E-02 | 0.325 |
| SPOCK1 | Q08629 | Neurology | 0.57(0.37,0.88) | 1.20E-02 | 0.325 |
| CABP2 | Q9NPB3 | Neurology II | 0.74(0.58,0.94) | 1.21E-02 | 0.325 |
| CST5 | P28325 | Neurology | 0.77(0.62,0.94) | 1.23E-02 | 0.325 |
| CHM | P24386 | Neurology II | 0.75(0.60,0.94) | 1.43E-02 | 0.325 |
| CCN5 | O76076 | Neurology | 1.42(1.07,1.89) | 1.47E-02 | 0.325 |
| TNFRSF6B | O95407 | Neurology | 1.26(1.05,1.51) | 1.47E-02 | 0.325 |
| PCBP2 | Q15366 | Neurology II | 0.80(0.66,0.96) | 1.48E-02 | 0.325 |
| SCARB2 | Q14108 | Neurology | 1.49(1.08,2.06) | 1.58E-02 | 0.325 |
| VCAN | P13611 | Neurology | 1.57(1.09,2.26) | 1.58E-02 | 0.325 |
| MRPL58 | Q14197 | Neurology II | 0.76(0.61,0.95) | 1.62E-02 | 0.325 |
| GPC5 | P78333 | Neurology | 0.78(0.63,0.95) | 1.63E-02 | 0.325 |
| TNFRSF1A | P19438 | Neurology | 1.51(1.08,2.11) | 1.63E-02 | 0.325 |
| SCARF2 | Q96GP6 | Neurology | 1.60(1.09,2.34) | 1.65E-02 | 0.325 |
| ATXN2L | Q8WWM7 | Neurology II | 0.68(0.50,0.93) | 1.65E-02 | 0.325 |
| DSG2 | Q14126 | Neurology | 1.64(1.09,2.46) | 1.77E-02 | 0.325 |
| ACVRL1 | P37023 | Neurology | 1.66(1.09,2.52) | 1.79E-02 | 0.325 |
| CSNK2A1 | P68400 | Neurology II | 1.40(1.06,1.86) | 1.81E-02 | 0.325 |
| RBM17 | Q96I25 | Neurology II | 0.81(0.68,0.96) | 1.83E-02 | 0.325 |
| SPINT1 | O43278 | Neurology | 1.64(1.09,2.47) | 1.84E-02 | 0.325 |
| DDX53 | Q86TM3 | Neurology II | 0.63(0.43,0.92) | 1.85E-02 | 0.325 |
| MMP3 | P08254 | Neurology | 1.29(1.04,1.60) | 1.89E-02 | 0.325 |
| HHEX | Q03014 | Neurology II | 0.81(0.68,0.97) | 1.95E-02 | 0.325 |
| FRMD7 | Q6ZUT3 | Neurology II | 1.43(1.06,1.92) | 1.97E-02 | 0.325 |
| SYNGAP1 | Q96PV0 | Neurology II | 1.26(1.04,1.53) | 2.02E-02 | 0.326 |
| CX3CL1 | P78423 | Neurology | 1.47(1.06,2.04) | 2.11E-02 | 0.330 |
| RBFOX3 | A6NFN3 | Neurology II | 1.40(1.05,1.87) | 2.16E-02 | 0.330 |
| PRSS53 | Q2L4Q9 | Neurology II | 0.66(0.46,0.94) | 2.18E-02 | 0.330 |
| TIMP4 | Q99727 | Neurology | 1.36(1.04,1.77) | 2.36E-02 | 0.344 |
| HAVCR2 | Q8TDQ0 | Neurology | 1.41(1.05,1.90) | 2.37E-02 | 0.344 |
| FARSA | Q9Y285 | Neurology II | 0.78(0.63,0.97) | 2.70E-02 | 0.373 |
| MCEE | Q96PE7 | Neurology II | 0.66(0.46,0.96) | 2.79E-02 | 0.373 |
| RGMA | Q96B86 | Neurology | 1.48(1.04,2.11) | 2.81E-02 | 0.373 |
| NAA80 | Q93015 | Neurology II | 0.88(0.79,0.99) | 2.87E-02 | 0.373 |
| SUSD2 | Q9UGT4 | Neurology | 1.49(1.04,2.14) | 2.93E-02 | 0.373 |
| EDF1 | O60869 | Neurology II | 0.78(0.62,0.98) | 2.93E-02 | 0.373 |
| CLEC14A | Q86T13 | Neurology | 1.45(1.04,2.02) | 2.99E-02 | 0.373 |
| RGCC | Q9H4X1 | Neurology II | 0.83(0.70,0.98) | 3.08E-02 | 0.373 |
| PAMR1 | Q6UXH9 | Neurology | 0.64(0.43,0.96) | 3.11E-02 | 0.373 |
| GBP4 | Q96PP9 | Neurology | 0.76(0.59,0.98) | 3.16E-02 | 0.373 |
| TST | Q16762 | Neurology | 0.84(0.72,0.99) | 3.20E-02 | 0.373 |
| HDAC9 | Q9UKV0 | Neurology II | 0.75(0.57,0.98) | 3.21E-02 | 0.373 |
| EFNA4 | P52798 | Neurology | 1.43(1.03,1.98) | 3.24E-02 | 0.373 |
| SPINK1 | P00995 | Neurology | 1.31(1.02,1.68) | 3.43E-02 | 0.389 |
| CRTAM | O95727 | Neurology | 1.26(1.02,1.57) | 3.60E-02 | 0.402 |
| CRYBB2 | P43320 | Neurology II | 1.15(1.01,1.31) | 3.70E-02 | 0.407 |
| SLA2 | Q9H6Q3 | Neurology II | 0.88(0.77,0.99) | 3.76E-02 | 0.407 |
| CD99L2 | Q8TCZ2 | Neurology | 0.61(0.39,0.97) | 3.87E-02 | 0.413 |
| GTPBP2 | Q9BX10 | Neurology II | 0.84(0.71,0.99) | 4.01E-02 | 0.414 |
| SCN3B | Q9NY72 | Neurology II | 0.70(0.50,0.98) | 4.02E-02 | 0.414 |
| WFIKKN1 | Q96NZ8 | Neurology | 0.75(0.57,0.99) | 4.08E-02 | 0.414 |
| HJV | Q6ZVN8 | Neurology II | 0.71(0.51,0.99) | 4.11E-02 | 0.414 |
| RRAS | P10301 | Neurology II | 0.89(0.80,1.00) | 4.22E-02 | 0.420 |
| SMS | P52788 | Neurology II | 0.78(0.61,0.99) | 4.42E-02 | 0.434 |
| PDLIM5 | Q96HC4 | Neurology II | 0.90(0.80,1.00) | 4.61E-02 | 0.437 |
| MASP1 | P48740 | Neurology | 0.61(0.38,0.99) | 4.73E-02 | 0.437 |
| ARHGEF10 | O15013 | Neurology II | 0.87(0.75,1.00) | 4.80E-02 | 0.437 |
| TIMM8A | O60220 | Neurology II | 0.86(0.73,1.00) | 4.88E-02 | 0.437 |
| LAYN | Q6UX15 | Neurology | 1.37(1.00,1.86) | 4.92E-02 | 0.437 |
| PIBF1 | Q8WXW3-4 | Neurology II | 0.84(0.71,1.00) | 5.02E-02 | 0.437 |
| F11R | Q9Y624 | Neurology | 0.81(0.66,1.00) | 5.05E-02 | 0.437 |
| SV2A | Q7L0J3 | Neurology II | 0.79(0.62,1.00) | 5.08E-02 | 0.437 |
| ASAH2 | Q9NR71 | Neurology | 0.83(0.68,1.00) | 5.19E-02 | 0.437 |
| ODAM | A1E959 | Neurology | 0.80(0.64,1.00) | 5.21E-02 | 0.437 |
| NXPH1 | P58417 | Neurology | 0.73(0.54,1.00) | 5.27E-02 | 0.437 |
| RTN4IP1 | Q8WWV3 | Neurology II | 0.87(0.75,1.00) | 5.28E-02 | 0.437 |
| DCC | P43146 | Neurology II | 0.50(0.25,1.01) | 5.29E-02 | 0.437 |
| NGF | P01138 | Neurology | 0.40(0.16,1.01) | 5.30E-02 | 0.437 |
| GHRHR | Q02643 | Neurology | 1.18(1.00,1.39) | 5.43E-02 | 0.443 |
| DTX2 | Q86UW9 | Neurology II | 0.78(0.60,1.01) | 5.88E-02 | 0.461 |
| ALDH5A1 | P51649 | Neurology II | 0.75(0.55,1.01) | 5.89E-02 | 0.461 |
| DDHD2 | O94830 | Neurology II | 0.83(0.68,1.01) | 5.97E-02 | 0.461 |
| FCER2 | P06734 | Neurology | 1.20(0.99,1.44) | 6.01E-02 | 0.461 |
| TSC22D1 | Q15714 | Neurology II | 0.82(0.66,1.01) | 6.02E-02 | 0.461 |
| CLPS | P04118 | Neurology | 1.16(0.99,1.36) | 6.19E-02 | 0.461 |
| EFHD1 | Q9BUP0 | Neurology II | 1.40(0.98,2.00) | 6.22E-02 | 0.461 |
| BAX | Q07812 | Neurology | 0.88(0.77,1.01) | 6.23E-02 | 0.461 |
| NFIC | P08651 | Neurology II | 0.68(0.45,1.02) | 6.33E-02 | 0.461 |
| MINK1 | Q8N4C8 | Neurology II | 0.88(0.76,1.01) | 6.35E-02 | 0.461 |
| CAMLG | P49069 | Neurology II | 0.77(0.59,1.02) | 6.41E-02 | 0.461 |
| NRXN3 | Q9Y4C0 | Neurology II | 0.65(0.42,1.03) | 6.51E-02 | 0.461 |
| PVR | P15151 | Neurology | 1.32(0.98,1.79) | 6.55E-02 | 0.461 |
| TNFRSF21 | O75509 | Neurology | 1.46(0.98,2.17) | 6.55E-02 | 0.461 |
| CLSPN | Q9HAW4 | Neurology | 0.69(0.46,1.03) | 6.64E-02 | 0.461 |
| CORO6 | Q6QEF8 | Neurology II | 1.36(0.98,1.88) | 6.67E-02 | 0.461 |
| BLOC1S3 | Q6QNY0 | Neurology II | 0.72(0.51,1.02) | 6.74E-02 | 0.461 |
| AKT2 | P31751 | Neurology II | 0.91(0.82,1.01) | 6.85E-02 | 0.461 |
| TXNL1 | O43396 | Neurology II | 0.86(0.73,1.01) | 6.86E-02 | 0.461 |
| CNTN5 | O94779 | Neurology | 0.76(0.57,1.02) | 7.13E-02 | 0.467 |
| IGFBP4 | P22692 | Neurology | 1.24(0.98,1.58) | 7.32E-02 | 0.467 |
| AIDA | Q96BJ3 | Neurology II | 0.85(0.70,1.02) | 7.47E-02 | 0.467 |
| FZD8 | Q9H461 | Neurology II | 0.72(0.50,1.03) | 7.48E-02 | 0.467 |
| ERC2 | O15083 | Neurology II | 0.83(0.67,1.02) | 7.49E-02 | 0.467 |
| HTR1A | P08908 | Neurology II | 0.82(0.67,1.02) | 7.52E-02 | 0.467 |
| PFDN6 | O15212 | Neurology II | 0.79(0.61,1.02) | 7.52E-02 | 0.467 |
| CDK5RAP3 | Q96JB5 | Neurology II | 0.80(0.63,1.02) | 7.58E-02 | 0.467 |
| LMOD1 | P29536 | Neurology II | 1.36(0.97,1.91) | 7.58E-02 | 0.467 |
| FYB1 | O15117 | Neurology | 0.90(0.79,1.01) | 7.59E-02 | 0.467 |
| SNAPIN | O95295 | Neurology II | 0.81(0.64,1.02) | 7.74E-02 | 0.472 |
| FKBP14 | Q9NWM8 | Neurology II | 0.89(0.78,1.01) | 7.88E-02 | 0.473 |
| MRI1 | Q9BV20 | Neurology II | 0.84(0.70,1.02) | 7.88E-02 | 0.473 |
| ARHGEF1 | Q92888 | Neurology II | 0.88(0.76,1.02) | 7.95E-02 | 0.473 |
| GCC1 | Q96CN9 | Neurology II | 0.90(0.80,1.01) | 8.02E-02 | 0.473 |
| BCL2L1 | Q07817 | Neurology II | 0.89(0.78,1.01) | 8.17E-02 | 0.478 |
| SOWAHA | Q2M3V2 | Neurology II | 1.20(0.98,1.48) | 8.37E-02 | 0.479 |
| IFT20 | Q8IY31 | Neurology II | 0.75(0.53,1.04) | 8.41E-02 | 0.479 |
| NARS1 | O43776 | Neurology II | 0.79(0.60,1.03) | 8.52E-02 | 0.479 |
| AP2B1 | P63010 | Neurology II | 0.77(0.57,1.04) | 8.62E-02 | 0.479 |
| PAK4 | O96013 | Neurology | 0.84(0.68,1.03) | 8.66E-02 | 0.479 |
| LDLRAP1 | Q5SW96 | Neurology II | 0.89(0.78,1.02) | 8.66E-02 | 0.479 |
| PIGR | P01833 | Neurology | 1.30(0.96,1.77) | 8.80E-02 | 0.479 |
| RGS10 | O43665 | Neurology II | 0.82(0.65,1.03) | 8.91E-02 | 0.479 |
| ART5 | Q96L15 | Neurology II | 0.80(0.61,1.04) | 8.92E-02 | 0.479 |
| SORBS1 | Q9BX66 | Neurology II | 1.26(0.96,1.65) | 8.94E-02 | 0.479 |
| S100A14 | Q9HCY8 | Neurology II | 0.80(0.62,1.04) | 9.05E-02 | 0.479 |
| IVD | P26440 | Neurology | 1.20(0.97,1.49) | 9.06E-02 | 0.479 |
| HLA-A | P04439 | Neurology II | 1.42(0.95,2.12) | 9.10E-02 | 0.479 |
| GGT5 | P36269 | Neurology | 1.54(0.93,2.54) | 9.25E-02 | 0.479 |
| DLG4 | P78352 | Neurology II | 0.85(0.70,1.03) | 9.27E-02 | 0.479 |
| NTRK3 | Q16288 | Neurology | 1.53(0.93,2.52) | 9.28E-02 | 0.479 |
| HEPACAM2 | A8MVW5 | Neurology II | 1.30(0.96,1.76) | 9.31E-02 | 0.479 |
| ELAC1 | Q9H777 | Neurology II | 0.88(0.75,1.02) | 9.49E-02 | 0.485 |
| KRT14 | P02533 | Neurology | 1.31(0.95,1.79) | 9.56E-02 | 0.485 |
| STC1 | P52823 | Neurology | 1.22(0.96,1.56) | 9.73E-02 | 0.491 |
| NDRG1 | Q92597 | Neurology | 1.14(0.98,1.34) | 9.92E-02 | 0.496 |
| FGD3 | Q5JSP0 | Neurology II | 0.87(0.74,1.03) | 9.97E-02 | 0.496 |
| OTUD7B | Q6GQQ9 | Neurology II | 0.84(0.67,1.04) | 1.02E-01 | 0.500 |
| STX3 | Q13277 | Neurology II | 0.72(0.48,1.07) | 1.02E-01 | 0.500 |
| LPO | P22079 | Neurology | 1.17(0.97,1.41) | 1.04E-01 | 0.507 |
| LRTM2 | Q8N967 | Neurology II | 0.65(0.39,1.09) | 1.06E-01 | 0.513 |
| REEP4 | Q9H6H4 | Neurology II | 0.83(0.67,1.04) | 1.08E-01 | 0.518 |
| RAB3GAP1 | Q15042 | Neurology II | 0.79(0.60,1.05) | 1.09E-01 | 0.518 |
| VSIG4 | Q9Y279 | Neurology | 1.23(0.95,1.60) | 1.11E-01 | 0.518 |
| GAST | P01350 | Neurology II | 1.07(0.98,1.17) | 1.11E-01 | 0.518 |
| NOS1 | P29475 | Neurology | 1.18(0.96,1.45) | 1.13E-01 | 0.518 |
| LCN15 | Q6UWW0 | Neurology II | 1.17(0.96,1.42) | 1.13E-01 | 0.518 |
| MDM1 | Q8TC05 | Neurology II | 0.70(0.45,1.09) | 1.14E-01 | 0.518 |
| OXT | P01178 | Neurology | 0.94(0.87,1.02) | 1.15E-01 | 0.518 |
| GLP1R | P43220 | Neurology II | 0.72(0.48,1.08) | 1.15E-01 | 0.518 |
| TXNDC9 | O14530 | Neurology II | 0.81(0.62,1.05) | 1.15E-01 | 0.518 |
| CCL2 | P13500 | Neurology | 0.84(0.68,1.04) | 1.16E-01 | 0.518 |
| STXBP1 | P61764 | Neurology II | 0.87(0.74,1.03) | 1.16E-01 | 0.518 |
| PTPRN2 | Q92932 | Neurology | 0.76(0.53,1.07) | 1.17E-01 | 0.518 |
| LYSMD3 | Q7Z3D4 | Neurology II | 0.89(0.77,1.03) | 1.17E-01 | 0.518 |
| PLEKHO1 | Q53GL0 | Neurology II | 0.86(0.71,1.04) | 1.18E-01 | 0.519 |
| DOC2B | Q14184 | Neurology II | 1.28(0.94,1.75) | 1.19E-01 | 0.520 |
| SUOX | P51687 | Neurology II | 0.84(0.67,1.05) | 1.21E-01 | 0.526 |
| PIK3IP1 | Q96FE7 | Neurology | 1.35(0.92,1.99) | 1.23E-01 | 0.530 |
| GDNF | P39905 | Neurology | 1.26(0.94,1.69) | 1.24E-01 | 0.530 |
| MFGE8 | Q08431 | Neurology | 0.81(0.62,1.06) | 1.24E-01 | 0.530 |
| IFNL1 | Q8IU54 | Neurology | 1.21(0.95,1.55) | 1.26E-01 | 0.535 |
| CD300C | Q08708 | Neurology | 1.27(0.93,1.74) | 1.30E-01 | 0.548 |
| GLB1 | P16278 | Neurology | 0.80(0.60,1.07) | 1.31E-01 | 0.548 |
| GNLY | P22749 | Neurology | 1.17(0.95,1.44) | 1.32E-01 | 0.548 |
| RASA1 | P20936 | Neurology | 0.76(0.53,1.09) | 1.32E-01 | 0.548 |
| MYOC | Q99972 | Neurology | 1.21(0.94,1.56) | 1.35E-01 | 0.553 |
| IMPG1 | Q17R60 | Neurology II | 0.69(0.42,1.13) | 1.35E-01 | 0.553 |
| EREG | O14944 | Neurology | 0.89(0.77,1.04) | 1.36E-01 | 0.553 |
| GUCA2A | Q02747 | Neurology | 1.28(0.92,1.77) | 1.37E-01 | 0.553 |
| GNAS | O95467 | Neurology II | 0.88(0.74,1.04) | 1.37E-01 | 0.553 |
| PBLD | P30039 | Neurology | 0.84(0.66,1.06) | 1.38E-01 | 0.554 |
| AFP | P02771 | Neurology | 0.88(0.74,1.04) | 1.39E-01 | 0.554 |
| SPAG1 | Q07617 | Neurology II | 0.83(0.65,1.07) | 1.43E-01 | 0.558 |
| IMPA1 | P29218 | Neurology | 0.87(0.71,1.05) | 1.45E-01 | 0.558 |
| BOLA1 | Q9Y3E2 | Neurology II | 0.82(0.62,1.07) | 1.46E-01 | 0.558 |
| CTRL | P40313 | Neurology II | 1.15(0.95,1.39) | 1.46E-01 | 0.558 |
| DHRS4L2 | Q6PKH6 | Neurology II | 0.79(0.58,1.08) | 1.46E-01 | 0.558 |
| BMP4 | P12644 | Neurology | 1.19(0.94,1.50) | 1.47E-01 | 0.558 |
| DNLZ | Q5SXM8 | Neurology II | 0.88(0.74,1.05) | 1.48E-01 | 0.558 |
| PAFAH1B3 | Q15102 | Neurology II | 0.91(0.80,1.03) | 1.48E-01 | 0.558 |
| NCAM2 | O15394 | Neurology | 1.34(0.90,1.98) | 1.50E-01 | 0.558 |
| TTF2 | Q9UNY4 | Neurology II | 0.81(0.61,1.08) | 1.50E-01 | 0.558 |
| ROBO2 | Q9HCK4 | Neurology | 0.70(0.42,1.14) | 1.51E-01 | 0.558 |
| TJP3 | O95049 | Neurology II | 0.77(0.53,1.10) | 1.51E-01 | 0.558 |
| NMT1 | P30419 | Neurology II | 0.85(0.69,1.06) | 1.52E-01 | 0.558 |
| FKBP4 | Q02790 | Neurology | 0.83(0.64,1.07) | 1.53E-01 | 0.558 |
| GNGT1 | P63211 | Neurology II | 0.79(0.57,1.09) | 1.53E-01 | 0.558 |
| YAP1 | P46937 | Neurology II | 1.38(0.89,2.14) | 1.54E-01 | 0.558 |
| NCAN | O14594 | Neurology | 1.23(0.92,1.64) | 1.55E-01 | 0.558 |
| FLRT2 | O43155 | Neurology | 1.33(0.90,1.98) | 1.56E-01 | 0.558 |
| PHACTR2 | O75167 | Neurology II | 0.92(0.82,1.03) | 1.56E-01 | 0.558 |
| CLPP | Q16740 | Neurology | 0.92(0.81,1.03) | 1.58E-01 | 0.558 |
| DNM3 | Q9UQ16 | Neurology II | 0.83(0.64,1.07) | 1.58E-01 | 0.558 |
| FCRL5 | Q96RD9 | Neurology | 0.87(0.72,1.06) | 1.59E-01 | 0.558 |
| HTR1B | P28222 | Neurology II | 0.78(0.55,1.10) | 1.59E-01 | 0.558 |
| LELP1 | Q5T871 | Neurology II | 0.86(0.70,1.06) | 1.59E-01 | 0.558 |
| MYL6B | P14649 | Neurology II | 0.89(0.76,1.05) | 1.59E-01 | 0.558 |
| ADGRB3 | O60242 | Neurology | 1.27(0.91,1.77) | 1.60E-01 | 0.558 |
| SIGLEC15 | Q6ZMC9 | Neurology | 1.10(0.96,1.27) | 1.62E-01 | 0.558 |
| BST2 | Q10589 | Neurology | 1.16(0.94,1.43) | 1.64E-01 | 0.558 |
| HARS1 | P12081 | Neurology | 0.90(0.78,1.04) | 1.64E-01 | 0.558 |
| CACNB3 | P54284 | Neurology II | 0.89(0.77,1.05) | 1.64E-01 | 0.558 |
| RLN1 | P04808 | Neurology II | 1.17(0.94,1.45) | 1.65E-01 | 0.558 |
| MITD1 | Q8WV92 | Neurology | 0.92(0.82,1.03) | 1.66E-01 | 0.558 |
| TNFRSF10A | O00220 | Neurology | 1.23(0.92,1.66) | 1.66E-01 | 0.558 |
| DOK1 | Q99704 | Neurology II | 0.94(0.85,1.03) | 1.66E-01 | 0.558 |
| DLGAP5 | Q15398 | Neurology II | 0.80(0.58,1.10) | 1.67E-01 | 0.559 |
| AK2 | P54819 | Neurology II | 0.92(0.81,1.04) | 1.69E-01 | 0.563 |
| TNFRSF10B | O14763 | Neurology | 1.16(0.94,1.44) | 1.71E-01 | 0.567 |
| THBS2 | P35442 | Neurology | 1.22(0.92,1.64) | 1.72E-01 | 0.567 |
| PRTFDC1 | Q9NRG1 | Neurology | 0.93(0.84,1.03) | 1.73E-01 | 0.567 |
| CLEC1B | Q9P126 | Neurology | 0.92(0.82,1.04) | 1.75E-01 | 0.567 |
| KIAA0319 | Q5VV43 | Neurology II | 1.31(0.89,1.93) | 1.78E-01 | 0.567 |
| CDH3 | P22223 | Neurology | 1.23(0.91,1.68) | 1.79E-01 | 0.567 |
| EPHB6 | O15197 | Neurology | 1.27(0.90,1.81) | 1.79E-01 | 0.567 |
| DYNLT1 | P63172 | Neurology II | 0.88(0.74,1.06) | 1.79E-01 | 0.567 |
| MAX | P61244 | Neurology | 0.93(0.83,1.04) | 1.80E-01 | 0.567 |
| VSNL1 | P62760 | Neurology II | 1.24(0.90,1.70) | 1.80E-01 | 0.567 |
| CDHR1 | Q96JP9 | Neurology | 0.82(0.61,1.10) | 1.81E-01 | 0.567 |
| MAP4K5 | Q9Y4K4 | Neurology | 0.93(0.84,1.03) | 1.81E-01 | 0.567 |
| ILKAP | Q9H0C8 | Neurology | 0.87(0.71,1.07) | 1.82E-01 | 0.567 |
| TNFRSF9 | Q07011 | Neurology | 1.18(0.93,1.49) | 1.82E-01 | 0.567 |
| CDH4 | P55283 | Neurology II | 0.81(0.60,1.10) | 1.82E-01 | 0.567 |
| PECAM1 | P16284 | Neurology | 0.77(0.53,1.13) | 1.83E-01 | 0.568 |
| MRPL28 | Q13084 | Neurology II | 0.72(0.44,1.17) | 1.87E-01 | 0.578 |
| PNMA2 | Q9UL42 | Neurology II | 0.81(0.59,1.11) | 1.88E-01 | 0.578 |
| HSD17B14 | Q9BPX1 | Neurology II | 0.87(0.70,1.07) | 1.92E-01 | 0.587 |
| LMNB2 | Q03252 | Neurology II | 1.35(0.86,2.13) | 1.93E-01 | 0.587 |
| TNIP1 | Q15025 | Neurology II | 0.91(0.78,1.05) | 1.94E-01 | 0.587 |
| VAV3 | Q9UKW4 | Neurology II | 0.91(0.80,1.05) | 1.94E-01 | 0.587 |
| DNAJA4 | Q8WW22 | Neurology II | 1.12(0.94,1.34) | 1.96E-01 | 0.590 |
| CADPS | Q9ULU8 | Neurology II | 0.78(0.54,1.14) | 1.98E-01 | 0.592 |
| CHRM1 | P11229 | Neurology II | 0.77(0.51,1.15) | 1.98E-01 | 0.592 |
| GFRAL | Q6UXV0 | Neurology II | 1.14(0.93,1.40) | 1.99E-01 | 0.592 |
| CALCB | P10092 | Neurology II | 1.22(0.90,1.65) | 2.00E-01 | 0.593 |
| GOLGA3 | Q08378 | Neurology II | 0.89(0.75,1.06) | 2.03E-01 | 0.594 |
| KLHL41 | O60662 | Neurology II | 1.18(0.91,1.53) | 2.03E-01 | 0.594 |
| PPP1R14A | Q96A00 | Neurology II | 0.89(0.75,1.06) | 2.03E-01 | 0.594 |
| EBAG9 | O00559 | Neurology | 0.93(0.82,1.04) | 2.07E-01 | 0.597 |
| MICALL2 | Q8IY33 | Neurology II | 0.88(0.71,1.08) | 2.07E-01 | 0.597 |
| DEFB116 | Q30KQ4 | Neurology II | 0.88(0.72,1.07) | 2.08E-01 | 0.597 |
| LYPD1 | Q8N2G4 | Neurology | 1.11(0.94,1.30) | 2.09E-01 | 0.597 |
| MATN3 | O15232 | Neurology | 1.25(0.88,1.77) | 2.09E-01 | 0.597 |
| SPTBN2 | O15020 | Neurology II | 0.75(0.48,1.17) | 2.09E-01 | 0.597 |
| TMPRSS11D | O60235 | Neurology II | 1.23(0.89,1.71) | 2.10E-01 | 0.597 |
| CD274 | Q9NZQ7 | Neurology | 1.23(0.89,1.71) | 2.11E-01 | 0.597 |
| DKK4 | Q9UBT3 | Neurology | 1.17(0.92,1.49) | 2.12E-01 | 0.597 |
| NPTX1 | Q15818 | Neurology | 0.83(0.62,1.11) | 2.12E-01 | 0.597 |
| BATF | Q16520 | Neurology II | 0.89(0.74,1.07) | 2.13E-01 | 0.597 |
| ENO2 | P09104 | Neurology | 0.89(0.75,1.07) | 2.15E-01 | 0.600 |
| CRADD | P78560 | Neurology | 0.91(0.78,1.06) | 2.16E-01 | 0.601 |
| CD63 | P08962 | Neurology | 0.87(0.69,1.09) | 2.18E-01 | 0.602 |
| IGF2R | P11717 | Neurology | 1.35(0.84,2.19) | 2.18E-01 | 0.602 |
| EIF4G3 | O43432 | Neurology II | 0.88(0.72,1.08) | 2.19E-01 | 0.602 |
| MESD | Q14696 | Neurology | 0.94(0.86,1.04) | 2.20E-01 | 0.603 |
| ADAM8 | P78325 | Neurology | 1.26(0.87,1.84) | 2.23E-01 | 0.603 |
| PPCDC | Q96CD2 | Neurology | 0.86(0.67,1.10) | 2.23E-01 | 0.603 |
| PLXDC2 | Q6UX71 | Neurology II | 1.33(0.84,2.08) | 2.23E-01 | 0.603 |
| TSPAN7 | P41732 | Neurology II | 0.91(0.77,1.06) | 2.24E-01 | 0.603 |
| SSB | P05455 | Neurology | 1.12(0.93,1.35) | 2.25E-01 | 0.603 |
| PTH | P01270 | Neurology II | 0.90(0.76,1.07) | 2.26E-01 | 0.603 |
| ATP5PO | P48047 | Neurology | 1.18(0.90,1.53) | 2.29E-01 | 0.603 |
| CD74 | P04233 | Neurology | 1.21(0.89,1.65) | 2.31E-01 | 0.603 |
| TXLNA | P40222 | Neurology | 0.92(0.81,1.05) | 2.31E-01 | 0.603 |
| LAMP2 | P13473 | Neurology | 0.71(0.40,1.25) | 2.32E-01 | 0.603 |
| VWC2 | Q2TAL6 | Neurology | 1.19(0.90,1.57) | 2.32E-01 | 0.603 |
| APOH | P02749 | Neurology | 1.20(0.89,1.63) | 2.33E-01 | 0.603 |
| ECE1 | P42892 | Neurology | 0.82(0.60,1.13) | 2.33E-01 | 0.603 |
| DOCK9 | Q9BZ29 | Neurology II | 0.93(0.82,1.05) | 2.33E-01 | 0.603 |
| PLCB2 | Q00722 | Neurology II | 0.92(0.81,1.05) | 2.34E-01 | 0.603 |
| TNFSF14 | O43557 | Neurology | 0.88(0.72,1.08) | 2.35E-01 | 0.603 |
| KLF4 | O43474 | Neurology II | 0.81(0.57,1.15) | 2.35E-01 | 0.603 |
| TXNDC5 | Q8NBS9 | Neurology | 0.90(0.76,1.07) | 2.36E-01 | 0.603 |
| PRKAR2A | P13861 | Neurology II | 0.91(0.77,1.07) | 2.36E-01 | 0.603 |
| ASPSCR1 | Q9BZE9 | Neurology II | 0.89(0.73,1.08) | 2.38E-01 | 0.605 |
| MSR1 | P21757 | Neurology | 1.16(0.91,1.48) | 2.39E-01 | 0.605 |
| GPR101 | Q96P66 | Neurology II | 0.85(0.66,1.11) | 2.39E-01 | 0.605 |
| CLSTN3 | Q9BQT9 | Neurology II | 0.77(0.50,1.19) | 2.41E-01 | 0.608 |
| CD177 | Q8N6Q3 | Neurology | 0.95(0.88,1.03) | 2.45E-01 | 0.615 |
| SEZ6 | Q53EL9 | Neurology II | 1.28(0.84,1.95) | 2.49E-01 | 0.623 |
| INHBC | P55103 | Neurology | 0.86(0.67,1.11) | 2.50E-01 | 0.624 |
| EIF4B | P23588 | Neurology | 0.89(0.73,1.09) | 2.51E-01 | 0.624 |
| AP3S2 | P59780 | Neurology II | 0.85(0.64,1.13) | 2.54E-01 | 0.629 |
| NT5C1A | Q9BXI3 | Neurology II | 1.24(0.86,1.80) | 2.55E-01 | 0.630 |
| DRAXIN | Q8NBI3 | Neurology | 1.16(0.89,1.51) | 2.57E-01 | 0.631 |
| GIPC2 | Q8TF65 | Neurology II | 1.28(0.84,1.96) | 2.58E-01 | 0.631 |
| IMPACT | Q9P2X3 | Neurology II | 0.87(0.69,1.10) | 2.58E-01 | 0.631 |
| STK24 | Q9Y6E0 | Neurology | 0.90(0.74,1.09) | 2.62E-01 | 0.634 |
| DUSP3 | P51452 | Neurology | 0.92(0.79,1.07) | 2.63E-01 | 0.634 |
| TARBP2 | Q15633 | Neurology | 0.92(0.79,1.06) | 2.63E-01 | 0.634 |
| CRTAP | O75718 | Neurology II | 0.84(0.62,1.14) | 2.63E-01 | 0.634 |
| UPK3A | O75631 | Neurology II | 0.85(0.64,1.13) | 2.65E-01 | 0.637 |
| OPHN1 | O60890 | Neurology II | 0.93(0.83,1.05) | 2.66E-01 | 0.637 |
| SERPINB6 | P35237 | Neurology | 0.89(0.73,1.09) | 2.67E-01 | 0.638 |
| MAPT | P10636 | Neurology | 1.13(0.91,1.42) | 2.69E-01 | 0.640 |
| PHOSPHO1 | Q8TCT1 | Neurology | 1.24(0.85,1.82) | 2.70E-01 | 0.641 |
| CASP1 | P29466 | Neurology | 0.91(0.77,1.08) | 2.74E-01 | 0.644 |
| MMP9 | P14780 | Neurology | 1.11(0.92,1.34) | 2.74E-01 | 0.644 |
| DDR1 | Q08345 | Neurology | 1.32(0.80,2.16) | 2.75E-01 | 0.644 |
| WARS | P23381 | Neurology | 0.85(0.63,1.14) | 2.75E-01 | 0.644 |
| KCNIP4 | Q6PIL6 | Neurology | 1.12(0.91,1.37) | 2.78E-01 | 0.649 |
| SMPD1 | P17405 | Neurology | 0.88(0.70,1.11) | 2.80E-01 | 0.652 |
| BRSK2 | Q8IWQ3 | Neurology II | 0.82(0.57,1.18) | 2.81E-01 | 0.652 |
| PILRA | Q9UKJ1 | Neurology | 1.16(0.89,1.51) | 2.83E-01 | 0.652 |
| HNRNPUL1 | Q9BUJ2 | Neurology II | 0.86(0.65,1.14) | 2.83E-01 | 0.652 |
| GP6 | Q9HCN6 | Neurology | 0.93(0.82,1.06) | 2.88E-01 | 0.660 |
| CRYM | Q14894 | Neurology II | 0.90(0.74,1.09) | 2.88E-01 | 0.660 |
| ATP6V1F | Q16864 | Neurology | 0.91(0.76,1.09) | 2.89E-01 | 0.660 |
| CPM | P14384 | Neurology | 0.85(0.62,1.15) | 2.91E-01 | 0.660 |
| KLRK1 | P26718 | Neurology II | 1.16(0.88,1.52) | 2.91E-01 | 0.660 |
| TDRKH | Q9Y2W6 | Neurology | 0.93(0.81,1.07) | 2.92E-01 | 0.660 |
| MAD1L1 | Q9Y6D9 | Neurology | 1.12(0.90,1.40) | 2.93E-01 | 0.661 |
| DKK1 | O94907 | Neurology | 0.90(0.74,1.09) | 2.96E-01 | 0.661 |
| FMNL1 | O95466 | Neurology | 1.09(0.92,1.29) | 2.96E-01 | 0.661 |
| KEL | P23276 | Neurology | 1.18(0.86,1.62) | 2.97E-01 | 0.661 |
| RWDD1 | Q9H446 | Neurology | 0.91(0.76,1.09) | 2.97E-01 | 0.661 |
| CACNA1C | Q13936 | Neurology II | 0.79(0.51,1.24) | 3.03E-01 | 0.671 |
| APP | P05067 | Neurology | 0.91(0.77,1.09) | 3.04E-01 | 0.671 |
| MYOM1 | P52179 | Neurology II | 1.14(0.89,1.47) | 3.04E-01 | 0.671 |
| AGR2 | O95994 | Neurology | 1.06(0.95,1.19) | 3.08E-01 | 0.675 |
| ESYT2 | A0FGR8 | Neurology II | 0.92(0.78,1.08) | 3.10E-01 | 0.675 |
| DEFB104A_DEFB104B | Q8WTQ1 | Neurology II | 0.87(0.67,1.14) | 3.12E-01 | 0.675 |
| DSCAM | O60469 | Neurology II | 0.82(0.57,1.20) | 3.12E-01 | 0.675 |
| ANXA5 | P08758 | Neurology | 1.22(0.83,1.79) | 3.13E-01 | 0.675 |
| FTCD | O95954 | Neurology II | 0.92(0.79,1.08) | 3.13E-01 | 0.675 |
| FABP5 | Q01469 | Neurology | 0.92(0.78,1.09) | 3.14E-01 | 0.675 |
| CAMSAP1 | Q5T5Y3 | Neurology II | 0.94(0.83,1.06) | 3.14E-01 | 0.675 |
| RBP1 | P09455 | Neurology II | 0.90(0.72,1.11) | 3.15E-01 | 0.675 |
| TBR1 | Q16650 | Neurology II | 0.85(0.62,1.17) | 3.15E-01 | 0.675 |
| NRP2 | O60462 | Neurology | 1.22(0.83,1.80) | 3.17E-01 | 0.675 |
| PNLIPRP1 | P54315 | Neurology II | 1.09(0.92,1.29) | 3.17E-01 | 0.675 |
| PRND | Q9UKY0 | Neurology II | 1.14(0.88,1.47) | 3.20E-01 | 0.679 |
| EFNA1 | P20827 | Neurology | 1.22(0.83,1.79) | 3.21E-01 | 0.679 |
| PRKAG3 | Q9UGI9 | Neurology II | 0.80(0.51,1.25) | 3.23E-01 | 0.682 |
| DXO | O77932 | Neurology II | 0.86(0.64,1.16) | 3.27E-01 | 0.688 |
| CNTN3 | Q9P232 | Neurology | 0.84(0.59,1.19) | 3.32E-01 | 0.697 |
| TAX1BP1 | Q86VP1 | Neurology II | 0.90(0.73,1.11) | 3.33E-01 | 0.697 |
| CLEC2L | P0C7M8 | Neurology II | 0.87(0.65,1.16) | 3.34E-01 | 0.697 |
| NAPRT | Q6XQN6 | Neurology II | 0.82(0.54,1.23) | 3.35E-01 | 0.697 |
| TMPRSS11B | Q86T26 | Neurology II | 0.91(0.76,1.10) | 3.37E-01 | 0.699 |
| CHMP1A | Q9HD42 | Neurology | 0.93(0.81,1.08) | 3.40E-01 | 0.699 |
| IGBP1 | P78318 | Neurology II | 0.90(0.72,1.12) | 3.40E-01 | 0.699 |
| PSMD5 | Q16401 | Neurology II | 1.11(0.90,1.36) | 3.40E-01 | 0.699 |
| STX1B | P61266 | Neurology II | 1.15(0.86,1.53) | 3.43E-01 | 0.703 |
| BLOC1S2 | Q6QNY1 | Neurology II | 0.91(0.75,1.11) | 3.44E-01 | 0.704 |
| STIP1 | P31948 | Neurology | 0.92(0.77,1.09) | 3.49E-01 | 0.710 |
| PLSCR3 | Q9NRY6 | Neurology II | 0.90(0.72,1.13) | 3.49E-01 | 0.710 |
| CGN | Q9P2M7 | Neurology II | 0.84(0.58,1.21) | 3.52E-01 | 0.714 |
| NLGN1 | Q8N2Q7 | Neurology II | 0.90(0.73,1.12) | 3.55E-01 | 0.716 |
| SUMF2 | Q8NBJ7 | Neurology | 0.91(0.74,1.12) | 3.56E-01 | 0.716 |
| ZPR1 | O75312 | Neurology II | 1.06(0.93,1.21) | 3.56E-01 | 0.716 |
| TBCC | Q15814 | Neurology | 0.94(0.81,1.08) | 3.59E-01 | 0.720 |
| C2orf69 | Q8N8R5 | Neurology II | 0.89(0.69,1.15) | 3.60E-01 | 0.720 |
| CTRB1 | P17538 | Neurology | 1.10(0.90,1.34) | 3.62E-01 | 0.720 |
| TXNRD1 | Q16881 | Neurology | 0.89(0.69,1.14) | 3.62E-01 | 0.720 |
| DNAJC21 | Q5F1R6 | Neurology II | 0.85(0.60,1.21) | 3.64E-01 | 0.720 |
| SPRR3 | Q9UBC9 | Neurology II | 1.09(0.90,1.31) | 3.65E-01 | 0.720 |
| SNCG | O76070 | Neurology | 1.08(0.92,1.27) | 3.66E-01 | 0.720 |
| STAMBP | O95630 | Neurology | 0.92(0.77,1.10) | 3.66E-01 | 0.720 |
| ITGA5 | P08648 | Neurology | 0.81(0.52,1.27) | 3.67E-01 | 0.720 |
| CASP7 | P55210 | Neurology II | 0.93(0.79,1.09) | 3.68E-01 | 0.720 |
| AMFR | Q9UKV5 | Neurology | 1.09(0.91,1.30) | 3.71E-01 | 0.724 |
| SIGLEC5 | O15389 | Neurology | 1.05(0.95,1.16) | 3.72E-01 | 0.724 |
| JAM2 | P57087 | Neurology | 1.21(0.80,1.84) | 3.73E-01 | 0.724 |
| TBCB | Q99426 | Neurology | 0.95(0.85,1.06) | 3.75E-01 | 0.724 |
| NGRN | Q9NPE2 | Neurology II | 0.74(0.38,1.43) | 3.75E-01 | 0.724 |
| DIPK1C | Q0P6D2 | Neurology II | 0.85(0.60,1.21) | 3.78E-01 | 0.728 |
| LEO1 | Q8WVC0 | Neurology II | 0.81(0.51,1.30) | 3.80E-01 | 0.728 |
| PRDX1 | Q06830 | Neurology | 0.94(0.82,1.08) | 3.81E-01 | 0.728 |
| CLGN | O14967 | Neurology II | 0.87(0.64,1.19) | 3.82E-01 | 0.728 |
| RAC3 | P60763 | Neurology II | 0.91(0.73,1.13) | 3.86E-01 | 0.728 |
| ATXN10 | Q9UBB4 | Neurology | 0.93(0.80,1.09) | 3.87E-01 | 0.728 |
| DCUN1D1 | Q96GG9 | Neurology II | 0.92(0.76,1.11) | 3.88E-01 | 0.728 |
| CDKL5 | O76039 | Neurology II | 0.89(0.68,1.16) | 3.89E-01 | 0.728 |
| CTNNA1 | P35221 | Neurology II | 0.82(0.52,1.29) | 3.89E-01 | 0.728 |
| ARFIP1 | P53367 | Neurology II | 0.92(0.76,1.11) | 3.90E-01 | 0.728 |
| IGDCC3 | Q8IVU1 | Neurology II | 0.87(0.64,1.19) | 3.90E-01 | 0.728 |
| CCL19 | Q99731 | Neurology | 0.96(0.88,1.05) | 3.91E-01 | 0.728 |
| HDGFL2 | Q7Z4V5 | Neurology II | 0.91(0.74,1.13) | 3.91E-01 | 0.728 |
| NLGN2 | Q8NFZ4 | Neurology II | 1.12(0.87,1.45) | 3.91E-01 | 0.728 |
| FCER1A | P12319 | Neurology II | 1.26(0.74,2.16) | 3.93E-01 | 0.730 |
| MSLNL | Q96KJ4 | Neurology II | 0.85(0.58,1.24) | 3.94E-01 | 0.730 |
| CCS | O14618 | Neurology | 0.90(0.71,1.15) | 3.95E-01 | 0.730 |
| RANBP2 | P49792 | Neurology II | 0.83(0.54,1.27) | 3.96E-01 | 0.730 |
| MFAP3L | O75121 | Neurology II | 0.91(0.72,1.14) | 3.97E-01 | 0.730 |
| DUSP29 | Q68J44 | Neurology II | 1.08(0.90,1.30) | 3.99E-01 | 0.732 |
| KLB | Q86Z14 | Neurology | 1.07(0.92,1.25) | 4.01E-01 | 0.733 |
| CDH15 | P55291 | Neurology | 0.93(0.77,1.11) | 4.02E-01 | 0.733 |
| CC2D1A | Q6P1N0 | Neurology | 0.94(0.80,1.09) | 4.04E-01 | 0.735 |
| CGA | P01215 | Neurology | 0.93(0.78,1.10) | 4.05E-01 | 0.735 |
| TMSB10 | P63313 | Neurology | 0.94(0.80,1.09) | 4.08E-01 | 0.739 |
| AKT1S1 | Q96B36 | Neurology | 0.93(0.78,1.11) | 4.10E-01 | 0.740 |
| PSME2 | Q9UL46 | Neurology | 1.14(0.84,1.55) | 4.11E-01 | 0.740 |
| PPT1 | P50897 | Neurology II | 0.92(0.76,1.12) | 4.12E-01 | 0.740 |
| BRME1 | Q0VDD7 | Neurology II | 1.12(0.85,1.49) | 4.13E-01 | 0.740 |
| PDCD5 | O14737 | Neurology | 0.92(0.76,1.12) | 4.18E-01 | 0.747 |
| ULBP2 | Q9BZM5 | Neurology | 1.13(0.84,1.52) | 4.19E-01 | 0.747 |
| SETMAR | Q53H47 | Neurology | 0.85(0.57,1.26) | 4.22E-01 | 0.751 |
| CETN2 | P41208 | Neurology | 0.93(0.78,1.11) | 4.24E-01 | 0.752 |
| TNR | Q92752 | Neurology | 0.90(0.69,1.17) | 4.26E-01 | 0.752 |
| FBN2 | P35556 | Neurology II | 0.91(0.73,1.15) | 4.26E-01 | 0.752 |
| CPA2 | P48052 | Neurology | 1.07(0.91,1.26) | 4.27E-01 | 0.752 |
| CA6 | P23280 | Neurology | 1.08(0.90,1.29) | 4.29E-01 | 0.754 |
| FH | P07954 | Neurology II | 1.06(0.91,1.23) | 4.31E-01 | 0.754 |
| LRRC38 | Q5VT99 | Neurology II | 0.89(0.67,1.19) | 4.31E-01 | 0.754 |
| GKN1 | Q9NS71 | Neurology | 1.18(0.78,1.79) | 4.32E-01 | 0.754 |
| ZHX2 | Q9Y6X8 | Neurology II | 1.16(0.80,1.67) | 4.34E-01 | 0.754 |
| RGMB | Q6NW40 | Neurology | 0.85(0.56,1.28) | 4.35E-01 | 0.754 |
| CD99 | P14209 | Neurology | 1.25(0.71,2.21) | 4.36E-01 | 0.754 |
| CLNS1A | P54105 | Neurology II | 0.85(0.56,1.28) | 4.36E-01 | 0.754 |
| CACNB1 | Q02641 | Neurology II | 1.09(0.88,1.35) | 4.37E-01 | 0.754 |
| VWC2L | B2RUY7 | Neurology II | 1.11(0.85,1.44) | 4.38E-01 | 0.754 |
| OSTN | P61366 | Neurology II | 0.87(0.62,1.23) | 4.43E-01 | 0.760 |
| S100A16 | Q96FQ6 | Neurology | 0.93(0.76,1.13) | 4.45E-01 | 0.760 |
| PMVK | Q15126 | Neurology | 0.96(0.87,1.06) | 4.46E-01 | 0.760 |
| INPP5J | Q15735 | Neurology II | 0.88(0.64,1.22) | 4.46E-01 | 0.760 |
| GGA1 | Q9UJY5 | Neurology | 1.07(0.90,1.26) | 4.48E-01 | 0.760 |
| CNTNAP4 | Q9C0A0 | Neurology II | 0.95(0.82,1.09) | 4.48E-01 | 0.760 |
| SNRPB2 | P08579 | Neurology II | 1.11(0.84,1.47) | 4.50E-01 | 0.760 |
| CA2 | P00918 | Neurology | 1.06(0.91,1.24) | 4.51E-01 | 0.760 |
| FGR | P09769 | Neurology | 1.07(0.90,1.28) | 4.52E-01 | 0.760 |
| LAIR2 | Q6ISS4 | Neurology | 0.96(0.86,1.07) | 4.52E-01 | 0.760 |
| PDRG1 | Q9NUG6 | Neurology II | 0.90(0.68,1.19) | 4.54E-01 | 0.761 |
| TP73 | O15350 | Neurology II | 1.16(0.79,1.71) | 4.56E-01 | 0.763 |
| EIF1AX | P47813 | Neurology II | 0.91(0.71,1.17) | 4.57E-01 | 0.763 |
| CD300LG | Q6UXG3 | Neurology | 1.12(0.83,1.50) | 4.60E-01 | 0.764 |
| ARID3A | Q99856 | Neurology II | 0.88(0.62,1.24) | 4.60E-01 | 0.764 |
| CHP1 | Q99653 | Neurology II | 0.93(0.77,1.13) | 4.63E-01 | 0.764 |
| GID8 | Q9NWU2 | Neurology II | 0.85(0.54,1.32) | 4.63E-01 | 0.764 |
| TARM1 | B6A8C7 | Neurology II | 0.93(0.78,1.12) | 4.63E-01 | 0.764 |
| CD164 | Q04900 | Neurology | 0.84(0.53,1.34) | 4.69E-01 | 0.770 |
| IL34 | Q6ZMJ4 | Neurology | 1.13(0.82,1.56) | 4.69E-01 | 0.770 |
| ECSCR | Q19T08 | Neurology II | 0.90(0.68,1.19) | 4.71E-01 | 0.772 |
| GIGYF2 | Q6Y7W6 | Neurology II | 0.94(0.79,1.12) | 4.72E-01 | 0.772 |
| C7orf50 | Q9BRJ6 | Neurology II | 0.78(0.40,1.53) | 4.76E-01 | 0.776 |
| PGM2 | Q96G03 | Neurology II | 0.91(0.71,1.18) | 4.77E-01 | 0.776 |
| ATXN3 | P54252 | Neurology II | 0.93(0.75,1.14) | 4.79E-01 | 0.778 |
| ALDH1A1 | P00352 | Neurology | 0.93(0.75,1.14) | 4.81E-01 | 0.778 |
| MMP13 | P45452 | Neurology | 1.14(0.80,1.63) | 4.81E-01 | 0.778 |
| PTPRR | Q15256 | Neurology II | 1.13(0.80,1.60) | 4.82E-01 | 0.778 |
| DGCR6 | Q14129 | Neurology II | 1.11(0.83,1.48) | 4.87E-01 | 0.782 |
| LGALS8 | O00214 | Neurology | 0.94(0.77,1.13) | 4.88E-01 | 0.782 |
| TCL1A | P56279 | Neurology | 0.96(0.86,1.08) | 4.89E-01 | 0.782 |
| GRIK2 | Q13002 | Neurology II | 1.11(0.83,1.47) | 4.89E-01 | 0.782 |
| NAAA | Q02083 | Neurology | 0.93(0.77,1.14) | 4.99E-01 | 0.796 |
| LMOD2 | Q6P5Q4 | Neurology II | 0.92(0.74,1.16) | 5.03E-01 | 0.799 |
| MN1 | Q10571 | Neurology II | 0.92(0.72,1.18) | 5.03E-01 | 0.799 |
| MIF | P14174 | Neurology | 0.95(0.80,1.12) | 5.08E-01 | 0.803 |
| CUZD1 | Q86UP6 | Neurology II | 1.11(0.81,1.52) | 5.08E-01 | 0.803 |
| C1QBP | Q07021 | Neurology II | 1.06(0.89,1.26) | 5.09E-01 | 0.803 |
| CNTF | P26441 | Neurology II | 0.88(0.59,1.30) | 5.10E-01 | 0.803 |
| EPHA10 | Q5JZY3 | Neurology | 1.06(0.89,1.25) | 5.11E-01 | 0.803 |
| PPP3R1 | P63098 | Neurology | 0.91(0.69,1.21) | 5.12E-01 | 0.803 |
| RRP15 | Q9Y3B9 | Neurology II | 0.95(0.82,1.11) | 5.13E-01 | 0.803 |
| CDAN1 | Q8IWY9 | Neurology II | 1.20(0.70,2.06) | 5.14E-01 | 0.803 |
| HIP1R | O75146 | Neurology II | 1.10(0.82,1.49) | 5.19E-01 | 0.809 |
| LRTM1 | Q9HBL6 | Neurology II | 1.11(0.81,1.52) | 5.21E-01 | 0.809 |
| CDCP1 | Q9H5V8 | Neurology | 1.07(0.87,1.32) | 5.23E-01 | 0.809 |
| ITGAM | P11215 | Neurology | 0.89(0.62,1.28) | 5.23E-01 | 0.809 |
| PCARE | A6NGG8 | Neurology II | 1.05(0.91,1.21) | 5.24E-01 | 0.809 |
| MAP1LC3A | Q9H492 | Neurology II | 1.15(0.75,1.75) | 5.26E-01 | 0.811 |
| NSFL1C | Q9UNZ2 | Neurology | 0.95(0.80,1.12) | 5.30E-01 | 0.813 |
| HNF1A | P20823 | Neurology II | 1.06(0.88,1.27) | 5.30E-01 | 0.813 |
| SLC1A4 | P43007 | Neurology II | 1.08(0.84,1.40) | 5.32E-01 | 0.814 |
| IL17RA | Q96F46 | Neurology | 0.92(0.72,1.19) | 5.33E-01 | 0.814 |
| PPP1R14D | Q9NXH3 | Neurology II | 1.11(0.81,1.51) | 5.34E-01 | 0.814 |
| CDH23 | Q9H251 | Neurology II | 0.85(0.51,1.42) | 5.37E-01 | 0.816 |
| CALCA | P01258 | Neurology | 0.94(0.79,1.13) | 5.38E-01 | 0.816 |
| NUDT5 | Q9UKK9 | Neurology | 0.93(0.74,1.17) | 5.39E-01 | 0.816 |
| IL1RAP | Q9NPH3 | Neurology | 1.08(0.84,1.38) | 5.45E-01 | 0.816 |
| PTPN1 | P18031 | Neurology | 0.96(0.83,1.11) | 5.45E-01 | 0.816 |
| TFF1 | P04155 | Neurology | 1.04(0.91,1.19) | 5.45E-01 | 0.816 |
| RELT | Q969Z4 | Neurology | 1.12(0.77,1.64) | 5.46E-01 | 0.816 |
| ANXA3 | P12429 | Neurology | 0.95(0.81,1.12) | 5.47E-01 | 0.816 |
| FOLR2 | P14207 | Neurology | 1.12(0.77,1.63) | 5.48E-01 | 0.816 |
| GABRA4 | P48169 | Neurology II | 1.06(0.87,1.30) | 5.48E-01 | 0.816 |
| GPR158 | Q5T848 | Neurology II | 0.89(0.60,1.31) | 5.51E-01 | 0.816 |
| CIT | O14578 | Neurology II | 0.91(0.68,1.23) | 5.53E-01 | 0.816 |
| LETM1 | O95202 | Neurology II | 0.92(0.71,1.20) | 5.53E-01 | 0.816 |
| RAB6B | Q9NRW1 | Neurology | 1.07(0.85,1.35) | 5.55E-01 | 0.816 |
| RSPO1 | Q2MKA7 | Neurology | 1.11(0.79,1.56) | 5.56E-01 | 0.816 |
| CCAR2 | Q8N163 | Neurology II | 1.06(0.87,1.30) | 5.56E-01 | 0.816 |
| DPEP1 | P16444 | Neurology | 1.07(0.85,1.36) | 5.57E-01 | 0.816 |
| ISLR2 | Q6UXK2 | Neurology | 0.91(0.66,1.25) | 5.59E-01 | 0.816 |
| DEFB118 | Q96PH6 | Neurology II | 0.92(0.71,1.20) | 5.60E-01 | 0.816 |
| USP28 | Q96RU2 | Neurology II | 1.10(0.79,1.54) | 5.61E-01 | 0.816 |
| WASF3 | Q9UPY6 | Neurology | 0.96(0.84,1.10) | 5.63E-01 | 0.816 |
| DNPEP | Q9ULA0 | Neurology II | 1.07(0.85,1.36) | 5.63E-01 | 0.816 |
| LY96 | Q9Y6Y9 | Neurology | 0.91(0.66,1.25) | 5.65E-01 | 0.816 |
| SCIN | Q9Y6U3 | Neurology II | 0.95(0.78,1.14) | 5.65E-01 | 0.816 |
| NOS3 | P29474 | Neurology | 0.94(0.75,1.17) | 5.66E-01 | 0.816 |
| SCN3A | Q9NY46 | Neurology II | 1.11(0.78,1.57) | 5.66E-01 | 0.816 |
| HSP90B1 | P14625 | Neurology | 1.05(0.89,1.25) | 5.67E-01 | 0.816 |
| IL18RAP | O95256 | Neurology | 0.98(0.92,1.05) | 5.68E-01 | 0.816 |
| PTS | Q03393 | Neurology | 0.94(0.76,1.16) | 5.69E-01 | 0.816 |
| MAP2 | P11137 | Neurology II | 0.92(0.68,1.24) | 5.69E-01 | 0.816 |
| KRT8 | P05787 | Neurology II | 0.94(0.78,1.15) | 5.71E-01 | 0.816 |
| SCN2A | Q99250 | Neurology II | 1.08(0.83,1.40) | 5.71E-01 | 0.816 |
| CD3D | P04234 | Neurology II | 0.89(0.59,1.34) | 5.74E-01 | 0.818 |
| CRIP2 | P52943 | Neurology | 1.09(0.81,1.45) | 5.75E-01 | 0.818 |
| WWP2 | O00308 | Neurology | 0.94(0.76,1.16) | 5.76E-01 | 0.818 |
| DARS1 | P14868 | Neurology | 0.96(0.82,1.12) | 5.78E-01 | 0.820 |
| NMNAT1 | Q9HAN9 | Neurology | 1.04(0.90,1.21) | 5.81E-01 | 0.822 |
| PTK7 | Q13308 | Neurology | 1.09(0.80,1.47) | 5.85E-01 | 0.826 |
| SH3GLB2 | Q9NR46 | Neurology II | 0.95(0.78,1.15) | 5.88E-01 | 0.828 |
| SERPINB9 | P50453 | Neurology | 0.91(0.65,1.28) | 5.90E-01 | 0.828 |
| IPCEF1 | Q8WWN9 | Neurology | 0.97(0.85,1.10) | 5.91E-01 | 0.828 |
| MRPL46 | Q9H2W6 | Neurology | 0.89(0.58,1.36) | 5.92E-01 | 0.828 |
| DDX25 | Q9UHL0 | Neurology II | 0.90(0.62,1.31) | 5.92E-01 | 0.828 |
| BIN2 | Q9UBW5 | Neurology | 0.97(0.85,1.09) | 5.94E-01 | 0.829 |
| ARMCX2 | Q7L311 | Neurology II | 0.94(0.75,1.18) | 5.96E-01 | 0.831 |
| STC2 | O76061 | Neurology | 1.13(0.71,1.80) | 6.00E-01 | 0.834 |
| WASHC3 | Q9Y3C0 | Neurology II | 0.95(0.80,1.14) | 6.03E-01 | 0.835 |
| METAP1 | P53582 | Neurology | 1.11(0.75,1.64) | 6.05E-01 | 0.835 |
| CPPED1 | Q9BRF8 | Neurology | 0.96(0.81,1.13) | 6.08E-01 | 0.835 |
| GLT8D2 | Q9H1C3 | Neurology | 1.07(0.83,1.37) | 6.08E-01 | 0.835 |
| PADI4 | Q9UM07 | Neurology | 0.96(0.81,1.13) | 6.08E-01 | 0.835 |
| CLSTN1 | O94985 | Neurology | 1.06(0.86,1.31) | 6.11E-01 | 0.835 |
| FKBP5 | Q13451 | Neurology | 0.97(0.86,1.09) | 6.11E-01 | 0.835 |
| IDI2 | Q9BXS1 | Neurology | 1.06(0.84,1.35) | 6.11E-01 | 0.835 |
| BHLHE40 | O14503 | Neurology II | 0.95(0.78,1.16) | 6.12E-01 | 0.835 |
| PLA2G10 | O15496 | Neurology | 1.05(0.87,1.27) | 6.13E-01 | 0.835 |
| RPGR | Q92834 | Neurology II | 1.08(0.79,1.48) | 6.13E-01 | 0.835 |
| GSTT2B | P0CG30 | Neurology II | 1.01(0.96,1.07) | 6.18E-01 | 0.838 |
| CCDC50 | Q8IVM0 | Neurology II | 0.96(0.80,1.14) | 6.20E-01 | 0.838 |
| AHSP | Q9NZD4 | Neurology | 0.97(0.85,1.10) | 6.21E-01 | 0.838 |
| MMP8 | P22894 | Neurology | 1.04(0.89,1.22) | 6.21E-01 | 0.838 |
| PALM2 | Q8IXS6 | Neurology II | 1.09(0.78,1.52) | 6.21E-01 | 0.838 |
| TDGF1 | P13385 | Neurology | 0.98(0.91,1.06) | 6.22E-01 | 0.838 |
| NRN1 | Q9NPD7 | Neurology II | 1.09(0.78,1.53) | 6.25E-01 | 0.840 |
| ABHD14B | Q96IU4 | Neurology | 0.95(0.77,1.17) | 6.27E-01 | 0.840 |
| ASGR1 | P07306 | Neurology | 1.09(0.76,1.56) | 6.27E-01 | 0.840 |
| ELAVL4 | P26378 | Neurology II | 1.04(0.89,1.22) | 6.29E-01 | 0.841 |
| SIRT5 | Q9NXA8 | Neurology | 1.05(0.86,1.29) | 6.31E-01 | 0.842 |
| RHOC | P08134 | Neurology | 0.98(0.89,1.08) | 6.32E-01 | 0.842 |
| CSPG5 | O95196 | Neurology II | 0.93(0.71,1.23) | 6.34E-01 | 0.843 |
| VSTM1 | Q6UX27 | Neurology | 1.06(0.84,1.32) | 6.38E-01 | 0.845 |
| CSF2RA | P15509 | Neurology | 1.04(0.87,1.25) | 6.39E-01 | 0.845 |
| AMPD3 | Q01432 | Neurology II | 1.04(0.87,1.25) | 6.40E-01 | 0.845 |
| KCNC4 | Q03721 | Neurology II | 1.06(0.84,1.32) | 6.40E-01 | 0.845 |
| CD8A | P01732 | Neurology | 0.95(0.77,1.18) | 6.43E-01 | 0.847 |
| IL7R | P16871 | Neurology | 0.95(0.77,1.18) | 6.44E-01 | 0.847 |
| SMTN | P53814 | Neurology II | 0.97(0.84,1.11) | 6.49E-01 | 0.850 |
| IFNGR2 | P38484 | Neurology | 0.95(0.76,1.19) | 6.50E-01 | 0.850 |
| CNTN4 | Q8IWV2 | Neurology | 1.11(0.70,1.78) | 6.51E-01 | 0.850 |
| SEMA4D | Q92854 | Neurology | 0.92(0.63,1.33) | 6.51E-01 | 0.850 |
| GRIN2B | Q13224 | Neurology II | 0.94(0.73,1.22) | 6.52E-01 | 0.850 |
| BAG3 | O95817 | Neurology | 0.94(0.71,1.24) | 6.56E-01 | 0.850 |
| NID2 | Q14112 | Neurology | 0.97(0.83,1.13) | 6.56E-01 | 0.850 |
| PCDH12 | Q9NPG4 | Neurology II | 1.13(0.67,1.90) | 6.56E-01 | 0.850 |
| SERPINB1 | P30740 | Neurology | 0.97(0.85,1.10) | 6.61E-01 | 0.855 |
| S100G | P29377 | Neurology II | 0.94(0.71,1.24) | 6.62E-01 | 0.855 |
| ARSA | P15289 | Neurology | 0.95(0.74,1.22) | 6.73E-01 | 0.868 |
| OSBPL2 | Q9H1P3 | Neurology II | 0.93(0.65,1.33) | 6.75E-01 | 0.869 |
| TMPRSS5 | Q9H3S3 | Neurology | 1.07(0.79,1.43) | 6.77E-01 | 0.870 |
| SPACA5_SPACA5B | Q96QH8 | Neurology II | 1.09(0.71,1.68) | 6.80E-01 | 0.870 |
| DNMBP | Q6XZF7 | Neurology | 1.04(0.86,1.25) | 6.81E-01 | 0.870 |
| ING1 | Q9UK53 | Neurology | 0.95(0.74,1.22) | 6.82E-01 | 0.870 |
| MPO | P05164 | Neurology | 1.05(0.83,1.34) | 6.82E-01 | 0.870 |
| CXCL11 | O14625 | Neurology | 1.03(0.89,1.19) | 6.84E-01 | 0.871 |
| TPSD1 | Q9BZJ3 | Neurology II | 0.93(0.65,1.33) | 6.85E-01 | 0.871 |
| STEAP4 | Q687X5 | Neurology II | 1.04(0.86,1.25) | 6.90E-01 | 0.876 |
| SCARB1 | Q8WTV0 | Neurology | 0.96(0.77,1.19) | 6.92E-01 | 0.877 |
| ADAM22 | Q9P0K1 | Neurology | 0.94(0.68,1.29) | 6.95E-01 | 0.878 |
| UROS | P10746 | Neurology II | 0.96(0.78,1.18) | 6.95E-01 | 0.878 |
| FKBP7 | Q9Y680 | Neurology | 1.05(0.83,1.32) | 7.00E-01 | 0.882 |
| KIRREL2 | Q6UWL6 | Neurology | 1.06(0.79,1.41) | 7.02E-01 | 0.883 |
| DBI | P07108 | Neurology | 0.97(0.84,1.13) | 7.03E-01 | 0.883 |
| CD109 | Q6YHK3 | Neurology | 1.07(0.76,1.50) | 7.05E-01 | 0.884 |
| BLVRB | P30043 | Neurology | 1.03(0.88,1.22) | 7.07E-01 | 0.885 |
| TREML2 | Q5T2D2 | Neurology | 1.06(0.77,1.46) | 7.09E-01 | 0.886 |
| MAG | P20916 | Neurology II | 0.95(0.72,1.25) | 7.11E-01 | 0.887 |
| OTOA | Q7RTW8 | Neurology II | 0.95(0.73,1.24) | 7.12E-01 | 0.887 |
| IL13RA2 | Q14627 | Neurology II | 0.92(0.57,1.46) | 7.15E-01 | 0.889 |
| CLEC10A | Q8IUN9 | Neurology | 0.94(0.69,1.30) | 7.17E-01 | 0.890 |
| COL28A1 | Q2UY09 | Neurology II | 0.97(0.81,1.16) | 7.20E-01 | 0.892 |
| CD164L2 | Q6UWJ8 | Neurology II | 0.93(0.64,1.36) | 7.25E-01 | 0.896 |
| GGT1 | P19440 | Neurology | 0.96(0.76,1.21) | 7.28E-01 | 0.896 |
| TUBB3 | Q13509 | Neurology II | 1.09(0.67,1.77) | 7.30E-01 | 0.896 |
| NEO1 | Q92859 | Neurology II | 0.90(0.50,1.63) | 7.31E-01 | 0.896 |
| CARHSP1 | Q9Y2V2 | Neurology | 0.96(0.78,1.20) | 7.32E-01 | 0.896 |
| FZD10 | Q9ULW2 | Neurology II | 1.04(0.83,1.30) | 7.32E-01 | 0.896 |
| SLC39A14 | Q15043 | Neurology | 0.94(0.66,1.33) | 7.33E-01 | 0.896 |
| CLEC11A | Q9Y240 | Neurology | 1.04(0.82,1.32) | 7.34E-01 | 0.896 |
| PXN | P49023 | Neurology | 0.96(0.77,1.21) | 7.35E-01 | 0.896 |
| MYCBP2 | O75592 | Neurology II | 0.97(0.79,1.18) | 7.36E-01 | 0.896 |
| C2CD2L | O14523 | Neurology | 0.95(0.72,1.26) | 7.39E-01 | 0.896 |
| KRT5 | P13647 | Neurology | 0.96(0.76,1.21) | 7.41E-01 | 0.896 |
| BAP18 | Q8IXM2 | Neurology II | 0.97(0.79,1.18) | 7.42E-01 | 0.896 |
| GBA | P04062 | Neurology II | 0.96(0.74,1.24) | 7.43E-01 | 0.896 |
| KCNH2 | Q12809 | Neurology II | 1.03(0.87,1.22) | 7.45E-01 | 0.896 |
| BCAM | P50895 | Neurology | 1.08(0.67,1.74) | 7.47E-01 | 0.896 |
| TNXB | P22105 | Neurology | 0.93(0.59,1.45) | 7.47E-01 | 0.896 |
| LRP2BP | Q9P2M1 | Neurology II | 0.97(0.83,1.15) | 7.47E-01 | 0.896 |
| MYO6 | Q9UM54 | Neurology II | 0.94(0.65,1.37) | 7.47E-01 | 0.896 |
| EFNB2 | P52799 | Neurology II | 0.96(0.75,1.23) | 7.48E-01 | 0.896 |
| SH3BGRL2 | Q9UJC5 | Neurology II | 0.96(0.76,1.22) | 7.52E-01 | 0.899 |
| SMARCA2 | P51531 | Neurology | 1.03(0.87,1.20) | 7.55E-01 | 0.902 |
| TMEM25 | Q86YD3 | Neurology II | 0.96(0.71,1.28) | 7.60E-01 | 0.902 |
| MDGA1 | Q8NFP4 | Neurology | 0.97(0.83,1.15) | 7.61E-01 | 0.902 |
| BRD2 | P25440 | Neurology II | 0.94(0.63,1.40) | 7.61E-01 | 0.902 |
| C1QTNF6 | Q9BXI9 | Neurology II | 0.94(0.61,1.43) | 7.65E-01 | 0.902 |
| LRPAP1 | P30533 | Neurology | 0.97(0.79,1.19) | 7.66E-01 | 0.902 |
| PTEN | P60484 | Neurology | 1.06(0.72,1.55) | 7.66E-01 | 0.902 |
| SAG | P10523 | Neurology II | 0.96(0.73,1.26) | 7.66E-01 | 0.902 |
| HNMT | P50135 | Neurology | 1.04(0.82,1.31) | 7.67E-01 | 0.902 |
| AZI2 | Q9H6S1 | Neurology II | 0.96(0.76,1.22) | 7.67E-01 | 0.902 |
| NOMO1 | Q15155 | Neurology | 0.93(0.58,1.50) | 7.70E-01 | 0.905 |
| SCN2B | O60939 | Neurology II | 0.93(0.58,1.50) | 7.73E-01 | 0.907 |
| LIF | P15018 | Neurology | 0.97(0.77,1.22) | 7.75E-01 | 0.908 |
| PRL | P01236 | Neurology | 1.02(0.87,1.20) | 7.78E-01 | 0.909 |
| TIGAR | Q9NQ88 | Neurology | 0.96(0.75,1.24) | 7.79E-01 | 0.909 |
| SCARA5 | Q6ZMJ2 | Neurology | 1.05(0.73,1.52) | 7.83E-01 | 0.912 |
| TEX101 | Q9BY14 | Neurology II | 0.97(0.77,1.22) | 7.94E-01 | 0.924 |
| DCTN6 | O00399 | Neurology | 0.98(0.83,1.15) | 7.97E-01 | 0.926 |
| PMS1 | P54277 | Neurology II | 1.03(0.80,1.34) | 8.02E-01 | 0.930 |
| FOSB | P53539 | Neurology | 1.04(0.77,1.40) | 8.08E-01 | 0.932 |
| SFRP1 | Q8N474 | Neurology | 0.97(0.74,1.26) | 8.08E-01 | 0.932 |
| TPPP3 | Q9BW30 | Neurology | 1.04(0.77,1.39) | 8.12E-01 | 0.932 |
| KCTD5 | Q9NXV2 | Neurology II | 0.96(0.71,1.31) | 8.12E-01 | 0.932 |
| FUT3_FUT5 | P21217_Q11128 | Neurology | 0.97(0.76,1.24) | 8.15E-01 | 0.932 |
| SOD2 | P04179 | Neurology | 0.96(0.71,1.31) | 8.19E-01 | 0.932 |
| CD34 | P28906 | Neurology | 0.95(0.59,1.51) | 8.20E-01 | 0.932 |
| AMIGO1 | Q86WK6 | Neurology II | 1.03(0.79,1.34) | 8.22E-01 | 0.932 |
| FNDC1 | Q4ZHG4 | Neurology II | 0.97(0.73,1.28) | 8.23E-01 | 0.932 |
| PEBP1 | P30086 | Neurology | 0.98(0.80,1.19) | 8.24E-01 | 0.932 |
| SLIT2 | O94813 | Neurology | 0.98(0.78,1.21) | 8.24E-01 | 0.932 |
| CERT | Q9Y5P4 | Neurology | 0.98(0.81,1.18) | 8.28E-01 | 0.932 |
| ANXA10 | Q9UJ72 | Neurology | 0.98(0.86,1.13) | 8.29E-01 | 0.932 |
| IFNL2 | Q8IZJ0 | Neurology II | 0.98(0.78,1.22) | 8.29E-01 | 0.932 |
| SPTLC1 | O15269 | Neurology II | 0.96(0.66,1.39) | 8.29E-01 | 0.932 |
| RBKS | Q9H477 | Neurology | 0.98(0.78,1.23) | 8.30E-01 | 0.932 |
| KLRC1 | P26715 | Neurology II | 0.97(0.77,1.23) | 8.30E-01 | 0.932 |
| PSME1 | Q06323 | Neurology | 0.97(0.71,1.31) | 8.32E-01 | 0.932 |
| VTA1 | Q9NP79 | Neurology | 0.98(0.84,1.15) | 8.34E-01 | 0.932 |
| SEPTIN3 | Q9UH03 | Neurology II | 1.02(0.86,1.21) | 8.36E-01 | 0.932 |
| PLB1 | Q6P1J6 | Neurology II | 1.02(0.82,1.27) | 8.37E-01 | 0.932 |
| SOX9 | P48436 | Neurology II | 0.95(0.57,1.57) | 8.37E-01 | 0.932 |
| THY1 | P04216 | Neurology | 1.04(0.72,1.51) | 8.38E-01 | 0.932 |
| ARID4B | Q4LE39 | Neurology | 0.97(0.73,1.29) | 8.39E-01 | 0.932 |
| CBLN1 | P23435 | Neurology II | 0.98(0.85,1.14) | 8.39E-01 | 0.932 |
| MTHFD2 | P13995 | Neurology II | 0.95(0.56,1.59) | 8.39E-01 | 0.932 |
| FRZB | Q92765 | Neurology | 0.95(0.61,1.50) | 8.41E-01 | 0.932 |
| C19orf12 | Q9NSK7 | Neurology | 1.02(0.85,1.21) | 8.43E-01 | 0.932 |
| PARK7 | Q99497 | Neurology | 0.98(0.79,1.21) | 8.44E-01 | 0.932 |
| KIF20B | Q96Q89 | Neurology II | 1.02(0.85,1.21) | 8.44E-01 | 0.932 |
| GBP1 | P32455 | Neurology II | 1.02(0.83,1.26) | 8.45E-01 | 0.932 |
| OMP | P47874 | Neurology II | 1.03(0.78,1.35) | 8.45E-01 | 0.932 |
| CXCL13 | O43927 | Neurology | 0.98(0.82,1.18) | 8.47E-01 | 0.933 |
| CASP10 | Q92851 | Neurology | 0.98(0.84,1.16) | 8.49E-01 | 0.934 |
| PLIN1 | O60240 | Neurology | 1.03(0.75,1.42) | 8.51E-01 | 0.935 |
| GPKOW | Q92917 | Neurology | 1.04(0.70,1.53) | 8.57E-01 | 0.940 |
| XRCC4 | Q13426 | Neurology | 1.03(0.76,1.39) | 8.65E-01 | 0.944 |
| RNASEH2A | O75792 | Neurology II | 0.98(0.74,1.29) | 8.65E-01 | 0.944 |
| TPBGL | P0DKB5 | Neurology II | 0.97(0.71,1.34) | 8.65E-01 | 0.944 |
| ATF2 | P15336 | Neurology | 1.01(0.85,1.21) | 8.68E-01 | 0.946 |
| CAPS | Q13938 | Neurology II | 0.99(0.87,1.13) | 8.71E-01 | 0.946 |
| SATB1 | Q01826 | Neurology II | 0.96(0.62,1.51) | 8.71E-01 | 0.946 |
| CHGB | P05060 | Neurology | 1.02(0.78,1.34) | 8.73E-01 | 0.946 |
| EZR | P15311 | Neurology | 0.96(0.60,1.54) | 8.73E-01 | 0.946 |
| SEPTIN8 | Q92599 | Neurology II | 1.03(0.68,1.57) | 8.82E-01 | 0.952 |
| GART | P22102 | Neurology II | 0.96(0.59,1.58) | 8.83E-01 | 0.952 |
| SLC27A4 | Q6P1M0 | Neurology | 0.99(0.83,1.18) | 8.84E-01 | 0.952 |
| KIRREL1 | Q96J84 | Neurology II | 1.02(0.81,1.27) | 8.84E-01 | 0.952 |
| LYPLA2 | O95372 | Neurology II | 0.99(0.87,1.12) | 8.86E-01 | 0.953 |
| FAM171A2 | A8MVW0 | Neurology II | 0.99(0.91,1.08) | 8.87E-01 | 0.953 |
| MORC3 | Q14149 | Neurology II | 1.03(0.66,1.61) | 8.89E-01 | 0.953 |
| SOX2 | P48431 | Neurology II | 0.96(0.55,1.67) | 8.91E-01 | 0.954 |
| ENO1 | P06733 | Neurology | 0.99(0.86,1.14) | 8.99E-01 | 0.961 |
| GRN | P28799 | Neurology | 0.97(0.61,1.56) | 9.07E-01 | 0.968 |
| PLA2G7 | Q13093 | Neurology | 0.98(0.70,1.38) | 9.09E-01 | 0.968 |
| GOLM2 | Q6P4E1 | Neurology | 1.03(0.63,1.67) | 9.11E-01 | 0.968 |
| MUC13 | Q9H3R2 | Neurology | 0.99(0.79,1.24) | 9.12E-01 | 0.968 |
| FGFBP3 | Q8TAT2 | Neurology II | 0.98(0.68,1.40) | 9.12E-01 | 0.968 |
| SAFB2 | Q14151 | Neurology II | 1.03(0.65,1.62) | 9.13E-01 | 0.968 |
| LILRA2 | Q8N149 | Neurology | 1.02(0.75,1.39) | 9.15E-01 | 0.968 |
| ARHGEF5 | Q12774 | Neurology II | 1.01(0.84,1.21) | 9.23E-01 | 0.975 |
| LRFN2 | Q9ULH4 | Neurology II | 1.01(0.76,1.35) | 9.25E-01 | 0.976 |
| PSMD1 | Q99460 | Neurology II | 0.99(0.75,1.30) | 9.31E-01 | 0.980 |
| PLAU | P00749 | Neurology | 0.98(0.68,1.42) | 9.34E-01 | 0.980 |
| ITPRIP | Q8IWB1 | Neurology II | 1.01(0.75,1.37) | 9.34E-01 | 0.980 |
| FHIT | P49789 | Neurology | 0.99(0.85,1.16) | 9.35E-01 | 0.980 |
| LRP2 | P98164 | Neurology II | 1.01(0.71,1.46) | 9.37E-01 | 0.980 |
| OBP2B | Q9NPH6 | Neurology | 1.01(0.84,1.21) | 9.38E-01 | 0.980 |
| ADGRV1 | Q8WXG9 | Neurology II | 1.01(0.83,1.22) | 9.38E-01 | 0.980 |
| INSL3 | P51460 | Neurology II | 0.99(0.86,1.15) | 9.42E-01 | 0.980 |
| EPB41L5 | Q9HCM4 | Neurology II | 1.01(0.81,1.25) | 9.43E-01 | 0.980 |
| LBR | Q14739 | Neurology | 1.01(0.85,1.19) | 9.44E-01 | 0.980 |
| SLC44A4 | Q53GD3 | Neurology II | 0.99(0.82,1.20) | 9.44E-01 | 0.980 |
| CRYGD | P07320 | Neurology II | 1.00(0.87,1.14) | 9.49E-01 | 0.984 |
| CNGB3 | Q9NQW8 | Neurology II | 0.99(0.77,1.28) | 9.51E-01 | 0.985 |
| COPE | O14579 | Neurology | 1.01(0.78,1.30) | 9.54E-01 | 0.985 |
| CCDC28A | Q8IWP9 | Neurology II | 0.99(0.63,1.55) | 9.54E-01 | 0.985 |
| APRT | P07741 | Neurology | 0.99(0.78,1.26) | 9.56E-01 | 0.986 |
| SLC16A1 | P53985 | Neurology | 0.99(0.81,1.22) | 9.59E-01 | 0.987 |
| OCLN | Q16625 | Neurology II | 0.99(0.73,1.35) | 9.60E-01 | 0.987 |
| BCAT2 | O15382 | Neurology II | 0.99(0.77,1.28) | 9.61E-01 | 0.987 |
| HMOX2 | P30519 | Neurology | 0.99(0.77,1.29) | 9.63E-01 | 0.987 |
| ATXN2 | Q99700 | Neurology II | 1.00(0.88,1.13) | 9.68E-01 | 0.991 |
| AKR1B10 | O60218 | Neurology II | 1.00(0.80,1.24) | 9.69E-01 | 0.991 |
| AGBL2 | Q5U5Z8 | Neurology II | 1.00(0.86,1.15) | 9.71E-01 | 0.991 |
| CLIC5 | Q9NZA1 | Neurology II | 1.01(0.65,1.55) | 9.72E-01 | 0.991 |
| TFAP2A | P05549 | Neurology II | 1.00(0.79,1.25) | 9.74E-01 | 0.991 |
| LXN | Q9BS40 | Neurology | 1.00(0.84,1.18) | 9.76E-01 | 0.991 |
| SULT1A1 | P50225 | Neurology | 1.00(0.92,1.09) | 9.76E-01 | 0.991 |
| SKAP1 | Q86WV1 | Neurology | 1.00(0.86,1.17) | 9.79E-01 | 0.991 |
| TRIM40 | Q6P9F5 | Neurology II | 1.00(0.83,1.20) | 9.79E-01 | 0.991 |
| SESTD1 | Q86VW0 | Neurology | 1.00(0.84,1.20) | 9.81E-01 | 0.992 |
| TBC1D17 | Q9HA65 | Neurology | 1.00(0.78,1.29) | 9.88E-01 | 0.996 |
| SCT | P09683 | Neurology II | 1.00(0.76,1.31) | 9.90E-01 | 0.996 |
| GFRA3 | O60609 | Neurology | 1.00(0.67,1.51) | 9.91E-01 | 0.996 |
| BST1 | Q10588 | Neurology | 1.00(0.79,1.27) | 9.92E-01 | 0.996 |
| PAEP | P09466 | Neurology | 1.00(0.89,1.13) | 9.93E-01 | 0.996 |
| FUT8 | Q9BYC5 | Neurology | 1.00(0.82,1.22) | 9.95E-01 | 0.996 |
| GSTP1 | P09211 | Neurology | 1.00(0.85,1.18) | 9.95E-01 | 0.996 |
| CALB2 | P22676 | Neurology | 1.00(0.69,1.45) | 9.96E-01 | 0.996 |

**^*^**Cox regression model adjusted for age and sex. *P* value was calculated under two-sided tests, and statistical significance was defined as a false discovery rate (FDR)-corrected *P* value <0.05, adjusted for the number of proteins tested (n=726).

**Table S9. Cox regression analysis for the associations between 730 proteins from the oncology panel and the risk of new-onset Alzheimer’s disease in the training set. ^*^**

| **Target Name** | **UniProt** | **Panel** | **HR (95%CI)** | ***P* value** | **FDR-corrected *P* value** |
| --- | --- | --- | --- | --- | --- |
| GFAP | P14136 | Oncology | 3.42(2.90,4.04) | 2.92E-47 | <0.001 |
| PDGFC | Q9NRA1 | Oncology | 0.45(0.30,0.68) | 1.30E-04 | 0.029 |
| CEACAM5 | P06731 | Oncology | 1.37(1.16,1.61) | 1.54E-04 | 0.029 |
| EDA2R | Q9HAV5 | Oncology | 1.69(1.28,2.22) | 1.96E-04 | 0.029 |
| OMG | P23515 | Oncology | 0.73(0.62,0.86) | 2.00E-04 | 0.029 |
| HPGDS | O60760 | Oncology | 0.58(0.42,0.79) | 5.83E-04 | 0.071 |
| ERP44 | Q9BS26 | Oncology | 0.46(0.29,0.72) | 7.71E-04 | 0.080 |
| CA12 | O43570 | Oncology | 1.70(1.22,2.37) | 1.72E-03 | 0.157 |
| PALM | O75781 | Oncology II | 1.81(1.23,2.66) | 2.68E-03 | 0.199 |
| FURIN | P09958 | Oncology | 0.61(0.45,0.85) | 3.10E-03 | 0.199 |
| KAZALD1 | Q96I82 | Oncology | 1.48(1.14,1.93) | 3.54E-03 | 0.199 |
| KLK4 | Q9Y5K2 | Oncology | 1.28(1.08,1.52) | 3.58E-03 | 0.199 |
| EBI3_IL27 | Q14213_Q8NEV9 | Oncology | 1.68(1.18,2.40) | 3.94E-03 | 0.199 |
| CD101 | Q93033 | Oncology II | 0.68(0.52,0.89) | 4.51E-03 | 0.199 |
| DSG4 | Q86SJ6 | Oncology | 0.72(0.58,0.91) | 4.55E-03 | 0.199 |
| ITGAV | P06756 | Oncology | 0.38(0.19,0.74) | 4.58E-03 | 0.199 |
| ADAM9 | Q13443 | Oncology II | 1.80(1.20,2.71) | 4.75E-03 | 0.199 |
| EPHA2 | P29317 | Oncology | 1.63(1.16,2.29) | 4.90E-03 | 0.199 |
| HSPB6 | O14558 | Oncology | 1.45(1.11,1.90) | 5.85E-03 | 0.225 |
| SCLY | Q96I15 | Oncology | 0.70(0.55,0.91) | 6.24E-03 | 0.228 |
| ERBB2 | P04626 | Oncology | 0.54(0.34,0.85) | 8.41E-03 | 0.292 |
| WFDC2 | Q14508 | Oncology | 1.41(1.09,1.83) | 9.57E-03 | 0.305 |
| ITGAX | P20702 | Oncology II | 0.67(0.50,0.91) | 9.60E-03 | 0.305 |
| PLXDC1 | Q8IUK5 | Oncology | 0.58(0.38,0.88) | 1.14E-02 | 0.322 |
| SDHB | P21912 | Oncology II | 0.74(0.59,0.94) | 1.15E-02 | 0.322 |
| ADAMTS8 | Q9UP79 | Oncology | 0.73(0.57,0.93) | 1.19E-02 | 0.322 |
| AREG | P15514 | Oncology | 1.36(1.07,1.74) | 1.34E-02 | 0.322 |
| IL13RA1 | P78552 | Oncology | 0.55(0.35,0.89) | 1.40E-02 | 0.322 |
| ARHGAP5 | Q13017 | Oncology II | 0.77(0.63,0.95) | 1.41E-02 | 0.322 |
| ALDH2 | P05091 | Oncology II | 0.64(0.45,0.92) | 1.44E-02 | 0.322 |
| GPA33 | Q99795 | Oncology | 0.90(0.83,0.98) | 1.48E-02 | 0.322 |
| VSIG2 | Q96IQ7 | Oncology II | 1.26(1.05,1.51) | 1.50E-02 | 0.322 |
| DLL1 | O00548 | Oncology | 1.56(1.09,2.23) | 1.56E-02 | 0.322 |
| CREG1 | O75629 | Oncology | 0.69(0.51,0.93) | 1.57E-02 | 0.322 |
| CENPJ | Q9HC77 | Oncology II | 0.77(0.63,0.95) | 1.57E-02 | 0.322 |
| BRAP | Q7Z569 | Oncology II | 0.87(0.77,0.97) | 1.62E-02 | 0.322 |
| GCG | P01275 | Oncology | 0.90(0.82,0.98) | 1.63E-02 | 0.322 |
| PDCD1LG2 | Q9BQ51 | Oncology | 1.52(1.08,2.15) | 1.76E-02 | 0.333 |
| GORASP2 | Q9H8Y8 | Oncology II | 0.70(0.52,0.94) | 1.78E-02 | 0.333 |
| NCR3LG1 | Q68D85 | Oncology II | 1.56(1.08,2.27) | 1.87E-02 | 0.337 |
| PDXDC1 | Q6P996 | Oncology II | 1.23(1.03,1.46) | 1.90E-02 | 0.337 |
| MAPKAPK2 | P49137 | Oncology II | 0.80(0.67,0.96) | 1.94E-02 | 0.337 |
| ACRBP | Q8NEB7 | Oncology II | 0.65(0.45,0.94) | 2.13E-02 | 0.362 |
| MMP12 | P39900 | Oncology | 1.26(1.03,1.53) | 2.19E-02 | 0.363 |
| GALNT2 | Q10471 | Oncology | 0.62(0.41,0.94) | 2.29E-02 | 0.371 |
| UFD1 | Q92890 | Oncology II | 0.90(0.82,0.99) | 2.44E-02 | 0.381 |
| RAD51 | Q06609 | Oncology II | 0.69(0.50,0.95) | 2.45E-02 | 0.381 |
| NPY | P01303 | Oncology | 0.83(0.70,0.98) | 2.66E-02 | 0.397 |
| P4HB | P07237 | Oncology | 0.71(0.53,0.96) | 2.70E-02 | 0.397 |
| ADGRG1 | Q9Y653 | Oncology | 1.14(1.02,1.29) | 2.72E-02 | 0.397 |
| AMOTL2 | Q9Y2J4 | Oncology II | 0.69(0.50,0.96) | 2.84E-02 | 0.404 |
| CES3 | Q6UWW8 | Oncology | 0.82(0.69,0.98) | 2.88E-02 | 0.404 |
| GPD1 | P21695 | Oncology II | 0.75(0.58,0.97) | 2.99E-02 | 0.412 |
| MAGEA3 | P43357 | Oncology II | 0.87(0.76,0.99) | 3.05E-02 | 0.412 |
| GFRA1 | P56159 | Oncology | 1.50(1.04,2.18) | 3.14E-02 | 0.417 |
| SMPDL3B | Q92485 | Oncology II | 0.78(0.63,0.98) | 3.20E-02 | 0.417 |
| LYPD8 | Q6UX82 | Oncology | 1.26(1.02,1.56) | 3.37E-02 | 0.419 |
| KLK14 | Q9P0G3 | Oncology | 1.29(1.02,1.64) | 3.49E-02 | 0.419 |
| HTRA2 | O43464 | Oncology | 0.82(0.67,0.99) | 3.53E-02 | 0.419 |
| KIAA1549 | Q9HCM3 | Oncology II | 0.82(0.68,0.99) | 3.54E-02 | 0.419 |
| CIRBP | Q14011 | Oncology II | 0.86(0.75,0.99) | 3.56E-02 | 0.419 |
| GAGE2A | Q6NT46 | Oncology II | 0.76(0.59,0.98) | 3.56E-02 | 0.419 |
| STOML2 | Q9UJZ1 | Oncology II | 0.84(0.72,0.99) | 3.74E-02 | 0.433 |
| MANSC1 | Q9H8J5 | Oncology | 1.52(1.02,2.25) | 3.84E-02 | 0.437 |
| EGFL7 | Q9UHF1 | Oncology | 0.73(0.54,0.98) | 3.91E-02 | 0.437 |
| RAPGEF2 | Q9Y4G8 | Oncology II | 0.75(0.57,0.99) | 3.95E-02 | 0.437 |
| TRIM58 | Q8NG06 | Oncology II | 0.86(0.74,0.99) | 4.08E-02 | 0.439 |
| MTSS2 | Q765P7 | Oncology II | 0.87(0.76,1.00) | 4.26E-02 | 0.439 |
| NBL1 | P41271 | Oncology | 1.52(1.01,2.29) | 4.27E-02 | 0.439 |
| CALB1 | P05937 | Oncology | 1.30(1.01,1.68) | 4.45E-02 | 0.439 |
| HAO1 | Q9UJM8 | Oncology | 0.91(0.83,1.00) | 4.45E-02 | 0.439 |
| SLC13A1 | Q9BZW2 | Oncology II | 1.19(1.00,1.41) | 4.50E-02 | 0.439 |
| FLT1 | P17948 | Oncology | 1.66(1.01,2.72) | 4.53E-02 | 0.439 |
| ITGB1BP1 | O14713 | Oncology | 1.33(1.01,1.76) | 4.53E-02 | 0.439 |
| SAP18 | O00422 | Oncology II | 0.82(0.68,1.00) | 4.57E-02 | 0.439 |
| CCDC134 | Q9H6E4 | Oncology II | 0.75(0.56,1.00) | 4.79E-02 | 0.439 |
| IL22 | Q9GZX6 | Oncology II | 1.19(1.00,1.42) | 4.82E-02 | 0.439 |
| IL6 | P05231 | Oncology | 1.14(1.00,1.29) | 4.87E-02 | 0.439 |
| LAT2 | Q9GZY6 | Oncology | 0.91(0.83,1.00) | 4.95E-02 | 0.439 |
| TXK | P42681 | Oncology II | 0.81(0.65,1.00) | 4.98E-02 | 0.439 |
| DYNLT3 | P51808 | Oncology II | 0.73(0.53,1.00) | 5.00E-02 | 0.439 |
| SMAD2 | Q15796 | Oncology II | 0.82(0.67,1.00) | 5.08E-02 | 0.439 |
| NELL1 | Q92832 | Oncology | 0.77(0.59,1.00) | 5.09E-02 | 0.439 |
| ARSB | P15848 | Oncology | 0.81(0.65,1.00) | 5.17E-02 | 0.439 |
| DTX3 | Q8N9I9 | Oncology | 1.54(1.00,2.38) | 5.26E-02 | 0.439 |
| LGALS7_LGALS7B | P47929 | Oncology | 1.28(1.00,1.64) | 5.36E-02 | 0.439 |
| MAMDC4 | Q6UXC1 | Oncology II | 0.78(0.61,1.01) | 5.48E-02 | 0.439 |
| CD82 | P27701 | Oncology II | 0.68(0.46,1.01) | 5.49E-02 | 0.439 |
| TGFBR2 | P37173 | Oncology | 1.35(0.99,1.83) | 5.51E-02 | 0.439 |
| ERI1 | Q8IV48 | Oncology II | 0.66(0.43,1.01) | 5.55E-02 | 0.439 |
| ARHGAP1 | Q07960 | Oncology | 0.78(0.60,1.01) | 5.57E-02 | 0.439 |
| MAPRE3 | Q9UPY8 | Oncology II | 0.90(0.80,1.00) | 5.58E-02 | 0.439 |
| THTPA | Q9BU02 | Oncology II | 0.86(0.74,1.00) | 5.60E-02 | 0.439 |
| DRG2 | P55039 | Oncology | 0.82(0.67,1.01) | 5.82E-02 | 0.439 |
| ARF6 | P62330 | Oncology II | 0.89(0.79,1.00) | 5.93E-02 | 0.439 |
| HDGF | P51858 | Oncology | 0.89(0.79,1.00) | 5.97E-02 | 0.439 |
| PPP1CC | P36873 | Oncology II | 0.86(0.73,1.01) | 6.11E-02 | 0.439 |
| SLAMF8 | Q9P0V8 | Oncology | 1.23(0.99,1.53) | 6.31E-02 | 0.439 |
| DCXR | Q7Z4W1 | Oncology | 0.82(0.66,1.01) | 6.38E-02 | 0.439 |
| PSAPL1 | Q6NUJ1 | Oncology II | 1.28(0.99,1.66) | 6.46E-02 | 0.439 |
| UBE2B | P63146 | Oncology II | 0.64(0.40,1.03) | 6.47E-02 | 0.439 |
| ANKRD54 | Q6NXT1 | Oncology | 1.18(0.99,1.41) | 6.49E-02 | 0.439 |
| STAU1 | O95793 | Oncology II | 0.80(0.63,1.01) | 6.51E-02 | 0.439 |
| CD302 | Q8IX05 | Oncology | 1.38(0.98,1.96) | 6.67E-02 | 0.439 |
| ENSA | O43768 | Oncology II | 0.77(0.59,1.02) | 6.72E-02 | 0.439 |
| TARS1 | P26639 | Oncology II | 0.71(0.49,1.03) | 6.78E-02 | 0.439 |
| SUSD4 | Q5VX71 | Oncology II | 1.32(0.98,1.77) | 6.82E-02 | 0.439 |
| SEPTIN7 | Q16181 | Oncology II | 0.74(0.54,1.02) | 6.92E-02 | 0.439 |
| DTNB | O60941 | Oncology II | 1.32(0.98,1.78) | 6.93E-02 | 0.439 |
| CHAC2 | Q8WUX2 | Oncology | 0.83(0.68,1.01) | 6.94E-02 | 0.439 |
| PTTG1 | O95997 | Oncology II | 0.72(0.50,1.03) | 6.96E-02 | 0.439 |
| ITGB5 | P18084 | Oncology | 0.74(0.54,1.02) | 7.04E-02 | 0.439 |
| NDUFB7 | P17568 | Oncology II | 0.87(0.75,1.01) | 7.04E-02 | 0.439 |
| YARS1 | P54577 | Oncology II | 0.92(0.84,1.01) | 7.10E-02 | 0.439 |
| CDHR2 | Q9BYE9 | Oncology | 0.87(0.74,1.01) | 7.16E-02 | 0.439 |
| ARHGAP30 | Q7Z6I6 | Oncology II | 0.68(0.44,1.04) | 7.21E-02 | 0.439 |
| RAB2B | Q8WUD1 | Oncology II | 0.82(0.65,1.02) | 7.27E-02 | 0.439 |
| ADM | P35318 | Oncology | 1.43(0.97,2.13) | 7.37E-02 | 0.439 |
| PODXL2 | Q9NZ53 | Oncology | 1.44(0.97,2.14) | 7.39E-02 | 0.439 |
| JPT2 | Q9H910 | Oncology II | 0.87(0.75,1.01) | 7.55E-02 | 0.439 |
| SIAE | Q9HAT2 | Oncology | 0.80(0.62,1.02) | 7.58E-02 | 0.439 |
| GADD45B | O75293 | Oncology II | 0.72(0.49,1.04) | 7.65E-02 | 0.439 |
| WFDC12 | Q8WWY7 | Oncology | 1.17(0.98,1.39) | 7.67E-02 | 0.439 |
| PRUNE2 | Q8WUY3 | Oncology II | 1.42(0.96,2.09) | 7.68E-02 | 0.439 |
| MTSS1 | O43312 | Oncology II | 0.90(0.80,1.01) | 7.72E-02 | 0.439 |
| PRKRA | O75569 | Oncology | 0.85(0.72,1.02) | 7.76E-02 | 0.439 |
| DNAJB14 | Q8TBM8 | Oncology II | 0.82(0.66,1.02) | 7.76E-02 | 0.439 |
| ZFYVE19 | Q96K21 | Oncology II | 0.88(0.76,1.01) | 7.77E-02 | 0.439 |
| RANGAP1 | P46060 | Oncology | 0.78(0.60,1.03) | 7.81E-02 | 0.439 |
| RALY | Q9UKM9 | Oncology II | 0.75(0.54,1.03) | 7.85E-02 | 0.439 |
| KIR2DS4 | P43632 | Oncology II | 1.07(0.99,1.15) | 7.94E-02 | 0.439 |
| KIR3DL1 | P43629 | Oncology | 1.08(0.99,1.18) | 7.98E-02 | 0.439 |
| USP25 | Q9UHP3 | Oncology II | 0.85(0.71,1.02) | 8.00E-02 | 0.439 |
| CD300E | Q496F6 | Oncology | 1.26(0.97,1.63) | 8.21E-02 | 0.444 |
| GIPC3 | Q8TF64 | Oncology II | 0.89(0.78,1.02) | 8.26E-02 | 0.444 |
| TOP1 | P11387 | Oncology II | 1.11(0.99,1.25) | 8.27E-02 | 0.444 |
| IL3 | P08700 | Oncology II | 1.23(0.97,1.56) | 8.49E-02 | 0.452 |
| CCL8 | P80075 | Oncology | 0.85(0.71,1.02) | 8.64E-02 | 0.457 |
| FOS | P01100 | Oncology II | 1.38(0.95,2.00) | 8.83E-02 | 0.464 |
| TG | P01266 | Oncology II | 1.10(0.99,1.23) | 8.97E-02 | 0.468 |
| SMC3 | Q9UQE7 | Oncology II | 0.78(0.59,1.04) | 9.03E-02 | 0.468 |
| SMAD1 | Q15797 | Oncology | 0.88(0.77,1.02) | 9.11E-02 | 0.468 |
| CETN3 | O15182 | Oncology II | 0.86(0.72,1.02) | 9.21E-02 | 0.470 |
| CDKN1A | P38936 | Oncology | 0.91(0.82,1.02) | 9.27E-02 | 0.470 |
| RSPO3 | Q9BXY4 | Oncology | 1.40(0.94,2.08) | 9.41E-02 | 0.472 |
| EVI2B | P34910 | Oncology II | 1.27(0.96,1.68) | 9.44E-02 | 0.472 |
| NCS1 | P62166 | Oncology | 1.40(0.94,2.07) | 9.62E-02 | 0.478 |
| NACC1 | Q96RE7 | Oncology II | 0.81(0.63,1.04) | 9.70E-02 | 0.478 |
| MEP1A | Q16819 | Oncology II | 0.85(0.71,1.03) | 9.90E-02 | 0.479 |
| LARP1 | Q6PKG0 | Oncology II | 0.83(0.66,1.04) | 9.91E-02 | 0.479 |
| CPXM1 | Q96SM3 | Oncology | 0.87(0.74,1.03) | 9.96E-02 | 0.479 |
| VSTM2B | A6NLU5 | Oncology II | 0.73(0.50,1.06) | 9.97E-02 | 0.479 |
| GALNT10 | Q86SR1 | Oncology | 0.71(0.47,1.07) | 1.02E-01 | 0.484 |
| PARD3 | Q8TEW0 | Oncology II | 0.85(0.70,1.03) | 1.02E-01 | 0.484 |
| FNTA | P49354 | Oncology II | 0.71(0.47,1.07) | 1.03E-01 | 0.485 |
| PITHD1 | Q9GZP4 | Oncology II | 1.13(0.97,1.32) | 1.06E-01 | 0.496 |
| TNFRSF19 | Q9NS68 | Oncology | 1.32(0.94,1.84) | 1.07E-01 | 0.498 |
| CLASP1 | Q7Z460 | Oncology II | 0.85(0.69,1.04) | 1.10E-01 | 0.508 |
| STAM | Q92783 | Oncology II | 0.73(0.49,1.08) | 1.12E-01 | 0.510 |
| TAB2 | Q9NYJ8 | Oncology II | 0.90(0.80,1.02) | 1.12E-01 | 0.510 |
| PAFAH2 | Q99487 | Oncology II | 0.80(0.60,1.06) | 1.14E-01 | 0.510 |
| FKBPL | Q9UIM3 | Oncology II | 0.83(0.66,1.05) | 1.15E-01 | 0.510 |
| TADA3 | O75528 | Oncology II | 0.76(0.54,1.07) | 1.15E-01 | 0.510 |
| IL12A_IL12B | P29459_P29460 | Oncology | 1.12(0.97,1.28) | 1.16E-01 | 0.510 |
| SNX2 | O60749 | Oncology II | 0.87(0.74,1.03) | 1.16E-01 | 0.510 |
| INPP1 | P49441 | Oncology | 0.84(0.67,1.05) | 1.17E-01 | 0.510 |
| CRISP2 | P16562 | Oncology | 0.86(0.72,1.04) | 1.18E-01 | 0.510 |
| ATRAID | Q6UW56 | Oncology II | 1.34(0.93,1.95) | 1.18E-01 | 0.510 |
| VPS4B | O75351 | Oncology II | 0.89(0.78,1.03) | 1.18E-01 | 0.510 |
| YES1 | P07947 | Oncology | 0.92(0.84,1.02) | 1.19E-01 | 0.511 |
| NEK7 | Q8TDX7 | Oncology II | 0.85(0.70,1.04) | 1.21E-01 | 0.517 |
| ARG2 | P78540 | Oncology II | 0.76(0.54,1.08) | 1.22E-01 | 0.518 |
| CHCHD6 | Q9BRQ6 | Oncology II | 1.37(0.92,2.06) | 1.23E-01 | 0.519 |
| AIFM1 | O95831 | Oncology | 0.92(0.82,1.02) | 1.24E-01 | 0.520 |
| CD27 | P26842 | Oncology | 1.22(0.94,1.58) | 1.26E-01 | 0.523 |
| IST1 | P53990 | Oncology II | 0.90(0.79,1.03) | 1.26E-01 | 0.523 |
| ANGPT2 | O15123 | Oncology | 1.25(0.94,1.68) | 1.28E-01 | 0.528 |
| RBP2 | P50120 | Oncology | 1.12(0.97,1.29) | 1.35E-01 | 0.541 |
| NTF4 | P34130 | Oncology | 0.76(0.53,1.09) | 1.36E-01 | 0.541 |
| PDP1 | Q9P0J1 | Oncology | 0.82(0.63,1.06) | 1.36E-01 | 0.541 |
| WIF1 | Q9Y5W5 | Oncology | 1.28(0.93,1.76) | 1.37E-01 | 0.541 |
| AHSA1 | O95433 | Oncology II | 1.15(0.96,1.39) | 1.37E-01 | 0.541 |
| TMED8 | Q6PL24 | Oncology II | 0.91(0.80,1.03) | 1.38E-01 | 0.541 |
| FOXO3 | O43524 | Oncology | 0.91(0.80,1.03) | 1.39E-01 | 0.541 |
| LYN | P07948 | Oncology | 0.90(0.79,1.03) | 1.39E-01 | 0.541 |
| EFCAB2 | Q5VUJ9 | Oncology II | 0.74(0.49,1.10) | 1.40E-01 | 0.541 |
| PTPRK | Q15262 | Oncology II | 0.60(0.31,1.18) | 1.40E-01 | 0.541 |
| SMOC1 | Q9H4F8 | Oncology | 1.28(0.92,1.79) | 1.41E-01 | 0.541 |
| STAT5B | P51692 | Oncology | 0.94(0.87,1.02) | 1.41E-01 | 0.541 |
| CMIP | Q8IY22 | Oncology II | 0.89(0.75,1.04) | 1.42E-01 | 0.541 |
| IQGAP2 | Q13576 | Oncology | 0.85(0.69,1.06) | 1.43E-01 | 0.541 |
| SHPK | Q9UHJ6 | Oncology II | 0.76(0.52,1.10) | 1.43E-01 | 0.541 |
| VWA1 | Q6PCB0 | Oncology | 0.82(0.62,1.07) | 1.44E-01 | 0.541 |
| PRTG | Q2VWP7 | Oncology | 0.72(0.46,1.12) | 1.45E-01 | 0.541 |
| VEGFC | P49767 | Oncology | 0.86(0.70,1.05) | 1.45E-01 | 0.541 |
| DNAJB1 | P25685 | Oncology | 0.91(0.80,1.03) | 1.46E-01 | 0.541 |
| VPS28 | Q9UK41 | Oncology II | 0.85(0.69,1.06) | 1.46E-01 | 0.541 |
| ERBIN | Q96RT1 | Oncology | 0.92(0.83,1.03) | 1.48E-01 | 0.542 |
| NDST1 | P52848 | Oncology II | 1.14(0.95,1.36) | 1.48E-01 | 0.542 |
| GFRA2 | O00451 | Oncology | 1.49(0.87,2.56) | 1.49E-01 | 0.542 |
| ZNF75D | P51815 | Oncology II | 1.16(0.95,1.42) | 1.50E-01 | 0.542 |
| TACC3 | Q9Y6A5 | Oncology | 0.93(0.85,1.03) | 1.51E-01 | 0.542 |
| PCSK7 | Q16549 | Oncology II | 0.79(0.57,1.09) | 1.52E-01 | 0.542 |
| TSPAN8 | P19075 | Oncology II | 0.91(0.80,1.04) | 1.52E-01 | 0.542 |
| TNFRSF12A | Q9NP84 | Oncology | 1.26(0.92,1.72) | 1.53E-01 | 0.542 |
| TRIM24 | O15164 | Oncology II | 0.86(0.69,1.06) | 1.53E-01 | 0.542 |
| RBP7 | Q96R05 | Oncology II | 1.16(0.95,1.41) | 1.55E-01 | 0.543 |
| GPC1 | P35052 | Oncology | 1.32(0.90,1.92) | 1.56E-01 | 0.543 |
| MSLN | Q13421 | Oncology | 0.87(0.71,1.06) | 1.57E-01 | 0.543 |
| NECTIN4 | Q96NY8 | Oncology | 1.28(0.91,1.79) | 1.57E-01 | 0.543 |
| TRIM25 | Q14258 | Oncology II | 0.92(0.81,1.03) | 1.57E-01 | 0.543 |
| SORD | Q00796 | Oncology | 0.89(0.75,1.05) | 1.59E-01 | 0.545 |
| LRP1 | Q07954 | Oncology | 1.35(0.89,2.03) | 1.61E-01 | 0.545 |
| NUBP1 | P53384 | Oncology II | 0.90(0.78,1.04) | 1.62E-01 | 0.545 |
| RAD23B | P54727 | Oncology | 0.83(0.64,1.08) | 1.63E-01 | 0.545 |
| VPS53 | Q5VIR6 | Oncology | 0.90(0.78,1.04) | 1.63E-01 | 0.545 |
| AP3B1 | O00203 | Oncology II | 0.88(0.74,1.05) | 1.63E-01 | 0.545 |
| SMAD3 | P84022 | Oncology II | 0.83(0.64,1.08) | 1.63E-01 | 0.545 |
| CNPY4 | Q8N129 | Oncology | 0.90(0.77,1.05) | 1.64E-01 | 0.545 |
| KDR | P35968 | Oncology | 0.73(0.47,1.14) | 1.65E-01 | 0.545 |
| MTIF3 | Q9H2K0 | Oncology II | 0.90(0.77,1.05) | 1.65E-01 | 0.545 |
| GRSF1 | Q12849 | Oncology II | 0.88(0.74,1.05) | 1.68E-01 | 0.552 |
| PPP1R12A | O14974 | Oncology | 0.92(0.82,1.04) | 1.70E-01 | 0.557 |
| RPE | Q96AT9 | Oncology II | 0.87(0.71,1.06) | 1.71E-01 | 0.557 |
| GUCY2C | P25092 | Oncology II | 1.11(0.95,1.30) | 1.75E-01 | 0.568 |
| CEP20 | Q96NB1 | Oncology | 0.90(0.77,1.05) | 1.77E-01 | 0.569 |
| PPP1R12B | O60237 | Oncology II | 0.87(0.71,1.07) | 1.77E-01 | 0.569 |
| SHC1 | P29353 | Oncology II | 1.08(0.97,1.21) | 1.79E-01 | 0.571 |
| CDH22 | Q9UJ99 | Oncology II | 0.72(0.44,1.17) | 1.80E-01 | 0.571 |
| PCYT2 | Q99447 | Oncology II | 0.89(0.75,1.06) | 1.80E-01 | 0.571 |
| BRD1 | O95696 | Oncology II | 0.72(0.45,1.17) | 1.81E-01 | 0.572 |
| CCN1 | O00622 | Oncology | 0.90(0.78,1.05) | 1.82E-01 | 0.573 |
| OGT | O15294 | Oncology II | 0.85(0.67,1.08) | 1.90E-01 | 0.595 |
| KLK8 | O60259 | Oncology | 1.23(0.90,1.67) | 1.92E-01 | 0.596 |
| TPRKB | Q9Y3C4 | Oncology II | 0.86(0.68,1.08) | 1.96E-01 | 0.596 |
| CEACAM1 | P13688 | Oncology | 0.73(0.45,1.18) | 1.98E-01 | 0.596 |
| ACOT13 | Q9NPJ3 | Oncology II | 0.93(0.82,1.04) | 1.98E-01 | 0.596 |
| TCP11 | Q8WWU5 | Oncology II | 0.83(0.62,1.10) | 1.98E-01 | 0.596 |
| CDKN2D | P55273 | Oncology | 0.93(0.82,1.04) | 2.00E-01 | 0.596 |
| DAB2 | P98082 | Oncology | 0.94(0.85,1.04) | 2.00E-01 | 0.596 |
| TFPI2 | P48307 | Oncology | 1.19(0.91,1.54) | 2.00E-01 | 0.596 |
| BSND | Q8WZ55 | Oncology II | 0.75(0.48,1.16) | 2.00E-01 | 0.596 |
| GAS2 | O43903 | Oncology II | 0.82(0.61,1.11) | 2.00E-01 | 0.596 |
| LYPD3 | O95274 | Oncology | 1.24(0.89,1.74) | 2.01E-01 | 0.596 |
| DUSP13 | Q6B8I1 | Oncology II | 0.81(0.58,1.12) | 2.02E-01 | 0.596 |
| KLK1 | P06870 | Oncology | 1.06(0.97,1.15) | 2.03E-01 | 0.596 |
| NAMPT | P43490 | Oncology | 1.14(0.93,1.38) | 2.05E-01 | 0.596 |
| MPI | P34949 | Oncology | 0.92(0.80,1.05) | 2.09E-01 | 0.596 |
| RRM2B | Q7LG56 | Oncology | 0.89(0.75,1.06) | 2.09E-01 | 0.596 |
| UBAC1 | Q9BSL1 | Oncology | 0.81(0.59,1.13) | 2.13E-01 | 0.596 |
| ZNF830 | Q96NB3 | Oncology II | 0.89(0.73,1.07) | 2.13E-01 | 0.596 |
| WFDC1 | Q9HC57 | Oncology II | 0.83(0.62,1.11) | 2.14E-01 | 0.596 |
| HBEGF | Q99075 | Oncology | 0.91(0.78,1.06) | 2.17E-01 | 0.596 |
| MTHFSD | Q2M296 | Oncology II | 0.93(0.82,1.05) | 2.17E-01 | 0.596 |
| CD28 | P10747 | Oncology | 1.21(0.89,1.63) | 2.20E-01 | 0.596 |
| SLAMF6 | Q96DU3 | Oncology | 1.20(0.90,1.61) | 2.21E-01 | 0.596 |
| TMPRSS15 | P98073 | Oncology | 0.93(0.82,1.05) | 2.21E-01 | 0.596 |
| MTUS1 | Q9ULD2 | Oncology II | 0.85(0.66,1.10) | 2.21E-01 | 0.596 |
| PSMC3 | P17980 | Oncology II | 1.28(0.86,1.89) | 2.21E-01 | 0.596 |
| MINDY1 | Q8N5J2 | Oncology II | 0.93(0.82,1.05) | 2.22E-01 | 0.596 |
| TRDMT1 | O14717 | Oncology II | 0.90(0.77,1.06) | 2.22E-01 | 0.596 |
| ERBB4 | Q15303 | Oncology | 1.34(0.84,2.15) | 2.23E-01 | 0.596 |
| FAM3D | Q96BQ1 | Oncology II | 1.10(0.94,1.28) | 2.23E-01 | 0.596 |
| KAZN | Q674X7 | Oncology II | 0.93(0.82,1.05) | 2.23E-01 | 0.596 |
| KIR2DL2 | P43627 | Oncology II | 1.09(0.95,1.27) | 2.23E-01 | 0.596 |
| VCPKMT | Q9H867 | Oncology II | 1.09(0.95,1.24) | 2.23E-01 | 0.596 |
| FLI1 | Q01543 | Oncology | 0.94(0.84,1.04) | 2.24E-01 | 0.596 |
| MAGED1 | Q9Y5V3 | Oncology | 0.86(0.67,1.10) | 2.24E-01 | 0.596 |
| SNAP29 | O95721 | Oncology | 0.93(0.82,1.05) | 2.24E-01 | 0.596 |
| NFU1 | Q9UMS0 | Oncology II | 0.92(0.81,1.05) | 2.24E-01 | 0.596 |
| KIR2DL3 | P43628 | Oncology | 1.09(0.95,1.26) | 2.25E-01 | 0.596 |
| SLK | Q9H2G2 | Oncology II | 0.89(0.73,1.08) | 2.26E-01 | 0.596 |
| PHLDB2 | Q86SQ0 | Oncology II | 0.91(0.78,1.06) | 2.28E-01 | 0.596 |
| RFC4 | P35249 | Oncology II | 0.67(0.34,1.29) | 2.28E-01 | 0.596 |
| NFKB2 | Q00653 | Oncology II | 0.84(0.63,1.12) | 2.29E-01 | 0.596 |
| CREB3 | O43889 | Oncology II | 1.14(0.92,1.42) | 2.30E-01 | 0.596 |
| ANGPTL7 | O43827 | Oncology | 1.21(0.88,1.66) | 2.32E-01 | 0.596 |
| SFTPA1 | Q8IWL2 | Oncology | 1.12(0.93,1.35) | 2.32E-01 | 0.596 |
| COL4A4 | P53420 | Oncology II | 0.87(0.68,1.10) | 2.32E-01 | 0.596 |
| TDP1 | Q9NUW8 | Oncology II | 0.88(0.71,1.09) | 2.32E-01 | 0.596 |
| ATP6V1D | Q9Y5K8 | Oncology | 0.75(0.47,1.20) | 2.33E-01 | 0.596 |
| TAGLN3 | Q9UI15 | Oncology II | 0.87(0.68,1.10) | 2.33E-01 | 0.596 |
| VTCN1 | Q7Z7D3 | Oncology | 1.26(0.86,1.83) | 2.34E-01 | 0.596 |
| STX16 | O14662 | Oncology | 0.88(0.71,1.09) | 2.35E-01 | 0.596 |
| LRRFIP1 | Q32MZ4 | Oncology II | 0.85(0.64,1.12) | 2.36E-01 | 0.596 |
| SERPINH1 | P50454 | Oncology II | 0.94(0.85,1.04) | 2.36E-01 | 0.596 |
| KIFBP | Q96EK5 | Oncology | 0.92(0.80,1.06) | 2.37E-01 | 0.596 |
| FGF23 | Q9GZV9 | Oncology | 0.87(0.69,1.10) | 2.38E-01 | 0.596 |
| TBL1X | O60907 | Oncology | 0.92(0.80,1.06) | 2.38E-01 | 0.596 |
| ALPP | P05187 | Oncology | 0.94(0.85,1.04) | 2.40E-01 | 0.596 |
| FLT3 | P36888 | Oncology | 1.22(0.88,1.70) | 2.40E-01 | 0.596 |
| SIRT2 | Q8IXJ6 | Oncology | 0.93(0.81,1.05) | 2.40E-01 | 0.596 |
| CCND2 | P30279 | Oncology II | 0.81(0.56,1.15) | 2.40E-01 | 0.596 |
| ATG16L1 | Q676U5 | Oncology II | 0.91(0.78,1.06) | 2.41E-01 | 0.596 |
| GPR15L | Q6UWK7 | Oncology II | 1.13(0.92,1.40) | 2.41E-01 | 0.596 |
| RTBDN | Q9BSG5 | Oncology | 0.79(0.54,1.17) | 2.44E-01 | 0.600 |
| DNAJA1 | P31689 | Oncology II | 0.83(0.60,1.14) | 2.45E-01 | 0.600 |
| SH3BP1 | Q9Y3L3 | Oncology II | 0.84(0.63,1.13) | 2.45E-01 | 0.600 |
| ENOPH1 | Q9UHY7 | Oncology II | 0.85(0.65,1.12) | 2.47E-01 | 0.601 |
| LAMTOR5 | O43504 | Oncology II | 0.81(0.57,1.15) | 2.47E-01 | 0.601 |
| PSMA1 | P25786 | Oncology | 0.85(0.64,1.12) | 2.52E-01 | 0.606 |
| FGF9 | P31371 | Oncology II | 0.82(0.59,1.15) | 2.52E-01 | 0.606 |
| METAP2 | P50579 | Oncology | 0.89(0.74,1.08) | 2.53E-01 | 0.606 |
| GSTA3 | Q16772 | Oncology | 0.92(0.79,1.06) | 2.55E-01 | 0.606 |
| TJAP1 | Q5JTD0 | Oncology | 0.92(0.81,1.06) | 2.57E-01 | 0.606 |
| VPS37A | Q8NEZ2 | Oncology | 0.92(0.80,1.06) | 2.57E-01 | 0.606 |
| MORN4 | Q8NDC4 | Oncology II | 0.89(0.73,1.09) | 2.57E-01 | 0.606 |
| DDX58 | O95786 | Oncology | 1.12(0.92,1.36) | 2.58E-01 | 0.606 |
| FUS | P35637 | Oncology | 0.84(0.62,1.14) | 2.58E-01 | 0.606 |
| SCP2 | P22307 | Oncology | 0.91(0.76,1.08) | 2.58E-01 | 0.606 |
| AMIGO2 | Q86SJ2 | Oncology | 0.75(0.46,1.23) | 2.60E-01 | 0.606 |
| S100A4 | P26447 | Oncology | 0.89(0.73,1.09) | 2.60E-01 | 0.606 |
| FAM13A | O94988 | Oncology II | 0.89(0.73,1.09) | 2.60E-01 | 0.606 |
| S100A3 | P33764 | Oncology II | 0.81(0.56,1.17) | 2.62E-01 | 0.608 |
| SUGT1 | Q9Y2Z0 | Oncology | 0.92(0.80,1.06) | 2.63E-01 | 0.608 |
| EVPL | Q92817 | Oncology II | 0.77(0.49,1.21) | 2.63E-01 | 0.608 |
| COX5B | P10606 | Oncology | 0.91(0.76,1.08) | 2.65E-01 | 0.608 |
| TMEM106A | Q96A25 | Oncology II | 0.90(0.74,1.09) | 2.65E-01 | 0.608 |
| OTUD6B | Q8N6M0 | Oncology II | 0.88(0.70,1.10) | 2.67E-01 | 0.611 |
| GFER | P55789 | Oncology | 0.90(0.75,1.08) | 2.71E-01 | 0.618 |
| XCL1 | P47992 | Oncology | 1.12(0.92,1.36) | 2.76E-01 | 0.624 |
| SPRING1 | Q9H741 | Oncology II | 0.87(0.67,1.12) | 2.76E-01 | 0.624 |
| NUDT2 | P50583 | Oncology | 0.89(0.72,1.10) | 2.77E-01 | 0.624 |
| CTAG1A_CTAG1B | P78358 | Oncology II | 0.85(0.64,1.14) | 2.77E-01 | 0.624 |
| SDCCAG8 | Q86SQ7 | Oncology II | 0.94(0.84,1.05) | 2.78E-01 | 0.624 |
| SERPINI2 | O75830 | Oncology II | 1.14(0.90,1.44) | 2.80E-01 | 0.625 |
| AKR1B1 | P15121 | Oncology | 0.92(0.78,1.07) | 2.81E-01 | 0.625 |
| PTPRH | Q9HD43 | Oncology II | 1.16(0.89,1.51) | 2.83E-01 | 0.625 |
| ACP6 | Q9NPH0 | Oncology | 0.92(0.79,1.07) | 2.84E-01 | 0.625 |
| NENF | Q9UMX5 | Oncology II | 0.93(0.82,1.06) | 2.85E-01 | 0.625 |
| DNM1 | Q05193 | Oncology II | 0.94(0.84,1.05) | 2.86E-01 | 0.625 |
| RGL2 | O15211 | Oncology II | 0.88(0.69,1.11) | 2.86E-01 | 0.625 |
| CDC42BPB | Q9Y5S2 | Oncology II | 0.93(0.82,1.06) | 2.87E-01 | 0.625 |
| KLK13 | Q9UKR3 | Oncology | 1.15(0.89,1.47) | 2.90E-01 | 0.625 |
| MSRA | Q9UJ68 | Oncology | 0.92(0.78,1.08) | 2.90E-01 | 0.625 |
| NT5E | P21589 | Oncology | 0.89(0.73,1.10) | 2.91E-01 | 0.625 |
| LRRC37A2 | A6NM11 | Oncology II | 0.85(0.63,1.15) | 2.91E-01 | 0.625 |
| LSM1 | O15116 | Oncology | 0.89(0.71,1.11) | 2.93E-01 | 0.625 |
| AP1G2 | O75843 | Oncology II | 0.91(0.77,1.08) | 2.94E-01 | 0.625 |
| NUCB2 | P80303 | Oncology | 0.85(0.62,1.16) | 2.95E-01 | 0.625 |
| ADGRE1 | Q14246 | Oncology II | 1.13(0.90,1.43) | 2.95E-01 | 0.625 |
| HGS | O14964 | Oncology | 0.92(0.79,1.07) | 2.96E-01 | 0.625 |
| LAG3 | P18627 | Oncology | 0.86(0.64,1.15) | 2.96E-01 | 0.625 |
| ATG4A | Q8WYN0 | Oncology | 0.90(0.74,1.10) | 2.97E-01 | 0.625 |
| SRC | P12931 | Oncology | 0.95(0.87,1.04) | 2.97E-01 | 0.625 |
| CYB5R2 | Q6BCY4 | Oncology II | 0.88(0.69,1.12) | 2.97E-01 | 0.625 |
| SSNA1 | O43805 | Oncology II | 0.87(0.67,1.13) | 2.97E-01 | 0.625 |
| CFC1 | P0CG37 | Oncology | 1.13(0.89,1.43) | 3.04E-01 | 0.635 |
| POLR2F | P61218 | Oncology | 1.17(0.86,1.59) | 3.06E-01 | 0.635 |
| MAPK13 | O15264 | Oncology II | 1.17(0.86,1.59) | 3.06E-01 | 0.635 |
| MORF4L1 | Q9UBU8 | Oncology II | 1.17(0.87,1.58) | 3.06E-01 | 0.635 |
| PDIA2 | Q13087 | Oncology II | 1.17(0.86,1.59) | 3.06E-01 | 0.635 |
| USO1 | O60763 | Oncology | 0.94(0.83,1.06) | 3.11E-01 | 0.643 |
| FGF21 | Q9NSA1 | Oncology | 0.95(0.87,1.05) | 3.16E-01 | 0.650 |
| PPM1A | P35813 | Oncology | 0.82(0.56,1.21) | 3.16E-01 | 0.650 |
| ZNRF4 | Q8WWF5 | Oncology II | 0.88(0.68,1.13) | 3.17E-01 | 0.650 |
| CCT5 | P48643 | Oncology | 0.87(0.67,1.14) | 3.25E-01 | 0.658 |
| GABARAP | O95166 | Oncology II | 0.86(0.65,1.16) | 3.26E-01 | 0.658 |
| DKKL1 | Q9UK85 | Oncology | 1.07(0.94,1.22) | 3.27E-01 | 0.658 |
| DCDC2C | A8MYV0 | Oncology II | 1.11(0.90,1.36) | 3.27E-01 | 0.658 |
| FUT1 | P19526 | Oncology II | 0.89(0.70,1.13) | 3.28E-01 | 0.658 |
| RNF4 | P78317 | Oncology II | 0.90(0.72,1.11) | 3.28E-01 | 0.658 |
| CTSF | Q9UBX1 | Oncology | 0.87(0.65,1.15) | 3.30E-01 | 0.658 |
| LTBP3 | Q9NS15 | Oncology | 0.89(0.71,1.12) | 3.30E-01 | 0.658 |
| GPHA2 | Q96T91 | Oncology II | 1.14(0.87,1.49) | 3.30E-01 | 0.658 |
| IL25 | Q9H293 | Oncology II | 1.22(0.82,1.81) | 3.30E-01 | 0.658 |
| LMNB1 | P20700 | Oncology II | 1.11(0.90,1.37) | 3.31E-01 | 0.658 |
| ERN1 | O75460 | Oncology II | 0.84(0.58,1.20) | 3.33E-01 | 0.661 |
| ATOX1 | O00244 | Oncology | 0.92(0.79,1.08) | 3.34E-01 | 0.661 |
| LYAR | Q9NX58 | Oncology | 1.10(0.91,1.32) | 3.39E-01 | 0.669 |
| GH2 | P01242 | Oncology | 0.96(0.90,1.04) | 3.42E-01 | 0.673 |
| DEFB4A_DEFB4B | O15263 | Oncology | 0.96(0.89,1.04) | 3.43E-01 | 0.673 |
| CPLX2 | Q6PUV4 | Oncology II | 1.19(0.83,1.71) | 3.46E-01 | 0.677 |
| FCRLB | Q6BAA4 | Oncology | 1.09(0.91,1.30) | 3.50E-01 | 0.683 |
| SPINK6 | Q6UWN8 | Oncology | 0.90(0.73,1.12) | 3.51E-01 | 0.683 |
| BCL2 | P10415 | Oncology II | 0.88(0.68,1.15) | 3.52E-01 | 0.683 |
| KLK11 | Q9UBX7 | Oncology | 1.18(0.83,1.66) | 3.54E-01 | 0.684 |
| COL24A1 | Q17RW2 | Oncology II | 0.90(0.72,1.12) | 3.54E-01 | 0.684 |
| CBLN4 | Q9NTU7 | Oncology | 0.85(0.59,1.21) | 3.57E-01 | 0.686 |
| CA7 | P43166 | Oncology II | 0.82(0.55,1.24) | 3.57E-01 | 0.686 |
| IGSF3 | O75054 | Oncology | 0.86(0.62,1.19) | 3.62E-01 | 0.694 |
| UNG | P13051 | Oncology II | 0.83(0.56,1.24) | 3.64E-01 | 0.696 |
| EPS8L2 | Q9H6S3 | Oncology | 0.84(0.58,1.23) | 3.71E-01 | 0.707 |
| CEACAM16 | Q2WEN9 | Oncology II | 1.08(0.91,1.28) | 3.72E-01 | 0.707 |
| COMMD9 | Q9P000 | Oncology II | 0.85(0.60,1.21) | 3.78E-01 | 0.715 |
| RET | P07949 | Oncology | 0.88(0.66,1.17) | 3.80E-01 | 0.715 |
| TBC1D23 | Q9NUY8 | Oncology | 0.95(0.83,1.07) | 3.80E-01 | 0.715 |
| GABARAPL1 | Q9H0R8 | Oncology II | 0.91(0.73,1.13) | 3.80E-01 | 0.715 |
| AMBP | P02760 | Oncology | 1.27(0.74,2.19) | 3.82E-01 | 0.717 |
| CDC26 | Q8NHZ8 | Oncology II | 0.92(0.76,1.11) | 3.84E-01 | 0.719 |
| CEP350 | Q5VT06 | Oncology II | 0.85(0.59,1.23) | 3.86E-01 | 0.721 |
| VMO1 | Q7Z5L0 | Oncology | 1.08(0.91,1.28) | 3.87E-01 | 0.721 |
| SF3B4 | Q15427 | Oncology | 0.93(0.80,1.09) | 3.88E-01 | 0.721 |
| TMOD4 | Q9NZQ9 | Oncology II | 1.15(0.84,1.59) | 3.90E-01 | 0.723 |
| GRPEL1 | Q9HAV7 | Oncology | 0.91(0.75,1.12) | 3.91E-01 | 0.723 |
| LTA4H | P09960 | Oncology | 0.92(0.76,1.12) | 3.94E-01 | 0.723 |
| TXNDC15 | Q96J42 | Oncology | 1.19(0.79,1.80) | 3.94E-01 | 0.723 |
| BTC | P35070 | Oncology | 0.93(0.79,1.10) | 3.95E-01 | 0.723 |
| MED18 | Q9BUE0 | Oncology | 0.94(0.80,1.09) | 3.95E-01 | 0.723 |
| L1CAM | P32004 | Oncology | 1.18(0.80,1.73) | 3.98E-01 | 0.723 |
| SFTPA2 | Q8IWL1 | Oncology | 1.10(0.89,1.36) | 3.98E-01 | 0.723 |
| PKN3 | Q6P5Z2 | Oncology II | 0.92(0.77,1.11) | 3.98E-01 | 0.723 |
| AARSD1 | Q9BTE6 | Oncology | 0.92(0.75,1.12) | 4.02E-01 | 0.728 |
| DNAJC9 | Q8WXX5 | Oncology II | 0.93(0.78,1.10) | 4.04E-01 | 0.730 |
| NMI | Q13287 | Oncology II | 1.08(0.90,1.30) | 4.05E-01 | 0.730 |
| SWAP70 | Q9UH65 | Oncology II | 0.85(0.58,1.25) | 4.07E-01 | 0.732 |
| TPPP2 | P59282 | Oncology II | 1.07(0.91,1.26) | 4.09E-01 | 0.734 |
| EFCAB14 | O75071 | Oncology II | 1.19(0.79,1.78) | 4.11E-01 | 0.734 |
| UPK3BL1 | B0FP48 | Oncology II | 0.89(0.68,1.17) | 4.11E-01 | 0.734 |
| BIRC2 | Q13490 | Oncology | 0.93(0.77,1.11) | 4.16E-01 | 0.741 |
| NUDT16 | Q96DE0 | Oncology II | 0.94(0.80,1.09) | 4.18E-01 | 0.741 |
| YJU2 | Q9BW85 | Oncology II | 0.84(0.55,1.28) | 4.18E-01 | 0.741 |
| CDC25A | P30304 | Oncology II | 1.15(0.82,1.59) | 4.19E-01 | 0.741 |
| SCAMP3 | O14828 | Oncology | 0.95(0.83,1.08) | 4.21E-01 | 0.742 |
| C4BPB | P20851 | Oncology | 1.12(0.84,1.50) | 4.23E-01 | 0.744 |
| CDC37 | Q16543 | Oncology | 0.95(0.83,1.08) | 4.26E-01 | 0.746 |
| PDCL2 | Q8N4E4 | Oncology II | 1.10(0.87,1.39) | 4.26E-01 | 0.746 |
| DPP6 | P42658 | Oncology | 0.87(0.63,1.22) | 4.28E-01 | 0.747 |
| TEK | Q02763 | Oncology | 1.23(0.73,2.07) | 4.31E-01 | 0.749 |
| EPN1 | Q9Y6I3 | Oncology II | 0.87(0.62,1.23) | 4.31E-01 | 0.749 |
| TAFA5 | Q7Z5A7 | Oncology | 1.13(0.83,1.53) | 4.34E-01 | 0.752 |
| SIGLEC6 | O43699 | Oncology | 0.87(0.63,1.22) | 4.35E-01 | 0.752 |
| FOLR1 | P15328 | Oncology | 1.17(0.79,1.72) | 4.36E-01 | 0.752 |
| MAP2K1 | Q02750 | Oncology II | 0.92(0.75,1.13) | 4.40E-01 | 0.754 |
| SUGP1 | Q8IWZ8 | Oncology II | 0.87(0.62,1.23) | 4.40E-01 | 0.754 |
| MORF4L2 | Q15014 | Oncology II | 0.86(0.59,1.26) | 4.41E-01 | 0.754 |
| RNF43 | Q68DV7 | Oncology II | 0.90(0.70,1.17) | 4.41E-01 | 0.754 |
| IFNAR1 | P17181 | Oncology II | 0.82(0.49,1.37) | 4.42E-01 | 0.754 |
| OGFR | Q9NZT2 | Oncology | 0.87(0.61,1.24) | 4.43E-01 | 0.754 |
| CDC123 | O75794 | Oncology II | 0.91(0.72,1.15) | 4.47E-01 | 0.759 |
| PQBP1 | O60828 | Oncology | 0.92(0.74,1.14) | 4.49E-01 | 0.759 |
| CYTH3 | O43739 | Oncology II | 1.07(0.90,1.28) | 4.49E-01 | 0.759 |
| CASP8 | Q14790 | Oncology | 0.93(0.77,1.12) | 4.50E-01 | 0.759 |
| FGFBP1 | Q14512 | Oncology | 1.14(0.81,1.61) | 4.58E-01 | 0.770 |
| BCL7B | Q9BQE9 | Oncology II | 0.89(0.65,1.21) | 4.60E-01 | 0.772 |
| PFKFB2 | O60825 | Oncology | 0.95(0.82,1.10) | 4.63E-01 | 0.774 |
| LYZL2 | Q7Z4W2 | Oncology II | 1.14(0.80,1.63) | 4.64E-01 | 0.774 |
| DPEP2 | Q9H4A9 | Oncology | 0.86(0.58,1.28) | 4.65E-01 | 0.774 |
| DCBLD2 | Q96PD2 | Oncology | 1.13(0.81,1.56) | 4.66E-01 | 0.774 |
| CD5 | P06127 | Oncology | 1.11(0.83,1.50) | 4.69E-01 | 0.774 |
| PFDN4 | Q9NQP4 | Oncology II | 0.90(0.69,1.19) | 4.69E-01 | 0.774 |
| ACAA1 | P09110 | Oncology | 0.95(0.82,1.10) | 4.72E-01 | 0.774 |
| PDCD1 | Q15116 | Oncology | 1.10(0.85,1.41) | 4.72E-01 | 0.774 |
| GIMAP8 | Q8ND71 | Oncology II | 1.10(0.85,1.41) | 4.72E-01 | 0.774 |
| ADAMTS15 | Q8TE58 | Oncology | 0.92(0.72,1.16) | 4.73E-01 | 0.774 |
| FXN | Q16595 | Oncology | 0.94(0.81,1.10) | 4.73E-01 | 0.774 |
| HS3ST3B1 | Q9Y662 | Oncology | 0.90(0.66,1.21) | 4.75E-01 | 0.774 |
| VAT1 | Q99536 | Oncology | 1.23(0.69,2.20) | 4.75E-01 | 0.774 |
| VSIG10 | Q8N0Z9 | Oncology II | 0.86(0.57,1.30) | 4.80E-01 | 0.778 |
| NMRK2 | Q9NPI5 | Oncology II | 0.87(0.60,1.27) | 4.83E-01 | 0.778 |
| ITGB7 | P26010 | Oncology | 0.91(0.71,1.17) | 4.84E-01 | 0.778 |
| JMJD1C | Q15652 | Oncology II | 0.91(0.71,1.18) | 4.84E-01 | 0.778 |
| PBXIP1 | Q96AQ6 | Oncology II | 1.16(0.76,1.78) | 4.84E-01 | 0.778 |
| TRIM26 | Q12899 | Oncology II | 0.91(0.71,1.18) | 4.84E-01 | 0.778 |
| SLC28A1 | O00337 | Oncology II | 0.92(0.72,1.17) | 4.88E-01 | 0.783 |
| PCDHB15 | Q9Y5E8 | Oncology II | 1.12(0.81,1.55) | 4.89E-01 | 0.783 |
| GFOD2 | Q3B7J2 | Oncology | 0.97(0.88,1.07) | 4.92E-01 | 0.786 |
| DCUN1D2 | Q6PH85 | Oncology II | 0.94(0.79,1.12) | 4.93E-01 | 0.786 |
| PPY | P01298 | Oncology | 1.04(0.93,1.16) | 4.94E-01 | 0.786 |
| CA11 | O75493 | Oncology | 0.87(0.59,1.29) | 4.97E-01 | 0.788 |
| CCN4 | O95388 | Oncology | 1.10(0.84,1.45) | 4.98E-01 | 0.788 |
| REST | Q13127 | Oncology II | 0.87(0.57,1.32) | 5.00E-01 | 0.788 |
| TMED1 | Q13445 | Oncology II | 0.91(0.71,1.18) | 5.00E-01 | 0.788 |
| CES2 | O00748 | Oncology | 0.93(0.76,1.15) | 5.06E-01 | 0.793 |
| PLA2G15 | Q8NCC3 | Oncology | 0.87(0.57,1.32) | 5.06E-01 | 0.793 |
| CEACAM20 | Q6UY09 | Oncology II | 0.90(0.65,1.23) | 5.06E-01 | 0.793 |
| GSAP | A4D1B5 | Oncology | 1.04(0.92,1.19) | 5.07E-01 | 0.793 |
| INPPL1 | O15357 | Oncology | 0.96(0.86,1.08) | 5.09E-01 | 0.794 |
| ACADM | P11310 | Oncology II | 0.93(0.76,1.14) | 5.12E-01 | 0.794 |
| RNF41 | Q9H4P4 | Oncology | 0.94(0.79,1.12) | 5.14E-01 | 0.794 |
| SAT2 | Q96F10 | Oncology II | 1.06(0.88,1.28) | 5.14E-01 | 0.794 |
| TNPO1 | Q92973 | Oncology II | 0.88(0.59,1.30) | 5.14E-01 | 0.794 |
| SLC9A3R2 | Q15599 | Oncology II | 0.91(0.69,1.20) | 5.17E-01 | 0.794 |
| BGN | P21810 | Oncology | 1.03(0.94,1.14) | 5.19E-01 | 0.794 |
| CA14 | Q9ULX7 | Oncology | 0.90(0.66,1.23) | 5.19E-01 | 0.794 |
| MUC16 | Q8WXI7 | Oncology | 0.98(0.93,1.04) | 5.19E-01 | 0.794 |
| DCLRE1C | Q96SD1 | Oncology II | 1.15(0.75,1.76) | 5.19E-01 | 0.794 |
| LPCAT2 | Q7L5N7 | Oncology | 1.09(0.84,1.40) | 5.25E-01 | 0.801 |
| HAVCR1 | Q96D42 | Oncology | 1.06(0.89,1.26) | 5.26E-01 | 0.801 |
| BEX3 | Q00994 | Oncology II | 0.91(0.66,1.23) | 5.27E-01 | 0.801 |
| TRIAP1 | O43715 | Oncology | 0.94(0.78,1.13) | 5.29E-01 | 0.803 |
| KIAA1549L | Q6ZVL6 | Oncology II | 1.16(0.73,1.86) | 5.30E-01 | 0.803 |
| ENTPD2 | Q9Y5L3 | Oncology | 0.92(0.70,1.21) | 5.31E-01 | 0.803 |
| RASSF2 | P50749 | Oncology | 1.06(0.88,1.29) | 5.33E-01 | 0.804 |
| IMMT | Q16891 | Oncology II | 1.09(0.82,1.46) | 5.38E-01 | 0.804 |
| NAP1L4 | Q99733 | Oncology II | 0.91(0.67,1.24) | 5.41E-01 | 0.804 |
| FES | P07332 | Oncology | 0.93(0.72,1.19) | 5.42E-01 | 0.804 |
| FGFR2 | P21802 | Oncology | 1.17(0.71,1.94) | 5.42E-01 | 0.804 |
| RRM2 | P31350 | Oncology | 1.07(0.87,1.31) | 5.43E-01 | 0.804 |
| RTN4R | Q9BZR6 | Oncology | 0.91(0.67,1.23) | 5.43E-01 | 0.804 |
| C9orf40 | Q8IXQ3 | Oncology II | 1.05(0.90,1.23) | 5.43E-01 | 0.804 |
| SCG2 | P13521 | Oncology | 0.90(0.64,1.27) | 5.45E-01 | 0.804 |
| LUZP2 | Q86TE4 | Oncology II | 1.09(0.82,1.45) | 5.45E-01 | 0.804 |
| OGA | O60502 | Oncology II | 0.94(0.78,1.14) | 5.45E-01 | 0.804 |
| PCDH9 | Q9HC56 | Oncology II | 1.13(0.77,1.66) | 5.45E-01 | 0.804 |
| PSRC1 | Q6PGN9 | Oncology | 0.94(0.77,1.15) | 5.49E-01 | 0.808 |
| FLT4 | P35916 | Oncology | 0.90(0.65,1.26) | 5.51E-01 | 0.809 |
| SEZ6L | Q9BYH1 | Oncology | 1.13(0.76,1.68) | 5.54E-01 | 0.812 |
| PPP2R5A | Q15172 | Oncology II | 0.97(0.86,1.08) | 5.57E-01 | 0.815 |
| PXDNL | A1KZ92 | Oncology II | 0.92(0.71,1.20) | 5.58E-01 | 0.815 |
| MUCL3 | Q3MIW9 | Oncology II | 0.90(0.63,1.28) | 5.60E-01 | 0.816 |
| KLK15 | Q9H2R5 | Oncology II | 0.93(0.72,1.19) | 5.61E-01 | 0.816 |
| ADCYAP1R1 | P41586 | Oncology | 1.09(0.82,1.45) | 5.63E-01 | 0.817 |
| SLIRP | Q9GZT3 | Oncology II | 1.07(0.85,1.36) | 5.67E-01 | 0.821 |
| CYB5A | P00167 | Oncology II | 0.94(0.77,1.15) | 5.72E-01 | 0.824 |
| GNE | Q9Y223 | Oncology | 0.95(0.80,1.14) | 5.74E-01 | 0.824 |
| RP2 | O75695 | Oncology | 1.08(0.82,1.42) | 5.74E-01 | 0.824 |
| STX6 | O43752 | Oncology | 0.95(0.79,1.14) | 5.74E-01 | 0.824 |
| TK1 | P04183 | Oncology II | 0.91(0.66,1.26) | 5.75E-01 | 0.824 |
| INSL5 | Q9Y5Q6 | Oncology II | 0.96(0.84,1.10) | 5.77E-01 | 0.824 |
| ELOA | Q14241 | Oncology | 0.94(0.75,1.17) | 5.78E-01 | 0.824 |
| MOG | Q16653 | Oncology | 0.91(0.65,1.27) | 5.78E-01 | 0.824 |
| SEPTIN9 | Q9UHD8 | Oncology | 0.89(0.59,1.34) | 5.79E-01 | 0.824 |
| CDC27 | P30260 | Oncology | 0.95(0.78,1.15) | 5.81E-01 | 0.825 |
| BAMBI | Q13145 | Oncology | 1.12(0.75,1.66) | 5.83E-01 | 0.826 |
| CEP152 | O94986 | Oncology II | 0.91(0.65,1.27) | 5.95E-01 | 0.841 |
| CDNF | Q49AH0 | Oncology | 0.91(0.63,1.30) | 5.96E-01 | 0.841 |
| OFD1 | O75665 | Oncology II | 1.05(0.87,1.29) | 5.97E-01 | 0.841 |
| HS6ST1 | O60243 | Oncology | 0.92(0.66,1.28) | 6.03E-01 | 0.843 |
| ATP1B4 | Q9UN42 | Oncology II | 1.09(0.80,1.48) | 6.03E-01 | 0.843 |
| CWC15 | Q9P013 | Oncology II | 0.94(0.75,1.18) | 6.04E-01 | 0.843 |
| F3 | P13726 | Oncology | 1.12(0.74,1.70) | 6.05E-01 | 0.843 |
| PRC1 | O43663 | Oncology II | 0.92(0.66,1.28) | 6.05E-01 | 0.843 |
| LSM8 | O95777 | Oncology II | 0.96(0.82,1.13) | 6.07E-01 | 0.843 |
| CALCOCO1 | Q9P1Z2 | Oncology | 0.97(0.87,1.08) | 6.08E-01 | 0.843 |
| HAGH | Q16775 | Oncology | 1.04(0.89,1.21) | 6.09E-01 | 0.843 |
| LRIG1 | Q96JA1 | Oncology | 1.09(0.79,1.50) | 6.10E-01 | 0.843 |
| SPARC | P09486 | Oncology | 0.96(0.82,1.13) | 6.10E-01 | 0.843 |
| SIGLEC9 | Q9Y336 | Oncology | 1.11(0.75,1.63) | 6.11E-01 | 0.843 |
| DSG3 | P32926 | Oncology | 1.09(0.78,1.50) | 6.17E-01 | 0.849 |
| CTSV | O60911 | Oncology | 0.93(0.71,1.23) | 6.18E-01 | 0.849 |
| DUT | P33316 | Oncology II | 0.97(0.85,1.10) | 6.19E-01 | 0.849 |
| CPVL | Q9H3G5 | Oncology | 1.07(0.81,1.42) | 6.21E-01 | 0.851 |
| KRT18 | P05783 | Oncology | 0.97(0.84,1.11) | 6.23E-01 | 0.852 |
| CENPF | P49454 | Oncology II | 0.94(0.74,1.20) | 6.32E-01 | 0.860 |
| HRAS | P01112 | Oncology II | 0.97(0.84,1.12) | 6.33E-01 | 0.860 |
| ITIH5 | Q86UX2 | Oncology II | 0.93(0.68,1.26) | 6.33E-01 | 0.860 |
| SLITRK2 | Q9H156 | Oncology | 1.07(0.81,1.41) | 6.34E-01 | 0.860 |
| MAVS | Q7Z434 | Oncology | 0.97(0.86,1.10) | 6.36E-01 | 0.860 |
| CLINT1 | Q14677 | Oncology II | 1.07(0.81,1.42) | 6.36E-01 | 0.860 |
| BTNL10 | A8MVZ5 | Oncology II | 0.92(0.65,1.30) | 6.38E-01 | 0.861 |
| DHPS | P49366 | Oncology II | 1.08(0.79,1.46) | 6.40E-01 | 0.862 |
| SORCS2 | Q96PQ0 | Oncology | 1.08(0.78,1.50) | 6.43E-01 | 0.863 |
| LTB | Q06643 | Oncology II | 1.08(0.77,1.53) | 6.43E-01 | 0.863 |
| SH2B3 | Q9UQQ2 | Oncology | 0.97(0.87,1.09) | 6.46E-01 | 0.864 |
| VWA5A | O00534 | Oncology II | 1.04(0.88,1.24) | 6.46E-01 | 0.864 |
| SLC34A3 | Q8N130 | Oncology II | 0.93(0.70,1.25) | 6.49E-01 | 0.866 |
| OPTC | Q9UBM4 | Oncology | 1.05(0.84,1.33) | 6.61E-01 | 0.878 |
| LHB | P01229 | Oncology | 0.97(0.84,1.11) | 6.63E-01 | 0.878 |
| BTN1A1 | Q13410 | Oncology II | 0.93(0.65,1.31) | 6.63E-01 | 0.878 |
| GPIHBP1 | Q8IV16 | Oncology II | 1.04(0.86,1.26) | 6.63E-01 | 0.878 |
| GLYR1 | Q49A26 | Oncology II | 0.95(0.74,1.21) | 6.65E-01 | 0.878 |
| PCDH7 | O60245 | Oncology II | 1.08(0.75,1.57) | 6.66E-01 | 0.878 |
| RBP5 | P82980 | Oncology | 0.96(0.79,1.17) | 6.68E-01 | 0.878 |
| SMNDC1 | O75940 | Oncology II | 0.95(0.76,1.20) | 6.68E-01 | 0.878 |
| MNAT1 | P51948 | Oncology II | 0.93(0.68,1.29) | 6.70E-01 | 0.878 |
| CXCL8 | P10145 | Oncology | 1.04(0.88,1.22) | 6.71E-01 | 0.878 |
| CAPG | P40121 | Oncology | 1.04(0.86,1.27) | 6.72E-01 | 0.878 |
| XPNPEP2 | O43895 | Oncology | 1.03(0.91,1.15) | 6.74E-01 | 0.878 |
| CINP | Q9BW66 | Oncology II | 0.93(0.68,1.28) | 6.74E-01 | 0.878 |
| APEX1 | P27695 | Oncology | 0.96(0.79,1.16) | 6.75E-01 | 0.878 |
| GAD1 | Q99259 | Oncology II | 1.12(0.66,1.88) | 6.78E-01 | 0.881 |
| SERPINA9 | Q86WD7 | Oncology | 0.97(0.82,1.14) | 6.81E-01 | 0.881 |
| TET2 | Q6N021 | Oncology II | 0.97(0.84,1.12) | 6.81E-01 | 0.881 |
| KLK10 | O43240 | Oncology | 0.94(0.72,1.25) | 6.88E-01 | 0.886 |
| EGFLAM | Q63HQ2 | Oncology II | 1.09(0.72,1.65) | 6.88E-01 | 0.886 |
| MDK | P21741 | Oncology | 1.04(0.84,1.30) | 6.89E-01 | 0.886 |
| NPTN | Q9Y639 | Oncology | 0.97(0.83,1.13) | 6.91E-01 | 0.886 |
| REG3G | Q6UW15 | Oncology II | 0.93(0.67,1.31) | 6.91E-01 | 0.886 |
| PDE1C | Q14123 | Oncology II | 1.05(0.83,1.32) | 6.92E-01 | 0.886 |
| HSPA2 | P54652 | Oncology II | 1.08(0.73,1.61) | 6.94E-01 | 0.887 |
| CEP290 | O15078 | Oncology II | 0.95(0.74,1.23) | 6.96E-01 | 0.888 |
| CD86 | P42081 | Oncology II | 1.09(0.70,1.72) | 6.97E-01 | 0.888 |
| CRNN | Q9UBG3 | Oncology | 1.03(0.87,1.22) | 7.02E-01 | 0.892 |
| HBQ1 | P09105 | Oncology | 1.04(0.87,1.24) | 7.03E-01 | 0.892 |
| CLEC6A | Q6EIG7 | Oncology | 1.05(0.82,1.34) | 7.04E-01 | 0.892 |
| LRRC25 | Q8N386 | Oncology | 0.94(0.70,1.27) | 7.06E-01 | 0.893 |
| SSH3 | Q8TE77 | Oncology II | 1.09(0.70,1.70) | 7.12E-01 | 0.899 |
| SMAD5 | Q99717 | Oncology | 1.11(0.63,1.96) | 7.14E-01 | 0.899 |
| RAB44 | Q7Z6P3 | Oncology II | 0.94(0.68,1.30) | 7.14E-01 | 0.899 |
| PDIA5 | Q14554 | Oncology II | 0.96(0.76,1.21) | 7.16E-01 | 0.899 |
| MME | P08473 | Oncology | 0.97(0.85,1.12) | 7.17E-01 | 0.899 |
| PRDX6 | P30041 | Oncology | 0.97(0.80,1.16) | 7.18E-01 | 0.899 |
| MRPL52 | Q86TS9 | Oncology II | 1.08(0.70,1.68) | 7.22E-01 | 0.903 |
| DDAH1 | O94760 | Oncology | 1.05(0.81,1.36) | 7.25E-01 | 0.905 |
| CRACR2A | Q9BSW2 | Oncology | 1.02(0.90,1.17) | 7.30E-01 | 0.907 |
| SH3GL3 | Q99963 | Oncology II | 0.97(0.83,1.14) | 7.30E-01 | 0.907 |
| RABEPK | Q7Z6M1 | Oncology | 0.96(0.79,1.18) | 7.31E-01 | 0.907 |
| S100A12 | P80511 | Oncology | 1.03(0.88,1.19) | 7.33E-01 | 0.907 |
| ESPL1 | Q14674 | Oncology II | 0.96(0.75,1.23) | 7.34E-01 | 0.907 |
| KIAA2013 | Q8IYS2 | Oncology II | 0.96(0.75,1.23) | 7.34E-01 | 0.907 |
| IFIT3 | O14879 | Oncology II | 0.94(0.65,1.35) | 7.37E-01 | 0.907 |
| PAIP2B | Q9ULR5 | Oncology II | 0.96(0.73,1.24) | 7.37E-01 | 0.907 |
| CIAPIN1 | Q6FI81 | Oncology | 0.97(0.81,1.16) | 7.38E-01 | 0.907 |
| NINJ1 | Q92982 | Oncology | 0.96(0.77,1.20) | 7.42E-01 | 0.910 |
| ABL1 | P00519 | Oncology | 0.97(0.82,1.15) | 7.43E-01 | 0.910 |
| TGFB2 | P61812 | Oncology II | 0.96(0.74,1.23) | 7.44E-01 | 0.910 |
| EIF2AK2 | P19525 | Oncology II | 0.98(0.87,1.11) | 7.48E-01 | 0.913 |
| TPMT | P51580 | Oncology | 0.97(0.83,1.14) | 7.55E-01 | 0.920 |
| EPGN | Q6UW88 | Oncology II | 0.95(0.71,1.29) | 7.57E-01 | 0.921 |
| PSMD9 | O00233 | Oncology | 1.03(0.85,1.26) | 7.59E-01 | 0.922 |
| STXBP3 | O00186 | Oncology | 0.96(0.73,1.26) | 7.65E-01 | 0.928 |
| MZT1 | Q08AG7 | Oncology | 0.97(0.77,1.22) | 7.67E-01 | 0.929 |
| CAMKK1 | Q8N5S9 | Oncology | 1.04(0.80,1.35) | 7.71E-01 | 0.932 |
| ALMS1 | Q8TCU4 | Oncology II | 1.04(0.78,1.40) | 7.72E-01 | 0.932 |
| ERCC1 | P07992 | Oncology II | 0.94(0.63,1.42) | 7.75E-01 | 0.932 |
| IL2RG | P31785 | Oncology II | 1.05(0.75,1.47) | 7.75E-01 | 0.932 |
| ZNRD2 | O60232 | Oncology II | 1.03(0.85,1.24) | 7.77E-01 | 0.933 |
| ARAF | P10398 | Oncology II | 1.03(0.85,1.24) | 7.79E-01 | 0.934 |
| APBB1IP | Q7Z5R6 | Oncology | 0.97(0.75,1.24) | 7.90E-01 | 0.945 |
| DCTN2 | Q13561 | Oncology | 1.02(0.90,1.15) | 7.96E-01 | 0.948 |
| SRP14 | P37108 | Oncology | 0.98(0.81,1.18) | 7.96E-01 | 0.948 |
| GCNT1 | Q02742 | Oncology | 0.96(0.69,1.33) | 7.97E-01 | 0.948 |
| IKZF2 | Q9UKS7 | Oncology | 0.98(0.86,1.12) | 7.98E-01 | 0.948 |
| LEFTY2 | O00292 | Oncology | 1.03(0.82,1.29) | 8.00E-01 | 0.948 |
| DYNC1H1 | Q14204 | Oncology II | 0.96(0.71,1.30) | 8.00E-01 | 0.948 |
| NDUFS6 | O75380 | Oncology | 1.02(0.88,1.18) | 8.01E-01 | 0.948 |
| STX4 | Q12846 | Oncology | 0.98(0.80,1.18) | 8.04E-01 | 0.948 |
| GPRC5C | Q9NQ84 | Oncology II | 0.97(0.75,1.25) | 8.04E-01 | 0.948 |
| DCTN1 | Q14203 | Oncology | 1.02(0.86,1.21) | 8.06E-01 | 0.948 |
| CNTN2 | Q02246 | Oncology | 1.03(0.80,1.33) | 8.08E-01 | 0.948 |
| KHDC3L | Q587J8 | Oncology II | 1.02(0.85,1.24) | 8.08E-01 | 0.948 |
| ARHGAP25 | P42331 | Oncology | 0.98(0.81,1.18) | 8.14E-01 | 0.950 |
| CD300LF | Q8TDQ1 | Oncology | 1.02(0.85,1.23) | 8.14E-01 | 0.950 |
| CA9 | Q16790 | Oncology | 1.03(0.81,1.30) | 8.17E-01 | 0.950 |
| IZUMO1 | Q8IYV9 | Oncology II | 1.03(0.78,1.36) | 8.20E-01 | 0.950 |
| IL9 | P15248 | Oncology II | 1.03(0.82,1.29) | 8.21E-01 | 0.950 |
| TOP1MT | Q969P6 | Oncology II | 1.03(0.79,1.34) | 8.22E-01 | 0.950 |
| TP53 | P04637 | Oncology | 1.03(0.79,1.35) | 8.23E-01 | 0.950 |
| HDDC2 | Q7Z4H3 | Oncology II | 0.97(0.72,1.30) | 8.24E-01 | 0.950 |
| SPINT3 | P49223 | Oncology II | 1.01(0.94,1.09) | 8.24E-01 | 0.950 |
| ID4 | P47928 | Oncology II | 0.96(0.70,1.33) | 8.25E-01 | 0.950 |
| FAM3B | P58499 | Oncology | 1.04(0.75,1.43) | 8.26E-01 | 0.950 |
| CEACAM19 | Q7Z692 | Oncology II | 0.96(0.69,1.34) | 8.28E-01 | 0.950 |
| CD33 | P20138 | Oncology | 1.02(0.88,1.17) | 8.29E-01 | 0.950 |
| CDK1 | P06493 | Oncology II | 1.04(0.72,1.51) | 8.30E-01 | 0.950 |
| RBM25 | P49756 | Oncology II | 1.03(0.77,1.37) | 8.30E-01 | 0.950 |
| RCC1 | P18754 | Oncology II | 0.97(0.74,1.27) | 8.31E-01 | 0.950 |
| PHLDB1 | Q86UU1 | Oncology II | 0.97(0.76,1.25) | 8.32E-01 | 0.950 |
| H2AP | O75409 | Oncology II | 0.97(0.73,1.29) | 8.35E-01 | 0.951 |
| TEX33 | O43247 | Oncology II | 0.96(0.67,1.38) | 8.35E-01 | 0.951 |
| HMMR | O75330 | Oncology II | 1.03(0.79,1.34) | 8.42E-01 | 0.954 |
| FCGR2B | P31994 | Oncology | 1.02(0.84,1.24) | 8.43E-01 | 0.954 |
| COL9A2 | Q14055 | Oncology II | 0.97(0.70,1.33) | 8.43E-01 | 0.954 |
| PBK | Q96KB5 | Oncology II | 0.96(0.62,1.48) | 8.43E-01 | 0.954 |
| PPIE | Q9UNP9 | Oncology II | 1.02(0.83,1.26) | 8.44E-01 | 0.954 |
| LACRT | Q9GZZ8 | Oncology II | 0.97(0.75,1.27) | 8.50E-01 | 0.959 |
| SPRR1B | P22528 | Oncology II | 1.02(0.87,1.19) | 8.52E-01 | 0.960 |
| FGF7 | P21781 | Oncology II | 0.98(0.83,1.17) | 8.55E-01 | 0.960 |
| FOLR3 | P41439 | Oncology | 1.01(0.95,1.07) | 8.56E-01 | 0.960 |
| MYH7B | A7E2Y1 | Oncology II | 1.03(0.76,1.39) | 8.56E-01 | 0.960 |
| KLK6 | Q92876 | Oncology | 0.97(0.67,1.39) | 8.57E-01 | 0.960 |
| CRH | P06850 | Oncology | 1.01(0.89,1.16) | 8.61E-01 | 0.962 |
| GALNT7 | Q86SF2 | Oncology | 1.05(0.61,1.80) | 8.63E-01 | 0.962 |
| PRAME | P78395 | Oncology II | 1.05(0.61,1.81) | 8.63E-01 | 0.962 |
| IFI30 | P13284 | Oncology II | 0.97(0.69,1.36) | 8.66E-01 | 0.962 |
| RARRES1 | P49788 | Oncology | 0.97(0.68,1.39) | 8.67E-01 | 0.962 |
| BTLA | Q7Z6A9 | Oncology II | 1.01(0.87,1.19) | 8.67E-01 | 0.962 |
| UNC79 | Q9P2D8 | Oncology II | 1.02(0.80,1.30) | 8.69E-01 | 0.963 |
| TAP1 | Q03518 | Oncology II | 1.02(0.81,1.29) | 8.71E-01 | 0.963 |
| FEN1 | P39748 | Oncology | 1.01(0.85,1.21) | 8.73E-01 | 0.963 |
| TIGIT | Q495A1 | Oncology II | 0.97(0.67,1.40) | 8.74E-01 | 0.963 |
| GJA8 | P48165 | Oncology II | 1.02(0.82,1.26) | 8.75E-01 | 0.963 |
| BAIAP2 | Q9UQB8 | Oncology | 0.99(0.82,1.18) | 8.76E-01 | 0.963 |
| PCNA | P12004 | Oncology II | 1.02(0.83,1.25) | 8.79E-01 | 0.964 |
| IGLON5 | A6NGN9 | Oncology II | 0.99(0.85,1.15) | 8.83E-01 | 0.964 |
| CLMP | Q9H6B4 | Oncology | 0.96(0.59,1.56) | 8.84E-01 | 0.964 |
| SEZ6L2 | Q6UXD5 | Oncology | 1.04(0.63,1.70) | 8.84E-01 | 0.964 |
| TNFAIP2 | Q03169 | Oncology II | 0.98(0.73,1.31) | 8.85E-01 | 0.964 |
| AGR3 | Q8TD06 | Oncology | 1.01(0.84,1.23) | 8.86E-01 | 0.964 |
| PSCA | O43653 | Oncology II | 1.00(0.95,1.06) | 8.86E-01 | 0.964 |
| SNX18 | Q96RF0 | Oncology II | 1.02(0.75,1.39) | 8.88E-01 | 0.965 |
| RILP | Q96NA2 | Oncology | 1.01(0.84,1.22) | 8.93E-01 | 0.967 |
| IFIT1 | P09914 | Oncology II | 0.98(0.76,1.27) | 8.93E-01 | 0.967 |
| ATF4 | P18848 | Oncology II | 0.98(0.65,1.45) | 9.01E-01 | 0.974 |
| RUVBL1 | Q9Y265 | Oncology | 0.99(0.89,1.11) | 9.02E-01 | 0.974 |
| PVALB | P20472 | Oncology | 1.01(0.91,1.11) | 9.03E-01 | 0.974 |
| PODXL | O00592 | Oncology | 0.96(0.46,2.00) | 9.05E-01 | 0.974 |
| AIF1 | P55008 | Oncology | 0.99(0.79,1.23) | 9.12E-01 | 0.977 |
| WDR46 | O15213 | Oncology II | 1.02(0.71,1.46) | 9.12E-01 | 0.977 |
| TACSTD2 | P09758 | Oncology | 1.02(0.67,1.56) | 9.13E-01 | 0.977 |
| DPY30 | Q9C005 | Oncology | 1.01(0.84,1.22) | 9.15E-01 | 0.977 |
| MCTS1 | Q9ULC4 | Oncology II | 0.97(0.59,1.62) | 9.15E-01 | 0.977 |
| ZP4 | Q12836 | Oncology II | 0.98(0.65,1.48) | 9.15E-01 | 0.977 |
| ATP6AP2 | O75787 | Oncology | 0.99(0.74,1.32) | 9.20E-01 | 0.980 |
| CREBZF | Q9NS37 | Oncology II | 0.98(0.58,1.63) | 9.23E-01 | 0.980 |
| FMR1 | Q06787 | Oncology | 1.02(0.73,1.41) | 9.25E-01 | 0.980 |
| ICOSLG | O75144 | Oncology | 1.03(0.57,1.84) | 9.25E-01 | 0.980 |
| EDDM3B | P56851 | Oncology II | 0.99(0.79,1.24) | 9.25E-01 | 0.980 |
| CEACAM3 | P40198 | Oncology | 0.99(0.77,1.26) | 9.26E-01 | 0.980 |
| ANKRA2 | Q9H9E1 | Oncology II | 1.01(0.80,1.28) | 9.28E-01 | 0.980 |
| DDX1 | Q92499 | Oncology II | 1.01(0.77,1.33) | 9.32E-01 | 0.983 |
| HMBS | P08397 | Oncology | 0.99(0.77,1.28) | 9.34E-01 | 0.984 |
| INSL4 | Q14641 | Oncology II | 0.99(0.75,1.30) | 9.35E-01 | 0.984 |
| CAPN3 | P20807 | Oncology II | 0.99(0.70,1.39) | 9.41E-01 | 0.988 |
| GTF2IRD1 | Q9UHL9 | Oncology II | 0.99(0.82,1.21) | 9.42E-01 | 0.988 |
| FAM171B | Q6P995 | Oncology II | 0.98(0.60,1.61) | 9.45E-01 | 0.990 |
| MPRIP | Q6WCQ1 | Oncology II | 0.99(0.73,1.34) | 9.48E-01 | 0.991 |
| MANEAL | Q5VSG8 | Oncology II | 0.99(0.76,1.30) | 9.51E-01 | 0.993 |
| CEACAM18 | A8MTB9 | Oncology II | 0.99(0.82,1.20) | 9.53E-01 | 0.993 |
| ZBTB16 | Q05516 | Oncology | 1.00(0.85,1.17) | 9.58E-01 | 0.993 |
| MLLT1 | Q03111 | Oncology II | 1.01(0.82,1.23) | 9.58E-01 | 0.993 |
| MAP3K5 | Q99683 | Oncology | 1.00(0.89,1.12) | 9.59E-01 | 0.993 |
| CD3E | P07766 | Oncology II | 1.01(0.70,1.45) | 9.62E-01 | 0.993 |
| CD38 | P28907 | Oncology | 0.99(0.70,1.41) | 9.64E-01 | 0.993 |
| MAEA | Q7L5Y9 | Oncology | 1.01(0.75,1.34) | 9.64E-01 | 0.993 |
| SEMA4C | Q9C0C4 | Oncology | 1.01(0.67,1.52) | 9.65E-01 | 0.993 |
| TLR2 | O60603 | Oncology II | 0.99(0.56,1.75) | 9.66E-01 | 0.993 |
| MIA | Q16674 | Oncology | 1.01(0.74,1.37) | 9.68E-01 | 0.993 |
| BCL7A | Q4VC05 | Oncology II | 1.00(0.81,1.25) | 9.68E-01 | 0.993 |
| ARG1 | P05089 | Oncology | 1.00(0.81,1.24) | 9.69E-01 | 0.993 |
| CEP85 | Q6P2H3 | Oncology | 1.00(0.83,1.19) | 9.70E-01 | 0.993 |
| AKT3 | Q9Y243 | Oncology | 1.00(0.81,1.22) | 9.71E-01 | 0.993 |
| VNN2 | O95498 | Oncology | 1.00(0.78,1.29) | 9.73E-01 | 0.993 |
| CASP4 | P49662 | Oncology II | 1.00(0.92,1.08) | 9.73E-01 | 0.993 |
| CD1C | P29017 | Oncology | 0.99(0.63,1.56) | 9.77E-01 | 0.995 |
| KLK12 | Q9UKR0 | Oncology | 1.00(0.92,1.09) | 9.77E-01 | 0.995 |
| IDUA | P35475 | Oncology | 1.00(0.78,1.27) | 9.80E-01 | 0.996 |
| KIR3DL2 | P43630 | Oncology II | 1.00(0.85,1.18) | 9.83E-01 | 0.997 |
| GATA3 | P23771 | Oncology II | 1.00(0.60,1.67) | 9.87E-01 | 0.997 |
| SLMAP | Q14BN4 | Oncology II | 1.00(0.85,1.17) | 9.87E-01 | 0.997 |
| CD207 | Q9UJ71 | Oncology | 1.00(0.74,1.34) | 9.89E-01 | 0.997 |
| IGF1R | P08069 | Oncology | 1.00(0.58,1.70) | 9.90E-01 | 0.997 |
| RASGRF1 | Q13972 | Oncology II | 1.00(0.73,1.36) | 9.91E-01 | 0.997 |
| CPE | P16870 | Oncology | 1.00(0.74,1.35) | 9.95E-01 | 0.997 |
| THAP12 | O43422 | Oncology II | 1.00(0.86,1.17) | 9.95E-01 | 0.997 |
| NFKBIE | O00221 | Oncology | 1.00(0.79,1.26) | 9.96E-01 | 0.997 |
| ST3GAL1 | Q11201 | Oncology | 1.00(0.76,1.31) | 9.96E-01 | 0.997 |
| UXS1 | Q8NBZ7 | Oncology | 1.00(0.80,1.24) | 9.96E-01 | 0.997 |
| PPME1 | Q9Y570 | Oncology | 1.00(0.83,1.20) | 9.98E-01 | 0.998 |

**^*^**Cox regression model adjusted for age and sex. *P* value was calculated under two-sided tests, and statistical significance was defined as a false discovery rate (FDR)-corrected *P* value <0.05, adjusted for the number of proteins tested (n=730).

**Table S10. The top 10 key proteins in the protein-protein interaction (PPI) network.**

| **Rank** | **Key Nodes (Proteins)** | **UniProt** | ^*^**Betweenness Centrality** | **^†^Closeness Centrality** | ^‡^**Degree Centrality** |
| --- | --- | --- | --- | --- | --- |
| 1 | EGFR | P00533 | 186.47 | 0.31 | 24 |
| 2 | GFAP | P14136 | 65.20 | 0.28 | 14 |
| 3 | CHGA | P10645 | 47.80 | 0.28 | 14 |
| 4 | APOE | P02649 | 8.90 | 0.26 | 6 |
| 5 | VGF | O15240 | 8.67 | 0.26 | 6 |
| 6 | CEACAM5 | P06731 | 5.33 | 0.26 | 6 |
| 7 | SYT1 | P21579 | 2.90 | 0.24 | 6 |
| 8 | CD99L2 | Q8TCZ2 | 1.73 | 0.24 | 6 |
| 9 | ERBB3 | P21860 | 1.00 | 0.25 | 6 |
| 10 | GDF15 | Q99988 | 1.00 | 0.25 | 6 |

^*^Betweenness Centrality (BC): it measures how often a node lies on the shortest path between other nodes in the network. A higher BC value indicates that the node plays a more significant role in connecting different parts of the network.

^†^Closeness Centrality (CC): it measures how close a node is to all other nodes in the network. A higher CC value indicates that the node is located closer to other nodes in the network, enabling faster communication and interactions.

^‡^Degree Centrality (DC): it simply counts the number of links a node has to other nodes in the network. A higher DC value means that the node has more direct connections or interactions with other nodes in the network.
